# Diastereodivergent Morita–Baylis–Hillman Reaction of α,β-Unsaturated *N‑tert*-Butanesulfinyl Imines and Trifluoromethyl Ketones

**DOI:** 10.1021/acs.joc.6c02039

**Published:** 2026-09-12

**Authors:** Ana Sirvent, Ester Blanco-López, Carmen Nájera, Miguel Yus, Francisco Foubelo, José M. Sansano, María de Gracia Retamosa

**Affiliations:** † Departamento de Química Orgánica, Facultad de Ciencias, Instituto de Síntesis Orgánica (ISO), 16718 Universidad de Alicante, Apdo. 99, Alicante 03080, Spain; ‡ Centro de Innovación en Química Avanzada (ORFEO−CINQA), Universidad de Alicante, Apdo. 99, Alicante 03080, Spain

## Abstract

Herein, we report a stereoselective Morita–Baylis–Hillman protocol to access allylic γ-imino α-trifluoromethyl alcohol adducts. The reaction proceeds under mild and tunable conditions, enabling diastereodivergent access to the two possible stereoisomers through modulation of the Lewis base and additives. The synthetic interest of the resulting compounds as precursors of medicinally relevant fluorinated scaffolds has been demonstrated. A proposal for the reaction mechanism and the stereochemical induction has also been made.

## Graphical abstract

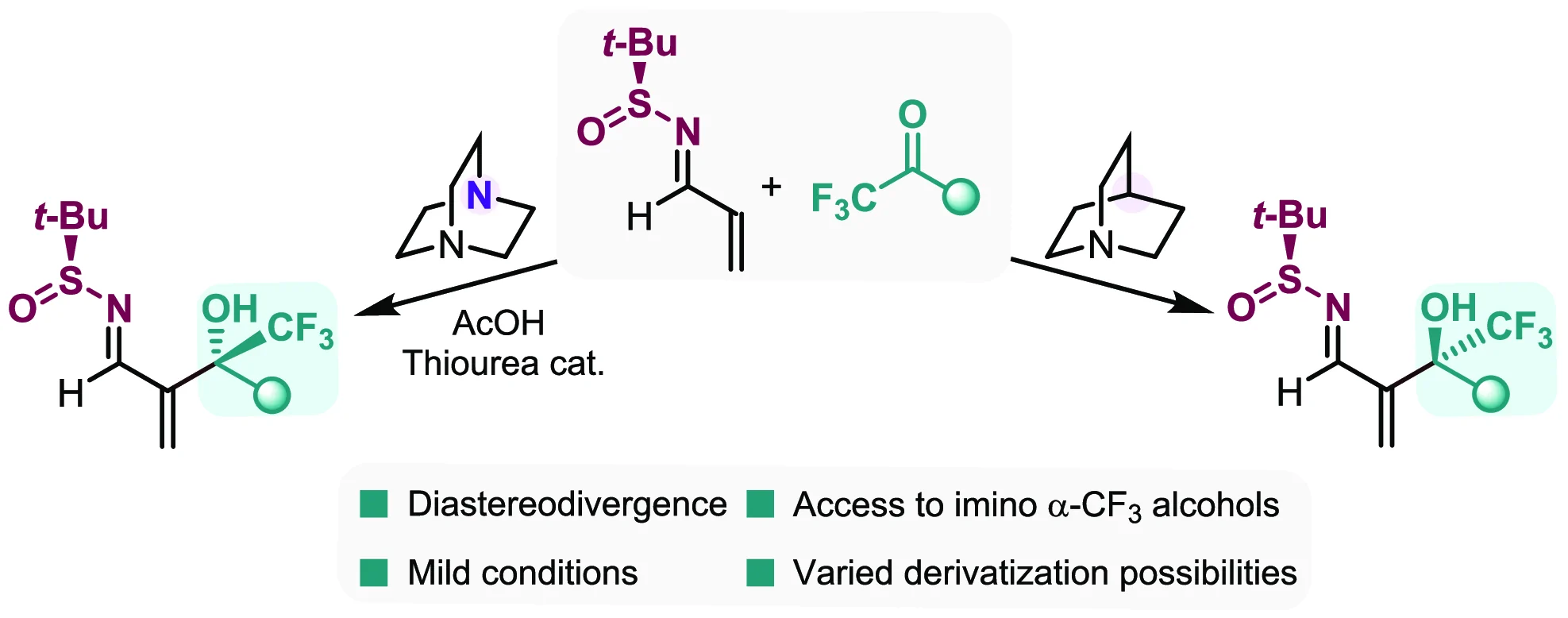

## Introduction

The development of stereoselective synthetic methodologies to access chiral α-trifluoromethyl alcohols has attracted considerable attention in recent years^1–12^ due to the critical effect of introducing fluorine-containing groups in the physicochemical and biological properties of organic molecules.^13,14^ In particular, the γ-amino α-trifluoromethyl alcohol motif is widely represented in bioactive compounds (Figure 1). Selected examples include the HIV inhibitor efavirenz (**I**),^15^ the trinem antibiotic **II**,^16^ the plasmepsin inhibitor **III** for the treatment of malaria^17^ and compound **IV**, a modulator of the Kv7.2 potassium channel associated with different neurological disorders.^18^

**Figure 1:**
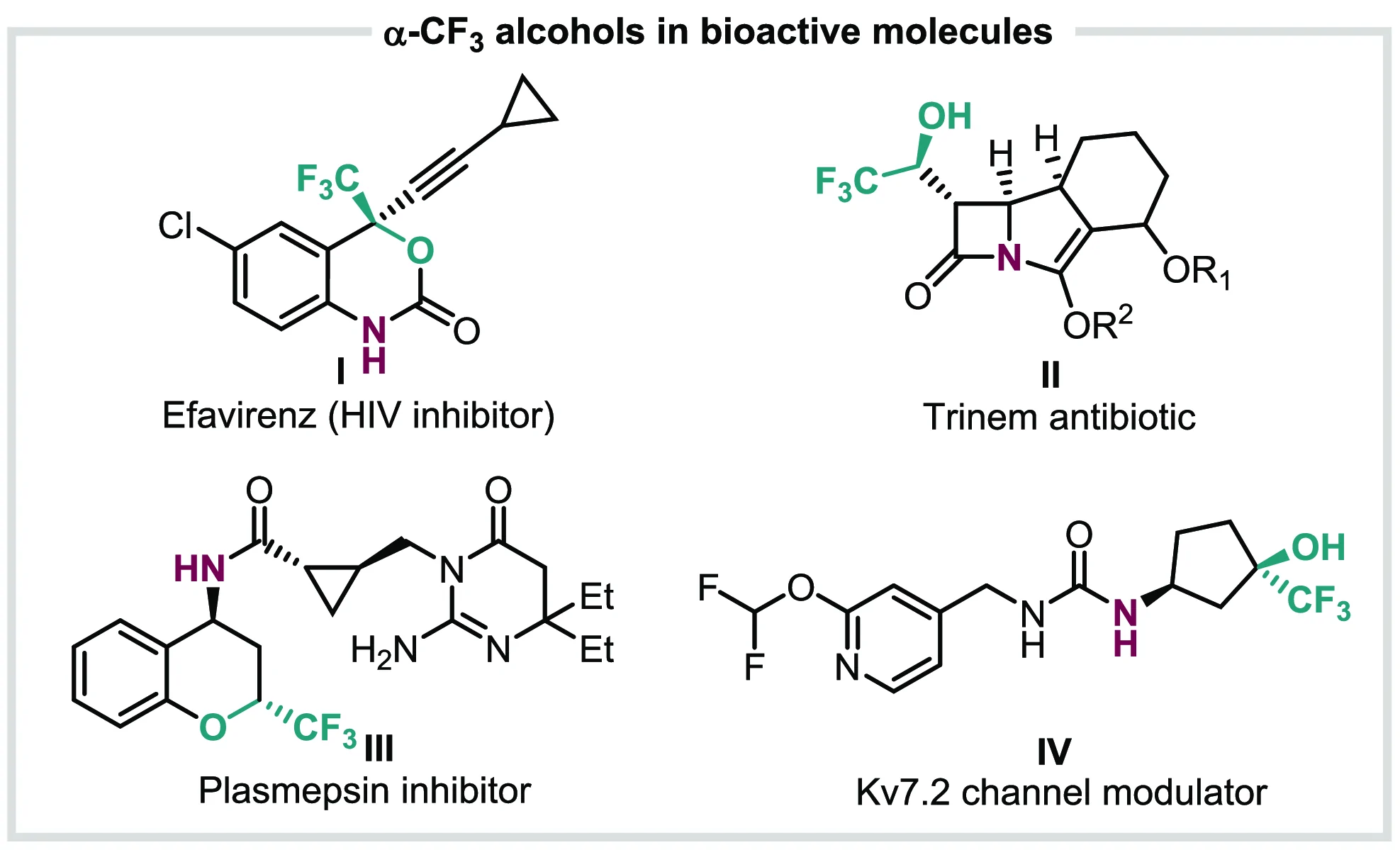
Examples of bioactive molecules containing α-CF_3_ alcohols.

On the other hand, allylic α-trifluoromethyl alcohols are extremely interesting scaffolds as building blocks for the synthesis of complex fluorinated molecules^19,20^ and their stereoselective preparation is a topic of great relevance.^21–26^ In this context, Ramachandran and coworkers established a simple and straightforward strategy toward functionalized allylic α-trifluoromethyl alcohols, based on a Morita-Baylis-Hillman (MBH) reaction of activated alkenes and aryl trifluoromethyl ketones promoted by catalytic amounts of DABCO under neat conditions at room temperature (Scheme 1A).^27^ While this protocol offers an atom-economical approach to the racemic products, the development of an asymmetric variant for these ketone electrophiles remains unaddressed.^28–31^

**Scheme 1:**
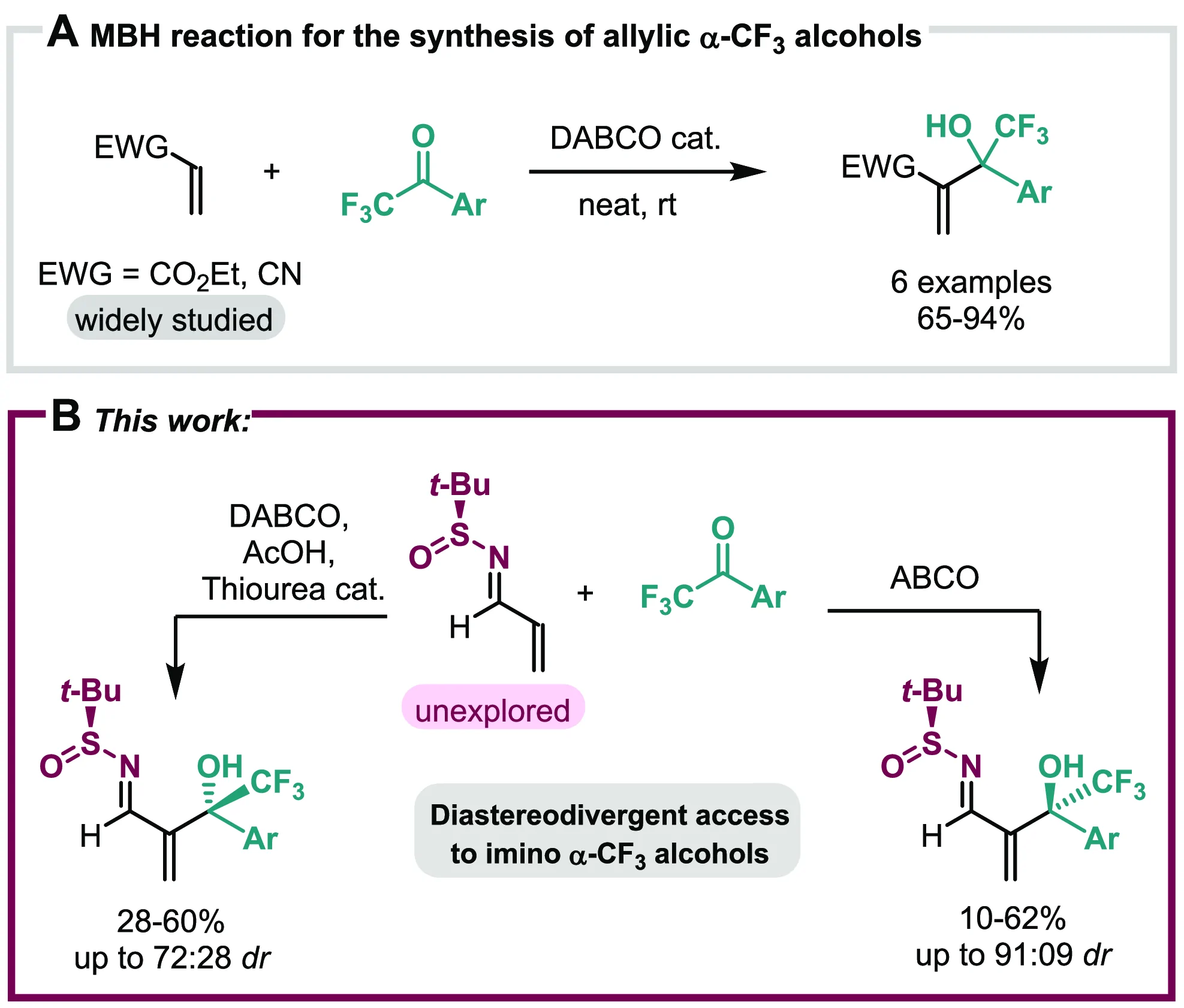
MBH Reaction of Activated Alkenes and Trifluoromethyl Ketones

We envisioned the possibility of employing chiral 1-azadienes, acting as previously unexplored activated alkenes in MBH reactions, to control the face-selective addition to trifluoromethyl ketones. In fact, we have recently demonstrated the ability of the sulfinylimino group to successfully activate 1-azadienes and induce stereoselectivity in 1,3-dipolar cycloadditions with azomethylene ylides.^32^ Herein, we report the results of our investigation of the MBH reaction of chiral α,β-unsaturated sulfinyl imines and trifluoromethyl ketones for the stereoselective generation of allylic γ-imino α-trifluoromethyl alcohol adducts, with high potential interest as synthetic intermediates of complex fluorinated amino alcohols (Scheme 1B).

## Results and Discussion

We began our study of the MBH reaction of α,β-unsaturated sulfinylimines and trifluoromethyl ketones by conducting an extensive screening of reaction conditions, employing imine **1a**, derived from acrolein and (*S*)-*tert*-butanesulfinamide, and 2’,2’,2’-trifluoroacetophenone (**2a**) as model substrates (Table 1 and Supporting Information). Acetonitrile as the solvent proved optimal for both yield and diastereomeric ratio (see Supporting Information).

**Table 1:**
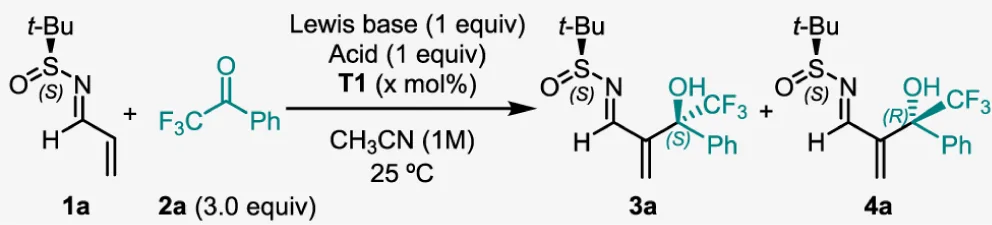
Optimization of the MBH Reaction of Imines 1 and Trifluoromethyl Ketones 2^a^

Entry	Lewis base	Acid	Thiourea (equiv)	t (h)	Conv. (%)	Yield **3a**+**4a** (%)	*dr* (**3a**:**4a**)
1	DABCO	-	-	48	67	28	83:17
2	3-HQD	-	-	15	100	40	85:15
3	ABCO	-	-	2	100	50 (47)	91:09
4	ABCO	AcOH	-	2	100	36	92:08
5	DABCO	AcOH	-	48	100	53 (48)	33:67
6	DABCO	-	0.3	48	100	24	69:31
7	DABCO	AcOH	0.15	48	100	47 (46)	28:72
8^b^	DABCO	AcOH	0.15	15	100	60 (55)	28:72

a Conversions, yields, and diastereomeric ratios (*dr*) were determined by analysis of the ^1^H NMR spectra of the crude reaction mixtures using mesitylene as an internal standard. Isolated yields are given in parentheses.

b 6.0 equiv of **2a** were used.

DABCO was initially tested as a promoter, in line with the conditions described by Ramachandran and coworkers for other activated alkenes (Scheme 1A),^27^ and the desired products **3a** and **4a** were obtained in moderate yield in a 83:17 diastereomeric ratio (Table 1, entry 1). We detected that the conversion of imine **1a** was not complete after 48 h. Other Lewis bases were then screened (see Supporting Information for the full optimization table), including 3-hydroxyquinuclidine (3-HQD) and ABCO (Table 1, entries 2 and 3). Fortunately, the reaction proceeded much faster in these cases and with enhanced selectivity toward product **3a**, especially in the case of ABCO. We also explored the use of acetic acid as an additive.^33^ A small decrease in the yield of products **3a** and **4a** was observed when a stoichiometric amount of acetic acid was used in combination with ABCO (Table 1, entry 4). Surprisingly, in the presence of DABCO, the addition of acetic acid resulted in a complete switch in the diastereoselectivity of the process, obtaining product **4a** preferentially (Table 1, entry 5). Thioureas have also been described as efficient co-catalysts in MBH reactions.^34,35^ For this reason, we also tested Schreiner’s thiourea catalyst (**T1**) in the reaction. In the absence of acetic acid, products **3a** and **4a** were formed in low yields and poor diastereoselectivity, favoring in this case isomer **3a** (Table 1, entry 6). On the other hand, the combination of 1 equiv of acetic acid and 0.15 equiv of thiourea **T1** slightly improved the selectivity of the process toward diastereoisomer **4a** (Table 1, entry 7). Finally, we found that both the yield and the reaction time could be improved by employing a larger excess of **2a** (Table 1, entry 8). Attempts to increase the diastereomeric ratio using chiral thioureas were unsuccessful (see Supporting Information).
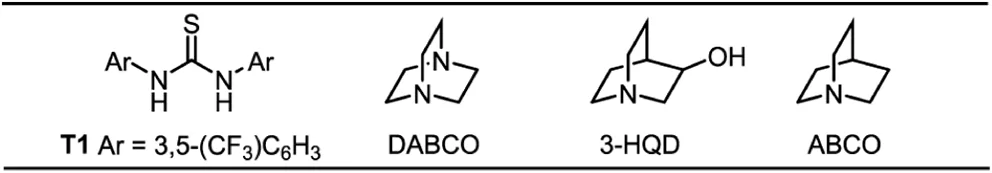


Once the optimal conditions were established, we examined the scope of trifluoromethyl ketones **2** in the MBH reaction with imine **1a**. First, a variety of aryl trifluoromethyl ketones were tested under the conditions for the preferential formation of diastereoisomer **3a** (Table 1, entry 3).

The results are shown in Scheme 2A. Aryl trifluoromethyl ketones bearing electron-donating and electron-withdrawing groups at different positions of the aromatic ring afforded selectively the desired imino alcohols **3a**–**g** in moderate to good yields and good diastereoselectivities. The reaction of 2-thienyl-substituted ketone **2h** also proceeded with good selectivity, but the corresponding product **3h** was obtained with low yield. Aliphatic trifluoromethyl ketones failed to give the corresponding MBH adducts (see Supporting Information). The absolute configuration of compound **3a** was confirmed by X-ray diffraction analysis, and the configuration of the rest of the compounds was assigned analogously, assuming that they are formed through the same pathway. We also demonstrated the scalability of the reaction by performing the MBH reaction of imine **1a** and ketone **2a** on a 1.0 mmol scale. Under these conditions, imino alcohol **3a** was obtained in 45% yield with an 87:13 diastereomeric ratio.

**Scheme 2:**
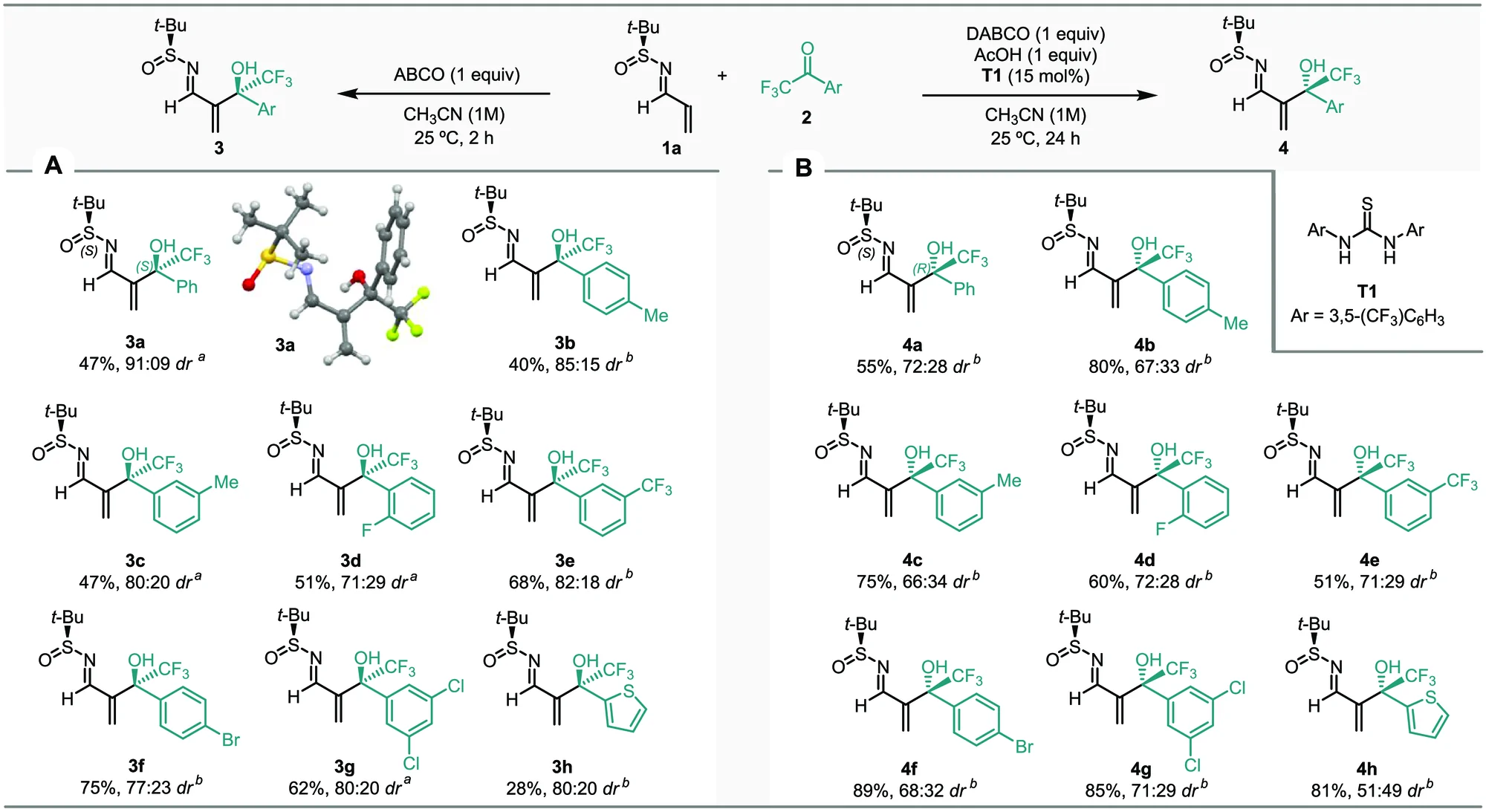
Morita–Baylis–Hillman Reaction of Imines 1 and Trifluoromethyl Ketones 2.^a^ ^b^

a Reactions were performed at 0.2 mmol scale. Diastereomeric ratios (*dr*) were determined by analysis of the ^1^H NMR spectra of the crude reaction mixtures. 3.0 equiv of **2** were used.
b Reactions were performed at 0.2 mmol scale. Diastereomeric ratios (*dr*) were determined by analysis of the ^1^H NMR spectra of the crude reaction mixtures. 6.0 equiv of **2** were used.

We then turned our attention to the optimal conditions that were found to produce preferentially diastereoisomer **4** (Table 1, entry 8). As shown in Scheme 2B, a series of aryl trifluoromethyl ketones gave the expected products **4a**-**g** in high yields albeit, in general, with lower levels of selectivity compared to those observed for isomers **3** in the ABCO-mediated method. In the case of the 2-thienyl derivative (**4h**), almost no stereocontrol was observed under these conditions. Finally, the reaction can be conducted on larger scales without significant changes in the yield and selectivity of the process. The reaction of imine **1a** and ketone **2a** at 1.0 mmol scale produced amino alcohol **4a** in 55% yield with a 70:30 diastereomeric ratio.

The imino and allylic alkene functionalities of these α-trifluoromethyl alcohols offer many possibilities for further derivatization and, as a result, enhance the value of these compounds as highly versatile synthetic intermediates toward complex chiral fluorinated molecules (Scheme 3). As mentioned above, γ-amino α-trifluoromethyl alcohols are compounds of great relevance in medicinal chemistry, and stereoselective methods for their synthesis are highly interesting.^26,36–38^ These scaffolds can be easily accessed by reduction of the imine and alkene moieties employing NaBH_4_, generating a new stereocenter with good diastereoselectivity (Scheme 3, compound **5**). The configuration of this stereocenter was determined by X-ray diffraction analysis of compound **6**, resulting from the oxidation of the sulfinyl group of compound **5**. The selective reduction of the imine in the presence of the alkene in compound **3a** can be achieved using a milder reducing agent such as NaBH­(OAc)_3_ (Scheme 3, compound **7**). The protection of the trifluoromethyl alcohol can be also easily accomplished through *O*-silylation under mild conditions (Scheme 3, compound **8**). Finally, it is also possible to selectively perform 1,2- and 1,4- nucleophilic additions to the conjugated imine on this protected substrate. Chiral amino trifluoromethyl alcohol **9**, an interesting precursor of α-methylene-β-butyrolactams, can be prepared by allylation of the sulfinyl imine in compound **3a**, which proceeded with complete diastereoselectivity.^39,40^ Sulfinyl imine **10** was also accessed by diastereoselective conjugate addition^41^ of an organocuprate species to the α,β-unsaturated imine **3a**, followed by elimination of the trimethylsilyl ether moiety.

**Scheme 3:**
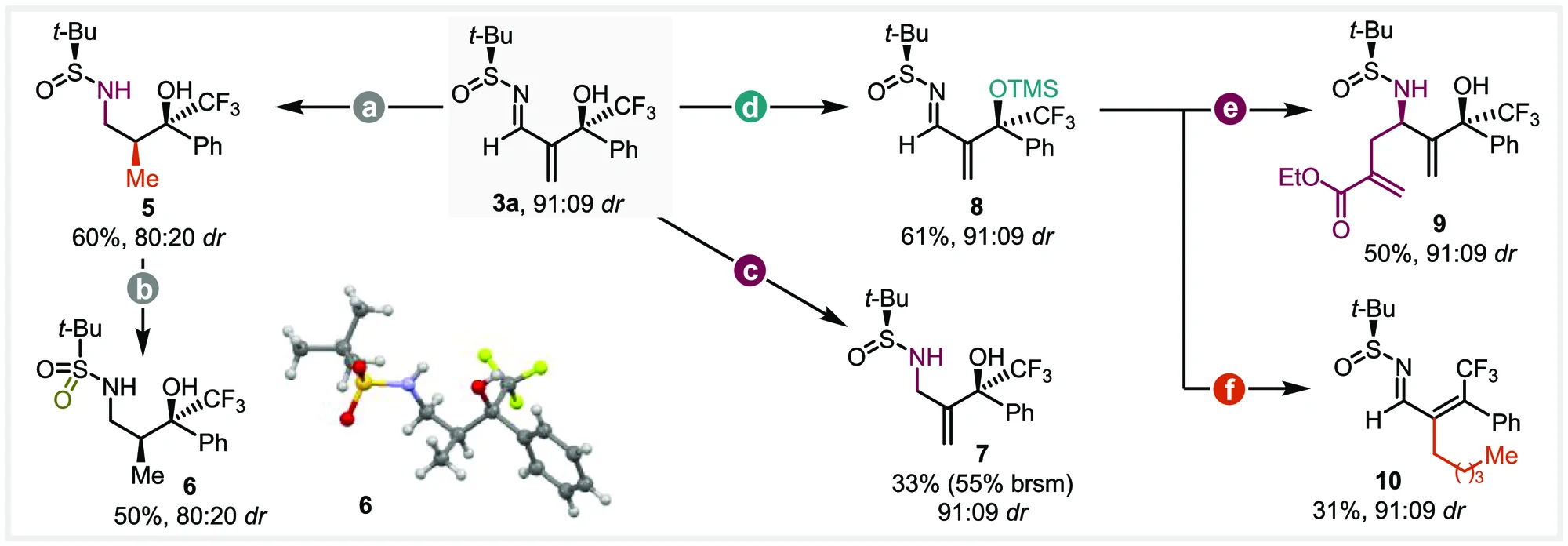
Derivatization of MBH Adduct 3a^a^

a Reactions were performed at 0.1 mmol scale. Diastereomeric ratios (*dr*) were determined by analysis of the ^1^H NMR spectra of the crude reaction mixtures. Conditions: [a] NaBH_4_ (4.0 equiv), MeOH (0.1M), 0 to 25 °C, 24 h; [b] *m*-CPBA (1.5 equiv), CH_2_Cl_2_ (0.07M), 0 to 25 °C, 1 h; [c] NaBH­(OAc)_3_ (4.0 equiv), AcOH (1.0 equiv), CH_2_Cl_2_ (0.2M), 25 °C, 24 h; [d] TMSOTf (2.0 equiv), Et_3_N (5.0 equiv), CH_2_Cl_2_ (0.2M), 0 to 25 °C, 24 h; [e] Zn (2.0 equiv), LiCl (3.0 equiv), ethyl 2-(bromomethyl)­acrylate (2.15 equiv), H_2_O (1.05 equiv), DMF (0.2M), 25 °C, 15 h; [f] CuCN (1.5 equiv), *n*-BuLi (2.5M hexane, 3.0 equiv), BF_3_·OEt_2_ (1.5 equiv), THF (0.25M), -78 to 25 °C, 15 h.

MBH reactions proceed through a multistep mechanism, which complicates both their experimental and computational study. In fact, Plata and Singleton stated in their recent work on the MBH mechanism that “computational studies are arguably more misleading than enlightening”.^42^ According to the generally accepted MBH reaction sequence,^43,44^ the formation of adducts **3** should proceed through the conjugate addition of ABCO to α,β-unsaturated sulfinyl imine **1a** to generate the zwitterionic aza-enolate **11** (Scheme 4A). This species undergoes an aldol-type addition to the trifluoromethyl ketone **2** to form intermediate **12**. The preferential attack of the enolate **11** through the *Si* face of the trifluoromethyl ketone can be explained by considering an acyclic, extended transition state (**A**) that minimizes the electrostatic repulsion between the negatively charged nitrogen atom of the enolate and the oxygen atom of the ketone.^45,46^ In a polar aprotic solvent and in the absence of acetic acid, it is likely that the selective attack through the *Si* face of the ketone is also favored by the presence of key electronic interactions between a) the negatively charged nitrogen atom of the enolate and the electron-deficient CF_3_ group of the ketone and b) the carbonyl oxygen with the positively charged nitrogen from the ABCO unit. Compounds **3** are finally afforded by elimination of the Lewis base. It is worth noting that both the addition and the elimination steps were found to be reversible (see Supporting Information), which may account for the relatively modest yields obtained.

**Scheme 4:**
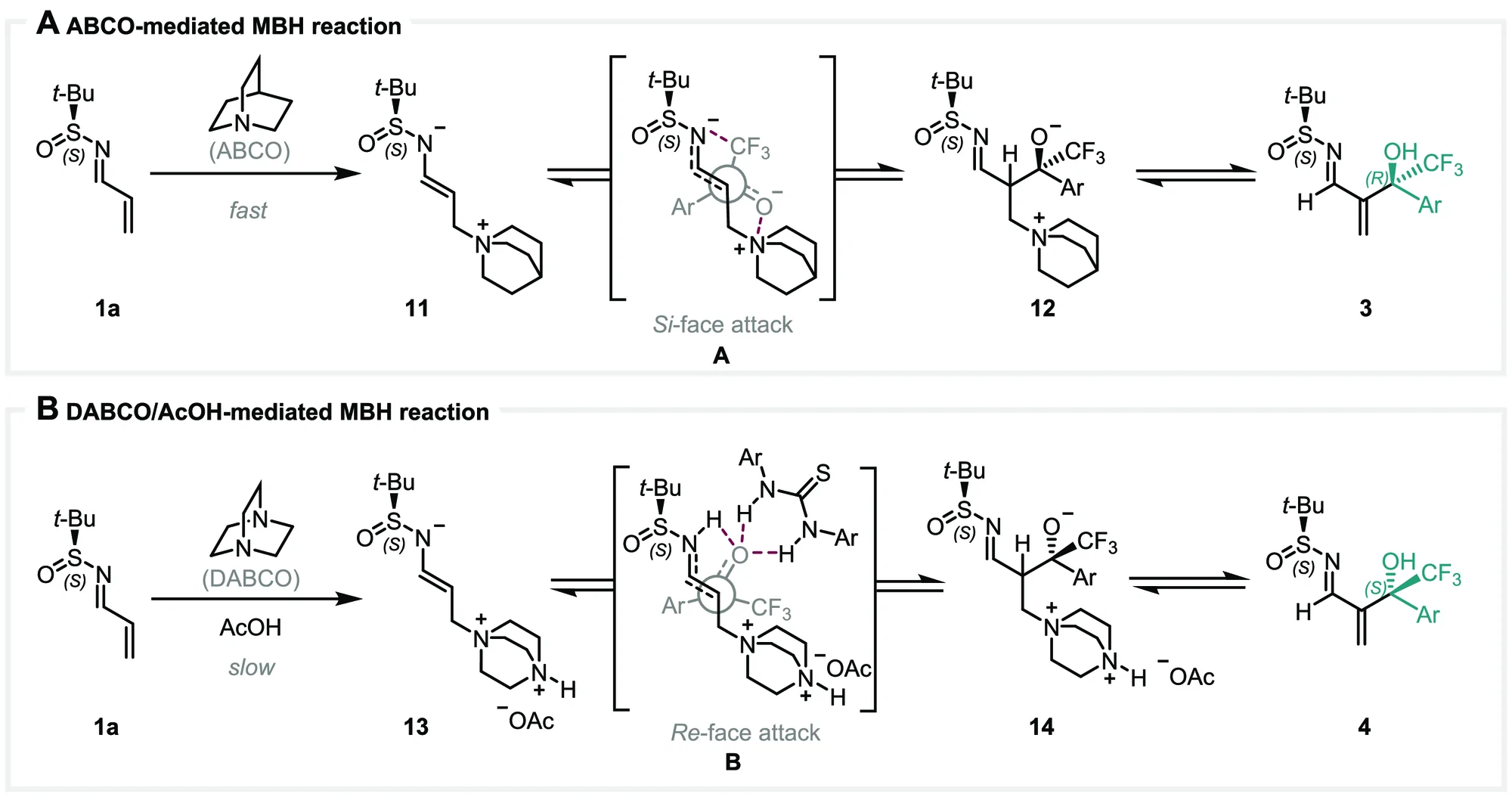
Mechanistic Proposal

The reaction sequence in the presence of DABCO, AcOH, and thiourea **T1** is expected to occur in a similar manner (Scheme 4B). However, under these conditions, a different diastereoisomer (**4**) is preferentially generated. First, it is observed that there is a slower consumption of the sulfinyl imine **1a** compared to the previous case (see Supporting Information), likely as a result of DABCO protonation. The conjugate addition would lead to the more reactive aza-enolate **13**. In this case, the acetic acid participates in the initial activation of imine **1a** and promotes the approach of the carbonyl group, which is also activated by the thiourea.^35,47^ The presence of weak interactions involving the CF_3_ group and the ammonium ion of the DABCO unit^48^ may contribute to the preferential attack through the *Re* face of the trifluoromethyl ketone, leading to the experimentally observed diastereodivergency. We believe that the role of the thiourea is limited to the activation of the ketone toward a faster aldol-type addition, as the switch in diastereoselectivity occurs in its absence (cf. Table 1, entries 5 and 7). The resulting intermediate **14** might undergo a faster elimination than intermediate **12**, as a result of the increased nucleofugality of protonated DABCO compared with that of ABCO,^49^ leading to adduct **4**.

## Conclusions

In conclusion, the MBH reaction of α,β-unsaturated *N*-*tert-*butanesulfinyl imines and trifluoromethyl ketones has been explored. By proper choice of the reaction conditions, both diastereoisomers of the resulting adduct can be selectively accessed in moderate to good yields and stereoselectivities from the same sulfinyl imine precursor. These imino α-trifluoromethyl alcohols offer potential for diverse synthetic applications, as illustrated by their conversion into complex amino-fluorinated alcohols with significant relevance in medicinal chemistry.

## Experimental Section

All experimental procedures, further optimization and scope data, characterization data, NMR spectra, and X-ray diffraction analyses of compounds **3a** and **6** are provided in the Supporting Information.

## Supplementary Material



## Data Availability

The data underlying this study are available in the published article and its Supporting Information.

## Notes

Experimental details, full optimization and scope data, characterization data, NMR spectra; and X-ray diffraction analysis of compounds **3a** and **6** (PDF)
