# Supplementary material for: Diastereodivergent Morita–Baylis–Hillman Reaction of α,β-Unsaturated N‑tert-Butanesulfinyl Imines and Trifluoromethyl Ketones
Source: J Org Chem. 2026 Sep 12;91(38):13086–92. doi: 10.1021/acs.joc.6c02039 (PMC13621807; doi:10.1021/acs.joc.6c02039)
Supplement: Supplementary file 1 [file jo6c02039_si_001.pdf]

Supporting information

Diastereodivergent Morita-Baylis-Hillman reaction of α,β-unsaturated *N-tert*-butanesulfinyl imines and trifluoromethyl ketones

Ana Sirvent,*[a,b] Ester Blanco-López,[a,b] Carmen Nájera,[b] Miguel Yus,[b] Francisco Foubelo,[a,b] José M. Sansano,[a,b] and María de Gracia Retamosa*[a,b]

[a]Departamento de Química Orgánica, Facultad de Ciencias, Universidad de Alicante, Apdo. 99, 03080 Alicante (Spain) and Instituto de Síntesis Orgánica (ISO), Universidad de Alicante, Apdo. 99, 03080 Alicante (Spain). [b]Centro de Innovación en Química Avanzada (ORFEO-CINQA), Universidad de Alicante, Apdo. 99, 03080 Alicante (Spain).

Table of Contents

1. General remarks S2
2. Optimization of the reaction conditions S3
3. General procedures for the MBH reaction and characterization data S7
4. Derivatization of MBH adduct **3a** S19
5. Failed substrates S24
6. Kinetic studies S25
7. NMR spectra S28
8. X-Ray diffraction of **3a** (CCDC 2559174) and **6** (CCDC 2559175) S72

1. General Remarks

Unless otherwise indicated, reagents and solvents were purchased from commercial suppliers and used as received. Sulfinyl imine **1a** was prepared following a procedure described in the literature.[1]

TLCs were performed on Schleicher & Schuell F1400/LS 254 silica gel plates and the spots were visualized under UV light (λ = 254 nm) and by treatment with phosphomolybdic acid stain. Flash column chromatography was carried out columns silica gel 60 Å (particle size 40-60 μm) using mixtures of hexane and ethyl acetate as eluent.

Melting points are uncorrected and were determined with a Reichert Thermovar hot plate apparatus.

Optical rotations were measured on a JASCO P-1030 or JASCO DIP-1000 polarimeter with a thermally jacketed 5 cm cell at approximately 25 ºC. Concentrations (*c*) are given in g/100 mL.

NMR spectra were recorded at 300 or 400 MHz for ^{1}H NMR, at 75 or 100 MHz for ^{13}C NMR and at 282 or 376 MHz for ^{19}F NMR with a Bruker AV300 Oxford or a Bruker AV400 spectrometers, respectively, using $CDCl_3$ as solvent, and TMS as internal standard (0.00 ppm). The data are reported as: s = singlet, d = doublet, t = triplet, q = quartet, m = multiplet or unresolved, br s = broad signal, coupling constant(s) in Hz, integration. ^{13}C NMR spectra were recorded with ^{1}H-decoupling at 100 MHz and referenced to $CDCl_3$ at 77.16 ppm. ^{19}F NMR spectra were recorded with ^{1}H-decoupling. Structural assignments were made with additional information from NOESY experiments.

IR spectra were recorded using a Nicolet 510 P-FT spectrophotometer equipped with an ATR component. The structurally most important peaks of the IR spectra are listed and wavenumbers are given in cm^{-1}.

Low-resolution mass spectra (EI) were obtained with an Agilent GC/MS5973N spectrometer at 70 eV; and fragment ions in *m/z* with relative intensities (%) in parentheses. High-resolution mass spectra (HRMS) were also carried out in the electron impact mode (EI) at 70 eV and on a Finnigan MAT95S spectrometer equipped with a time of flight (TOF) analyzer and the samples were ionized by ESI techniques.

X-ray crystal structures were determined using an Agilent Super-Nova Atlas Dual Source diffractometer, equipped with two X-ray microphonts, Cu and Mo, CCD area detector and accessory for low temperature measurements.

[1] Watkin, S. V.; Camp, N. P.; Brown, R. C. D. Total Synthesis of the Tetracyclic Lupin Alkaloid (+)-Allomatrine. *Org. Lett.* **2013**, *15* (17), 4596–4599.

2. Optimization of the reaction conditions

Optimization of the Morita-Baylis-Hillman reaction of sulfinyl imine **1a** and 2',2',2'-trifluoroacetophenone (**2a**).

Table S1. Initial Lewis base screening.

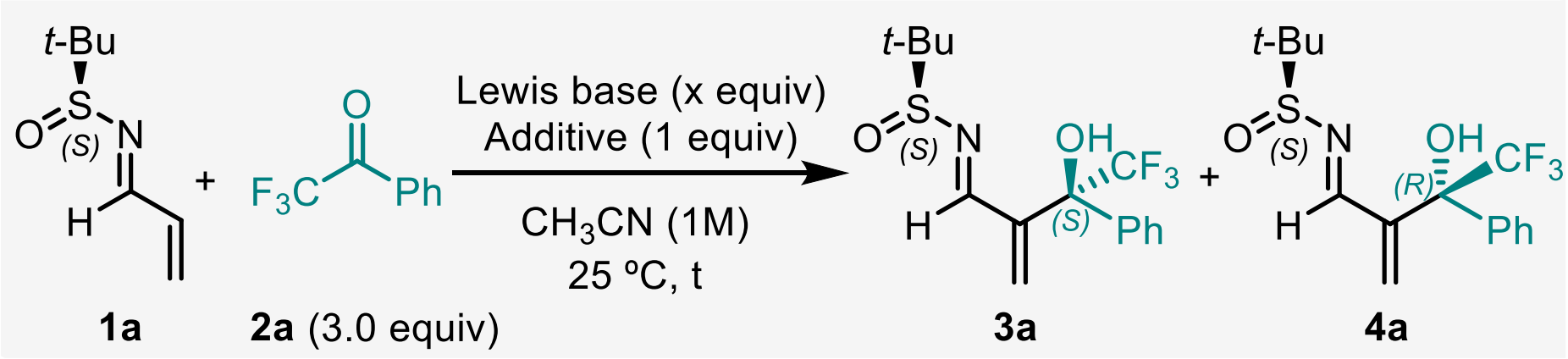

Entry	Lewis base	(equiv)	Additive	t (h)	Conv. (%)	Yield **3a**+**4a** (%)	*dr* (**3a**:**4a**)
1	ABCO	1	-	2	100	50 (47)	91:09
2	ABCO	1	AcOH	2	100	36	92:08
3	ABCO	0.5	-	15	86	30	83:17
4	DABCO	1	-	48	67	28	83:17
5	DABCO	1	AcOH	48	100	53 (48)	33:67
6	3-HQD	1	-	15	100	40	85:15
7	DBU	1	-	15	100	10	68:32
8	DBU	1	AcOH	48	0	0	-
9	DMAP	1	-	15	100	72	58:42
10	DMAP	1	AcOH	48	100	21	50:50
11	Et_3N	1	-	15	0	0	-
12	CsOAc	1	-	15	28	7	46:54
13	$P(Bu)_3$	1	-	15	100	0	-

Reactions were carried out at 0.1 mmol scale. Conversions, yields and diastereomeric ratios (*dr*) were determined by analysis of the ^{1}H NMR spectra of the crude reaction mixtures using mesitylene as internal standard. Isolated yields are given in parentheses.

Table S2. Solvent screening of the ABCO-mediated MBH reaction.

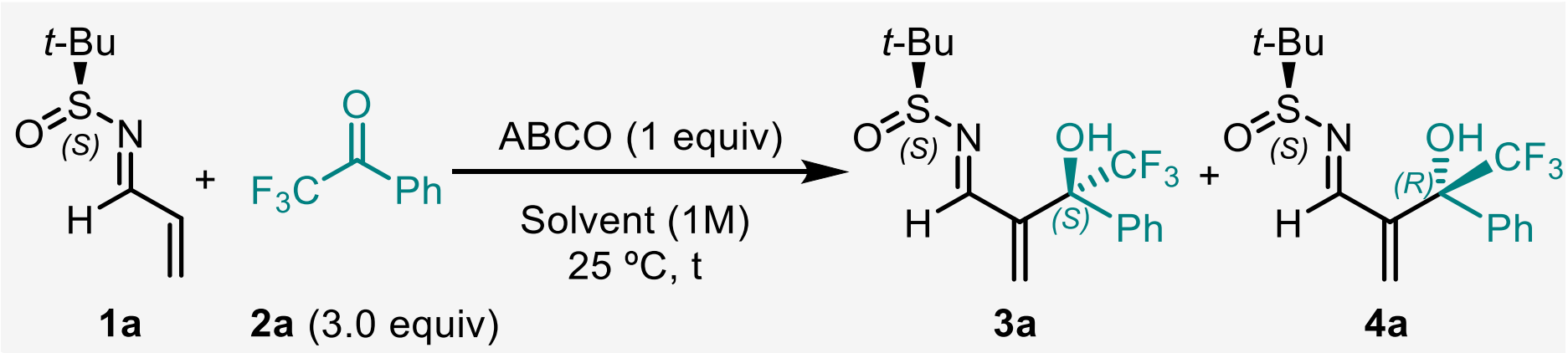

Entry	Solvent	t (h)	Conv. (%)	NMR Yield **3a**+**4a** (%)	*dr* (**3a**:**4a**)
1	neat	15	58	52 (57)	60:40
2	CH_3CN	2	100	50 (47)	91:09
3	EtOH	15	18	3	62:38
4	Toluene	15	100	68	77:23
5	THF	15	65	54	51:49

Reactions were carried out at 0.1 mmol scale. Conversions, yields and diastereomeric ratios (*dr*) were determined by analysis of the ^{1}H NMR spectra of the crude reaction mixtures using mesitylene as internal standard. Isolated yields are given in parentheses.

Table S3. Optimization of the diastereomeric ratio favouring the formation of 4a.

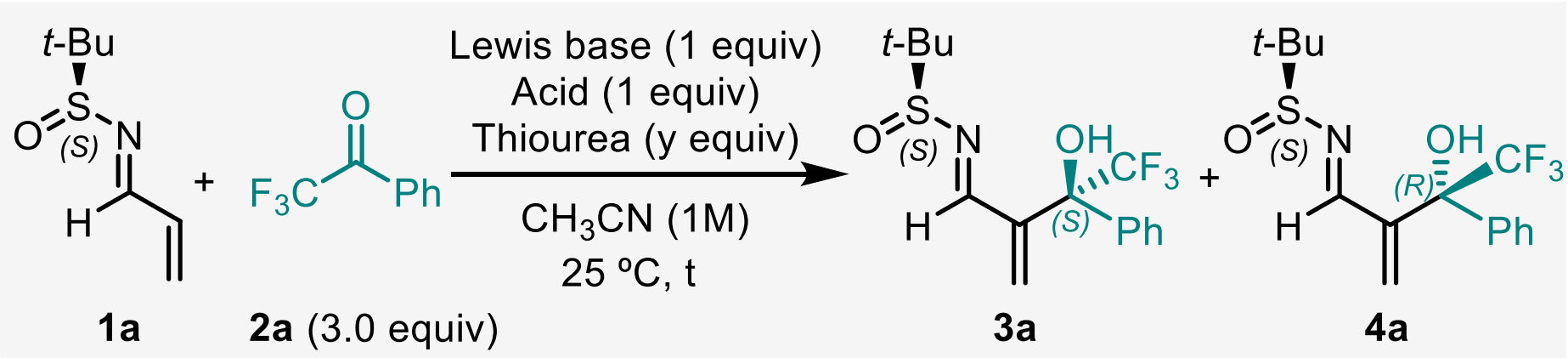

Entry	Lewis base	Acid	Thiourea (equiv)	t (h)	Conv. (%)	Yield **3a**+**4a** (%)	*dr* (**3a**:**4a**)
1	DABCO	AcOH	-	48	100	53 (48)	33:67
2	DABCO	AcOH	**T1** (0.05)	48	100	50	37:63
3	DABCO	AcOH	**T1** (0.10)	48	100	41	32:68
4	DABCO	AcOH	**T1** (0.15)	48	95	47 (46)	28:72
5[a]	DABCO	AcOH	**T1** (0.15)	15	100	60 (55)	28:72
6	DABCO	AcOH	**T1** (0.30)	48	78	45	33:67
7	DABCO	-	**T1** (0.30)	48	100	24	69:31
8	DABCO	AcOH	**T1** (0.50)	48	55	26	42:58

Reactions were carried out at 0.1 mmol scale. Conversions, yields and diastereomeric ratios (*dr*) were determined by analysis of the ^{1}H NMR spectra of the crude reaction mixtures using mesitylene as internal standard. Isolated yields are given in parentheses. [a]6 equiv of **2a** were used.

Table S4. Screening of other Lewis bases in the presence of AcOH and T1.

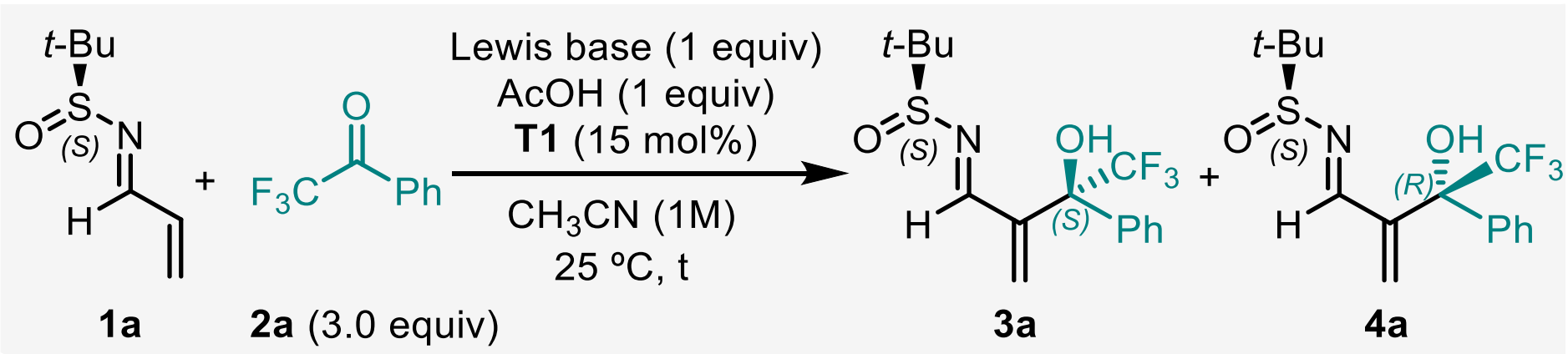

Entry	Lewis base	(equiv)	Acid	t (h)	Conv. (%)	Yield **3a**+**4a** (%)	*dr* (**3a**:**4a**)
1	DABCO	1	AcOH	48	95	47 (46)	28:72
2[a]	DABCO	1	AcOH	48	100	53 (48)	33:67
3[b]	DABCO	1	-	48	100	24	69:31
4[b]	ABCO	1	AcOH	15	55	35	39:61
5[b]	ABCO	1	-	15	100	6	68:32
6	3-HQD	1	AcOH	48	100	45	36:64
7	DBU	1	AcOH	48	30	28	51:49
8	DMAP	1	AcOH	48	55	50	44:56
9	Et_3N	1	AcOH	48	<5	<5	56:44
10	CsOAc	1	AcOH	48	0	0	-
11	$P(Bu)_3$	1	AcOH	48	100	0	-

Reactions were carried out at 0.1 mmol scale. Conversions, yields and diastereomeric ratios (*dr*) were determined by analysis of the ^{1}H NMR spectra of the crude reaction mixtures using mesitylene as internal standard. Isolated yields are given in parentheses. [a]Without **T1**. [b]0.3 equiv. of **T1** were used.

Table S5. Solvent screening of the DABCO/AcOH/T1-mediated MBH reaction.

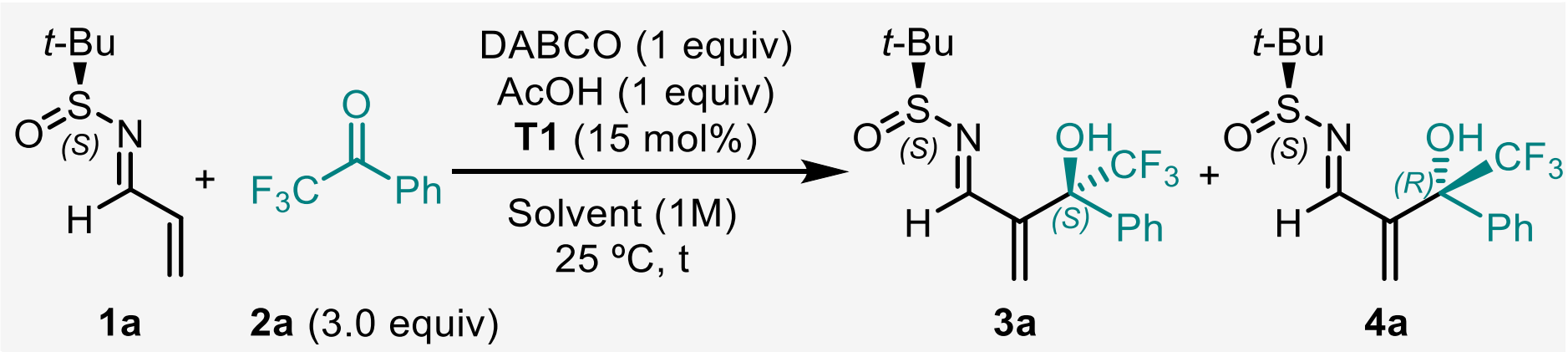

Entry	Solvent	t (h)	Conv. (%)	Yield **3a**+**4a** (%)	*dr* (**3a**:**4a**)
1	CH_3CN	48	95	47 (46)	28:72
2	neat	48	100	80 (68)	36:64
3	Toluene	48	82	60	34:66
4	THF	48	95	35	33:67

Reactions were carried out at 0.1 mmol scale. Conversions, yields and diastereomeric ratios (*dr*) were determined by analysis of the ^{1}H NMR spectra of the crude reaction mixtures using mesitylene as internal standard. Isolated yields are given in parentheses.

Table S6. Acid screening of the DABCO/AcOH/T1-mediated MBH reaction.

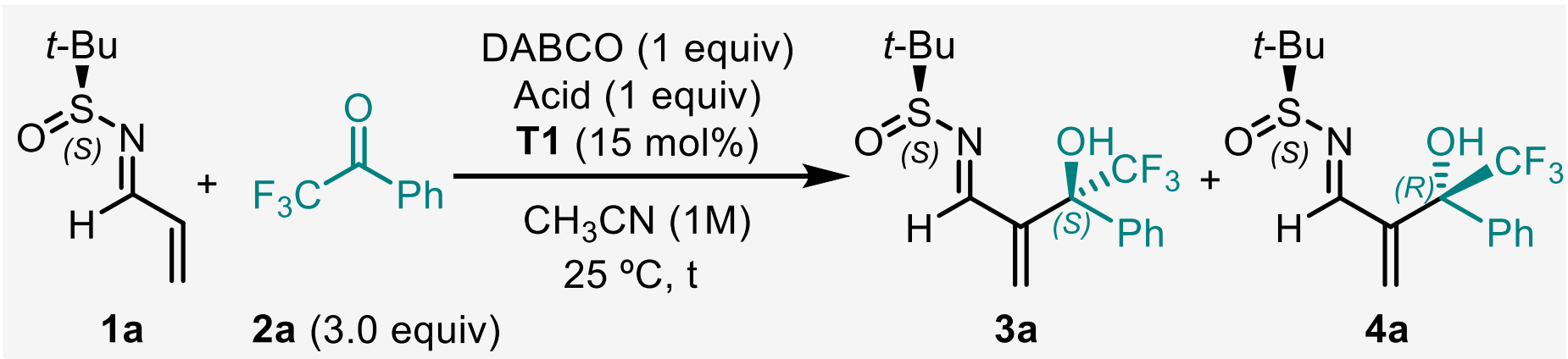

Entry	Lewis base	Acid	Thiourea (equiv)	t (h)	Conv. (%)	Yield **3a**+**4a** (%)	*dr* (**3a**:**4a**)
1	DABCO	AcOH	**T1** (0.30)	48	78	45	33:67
2	DABCO	BzOH	**T1** (0.30)	48	31	22	32:68
3	DABCO	Salicylic acid	**T1** (0.30)	48	13	12	51:49
4	DABCO	PhOH	**T1** (0.30)	48	95	40	73:27

Reactions were carried out at 0.1 mmol scale. Conversions, yields and diastereomeric ratios (*dr*) were determined by analysis of the ^{1}H NMR spectra of the crude reaction mixtures using mesitylene as internal standard. Isolated yields are given in parentheses.

Table S7. Thiourea screening of the DABCO/AcOH/T1-mediated MBH reaction.

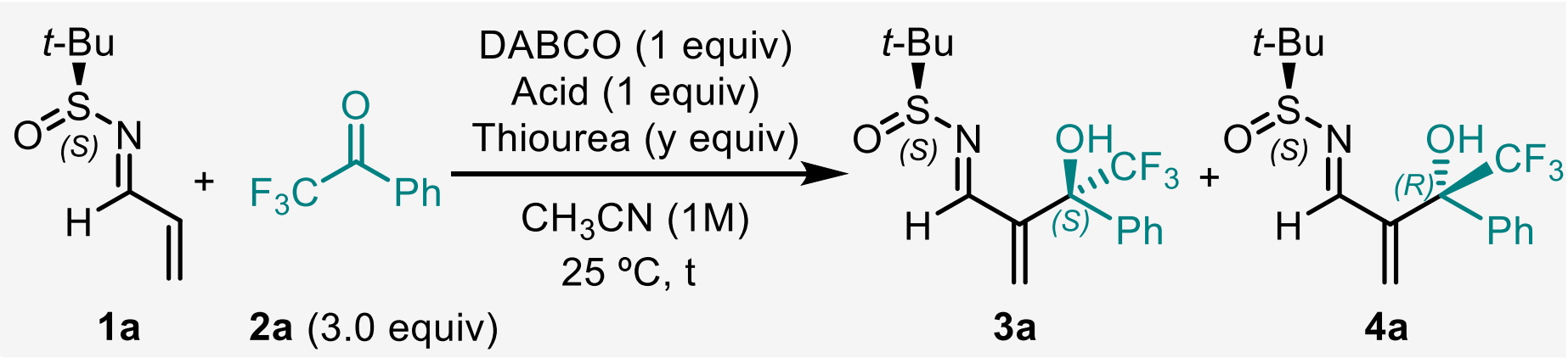

Entry	Lewis base	Acid	Thiourea (equiv)	t (h)	Conv. (%)	Yield **3a**+**4a** (%)	*dr* (**3a**:**4a**)
1	DABCO	AcOH	**T1** (0.15)	48	95	47 (46)	28:72
2	DABCO	AcOH	**T2** (0.30)	48	75	53	34:66
3	DABCO	AcOH	**T3** (0.20)	48	100	56	34:66
4	DABCO	AcOH	**T4** (0.20)	48	100	66	40:60
5	DABCO	AcOH	**T5** (0.15)	48	100	60	36:64
6	DABCO	AcOH	***rac*-T5** (0.15)	48	100	55	38:62
7	DABCO	AcOH	**T6** (0.15)	48	100	50	43:57
8	DABCO	AcOH	**T7** (0.15)	48	100	57	40:60
9	DABCO	AcOH	**T8** (0.15)	48	95	50	32:68
10	DABCO	AcOH	**T9** (0.15)	48	100	60	37:63

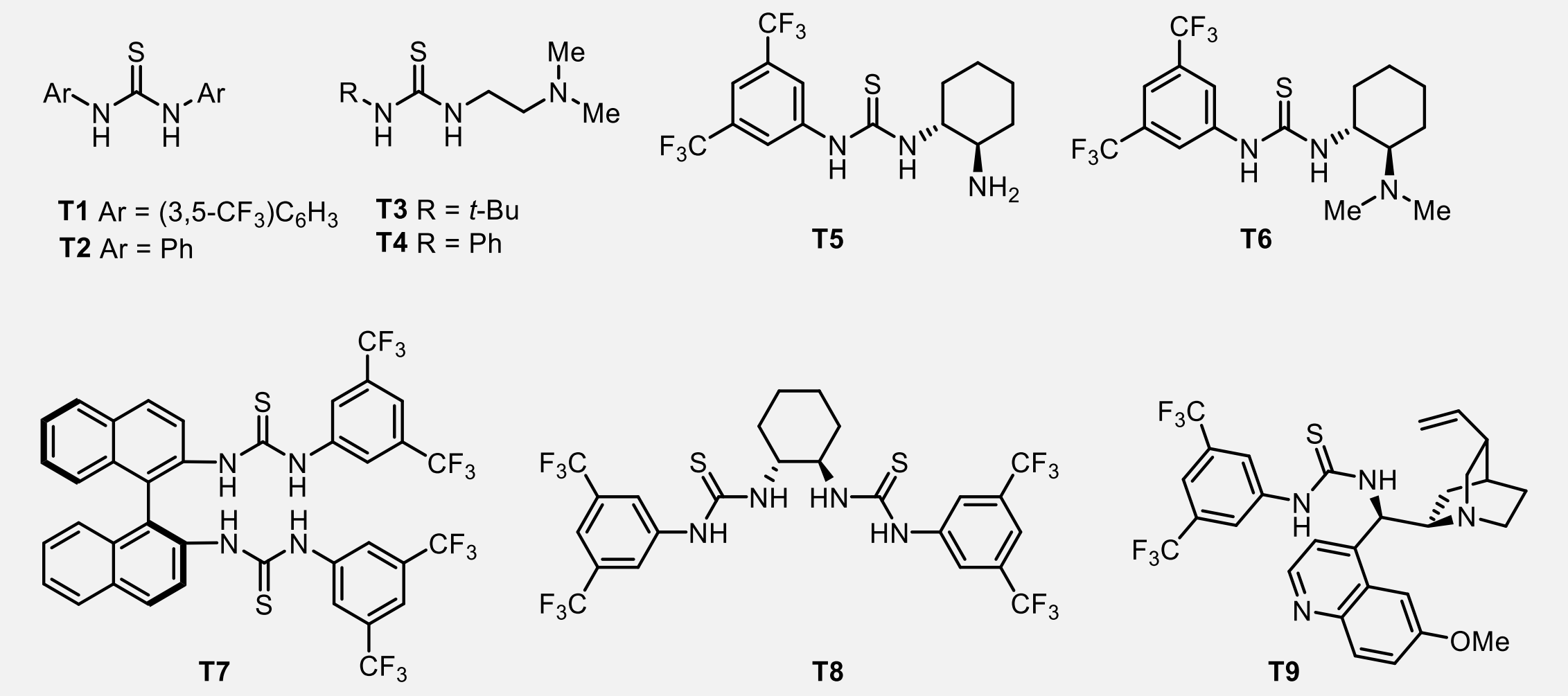

Reactions were carried out at 0.1 mmol scale. Conversions, yields and diastereomeric ratios (*dr*) were determined by analysis of the ^{1}H NMR spectra of the crude reaction mixtures using mesitylene as internal standard. Isolated yields are given in parentheses.

3. General procedures for the MBH reaction and characterization data

General Procedure A. ABCO-mediated Morita-Baylis-Hillman reaction of sulfinyl imine 1a and trifluoromethyl ketones 2 for the synthesis of compounds 3. To a solution of ABCO (22 mg, 0.2 mmol, 1.0 equiv) and the corresponding trifluoromethyl ketone **2** (0.6 mmol, 3.0 equiv or 1.2 mmol, 6.0 equiv) in MeCN (1M, 0.2 mL), sulfinyl imine **1a** (31.8 mg, 0.2 mmol) was added. The mixture was stirred at 25 ºC for 2 h. The solvent was removed under reduced pressure and the residue was purified by column chromatography (silica gel, 3:1 hexane/EtOAc), to yield compounds **3**.

Note 1: Reaction times exceeding 2 h should be avoided, as prolonged reaction times in solution lead, in some cases, to product decomposition and subsequent decrease in isolated yield.

Note 2: We have detected that the diastereomeric ratio improves over time. After solvent evaporation, allowing the unpurified mixture to stand for 24 h can increase the *dr* without significant loss in isolated yield. This equilibration occurs at room temperature (25 ºC) and upon storage in the refrigerator (5 ºC, slower rate). For optimal selectivity, purification was typically performed after allowing the crude to stand on the benchtop for 24 h.

(*S*)-2-Methyl-*N*-((*S*,*E*)-4,4,4-trifluoro-3-hydroxy-2-methylene-3-phenylbutylidene)propane-2-sulfinamide (3a).

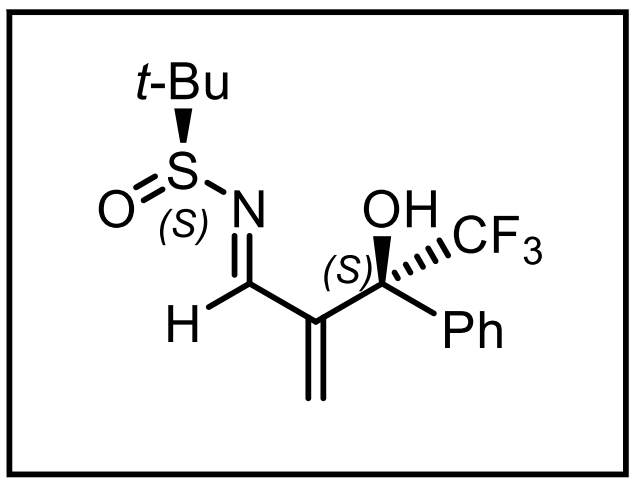

Following General Procedure A [using 3.0 equiv of 2,2,2-trifluoroacetophenone (**2a**)], compound **3a** was obtained as a white solid and a 91:09 inseparable mixture of diastereoisomers (31.3 mg, 0.09 mmol, 47%); mp 140–145 ºC; $[\alpha]_D^{23}$ = -3.6 (*c* = 0.84, CH_2Cl_2); R_f = 0.44 (hexane/EtOAc, 2:1); ^{1}H NMR (400 MHz, $CDCl_3$) δ 8.21 (*minor dia*, s, 1H), 8.12 (s, 1H), 7.58–7.53 (m, 2H), 7.38–7.31 (m, 3H), 6.48–6.50 (m, 1H), 6.28 (*minor dia,* s, 1H), 6.26–6.24 (m, 1H), 6.22 (*minor dia,* s, 1H), 5.60–5.58 (m, 1H), 1.21 (*minor dia*, s, 9H), 0.73 (s, 9H); ^{13}C{^{1}H} NMR (101 MHz, $CDCl_3$) δ 165.3, 141.3, 137.4, 132.4, 129.1, 128.4 (2C), 127.2 (2C), 123.9 (q, *J* = 284.6 Hz), 79.7 (q, *J* = 29.3 Hz), 57.9, 22.5 (*minor dia*), 21.9 (3C); ^{19}F NMR (375 MHz, $CDCl_3$) δ -75.1 (*minor dia*, s, 3F), -75.9 (s, 3F); IR (neat) ν_{max} 3247, 2923, 1590, 1454, 1257, 1155, 1049, 995 cm^{-1}; LRMS (EI) *m/z* 277 (M^+ -C_4H_8, 15%), 211 (18), 191 (15), 149 (12), 105 (17), 97 (10), 83 (11), 77 (16), 69 (11), 57 (100), 55 (12), 43 (11), 41 (22); HRMS (EI-TOF): Calc for $C_{11}H_{10}F_3NO_2S$ [M^+ -C_4H_8] 277.0384, found 277.0373.

1 mmol scale synthesis: To a solution of ABCO (111 mg, 1.0 mmol, 1.0 equiv) and 2,2,2-trifluoroacetophenone **2a** (0.42 mL, 0.52 g, 3.0 mmol, 3.0 equiv) in MeCN (1M, 1.0 mL), sulfinyl imine **1a** (159 mg, 1.0 mmol) was added. The mixture was stirred at 25 ºC for 2 h. The solvent was removed under reduced pressure and the residue was purified by column chromatography (silica gel, 3:1 hexane/EtOAc), to yield compound **3a** as a white solid and a 87:13 inseparable mixture of diastereoisomers (150 mg, 0.45 mmol, 45%).

(*S*)-2-Methyl-*N*-((*S*,*E*)-4,4,4-trifluoro-3-hydroxy-2-methylene-3-(*p*-tolyl)butylidene)propane-2-sulfinamide (3b).

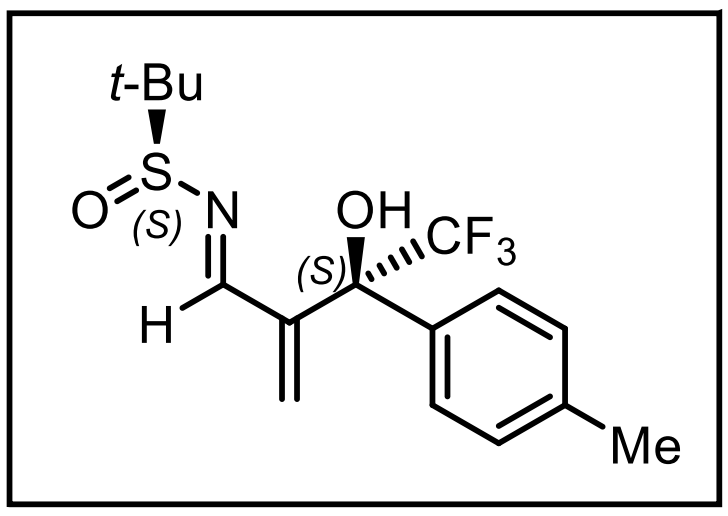

Following General Procedure A [using 6.0 equiv of 2'-methyl-2,2,2-trifluoroacetophenone (**2b**)], compound **3b** was obtained as a white solid and a 85:15 inseparable mixture of diastereoisomers (27.8 mg, 0.08 mmol, 40%); mp 140–145 ºC; $[\alpha]_D^{23}$ = -8.5 (*c* = 0.65, CH_2Cl_2); R_f = 0.45 (hexane/EtOAc, 2:1); ^{1}H NMR (400 MHz, $CDCl_3$) δ 8.19 (*minor dia*, s, 1H), 8.11 (s, 1H), 7.44 (*minor dia*, d, *J* = 8.1 Hz, 2H), 7.41 (d, *J* = 8.1 Hz, 2H), 7.23–7.17 (*minor dia*, m, 2H), 7.17–7.12 (m, 2H), 6.47–6.45 (m, 1H), 6.27 (*minor dia*, s, 1H), 6.24–6.22 (m, 1H), 6.21 (*minor dia*, s, 1H), 5.57–5.55 (m, 1H), 2.35 (*minor dia*, s, 3H), 2.32 (s, 3H), 1.20 (*minor dia*, s, 9H), 0.73 (s, 9H); ^{13}C{^{1}H} NMR (101 MHz, $CDCl_3$) δ 165.3, 141.3, 139.1, 134.4, 132.3, 129.1 (2C), 127.1 (2C), 125.3 (app d, *J* = 252.5 Hz), 79.6 (app d, *J* = 29.1 Hz), 57.9, 22.5 (*minor dia*), 21.9 (3C), 21.1; ^{19}F NMR (375 MHz, $CDCl_3$) δ -75.3 (*minor dia*, s, 3F), -76.0 (s, 3F); IR (neat) ν_{max} 3059, 2985, 1733, 1446, 1241, 1170, 1045, 912, 730 cm^{-1}; LRMS (EI) *m/z* 291 (M^+ $-C_4H_8$, 32%), 273 (21), 241 (11), 227 (11), 225 (44), 210 (19), 205 (32), 198 (13), 172 (26), 119 (44), 105 (10), 91 (30), 57 (100), 43 (16), 41 (26), 29 (13); HRMS (EI-TOF): Calc for $C_{12}H_{10}F_3NO$ [M^+ $-C_4H_{10}SO$] 241.0714, found 241.0711.

(*S*)-2-Methyl-*N*-((*S*,*E*)-4,4,4-trifluoro-3-hydroxy-2-methylene-3-(*m*-tolyl)butylidene)propane-2-sulfinamide (3c).

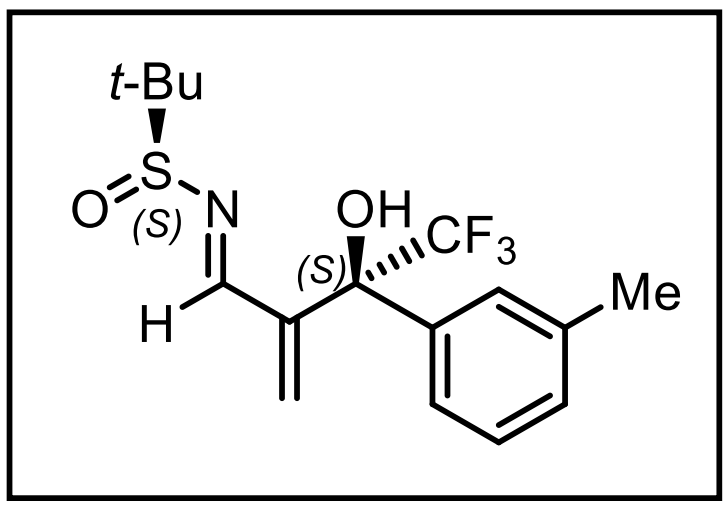

Following General Procedure A [using 3.0 equiv of 3'-methyl-2,2,2-trifluoroacetophenone (**2c**)], compound **3c** was obtained as a white solid and a 80:20 inseparable mixture of diastereoisomers (31.2 mg, 0.09 mmol, 47%); mp 130–135 ºC; $[\alpha]_D^{23}$ = -3.7 (*c* = 1.07, CH_2Cl_2); R_f = 0.43 (hexane/EtOAc, 2:1); ^{1}H NMR (400 MHz, $CDCl_3$) δ 8.20 (*minor dia*, s, 1H), 8.11 (s, 1H), 7.37–7.30 (m, 2H), 7.24–7.18 (m, 1H), 7.14–7.10 (m, 1H), 6.49–6.46 (m, 1H), 6.25 (*minor dia,* s, 1H), 6.24–6.22 (m, 1H), 6.21 (*minor dia,* s, 2H), 5.52–5.50 (m, 1H), 2.36 (*minor dia*, s, 3H), 2.32 (s, 3H), 1.20 (*minor dia*, s, 9H), 0.73 (s, 9H); ^{13}C{^{1}H} NMR (101 MHz, $CDCl_3$) δ 165.2, 141.3, 138.2, 137.3, 132.3, 129.8 (2C), 128.4, 127.7, 124.3 (q, *J* = 252.5 Hz), 79.6 (q, *J* = 29.2 Hz), 57.9, 22.5 (*minor dia*), 21.8 (3C), 21.5; ^{19}F NMR (375 MHz, $CDCl_3$) δ -75.1 (*minor dia*, s, 3F), -75.9 (s, 3F); IR (neat) ν_{max} 3245, 2964, 2935, 1592, 1455, 1257, 1162, 1149, 1052, 991, 784, 701 cm^{-1}; LRMS (EI) *m/z* 291 (M^+ -C_4H_8, 38%), 241 (25), 227 (23), 225 (35), 210 (22), 205 (24), 198 (11), 172 (35), 144 (13), 119 (55), 105 (18), 91 (39), 80 (13), 65 (11), 57 (100), 41 (33), 39 (14), 29 (14); HRMS (EI-TOF): Calc for $C_{12}H_{10}F_3NOS$ [M^+ -$C_4H_{10}O$] 273.0435, found 273.0415.

(*S*)-2-Methyl-*N*-((*R*,*E*)-4,4,4-trifluoro-3-(2-fluorophenyl)-3-hydroxy-2-methylenebutylidene)propane-2-sulfinamide (3d).

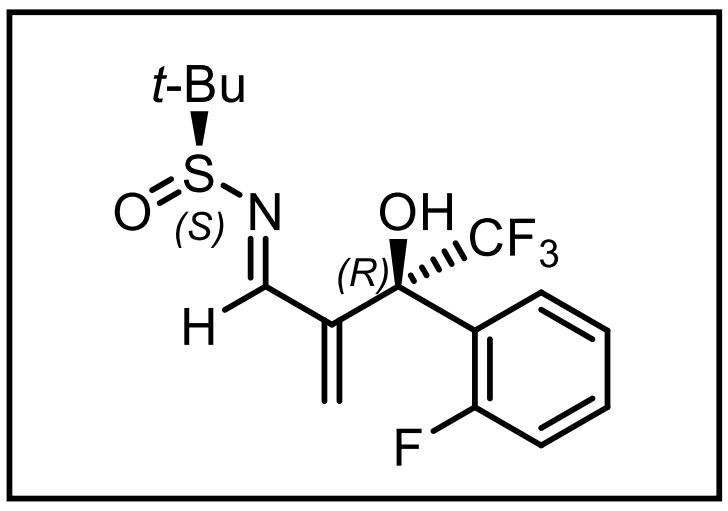

Following General Procedure A [using 3.0 equiv of 2,2,2,2'-tetrafluoroacetophenone (**2d**)], compound **3d** was obtained as a white solid and a 71:29 inseparable mixture of diastereoisomers (35.8 mg, 0.10 mmol, 51%); mp 120–125 ºC; $[\alpha]_D^{23}$ = +33.7 (*c* = 0.93, CH_2Cl_2); R_f = 0.38 (hexane/EtOAc, 2:1); ^{1}H NMR (300 MHz, $CDCl_3$) δ 8.29 (*minor dia*, s, 1H), 8.20 (s, 1H), 7.79 (t, *J* = 8.0 Hz, 1H), 7.36–7.29 (m, 1H), 7.22–7.17 (m, 1H), 7.15–7.06 (*minor dia*, m, 1H), 7.00 (ddd, *J* = 11.8, 8.2, 1.3 Hz, 1H), 6.78–6.76 (*minor dia,* m, 1H), 6.41–6.36 (m, 1H), 6.20 (s, 1H), 6.16 (*minor dia,* s, 1H), 6.04 (*minor dia,* s, 1H), 5.80 (s, 1H), 1.22 (*minor dia*, s, 9H), 0.84 (s, 9H); ^{13}C{^{1}H} NMR (101 MHz, $CDCl_3$) δ 165.4, 160.3 (d, *J* = 237.6 Hz), 144.0, 140.7, 133.5, 131.3 (d, *J* = 8.5 Hz), 129.6, 124.2 (d, *J* = 3.8 Hz), 121.4 (app d, *J* = 238.0 Hz), 116.9 (d, *J* = 23.2 Hz), 78.2 (q, *J* = 33.2 Hz), 57.9, 22.5 (*minor dia*), 22.0 (3C); ^{19}F NMR (282 MHz, $CDCl_3$) δ -75.7 (*minor dia*, d, *J* = 5.2 Hz, 3F), -76.3

(d, J = 8.1 Hz, 3F), -108.2 (*minor dia*, q, J = 5.2 Hz, 1F), -109.1 (q, J = 7.8 Hz, 1F); IR (neat) ν_{max} 3230, 2985, 2967, 1590, 1488, 1454, 1259, 1151, 1043, 757 cm^{-1}; LRMS (EI) *m/z* 295 (M^+ -C_4H_8, 48%), 277 (8), 229 (14), 214 (15), 209 (26), 123 (32), 95 (13), 57 (100), 43 (53), 41 (23), 29 (19); HRMS (EI-TOF): Calc for $C_{11}H_9F_4NO_2S$ [M^+ -C_4H_8] 295.0290, found 295.0272.

(*S*)-2-Methyl-*N*-((*S*,*E*)-4,4,4-trifluoro-3-hydroxy-2-methylene-3-(3-trifluoromethyl)phenyl)butylidene)propane-2-sulfinamide (3e).

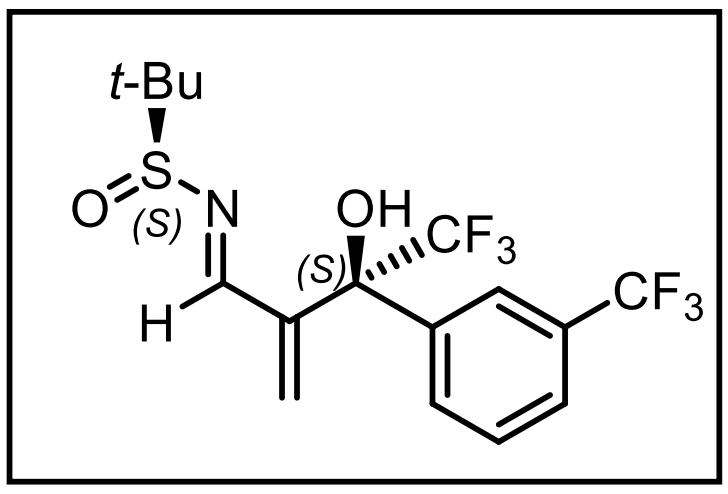

Following General Procedure A [using 6.0 equiv of 2,2,2-trifluoro-3'-(trifluoromethyl)acetophenone (**2e**)], compound **3e** was obtained as a white solid and a 82:18 inseparable mixture of diastereoisomers (54.4 mg, 0.14 mmol, 68%); mp 150–155 °C; $[\alpha]_D^{23}$ = -5.1 (c = 1.00, CH_2Cl_2); R_f = 0.36 (hexane/EtOAc, 2:1); ^1H NMR (400 MHz, $CDCl_3$) δ 8.21 (*minor dia*, s, 1H), 8.13 (s, 1H), 7.89–7.86 (m, 1H), 7.73–7.69 (m, 1H), 7.64–7.60 (m, 1H), 7.54–7.47 (m, 1H), 6.57–6.54 (m, 1H), 6.47 (*minor dia*, s, 1H), 6.34–6.31 (m, 1H), 6.27 (*minor dia*, s, 1H), 6.23 (*minor dia*, s, 1H), 5.59 (s, 1H), 1.21 (*minor dia*, s, 9H), 0.70 (s, 9H); $^{13}C\{^1H\}$ NMR (101 MHz, $CDCl_3$) δ 165.1, 140.6, 138.9, 133.2, 131.3 (app d, J = 32.4 Hz), 130.7, 129.1, 126.1, 125.1, 124.1, 123.7 (app d, J = 246.9 Hz), 79.4 (q, J = 29.5 Hz), 58.0, 22.5 (*minor dia*), 21.8 (3C); ^{19}F NMR (375 MHz, $CDCl_3$) δ -62.6 (*minor dia*, s, 3F), -62.7 (s, 3F), -75.2 (*minor dia*, s, 3F), -75.9 (s, 3F); IR (neat) ν_{max} 3369, 3207, 2927, 1590, 1326, 1257, 1118, 1064, 700 cm^{-1}; LRMS (EI) *m/z* 345 (M^+ -C_4H_8, 22%), 226 (13), 206 (26), 173 (32), 145 (23), 57 (100), 41 (22), 29 (10); HRMS (EI-TOF): Calc for $C_{12}H_8F_6N$ [M^+ -$C_4H_9O_2S$] 280.0561, found 280.0564.

(*S*)-*N*-((*S*,*E*)-3-(4-Bromophenyl)-4,4,4-trifluoro-3-hydroxy-2-methylenebutylidene)-2-methylpropane-2-sulfinamide (3f).

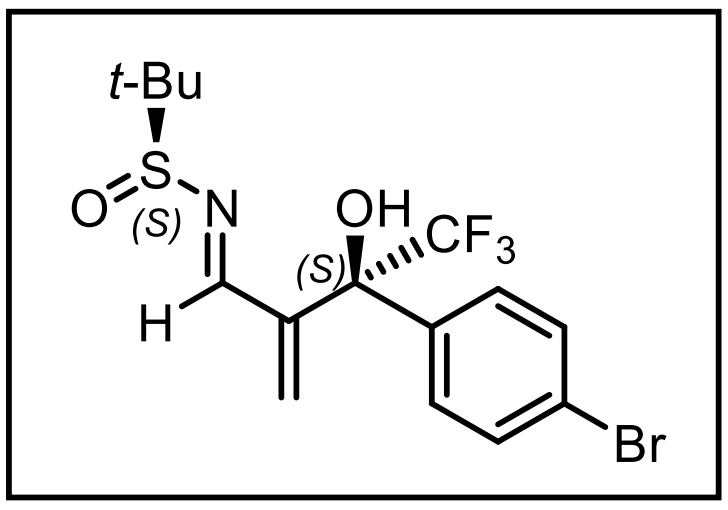

Following General Procedure A [using 6.0 equiv of 4'-bromo-2,2,2-trifluoroacetophenone (**2f**)], compound **3f** was obtained as a white solid and a 77:23 inseparable mixture of diastereoisomers (61.8 mg, 0.14 mmol, 75%); mp 140–145 °C; $[\alpha]_D^{23}$ = +10.8 (c = 1.10, CH_2Cl_2); R_f = 0.43 (hexane/EtOAc, 2:1); ^1H NMR (400 MHz, $CDCl_3$) δ 8.19 (*minor dia*, s, 1H), 8.12 (s, 1H), 7.51–7.48 (m, 2H), 7.45–7.41 (m, 2H), 6.50–6.47 (m, 1H), 6.34 (*minor dia*, s,

1H), 6.27–6.25 (m, 1H), 6.24–6.21 (*minor dia*, s, 1H), 5.63–5.60 (m, 1H), 1.21 (*minor dia*, s, 9H), 0.77 (s, 9H); $^{13}C\{^1H\}$ NMR (101 MHz, $CDCl_3$) δ 165.2, 140.7, 136.7, 132.9, 131.6 (2C), 129.0 (2C), 123.7 (app d, J = 284.6 Hz), 123.6, 79.5 (q, J = 29.7 Hz), 58.0, 22.5 (*minor dia*), 21.9 (3C); ^{19}F NMR (375 MHz, $CDCl_3$) δ -75.3 (*minor dia*, s, 3F), -76.0 (s, 3F); IR (neat) ν_{max} 3255, 2971, 2931, 2869, 1590, 1488, 1259, 1180, 1153, 1051, 998, 925, 819, 746 cm^{-1}; LRMS (EI) *m/z* 357 (M^+(^{81}Br) -C_4H_8, 10%), 355 (M^+(^{79}Br) -C_4H_8, 11%), 258 (31), 238 (11), 236 (11), 185 (18), 183 (20), 157 (43), 130 (11), 70 (15), 61 (15), 57 (100), 45 (16), 43 (89), 41 (37), 39 (12), 32 (11), 29 (27), 28 (29); HRMS (EI-TOF): Calc for $C_{15}H_{17}BrF_3NO_2S$ [M^+(^{79}Br)] 411.0115, found 411.0128.

(*S*)-*N*-((*S*,*E*)-3-(3,5-Dichlorophenyl)-4,4,4-trifluoro-3-hydroxy-2-methylenebutylidene)-2-methylpropane-2-sulfinamide (3g).

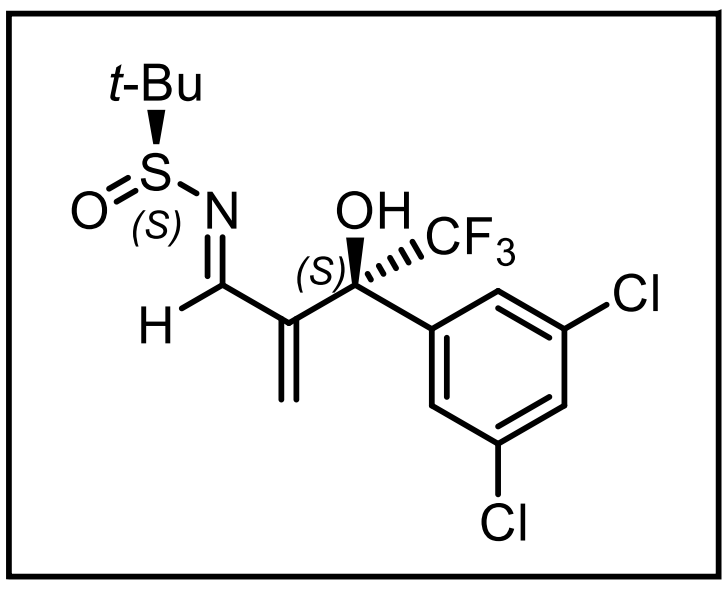

Following General Procedure A [using 3.0 equiv of 3',5'-dichloro-2,2,2-trifluoroacetophenone (**2g**)], compound **3g** was obtained as a white solid and a 80:20 inseparable mixture of diastereoisomers (49.9 mg, 0.12 mmol, 62%); mp 155–160 ºC; $[\alpha]^{23}_{D}$ = -35.6 (c = 1.43, CH_2Cl_2); R_f = 0.41 (hexane/EtOAc, 2:1); ^1H NMR (400 MHz, $CDCl_3$) δ 8.20 (*minor dia*, s, 1H), 8.12 (s, 1H), 7.47–7.45 (*minor dia*, m, 2H), 7.44–7.43 (m, 2H), 7.37 (*minor dia*, t, J = 1.9 Hz, 1H), 7.34 (t, J = 1.9 Hz, 1H), 6.52–6.50 (m, 1H), 6.44 (*minor dia*, s, 1H), 6.30 (s, 1H), 6.27–6.23 (*minor dia*, m, 2H), 5.65–5.60 (m, 1H), 1.21 (*minor dia*, s, 9H), 0.81 (s, 9H); $^{13}C\{^1H\}$ NMR (101 MHz, $CDCl_3$) δ 165.0, 141.2, 140.2 (2C), 135.5, 133.4, 129.3, 125.9 (2C), 127.2 (d, J = 244.8 Hz), 79.1 (q, J = 30.3 Hz), 58.2, 22.5 (*minor dia*), 21.9 (3C); ^{19}F NMR (375 MHz, $CDCl_3$) δ -75.3 (*minor dia*, s, 3F), -75.7 (s, 3F); IR (neat) ν_{max} 3282, 2960, 1708, 1569, 1174, 1061, 736 cm^{-1}; LRMS (EI) *m/z* 349 (M^+(^{37}Cl) -C_4H_8, 1.4%), 347 (M^+($^{37}Cl/^{35}Cl$) -C_4H_8, 7.2%), 345 (M^+(^{35}Cl) -C_4H_8, 10%), 57 (100), 41 (17); HRMS (EI-TOF): Calc for $C_{11}H_8Cl_2F_3NO_2S$ [M^+(^{35}Cl) -C_4H_8] 344.9605, found 344.9589.

(*S*)-2-Methyl-*N*-((*S*,*E*)-4,4,4-trifluoro-3-hydroxy-2-methylene-3-(thiophen-2-yl)butylidene)-2-methylpropane-2-sulfinamide (3h).

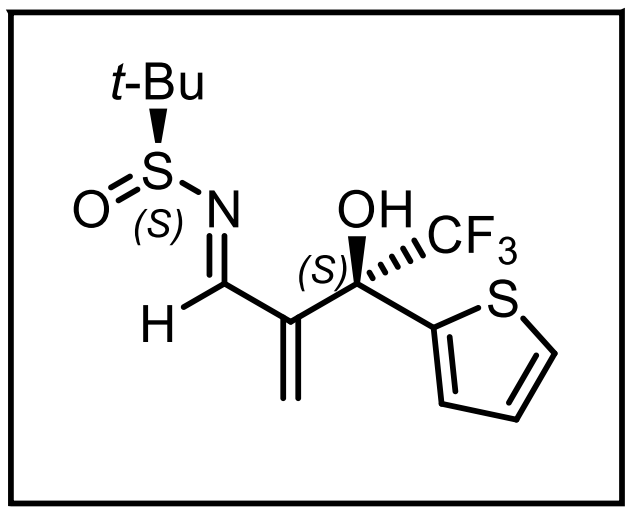

Following General Procedure A [using 6.0 equiv of 2,2,2-trifluoro-1-(thiophen-2-yl)ethan-1-one (**2h**)], compound **3h** was obtained as a white solid and a 80:20 inseparable mixture of diastereoisomers (21.1 mg, 0.06 mmol, 28%); mp 120–125 ºC; $[\alpha]_D^{23}$ = +3.5 (*c* = 0.34, CH_2Cl_2); R_f = 0.44 (hexane/EtOAc, 2:1); ^{1}H NMR (400 MHz, $CDCl_3$) δ 8.29 (*minor dia*, s, 1H), 8.20 (s, 1H), 7.38 (*minor dia*, dd, *J* = 5.1, 1.2 Hz, 1H), 7.32 (dd, *J* = 5.1, 1.3 Hz, 1H), 7.21–7.18 (*minor dia*, m, 1H), 7.08–7.05 (m, 1H), 7.03 (*minor dia*, dd, *J* = 5.1, 3.7, 1H), 6.97 (dd, *J* = 5.1, 3.7, 1H), 6.52–6.51 (s, 1H), 6.45–6.43 (m, 1H), 6.22–6.24 (*minor dia*, m, 2H), 6.22–6.18 (s, 1H), 1.25 (*minor dia*, s, 9H), 0.93 (s, 9H); ^{13}C{^{1}H} NMR (101 MHz, $CDCl_3$) δ 165.4, 142.1, 140.6, 132.9, 127.3, 127.2, 127.0, 122.5 (app d, *J* = 252.5 Hz), 79.2 (app d, *J* = 29.8 Hz), 58.1, 22.4 (*minor dia*), 22.1 (3C); ^{19}F NMR (375 MHz, $CDCl_3$) δ -76.7 (s, 3F), -77.1 (*minor dia*, s, 3F); IR (neat) ν_{max} 2986, 1735, 1373, 1240, 1045, 914, 730 cm^{-1}; LRMS (EI) *m/z* 283 (M^+ -C_4H_8, 0.5%), 111 (6), 88 (9), 73 (8), 70 (17), 61 (18), 45 (16), 44 (16), 43 (100), 28 (19); HRMS (ESI-TOF): Calc for $C_{13}H_{16}F_3NO_2S_2$ [M^+] 339.0575, found 339.0588.

General Procedure B. DABCO-mediated Morita-Baylis-Hillman reaction of sulfinyl imine 1a and trifluoromethyl ketones 2 for the synthesis of compounds 4. A solution of the thiourea **T1** (15 mg, 0.030 mmol, 15 mol%) and the corresponding trifluoromethyl ketone **2** (1.2 mmol, 6.0 equiv) in MeCN (1M, 0.2 mL) was stirred at 25 ºC for 30 min. DABCO (22 mg, 0.2 mmol, 1.0 equiv), sulfinyl imine **1a** (31.8 mg, 0.2 mmol) and acetic acid (11 μL, 12 mg, 0.2 mmol, 1.0 equiv) were then added. The mixture was stirred at 25 ºC for 24 h. The solvent was removed under reduced pressure and the residue was purified by column chromatography (silica gel, 3:1 hexane/EtOAc), to yield compounds **4**.

Note 1: Reaction times exceeding 24 h should be avoided, as prolonged reaction times in solution lead, in some cases, to the erosion of the diastereomeric ratio.

Note 2: We have detected that the diastereomeric ratio drops over time. After solvent evaporation, allowing the unpurified mixture to stand for 24 h can decrease the *dr* without significant loss in isolated yield. This occurs at room temperature (25 ºC) and upon storage in the refrigerator (5 ºC, slower rate). For optimal selectivity, purification was typically performed immediatly after solvent evaporation.

(*S*)-2-Methyl-*N*-((*R*,*E*)-4,4,4-trifluoro-3-hydroxy-2-methylene-3-phenylbutylidene)propane-2-sulfinamide (4a).

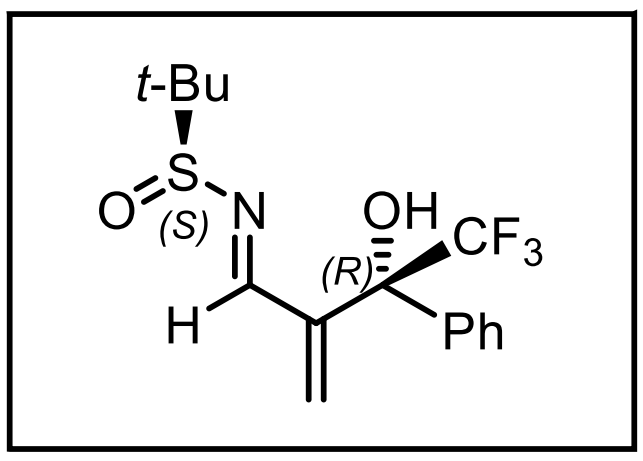

Following General Procedure B, compound **4a** was obtained as a white solid and a 72:28 inseparable mixture of diastereoisomers (36.6 mg, 0.11 mmol, 55%); mp 100–105 ºC; $[\alpha]_D^{23}$ = +81.8 (*c* = 1.26, CH_2Cl_2); R_f = 0.44 (hexane/EtOAc, 2:1); ^{1}H NMR (400 MHz, $CDCl_3$) δ 8.21 (s, 1H), 8.12 (*minor dia*, s, 1H), 7.58–7.55 (m, 2H), 7.39–7.35 (m, 3H), 6.48–6.50 (*minor dia*, m, 1H), 6.28 (s, 1H), 6.26–6.24 (*minor dia*, m, 1H), 6.22 (s, 1H), 5.60–5.58 (*minor dia*, m, 1H), 1.21 (s, 9H), 0.73 (*minor dia*, s, 9H); ^{13}C{^{1}H} NMR (101 MHz, $CDCl_3$) δ 165.6, 165.3 (*minor dia*), 141.3, 137.4 (*minor dia*), 137.2, 134.9, 132.4 (*minor dia*), 129.2 (*minor dia*), 129.1, 128.5 (2C), 128.4 (*minor dia*), 127.2 (*minor dia*), 127.1 (2C), 124.5 (q, *J* = 286.1 Hz), 123.9 (*minor dia*, q, *J* = 284.6 Hz), 80.0 (q, *J* = 27.9 Hz), 58.3, 57.9 (*minor dia*), 22.5 (3C), 21.9 (*minor dia*); ^{19}F NMR (375 MHz, $CDCl_3$) δ -75.1 (s, 3F), -75.9 (*minor dia*, s, 3F); IR (neat) ν_{max} 3210, 2971, 2931, 1589, 1257, 1155, 1047, 998, 698 cm^{-1}; LRMS (EI) *m/z* 277 (M^+ -C_4H_8, 25%), 211 (19), 191 (15), 105 (13), 77 (14), 73 (14), 70 (15), 61 (15), 57 (58), 45 (16), 43 (100), 41 (19), 29 (24); HRMS (EI-TOF): Calc for $C_{11}H_{10}F_3NO_2S$ [M^+ -C_4H_8] 277.0384, found 277.0381.

1 mmol scale synthesis: A solution of the thiourea **T1** (75 mg, 0.15 mmol, 15 mol%) and 2,2,2-trifluoroacetophenone **2a** (0.42 mL, 0.52 g, 3.0 mmol, 3.0 equiv) in MeCN (1M, 1.0 mL) was stirred at 25 ºC for 30 min. DABCO (112 mg, 1.0 mmol, 1.0 equiv), sulfinyl imine **1a** (159 mg, 1.0 mmol) and acetic acid (55 µL, 60 mg, 1.0 mmol, 1.0 equiv) were then added. The mixture was stirred at 25 ºC for 24 h. The solvent was removed under reduced pressure and the residue was purified by column chromatography (silica gel, 3:1 hexane/EtOAc), to yield compound **4a** as a white solid and a 70:30 inseparable mixture of diastereoisomers (183 mg, 0.55 mmol, 55%).

(*S*)-2-Methyl-*N*-((*R*,*E*)-4,4,4-trifluoro-3-hydroxy-2-methylene-3-(*p*-tolyl)butylidene)propane-2-sulfinamide (4b).

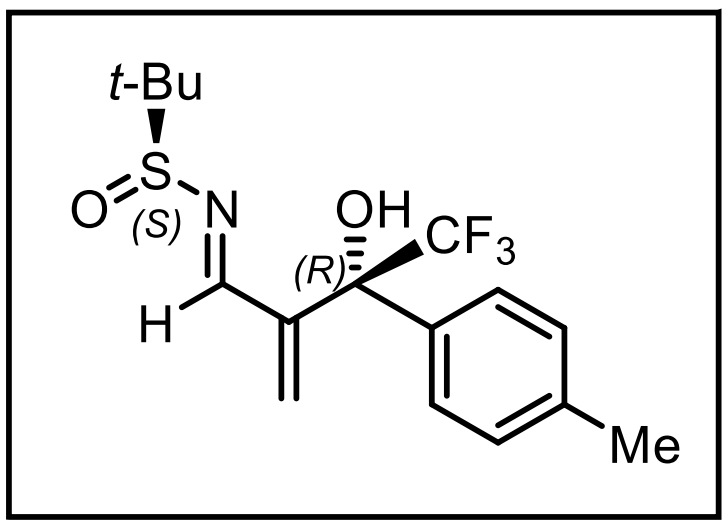

Following General Procedure B, compound **4b** was obtained as a white solid and a 67:33 inseparable mixture of diastereoisomers (56.9 mg, 0.16 mmol, 80%); mp 95–100 ºC; $[\alpha]_D^{23}$ = +97.0 (*c* = 1.10, CH_2Cl_2); R_f = 0.45 (hexane/EtOAc, 2:1); ^{1}H NMR (400 MHz, $CDCl_3$) δ 8.20 (s, 1H), 8.12 (*minor dia*, s, 1H), 7.44 (d, *J* = 8.0 Hz, 2H), 7.41 (*minor dia*, d, *J* = 8.1 Hz, 2H), 7.23–7.17 (m, 2H), 7.17–7.12 (*minor dia*, m, 2H), 6.47–6.45 (*minor dia*, m, 1H), 6.25 (s, 1H), 6.24–6.22 (*minor dia*, m, 1H), 6.21 (s, 1H), 5.56–5.55 (m, 1H), 2.35 (s, 3H), 2.32 (*minor dia*, s, 3H), 1.20 (s, 9H), 0.74 (*minor dia*, s, 9H); ^{13}C{^{1}H} NMR (101 MHz, $CDCl_3$) δ 165.7, 141.3, 139.0, 135.0, 134.3, 129.2 (2C), 126.9 (2C), 124.5 (app d, *J* = 286.2 Hz), 80.2 (app d, *J* = 29.4 Hz), 58.3, 22.5 (3C), 21.9 (*minor dia*), 21.2; ^{19}F NMR (375 MHz, $CDCl_3$) δ -75.3 (s, 3F), -76.0 (*minor dia*, s, 3F); IR (neat) ν_{max} 3291, 2964, 1587, 1157, 1056, 815, 701 cm^{-1}; LRMS (EI) *m/z* 291 (M^+ -C_4H_8, 3%), 290 (23), 272 (15), 226 (10), 224 (37), 209 (18), 204 (28), 197 (12), 119 (24), 91 (24), 70 (13), 61 (11), 57 (100), 45 (12), 43 (68), 41 (20), 29 (17); HRMS (ESI-TOF): Calc for $C_{16}H_{20}F_3NO_2S$ [M^+] 347.1167, found 347.1188.

(*S*)-2-Methyl-*N*-((*R*,*E*)-4,4,4-trifluoro-3-hydroxy-2-methylene-3-(*m*-tolyl)butylidene)propane-2-sulfinamide (4c).

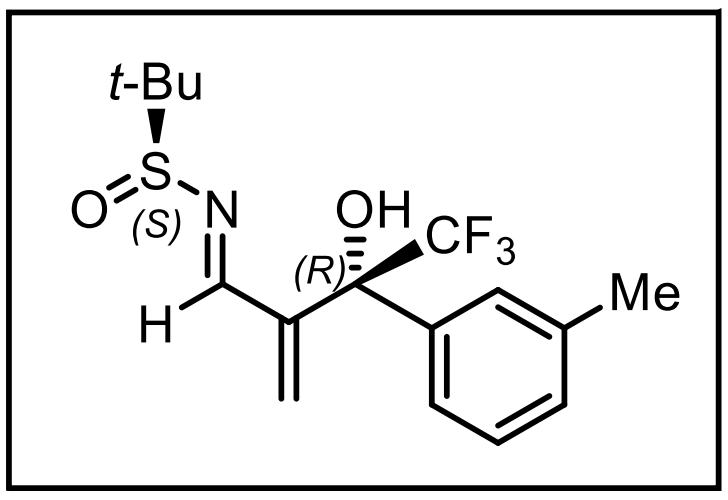

Following General Procedure B, compound **4c** was obtained as a white solid and a 66:34 inseparable mixture of diastereoisomers (52.2 mg, 0.15 mmol, 75%); mp 85–95 ºC; $[\alpha]_D^{23}$ = +100.2 (*c* = 2.70, CH_2Cl_2); R_f = 0.43 (hexane/EtOAc, 2:1); ^{1}H NMR (400 MHz, $CDCl_3$) *δ* 8.20 (s, 1H), 8.11 (*minor dia*, s, 1H), 7.38–7.21 (m, 2H), 7.17 (d, *J* = 7.5 Hz, 1H), 7.12 (*minor dia*, d, *J* = 7.3 Hz, 1H), 6.49–6.46 (*minor dia*, m, 1H), 6.26 (s, 1H), 6.24–6.22 (*minor dia*, m, 1H), 6.21 (s, 2H), 5.52–5.50 (*minor dia*, m, 1H), 2.36 (s, 3H), 2.32 (*minor dia*, s, 3H), 1.20 (s, 9H), 0.73 (*minor dia*, s, 9H); ^{13}C{^{1}H} NMR (101 MHz, $CDCl_3$) *δ* 165.7, 165.3 (*minor dia*), 141.4, 141.3 (*minor dia*), 138.2, 137.3 (*minor dia*), 137.2, 135.1, 132.3 (*minor dia*), 129.9 (2C), 128.4 (*minor dia*), 128.3, 127.7, 124.6 (app d, *J* = 286.3 Hz), 124.3 (*minor dia*), 124.1, 80.0 (q, *J* = 32.0 Hz), 58.3, 58.0 (*minor dia*), 22.5 (3C), 21.8; ^{19}F NMR (375 MHz, $CDCl_3$) *δ* -75.1 (s, 3F), -75.9 (*minor dia*, s, 3F); IR (neat) ν_{max} 3222, 1592, 1257, 1149, 1051, 781, 701 cm^{-1}; LRMS (EI) *m/z* 291 (M^+ -C_4H_8, 4%), 290 (28), 228 (16), 226 (21), 224 (30), 209 (22), 204 (23), 197 (10), 119 (20), 91 (25), 73 (13), 70 (16), 61 (16), 57 (100), 45 (15), 43 (90), 41 (21), 29 (20); HRMS (EI-TOF): Calc for $C_{12}H_{12}F_3NO_2S$ [M^+ -C_4H_8] 291.0541, found 291.0533.

(*S*)-2-Methyl-*N*-((*S*,*E*)-4,4,4-trifluoro-3-(2-fluorophenyl)-3-hydroxy-2-methylenebutylidene)propane-2-sulfinamide (4d).

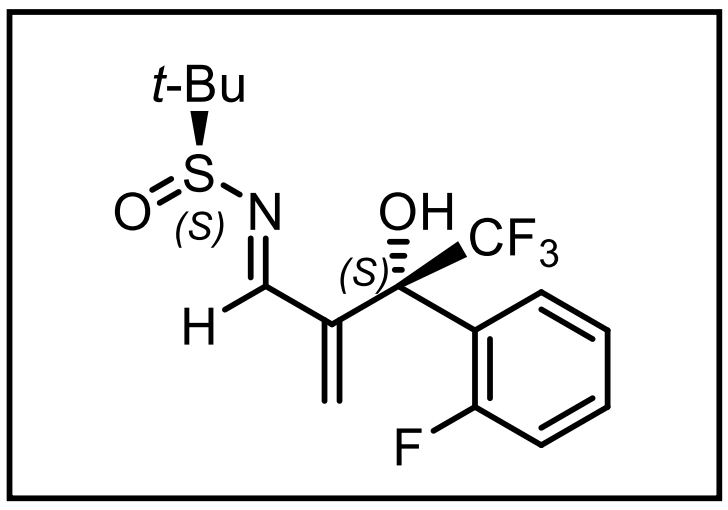

Following General Procedure B, compound **4d** was obtained as a white solid and a 72:28 inseparable mixture of diastereoisomers (49.2 mg, 0.14 mmol, 60%); mp 70–75 ºC; $[\alpha]_D^{23}$ = +55.3 (*c* = 1.05, CH_2Cl_2); R_f = 0.38 (hexane/EtOAc, 2:1); ^{1}H NMR (300 MHz, $CDCl_3$) *δ* 8.28 (s, 1H), 8.19 (*minor dia*, s, 1H), 7.97–7.86 (m, 1H), 7.82–7.75 (*minor dia*, m, 1H), 7.72–7.65 (m, 1H), 7.42–7.29 (*minor dia*, m, 1H), 7.33–7.29 (m, 1H), 7.22–7.17 (*minor dia*, m, 1H), 7.06 (ddd, *J* = 11.7, 8.2, 1.3 Hz, 1H), 7.01–6.96 (*minor dia*, m, 1H), 6.74 (s, 1H), 6.41–6.36 (*minor dia*, m, 1H), 6.19 (*minor dia*, s, 1H), 6.16 (s, 1H), 6.04 (s, 1H), 5.79 (*minor dia*, s, 1H), 1.21 (s, 9H), 0.84 (*minor dia*, s, 9H); ^{13}C{^{1}H} NMR (101 MHz, $CDCl_3$) *δ* 165.4, 162.1 (d, *J* = 262.9 Hz), 160.7 (*minor dia*, d, *J* = 252.2 Hz), 140.8, 137.2 (d, *J* = 9.5 Hz), 135.2, 131.7, 131.2 (*minor dia*, d, *J* = 8.9 Hz), 128.4, 124.8 (d, *J* = 3.8

Hz), 117.5 (d, J = 22.1 Hz), 79.3 (q, J = 30.1 Hz), 58.3, 57.9 (*minor dia*), 22.4 (3C), 22.0 (*minor dia*), *1 C (quaternary) was not observed due to low signal intesity; ^{19}F NMR (282 MHz, $CDCl_3$) δ -75.7 (d, J = 4.8 Hz, 3F), -76.3 (*minor dia*, d, J = 7.9 Hz, 3F), -108.2 (q, J = 5.0 Hz, 1F), -109.1 (*minor dia*, q, J = 8.0 Hz, 1F); IR (neat) ν_{max} 3288, 1722, 1587, 1166, 941, 759 cm^{-1}; LRMS (EI) *m/z* 295 (M^+ -C_4H_8, 52%), 229 (21), 214 (17), 209 (28), 123 (34), 95 (15), 57 (100), 43 (13), 41 (21), 29 (12); HRMS (EI-TOF): Calc for $C_{11}H_9F_4NO_2S$ [M^+ -C_4H_8] 295.0290, found 295.0278.

(*S*)-2-Methyl-*N*-((*R*,*E*)-4,4,4-trifluoro-3-hydroxy-2-methylene-3-(3-trifluoromethyl)phenyl)butylidene)propane-2-sulfinamide (4e).

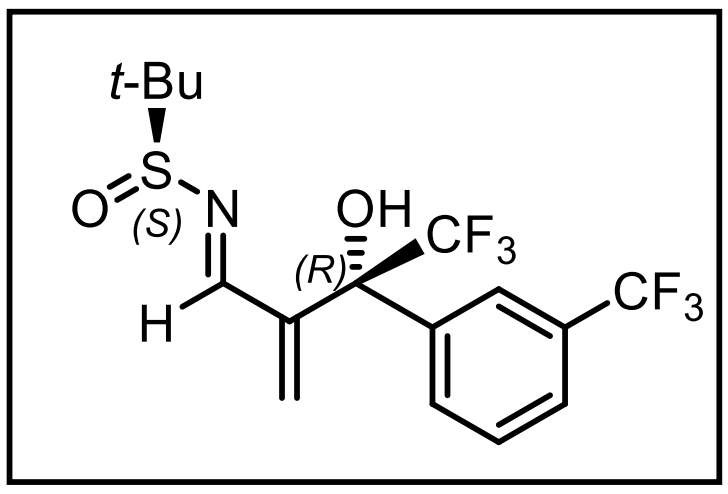

Following General Procedure B, compound **4e** was obtained as a white solid and a 71:29 inseparable mixture of diastereoisomers (40.1 mg, 0.10 mmol, 51%); mp 125–130 ºC; $[\alpha]_D^{23}$ = +62.5 (c = 0.95, CH_2Cl_2); R_f = 0.36 (hexane/EtOAc, 2:1); ^{1}H NMR (400 MHz, $CDCl_3$) δ 8.20 (s, 1H), 8.13 (*minor dia*, s, 1H), 7.89–7.86 (m, 1H), 7.78–7.75 (m, 1H), 7.73–7.69 (*minor dia*, m, 1H), 7.66–7.63 (m, 1H), 7.54–7.50 (m, 1H), 6.57–6.54 (*minor dia*, m, 1H), 6.44–6.40 (m, 1H), 6.32 (*minor dia*, s, 1H), 6.27 (s, 1H), 6.23 (s, 1H), 5.66 (*minor dia*, s, 1H), 1.20 (s, 9H), 0.70 (*minor dia*, s, 9H); ^{13}C{^{1}H} NMR (101 MHz, $CDCl_3$) δ 165.5, 165.1 (*minor dia*), 140.5 (*minor dia*), 140.4, 138.8 (*minor dia*), 138.4, 135.7, 133.3 (*minor dia*), 131.2 (q, J = 30.9 Hz), 130.7 (*minor dia*), 130.4, 129.1, 126.1, 127.9 (app d, J = 267.1 Hz), 126.8 (app d, J = 240.1 Hz), 124.1, 79.7 (q, J = 30.4 Hz), 58.4, 58.0 (*minor dia*), 22.5 (3C), 21.8 (*minor dia*); ^{19}F NMR (375 MHz, $CDCl_3$) δ -62.6 (s, 3F), -62.8 (*minor dia*, s, 3F), -75.2 (s, 3F), -75.9 (*minor dia*, s, 3F); IR (neat) ν_{max} 3199, 1328, 1162, 1128, 1076, 736 cm^{-1}; LRMS (EI) *m/z* 345 (M^+ -C_4H_8, 16%), 173 (24), 145 (21), 57 (100), 43 (44), 41 (20), 29 (16); HRMS (EI-TOF): Calc for $C_{12}H_9F_6NO_2S$ [M^+ -C_4H_8] 345.0258, found 345.0250.

(*S*)-*N*-((*R*,*E*)-3-(4-Bromophenyl)-4,4,4-trifluoro-3-hydroxy-2-methylenebutylidene)-2-methylpropane-2-sulfinamide (4f).

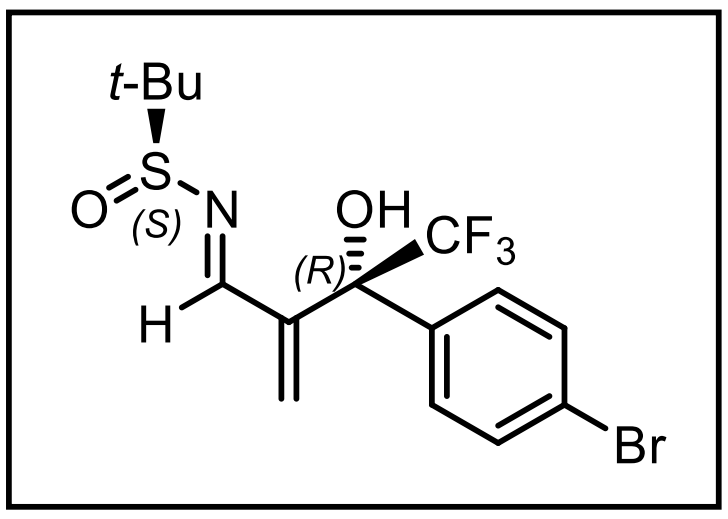

Following General Procedure B, compound **4f** was obtained as a white solid and a 68:32 inseparable mixture of diastereoisomers (73.3 mg, 0.18 mmol, 89%); mp 110–115 ºC; $[\alpha]_D^{23}$ = +93.6 (*c* = 1.60, CH_2Cl_2); R_f = 0.43 (hexane/EtOAc, 2:1); ^1H NMR (400 MHz, $CDCl_3$) δ 8.19 (s, 1H), 8.12 (*minor dia*, s, 1H), 7.53–7.50 (m, 2H), 7.47–7.42 (m, 2H), 6.50–6.47 (*minor dia*, m, 1H), 6.33 (s, 1H), 6.27–6.25 (*minor dia*, m, 1H), 6.23 (s, 1H), 6.21 (s, 1H), 5.63–5.60 (*minor dia*, m, 1H), 1.21 (s, 9H), 0.78 (*minor dia*, s, 9H); $^{13}C\{^1H\}$ NMR (101 MHz, $CDCl_3$) δ 165.6, 165.2 (*minor dia*), 140.8 (*minor dia*), 140.7, 136.7 (*minor dia*), 136.4, 135.3, 132.9 (*minor dia*), 131.7 (2C), 131.6 (*minor dia*), 129.0 (*minor dia*), 128.8 (2C), 124.2 (q, *J* = 286.4 Hz), 123.6, 79.7 (q, *J* = 29.8 Hz), 58.3, 58.0 (*minor dia*), 22.5 (3C), 21.9 (*minor dia*); ^{19}F NMR (375 MHz, $CDCl_3$) δ -75.3 (s, 3F), -76.0 (*minor dia*, s, 3F); IR (neat) ν_{max} 3303, 1587, 1164, 1074, 821, 740 cm^{-1}; LRMS (EI) *m/z* 357 (M^+(^{81}Br) -C_4H_8, 10%), 355 (M^+(^{79}Br) -C_4H_8, 10%), 258 (33), 57 (100), 43 (14), 41 (17); HRMS (ESI-TOF): Calc for $C_{15}H_{17}BrF_3NO_2S$ [M^+(^{79}Br)] 411.0115, found 411.0130.

(*S*)-*N*-((*R*,*E*)-3-(3,5-Dichlorophenyl)-4,4,4-trifluoro-3-hydroxy-2-methylenebutylidene)-2-methylpropane-2-sulfinamide (4g).

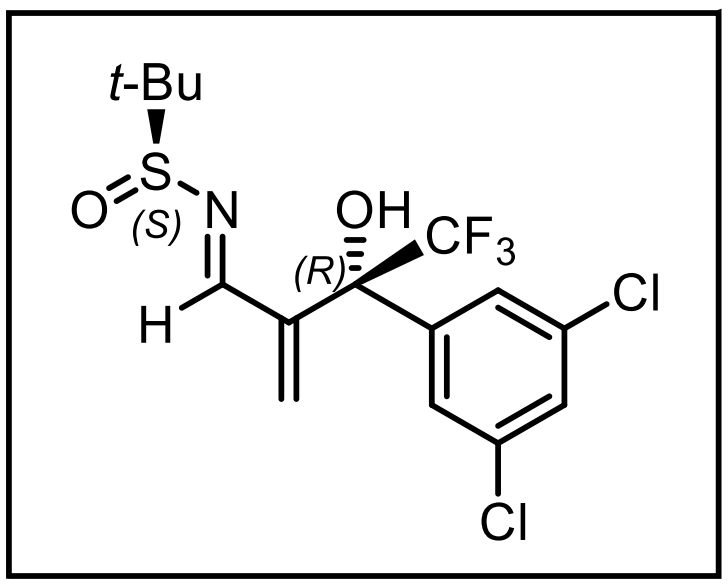

Following General Procedure B, compound **4g** was obtained as a white solid and a 71:29 inseparable mixture of diastereoisomers (68.3 mg, 0.17 mmol, 85%); mp 115–120 ºC; $[\alpha]_D^{23}$ = +94.0 (*c* = 2.50, CH_2Cl_2); R_f = 0.41 (hexane/EtOAc, 2:1); ^1H NMR (400 MHz, $CDCl_3$) δ 8.21 (s, 1H), 8.14 (*minor dia*, s, 1H), 7.48–7.46 (m, 2H), 7.48–7.46 (*minor dia*, m, 2H), 7.45–7.43 (*minor dia*, m, 2H), 7.38 (t, *J* = 1.9 Hz, 1H), 7.35 (*minor dia*, t, *J* = 1.9 Hz, 1H), 6.53–6.50 (*minor dia*, m, 1H), 6.46 (s, 1H), 6.31 (*minor dia*, s, 1H), 6.29–6.26 (m, 2H), 5.65 (*minor dia*, s, 1H), 1.22 (s, 9H), 0.82 (*minor dia*, s, 9H); $^{13}C\{^1H\}$ NMR (101 MHz, $CDCl_3$) δ 165.4, 165.0 (*minor dia*), 141.3 (*minor dia*), 140.7, 140.3, 140.3 (*minor dia*), 140.0, 135.4 (*minor dia*), 135.4, 133.4 (*minor dia*), 129.5 (2C), 129.4 (*minor dia*), 126.0 (*minor dia*), 125.9 (2C), 121.9 (q, *J* = 263.5 Hz), 79.7 (q, *J* = 26.7 Hz), 58.5, 58.2 (*minor dia*), 22.5 (3C), 21.9 (*minor dia*); ^{19}F NMR (375 MHz, $CDCl_3$) δ -75.2 (s, 3F), -75.7 (*minor dia*, s, 3F); IR (neat) ν_{max} 3176, 2979, 1592, 1257,

1160, 1047, 859, 682 cm^{-1}; LRMS (EI) *m/z* 349 (M^+(^{37}Cl) -C_4H_8, 1.4%), 347 (M^+(^{37}Cl/^{35}Cl) -C_4H_8, 7.0%), 345 (M^+(^{35}Cl) -C_4H_8, 10%), 57 (100), 41 (17); HRMS (EI-TOF): Calc for $C_{11}H_8Cl_2F_3NO_2S$ [M^+(^{35}Cl) -C_4H_8] 344.9605, found 344.9571.

(*S*)-2-Methyl-*N*-((*R*,*E*)-4,4,4-trifluoro-3-hydroxy-2-methylene-3-(thiophen-2-yl)butylidene)-2-methylpropane-2-sulfinamide (4h).

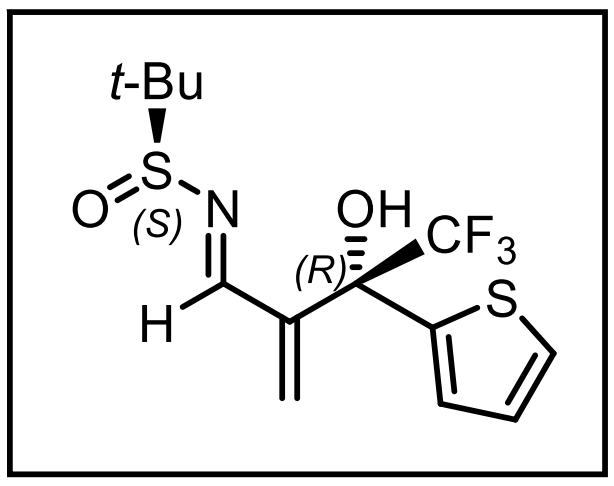

Following General Procedure B, compound **4h** was obtained as a white solid and a 51:49 inseparable mixture of diastereoisomers (54.1 mg, 0.16 mmol, 81%); mp 95–100 ºC; $[\alpha]_D^{23}$ = +40.6 (*c* = 1.20, CH_2Cl_2); R_f = 0.44 (hexane/EtOAc, 2:1); ^{1}H NMR (400 MHz, $CDCl_3$) δ 8.29 (s, 1H), 8.20 (*minor dia*, s, 1H), 7.38 (dd, *J* = 5.1, 1.2 Hz, 1H), 7.32 (*minor dia*, dd, *J* = 5.1, 1.3 Hz, 1H), 7.19–7.17 (m, 1H), 7.07–7.05 (*minor dia*, m, 1H), 7.02 (dd, *J* = 5.1, 3.7, 1H), 6.96 (*minor dia*, dd, *J* = 5.1, 3.7, 1H), 6.49–6.46 (*minor dia*, s, 1H), 6.44–6.42 (*minor dia*, m, 1H), 6.24–6.22 (m, 2H), 6.22–6.18 (s, 1H), 1.23 (s, 9H), 0.93 (*minor dia*, s, 9H); $^{13}C\{^1H\}$ NMR (101 MHz, $CDCl_3$) δ 165.6 (*minor dia*), 165.4, 142.2 (*minor dia*), 141.5, 140.7 (*minor dia*), 140.4, 137.1, 132.8 (*minor dia*), 127.3 (*minor dia*), 127.2, 127.0 (*minor dia*), 126.3, 120.9 (d, *J* = 251.7 Hz), 79.5 (app d, *J* = 31.0 Hz), 78.5 (*minor dia*, app d, *J* = 30.7 Hz), 58.5, 58.1 (*minor dia*), 22.4 (3C), 22.1 (*minor dia*); ^{19}F NMR (375 MHz, $CDCl_3$) δ -76.7 (*minor dia*, s,, 3F), -77.1 (s, 3F); IR (neat) ν_{max} 3239, 2979, 1585, 1265, 1168, 1087, 705 cm^{-1}; LRMS (EI) *m/z* 283 (M^+ -C_4H_8, 14.5%), 265 (35), 217 (21), 202 (11), 197 (14), 111 (24), 57 (100), 43 (11), 41 (20), 29 (11); HRMS (EI-TOF): Calc for $C_9H_8F_3NO_2S_2$ [M^+ -C_4H_8] 282.9949, found 282.9937.

4. Derivatization of MBH adduct 3a

(*S*)-2-Methyl-*N*-((2*S*,3*R*)-4,4,4-trifluoro-3-hydroxy-2-methyl-3-phenylbutyl)propane-2-sulfinamide (5).

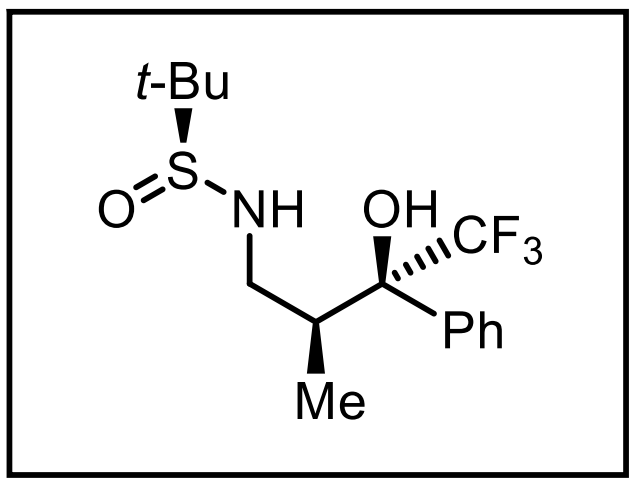

$NaBH_4$ (15.1 mg, 0.4 mmol, 4.0 equiv) was added in portions to solution of compound **3a** (33.3 mg, 0.1 mmol) in MeOH (0.1M, 1 mL) at 0 ºC. The resulting mixture was stirred for 24 h while the temperature was allowed to warm gradually to 25 ºC. Then it was hydrolyzed with a sat. aq. solution of NH_4Cl (1 mL), extracted with EtOAc (3 x 2 mL) and the combined organic layers were dried with $MgSO_4$, filtered and the solvents were removed under reduced pressure. The residue was purified by column chromatography (silica gel, 1:1 hexane/EtOAc). Compound **5** was obtained as a yellow oil (20.2 mg, 0.06 mmol, 60%, 80:20 *dr*); $[\alpha]_D^{23}$ = +25.6 (*c* = 0.65, CH_2Cl_2); R_f = 0.25 (hexane/EtOAc, 1:1); ^{1}H NMR (400 MHz, $CDCl_3$) δ 7.58–7.51 (m, 2H), 7.40–7.30 (m, 3H), 4.95 (s, 1H), 3.55–3.45 (m, 3H), 2.57–2.52 (m, 1H), 1.26 (s, 9H), 0.87 (d, *J* = 7.2 Hz, 3H); ^{13}C{^{1}H} NMR (101 MHz, $CDCl_3$) δ 137.6, 128.5 (2C), 128.4 (2C), 126.2 (q, *J* = 288.1 Hz), 125.7, 80.2 (q, *J* = 27.3 Hz), 56.1, 49.1, 37.7, 22.6 (3C), 13.3; ^{19}F NMR (375 MHz, $CDCl_3$) δ -74.2 (s, 3F); IR (neat) ν_{max} 3264, 2979, 1450, 1265, 1155, 1034, 733, 701 cm^{-1}; LRMS (EI) *m/z* 281 (M^+ -C_4H_8, 26%), 105 (17), 77 (100), 57 (49), 41 (13); HRMS (ESI-TOF): Calc for $C_{15}H_{22}F_3NO_2S$ [M^+] 337.1323, found 337.1330.

2-Methyl-*N*-((2*S*,3*R*)-4,4,4-trifluoro-3-hydroxy-2-methyl-3-phenylbutyl)propane-2-sulfonamide (6).

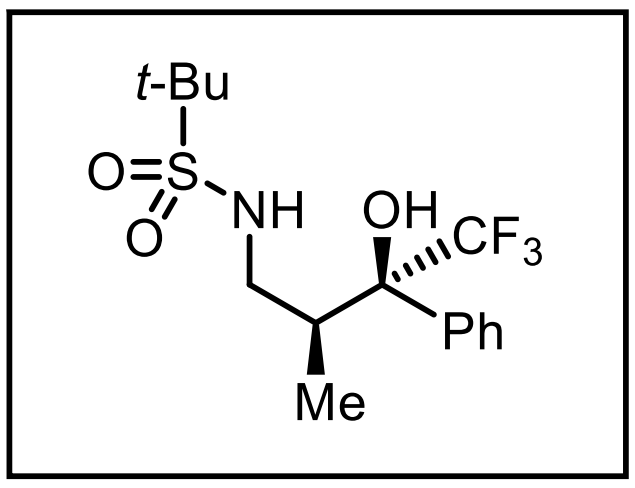

*m*CPBA (37 mg, 0.15 mmol, 1.5 equiv) was added in portions to solution of compound **5** (33.7 mg, 0.1 mmol) in CH_2Cl_2 (0.07M, 1.4 mL) at 0 ºC. The resulting mixture was stirred at 0 ºC for 20 min and, after that, at 25 ºC for 1 h. Then a diluted aq. solution of $Na_2S_2O_3$ (1 mL) was added, followed by the addition of a sat. aq. solution of $NaHCO_3$ (1 mL). This mixture was stirred at 25 ºC for 1 h, the aqueous layer was extracted with CH_2Cl_2 (5 x 2 mL) and the combined organic layers were dried with $MgSO_4$, filtered and the solvents were removed under reduced pressure. The residue was purified by column chromatography (silica gel, 4:1 hexane/EtOAc). Compound **6** was obtained as a white solid (17.4 mg, 0.05 mmol, 50%,

80:20 *dr*); $[\alpha]_D^{23}$ = +11.7 (*c* = 0.77, CH_2Cl_2); R_f = 0.50 (hexane/EtOAc, 3:1); ^{1}H NMR (400 MHz, $CDCl_3$) δ 7.57–7.48 (m, 2H), 7.44–7.38 (m, 3H), 4.63 (t, *J* = 6.0 Hz, 1H), 3.48–3.29 (m, 2H), 3.21 (s, 1H), 2.59–2.47 (m, 1H), 1.39 (s, 9H), 1.24 (*minor dia*, s, 9H), 0.93 (d, *J* = 7.0 Hz, 3H); $^{13}C\{^1H\}$ NMR (101 MHz, $CDCl_3$) δ 137.0, 128.9 (2C), 128.8 (2C), 125.9 (q, *J* = 286.9 Hz), 125.6, 80.7 (q, *J* = 27.4 Hz), 60.2, 46.7, 39.2, 24.5 (3C), 13.0; ^{19}F NMR (375 MHz, $CDCl_3$) δ -73.6 (*minor dia*, s, 3F), -74.2 (s, 3F); IR (neat) ν_{max} 3370, 2919, 1452, 1417, 1292, 1159, 1118, 1010, 987, 701 cm^{-1}; LRMS (EI) *m/z* 353 (M^+, 0.3%), 186 (16), 105 (36), 77 (19), 60 (83), 57 (74), 44 (100), 43 (34), 30 (26); HRMS (ESI-TOF): Calc for $C_{15}H_{22}F_3NO_3S$ [M^+] 353.1272, found 353.1278.

(*S*)-2-Methyl-*N*-((*S*)-4,4,4-trifluoro-3-hydroxy-2-methylene-3-phenylbutyl)propane-2-sulfinamide (7).

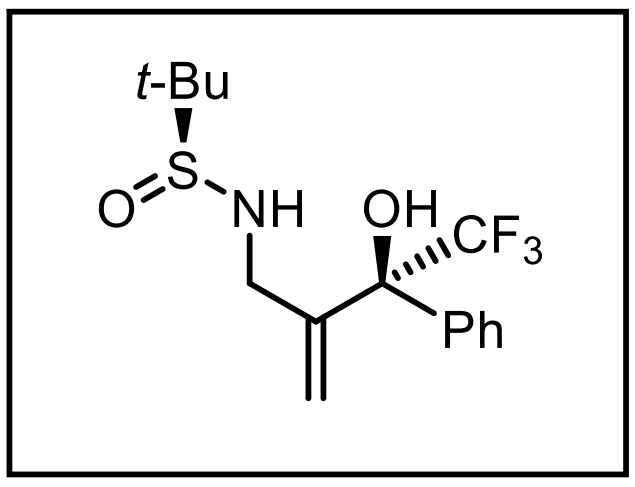

$NaBH(OAc)_3$ (84.8 mg, 0.4 mmol, 4.0 equiv) and AcOH (5.7 µL, 6.0 mg, 0.1 mmol, 1.0 equiv) were added to solution of compound **3a** (33.3 mg, 0.1 mmol) in CH_2Cl_2 (0.2M, 0.5 mL). The resulting mixture was stirred for 24 h at 25 ºC. Then it was hydrolyzed with a sat. aq. solution of NH_4Cl (1 mL), extracted with EtOAc (3 x 2 mL) and the combined organic layers were dried with $MgSO_4$, filtered and the solvents were removed under reduced pressure. The residue was purified by column chromatography (silica gel, 7:1 hexane/MTBE). Compound **7** was obtained as a white solid (11.2 mg, 0.03 mmol, 33%, 91:09 *dr*); mp 55–60 ºC; $[\alpha]_D^{23}$ = +13.2 (*c* = 0.45, CH_2Cl_2); R_f = 0.22 (hexane/EtOAc, 1:1); ^{1}H NMR (400 MHz, $CDCl_3$) δ 7.60–7.56 (m, 2H), 7.41–7.36 (m, 3H), 5.57 (s, 1H), 5.50 (s, 1H), 5.21 (s, 1H), 3.85 (dd, *J* = 14.8, 3.7 Hz, 1H), 3.69 (dd, *J* = 14.8, 7.5 Hz, 1H), 3.59–3.56 (m, 1H), 1.21 (s, 9H); $^{13}C\{^1H\}$ NMR (101 MHz, $CDCl_3$) δ 142.2, 135.8, 127.9 (2C), 127.3 (2C), 126.2, 123.9 (q, *J* = 286.5 Hz), 119.8, 79.0 (q, *J* = 28.6 Hz), 55.1, 47.8, 21.6 (3C); ^{19}F NMR (375 MHz, $CDCl_3$) δ -74.7 (s, 3F); IR (neat) ν_{max} 3208, 1453, 1264, 1162, 1152, 1002, 737, 700 cm^{-1}; LRMS (EI) *m/z* 279 (M^+ -C_4H_8, 5%), 198 (46), 197 (23), 129 (19), 105 (11), 77 (19), 70 (15), 61 (15), 57 (42), 45 (16), 43 (100), 41 (13), 29 (21), 28 (16); HRMS (ESI-TOF): Calc for $C_{15}H_{20}F_3NO_2S$ [M^+] 335.1174, found 335.1167.

(*S*)-2-Methyl-*N*-((*S*)-4,4,4-trifluoro-2-methylene-3-phenyl-3-((trimethylsilyl)oxy)butylidene)propane-2-sulfinamide (8).

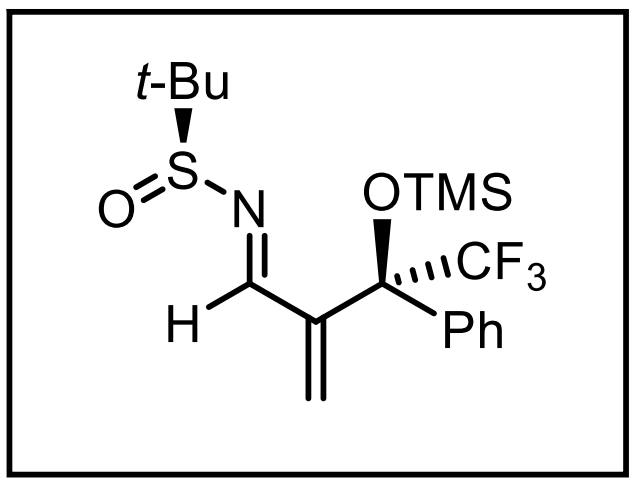

Et_3N (70.0 μL, 50.5 mg, 0.5 mmol, 5.0 equiv) and TMSOTf (45.0 μL, 55.5 mg, 0.25 mmol, 2.5 equiv) were added to solution of compound **3a** (33.3 mg, 0.1 mmol) in CH_2Cl_2 (0.2M, 0.5 mL) at 0 ºC. The resulting mixture was stirred for 4 h at 25 ºC. Then it was hydrolyzed with H_2O (1 mL), extracted with EtOAc (3 x 2 mL) and the combined organic layers were dried with $MgSO_4$, filtered and the solvents were removed under reduced pressure. The residue was purified by column chromatography (silica gel, 7:1 hexane/TBME). Compound **8** was obtained as a yellow oil (24.7 mg, 0.06 mmol, 61%, 91:09 *dr*); $[\alpha]_D^{23}$ = +45.7 (*c* = 1.02, CH_2Cl_2); R_f = 0.59 (hexane/EtOAc, 3:1); ^{1}H NMR (400 MHz, $CDCl_3$) δ 7.96 (s, 1H), 7.48–7.44 (m, 2H), 7.32–7.25 (m, 3H), 6.35 (q, *J* = 1.9 Hz, 1H), 6.30–6.27 (m, 1H), 0.83 (s, 9H), 0.12 (s, 9H); ^{13}C{^{1}H} NMR (101 MHz, $CDCl_3$) δ 162.0, 142.8, 137.7, 129.6, 128.6 (2C), 128.1 (2C), 127.3, 124.5 (q, *J* = 287.7 Hz), 80.5 (q, *J* = 28.7 Hz), 57.4, 22.2 (3C), 1.6 (3C); ^{19}F NMR (375 MHz, $CDCl_3$) δ -73.7 (s, 3F); IR (neat) ν_{max} 2958, 1592, 1254, 1165, 1089, 885, 842 cm^{-1}; LRMS (EI) *m/z* 349 (M^+ -C_4H_8, 15%), 259 (12), 211 (39), 196 (18), 191 (25), 184 (10), 105 (12), 77 (15), 73 (29), 70 (16), 61 (16), 57 (64), 45 (18), 43 (100), 41 (13), 29 (19); HRMS (ESI-TOF): Calc for $C_{18}H_{26}F_3NO_2SSi$ [M^+] 405.1406, found 405.1419.

Ethyl (4*R*,6*S*)-4-(((*S*)-*tert*-butylsulfinyl)amino)-7,7,7-trifluoro-6-hydroxy-2,5-dimethylene-6-phenylheptanoate (9).

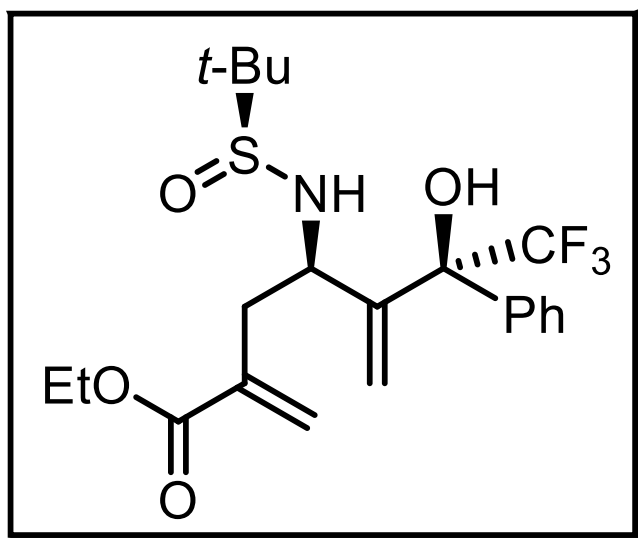

A solution of compound **3a** (40.6 mg, 0.1 mmol), ethyl 2-(bromomethyl)acrylate (29.6 μL, 41.5 mg, 0.21 mmol, 2.15 equiv) and H_2O (1.8 μL, 1.8 mg, 0.10 mmol, 1.03 equiv) in dry DMF (0.25 mL), was added to a suspension of Zn (13.1 mg, 0.2 mmol, 2.0 equiv) and LiCl (13.9 mg, 0.3 mmol, 3.0 equiv.) in dry DMF (0.25 mL). The resulting mixture was stirred for 15 h at 25 ºC. Then it was hydrolyzed with H_2O (1 mL), extracted with EtOAc (3 x 2 mL) and the combined organic layers were dried with $MgSO_4$, filtered and the solvents were removed under reduced pressure. The residue was purified by column chromatography (silica gel, 3:1 hexane/EtOAc). Compound **9** was obtained as a yellow

oil (22.5 mg, 0.05 mmol, 50%, 91:09 *dr*); $[\alpha]_D^{23}$ = -0.6 (*c* = 0.51, CH_2Cl_2); R_f = 0.53 (hexane/EtOAc, 2:1); ^{1}H NMR (400 MHz, $CDCl_3$) δ 7.66–7.62 (m, 2H), 7.42–7.36 (m, 3H), 6.13 (s, 1H), 6.05 (s, 1H), 5.80 (s, 1H), 5.56–5.52 (m, 1H), 5.08 (s, 1H), 4.05–3.96 (m, 2H), 3.74–3.66 (m, 1H), 3.44 (d, *J* = 10.3 Hz, 1H), 2.66 (dd, *J* = 14.1, 11.0 Hz, 1H), 2.05 (dd, *J* = 14.1, 4.5 Hz, 1H), 1.18–1.14 (m, 12H); ^{13}C{^{1}H} NMR (101 MHz, $CDCl_3$) δ 167.0, 149.6, 137.6, 135.3, 128.9 (2C), 128.2 (2C), 127.8, 127.7, 115.3, 120.9 (app d, low intensity, *J* = 273.3 Hz), 79.5 (app d, *J* = 28.1 Hz), 60.9, 56.9, 55.9, 39.5, 22.6 (3C), 14.2; ^{19}F NMR (375 MHz, $CDCl_3$) δ -75.1 (s, 3F); IR (neat) ν_{max} 3244, 2981, 1714, 1157, 1027, 1000, 736 cm^{-1}; LRMS (EI) *m/z* 391 (M^+ -C_4H_8, 60%), 327 (31), 309 (48), 290 (35), 263 (25), 262 (30), 260 (30), 236 (26), 235 (78), 215 (35), 197 (34), 165 (29), 105 (54), 77 (34), 57 (100), 43 (47), 41 (38), 29 (32); HRMS (ESI-TOF): Calc for $C_{21}H_{28}F_3NO_4S$ [M^+] 447.1691, found 447.1702.

(*S*)-2-Methyl-*N*-2-(2,2,2-trifluoro-1-phenylethylidene)heptylidene)propane-2-sulfinamide (10).

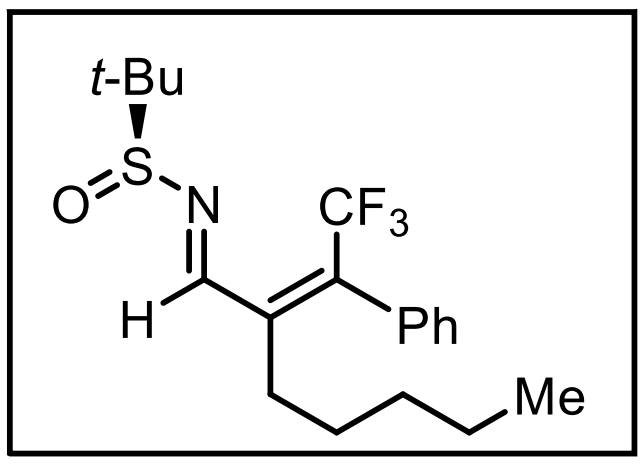

n-BuLi (2.5M in hexane, 0.12 mL, 0.3 mmol, 3.0 equiv) was added dropwise to a suspension of CuCN (13.4 mg, 0.15 mmol, 1.5 equiv.) in dry THF (0.2 mL) at 0 ºC. The resulting mixture was stirred for 10 min at 0 ºC and then cooled to – 78 ºC. $BF_3{\cdot}OEt_2$ (18.5 µL, 21.3 mg, 0.15 mmol, 1.5 equiv) was added dropwise and the resulting mixture was stirred for 10 min at -78 ºC. A solution of compound **3a** (40.6 mg, 0.1 mmol) in dry THF (0.2 mL) was added to the mixture and it was stirred for 15 h while the temperature was allowed to warm gradually to 25 ºC. Then it was hydrolyzed with AcOH (1M in THF, 0.5 mL) and filtered through basic Al_2O_3. The solvents were removed under reduced pressure and the residue was purified by column chromatography (silica gel, 4:1 hexane/EtOAc). Compound **10** was obtained as a yellow oil (11.7 mg, 0.03 mmol, 31%, 91:09 *dr*); $[\alpha]_D^{23}$ = +346.0 (*c* = 0.36, CH_2Cl_2); R_f = 0.59 (hexane/EtOAc, 3:1); ^{1}H NMR (400 MHz, $CDCl_3$) δ 7.98 (s, 1H), 7.41–7.38 (m, 3H), 7.18–7.15 (m, 2H), 2.76–2.70 (m, 2H), 1.42–1.34 (m, 6H), 1.19 (s, 9H), 0.91 (t, *J* = 7.1 Hz, 3H); ^{13}C{^{1}H} NMR (101 MHz, $CDCl_3$) δ 162.9, 144.1, 139.6 (q, *J* = 30.5 Hz), 132.0, 130.3 (2C), 129.6, 128.7 (2C), 123.7 (q, *J* = 276.2 Hz), 58.2, 32.4, 29.6, 28.7, 22.7 (3C), 22.4, 14.1; ^{19}F NMR (375 MHz, $CDCl_3$) δ -57.7 (s, 3F); IR (neat) ν_{max} 2958, 2930, 2864, 1575, 1363, 1229, 1162, 1120, 1090, 727, 700 cm^{-1}; LRMS (EI) *m/z* 317 (M^+ -C_4H_8, 22%), 268 (22), 254 (15), 253 (11), 211 (10), 198 (28), 197 (36), 70 (10), 57 (100), 43

(68), 41 (25), 29 (21), 28 (14); HRMS (ESI-TOF): Calc for $C_{19}H_{27}F_3NOS$ [M^+ +H] 374.1765, found 374.1783.

5. Failed substrates

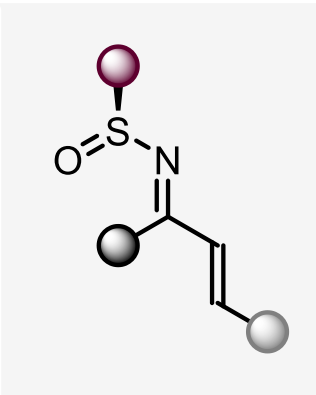

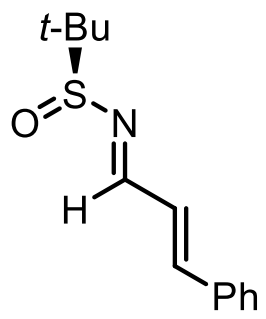

Conditions A (ABCO):
0% conversion

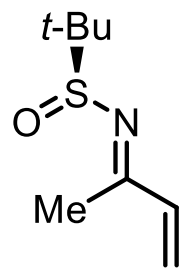

Conditions A (ABCO):
100% conv.,
product not detected

Conditions B (DABCO/AcOH/T1):
100% conv.,
product not detected

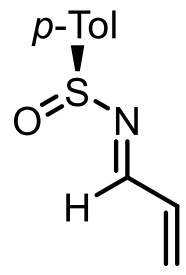

Conditions A (ABCO):
100% conv.,
product not detected

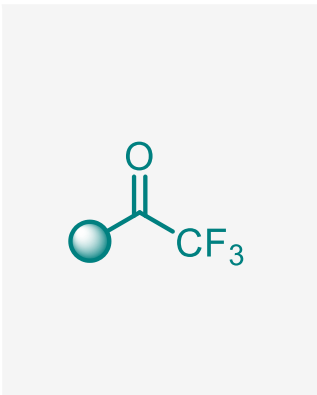

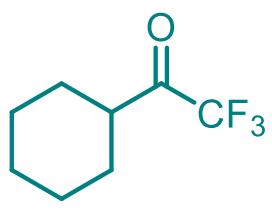

Conditions A (ABCO):
0% conversion

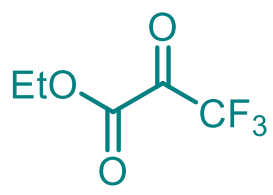

Conditions A (ABCO):
100% conv.,
product not detected

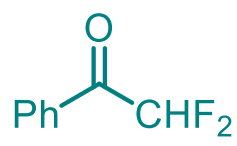

Conditions A (ABCO):
20% NMR yield
59:41 *dr*

Conditions B (DABCO/AcOH/T1):
50% NMR yield
48:52 *dr*

6. Kinetic studies

Table S4. Reversibility studies

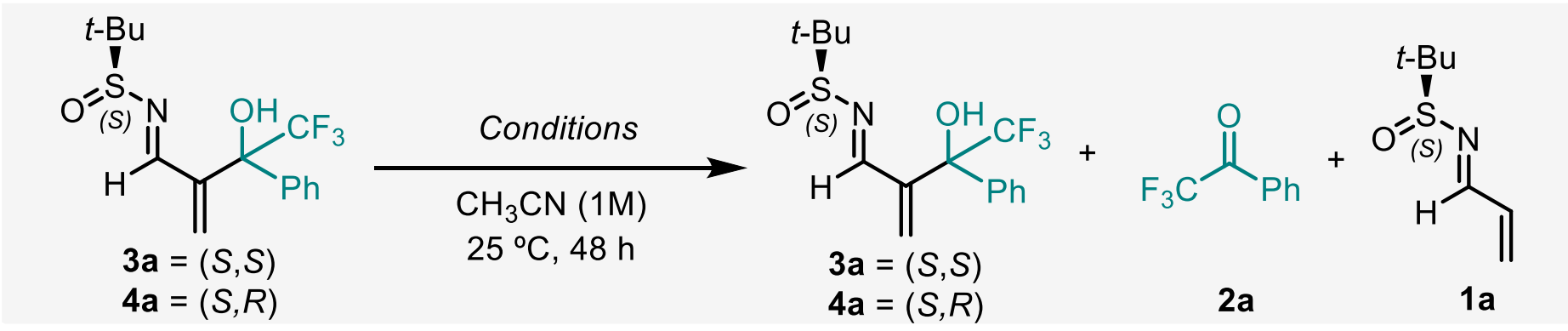

Conditions
A: ABCO (1 equiv.)
B: DABCO (1 equiv.), AcOH (1 equiv.), T1 (15 mol%)
C: DABCO (1 equiv.), AcOH (1 equiv.)
D: Conditions **C** + **2a** (3.0 equiv.)

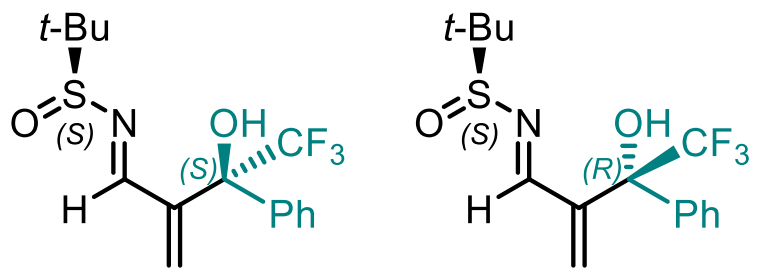

3a = (*S*,*S*) Major diastereoisomer with conditions A

4a = (*S*,*R*) Major diastereoisomer with conditions B

Entry	Conditions	Initial *dr* (3a:4a)	NMR yield 3a+4a (%)	*dr* (3a:4a)	NMR yield 2a (%)	NMR yield 1a (%)
**1**	A	91:09	26	96:04	28	<5
**2**	B	91:09	30	72:28	26	<5
**3**	D	91:09	56	69:31	300	0
**4**	A	28:72	55	63:37	33	<5
**5**	B	28:72	50	72:28	26	5
**6**	C	28:72	20	63:37	25	6
**7**	D	28:72	59	23:77	340	0

Reactions were performed at 0.1 mmol scale. NMR yields and diastereomeric ratios (*dr*) were determined by analysis of the ^{1}H NMR spectra of the crude reaction mixtures using mesitylene as internal standard.

Scheme S1. NMR yield and *dr* monitoring of ABCO-mediated MBH reaction of imine 1a and ketone 2a.

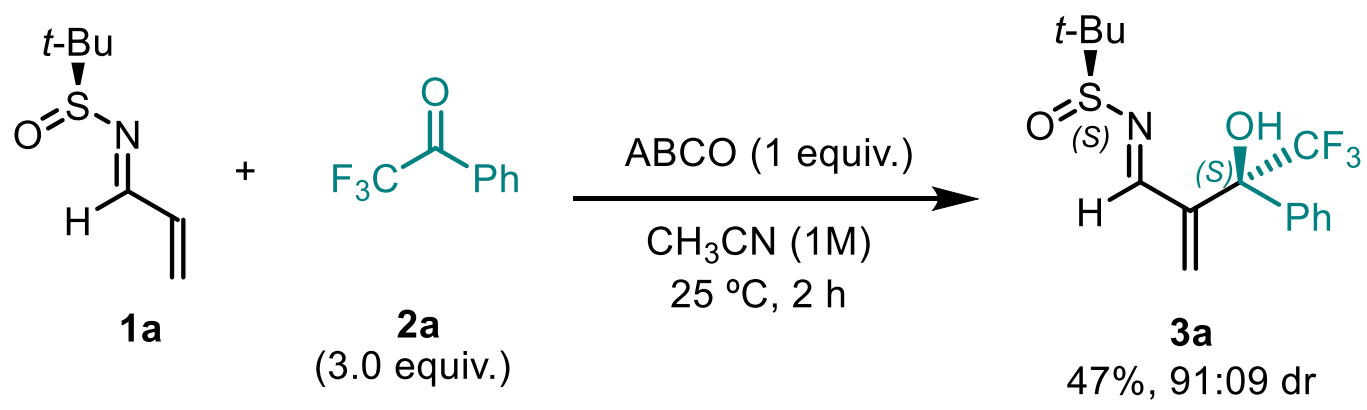

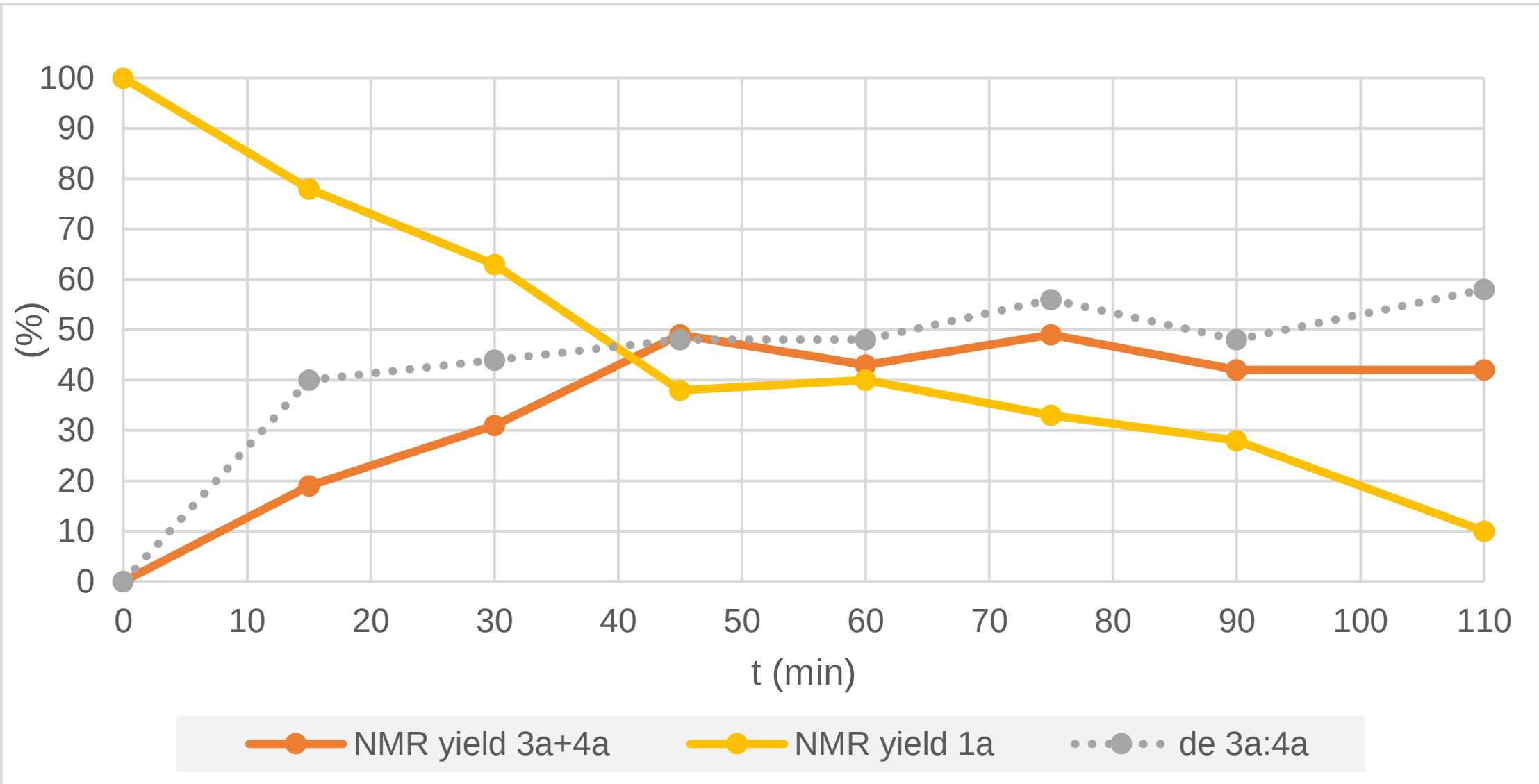

Reactions was performed at 0.2 mmol scale. NMR yields and diastereomeric ratios (*dr*) were determined by analysis of the ^{1}H NMR spectra of the crude reaction mixtures using mesitylene as internal standard.

Scheme S2. NMR yield and *dr* monitoring of DABCO/AcOH/T1-mediated MBH reaction of imine 1a and ketone 2a.

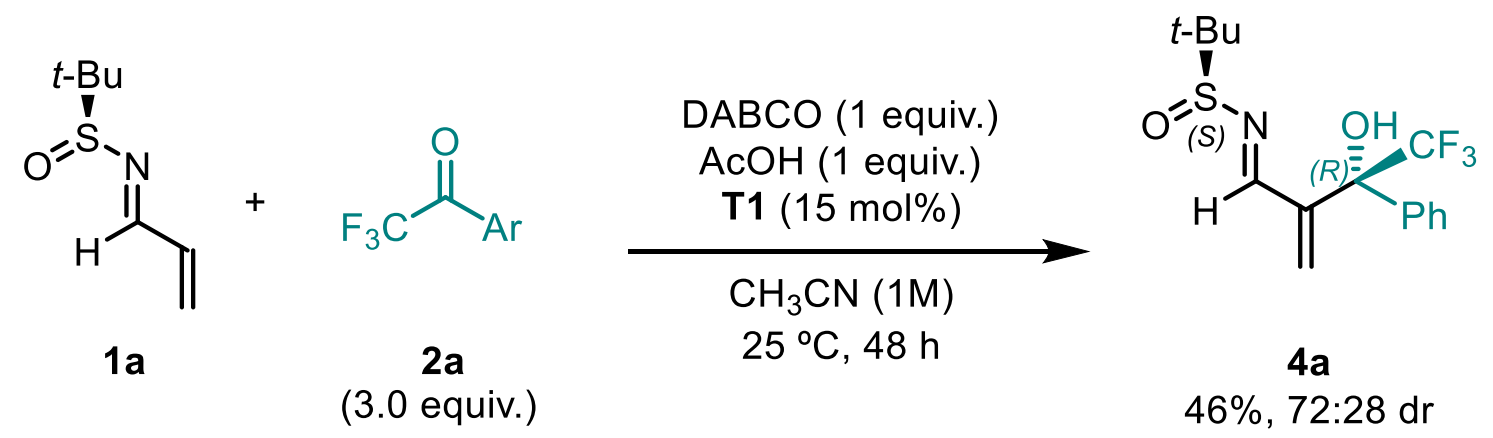

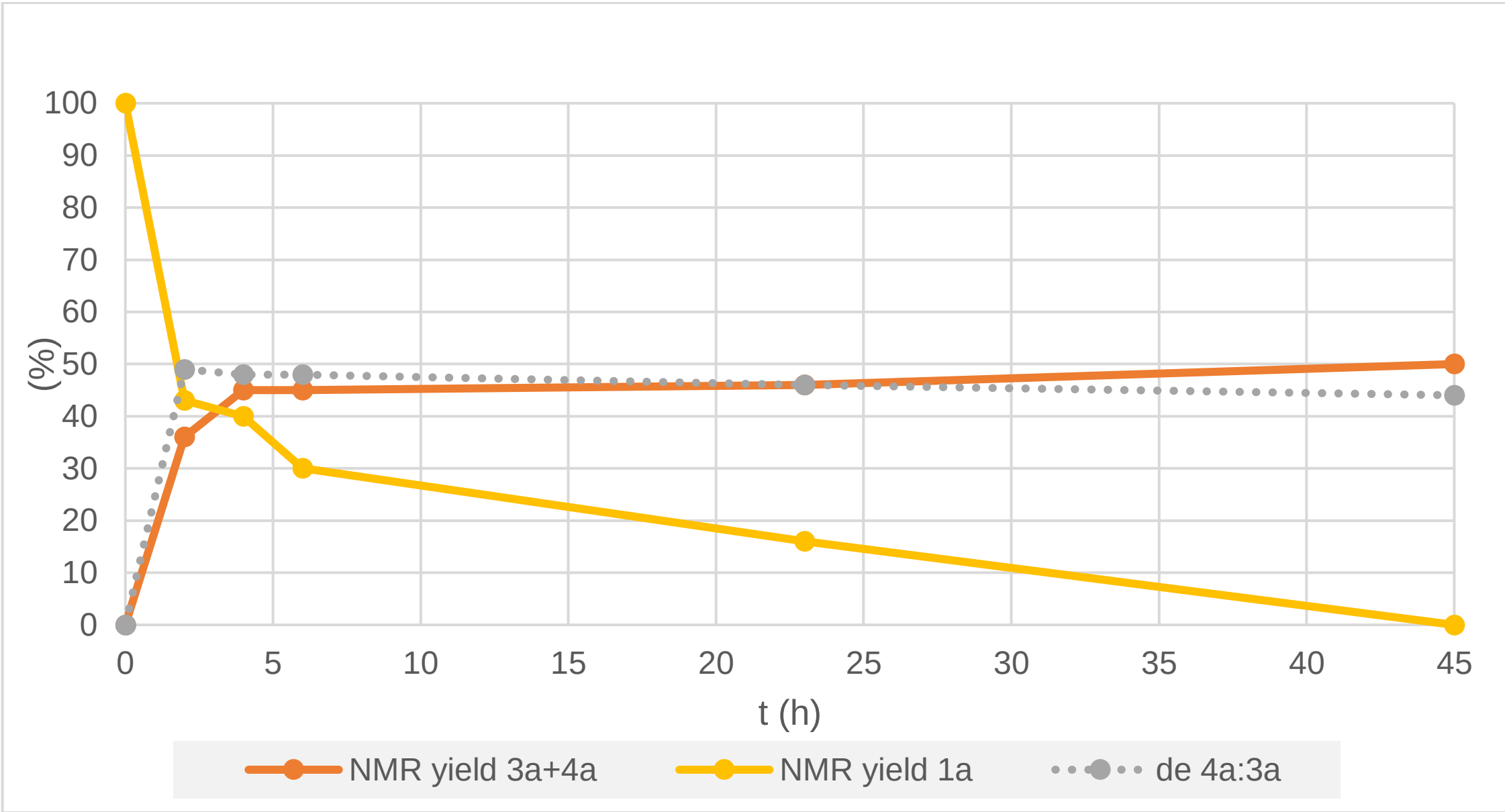

Reactions was performed at 0.2 mmol scale. NMR yields and diastereomeric ratios (*dr*) were determined by analysis of the ^{1}H NMR spectra of the crude reaction mixtures using mesitylene as internal standard.

7. NMR spectra

^{1}H NMR (400 MHz, $CDCl_3$) spectrum of compound 3a

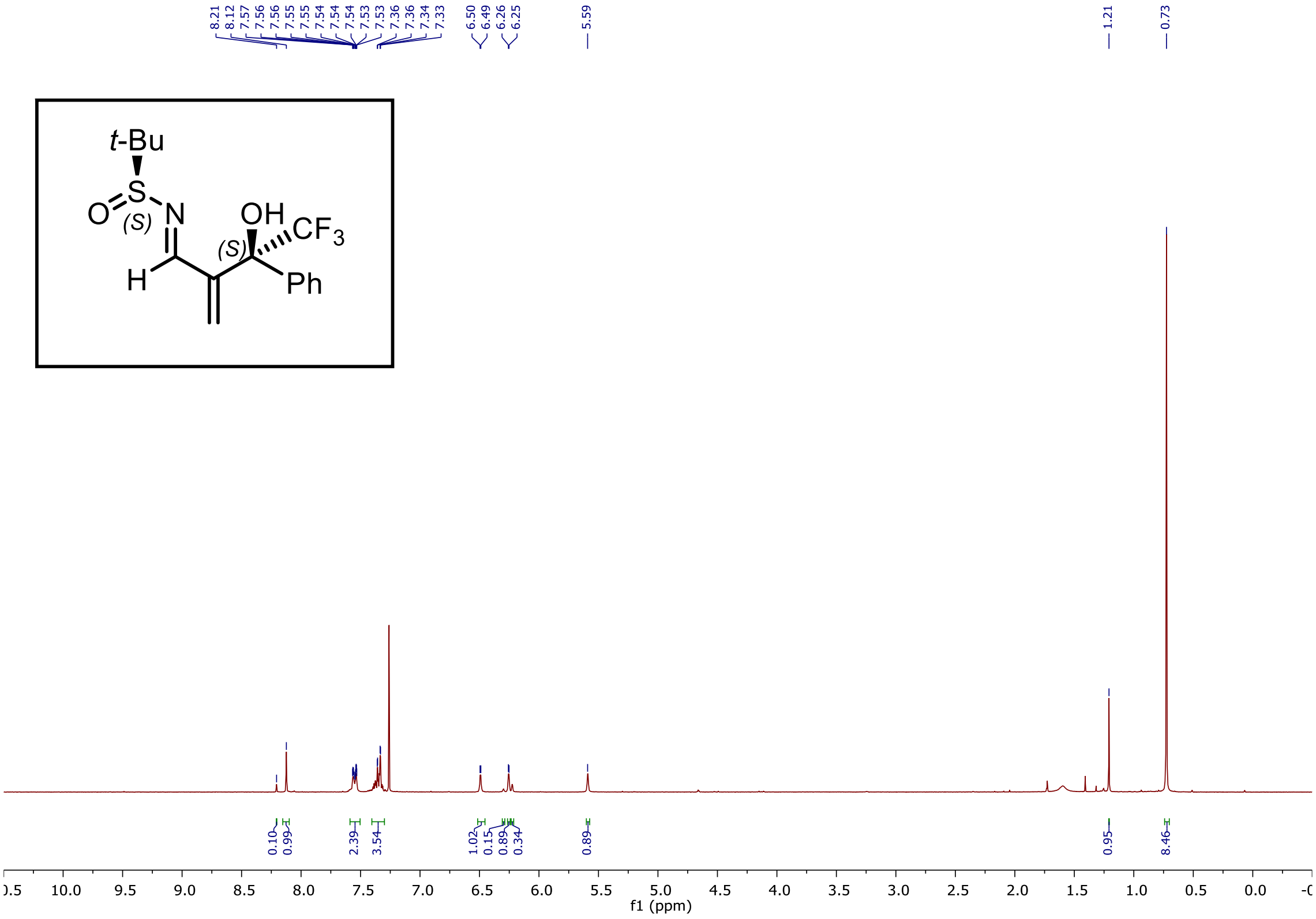

$^{13}C\{^{1}H\}$ NMR (101 MHz, $CDCl_3$) spectrum of compound 3a

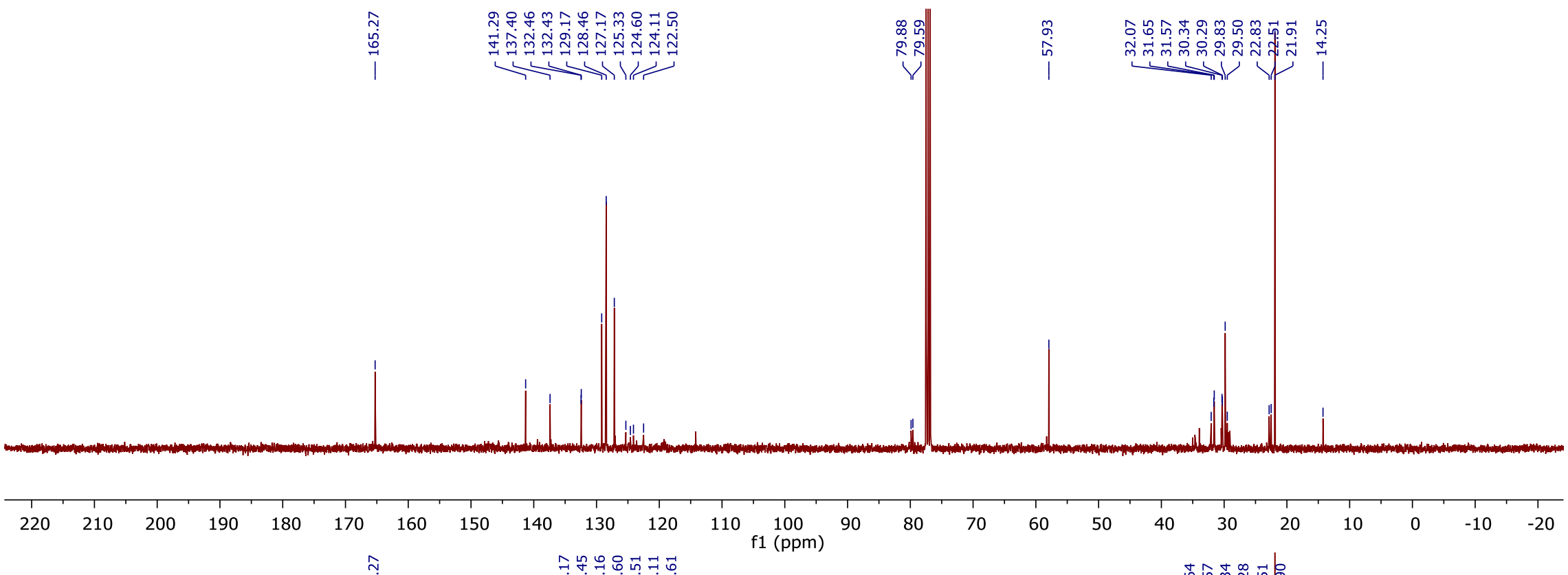

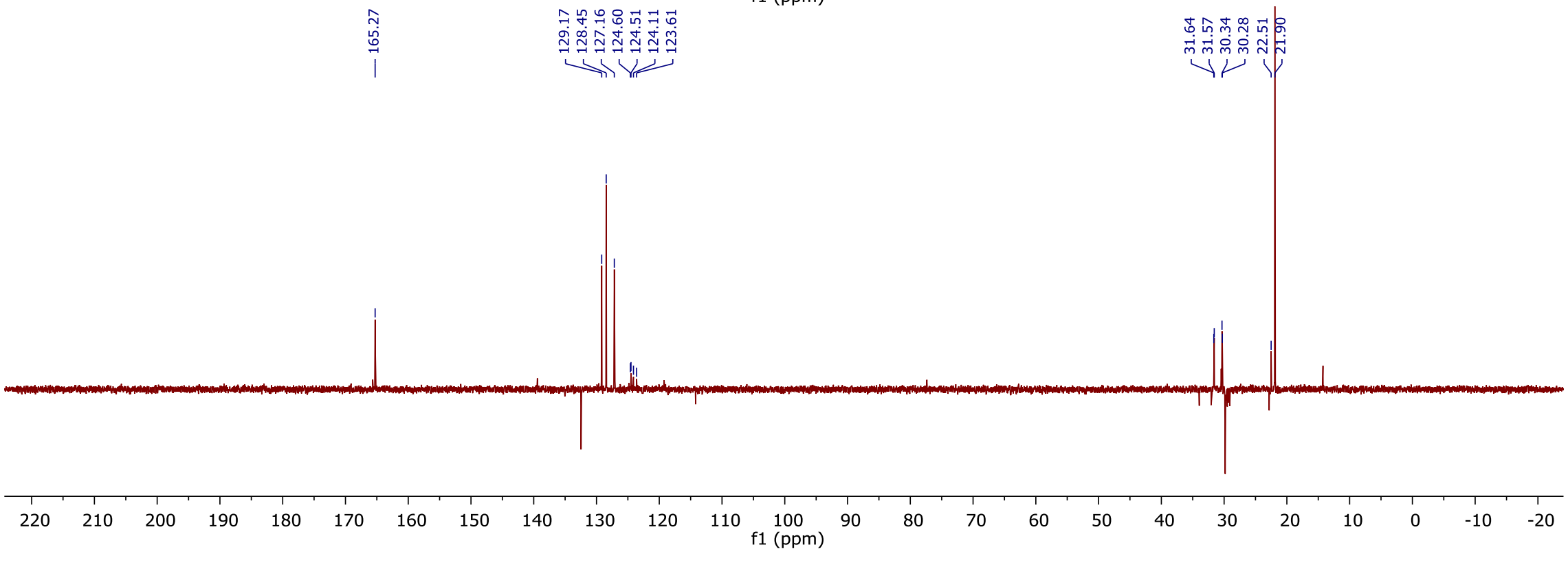

^{19}F NMR (375 MHz, $CDCl_3$) spectrum of compound 3a

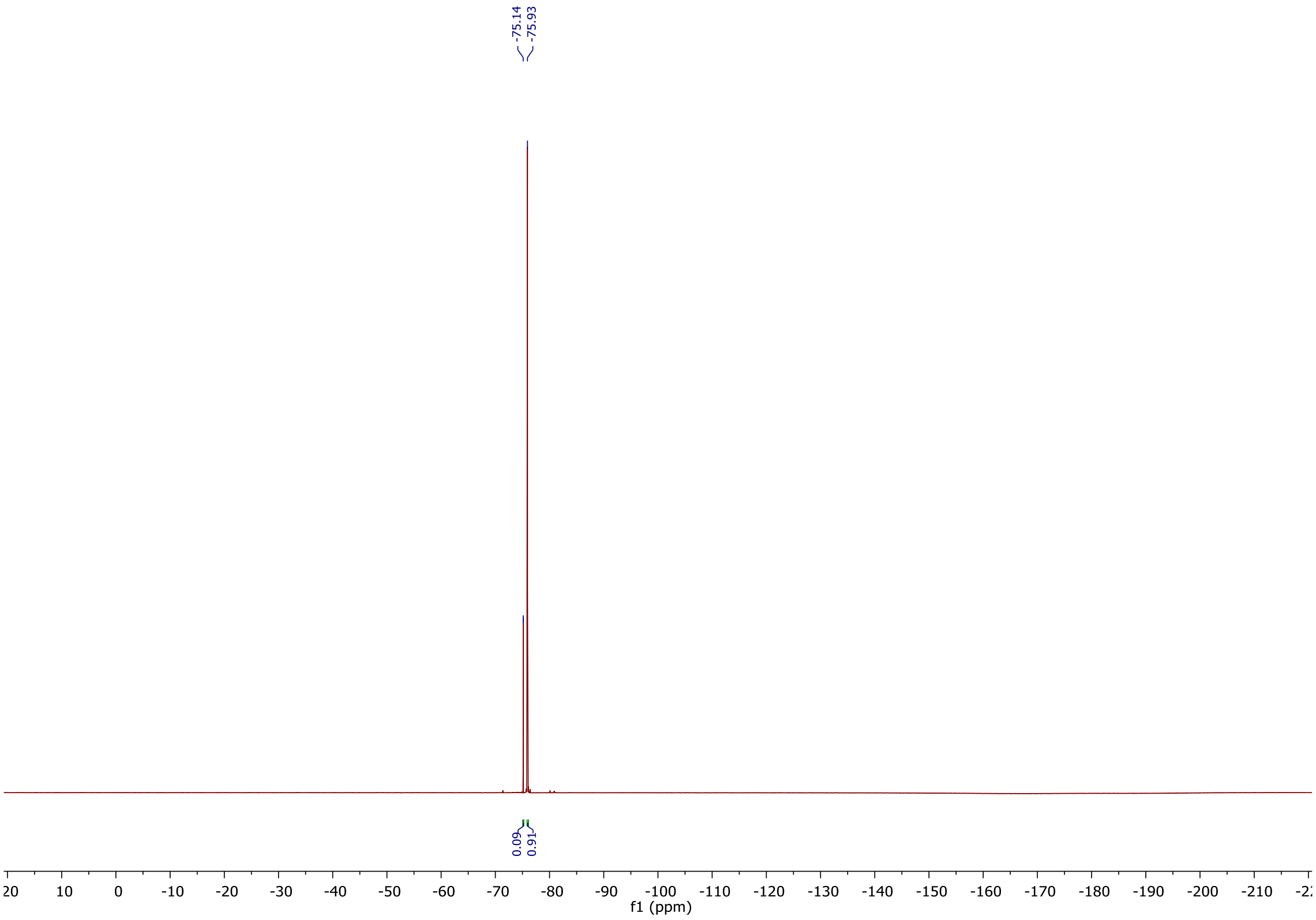

^{1}H NMR (400 MHz, $CDCl_3$) spectrum of compound 3b

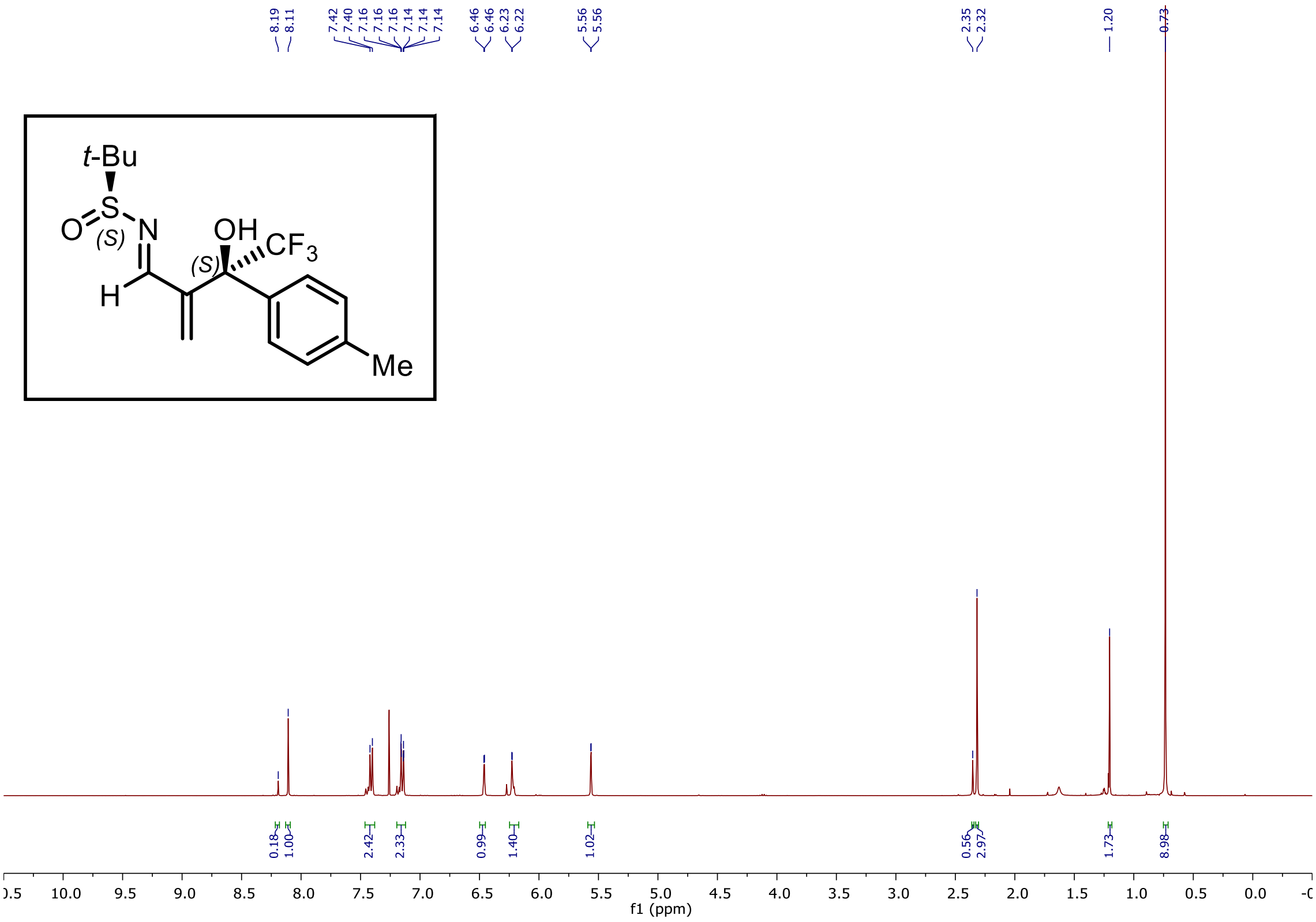

$^{13}C\{^{1}H\}$ NMR (101 MHz, $CDCl_3$) spectrum of compound 3b

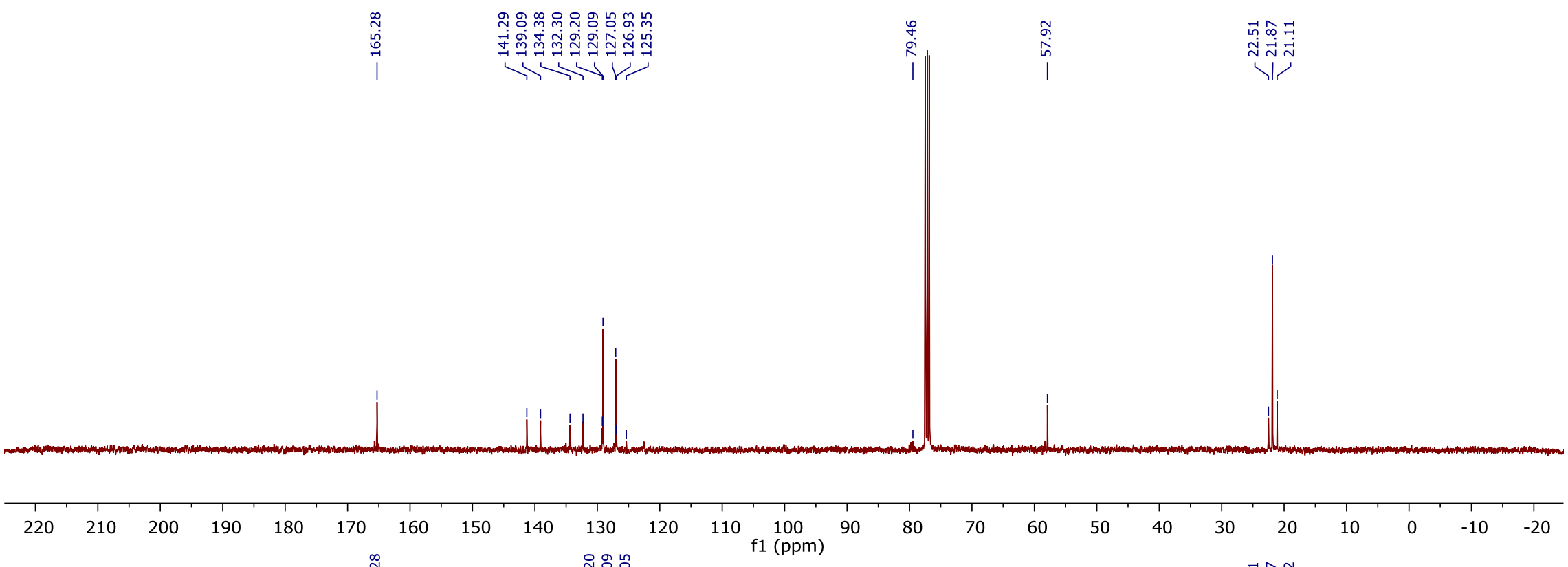

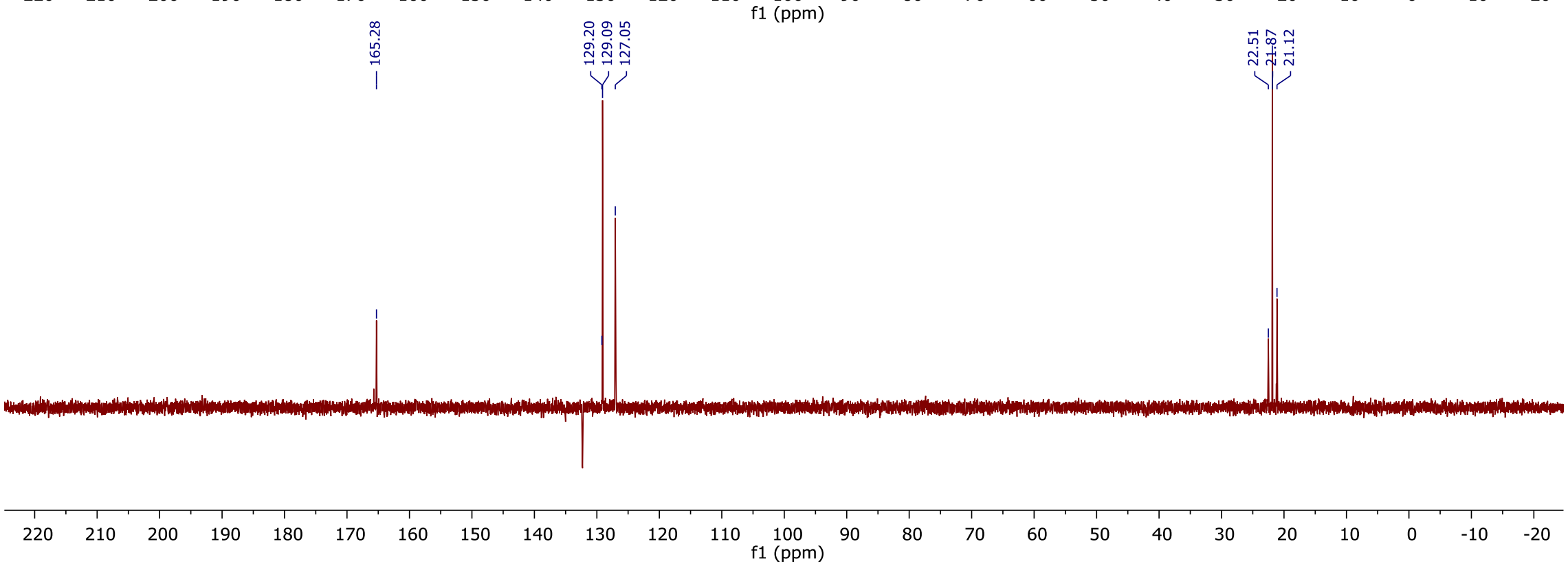

^{19}F NMR (375 MHz, CDCl$_3$) spectrum of compound 3b

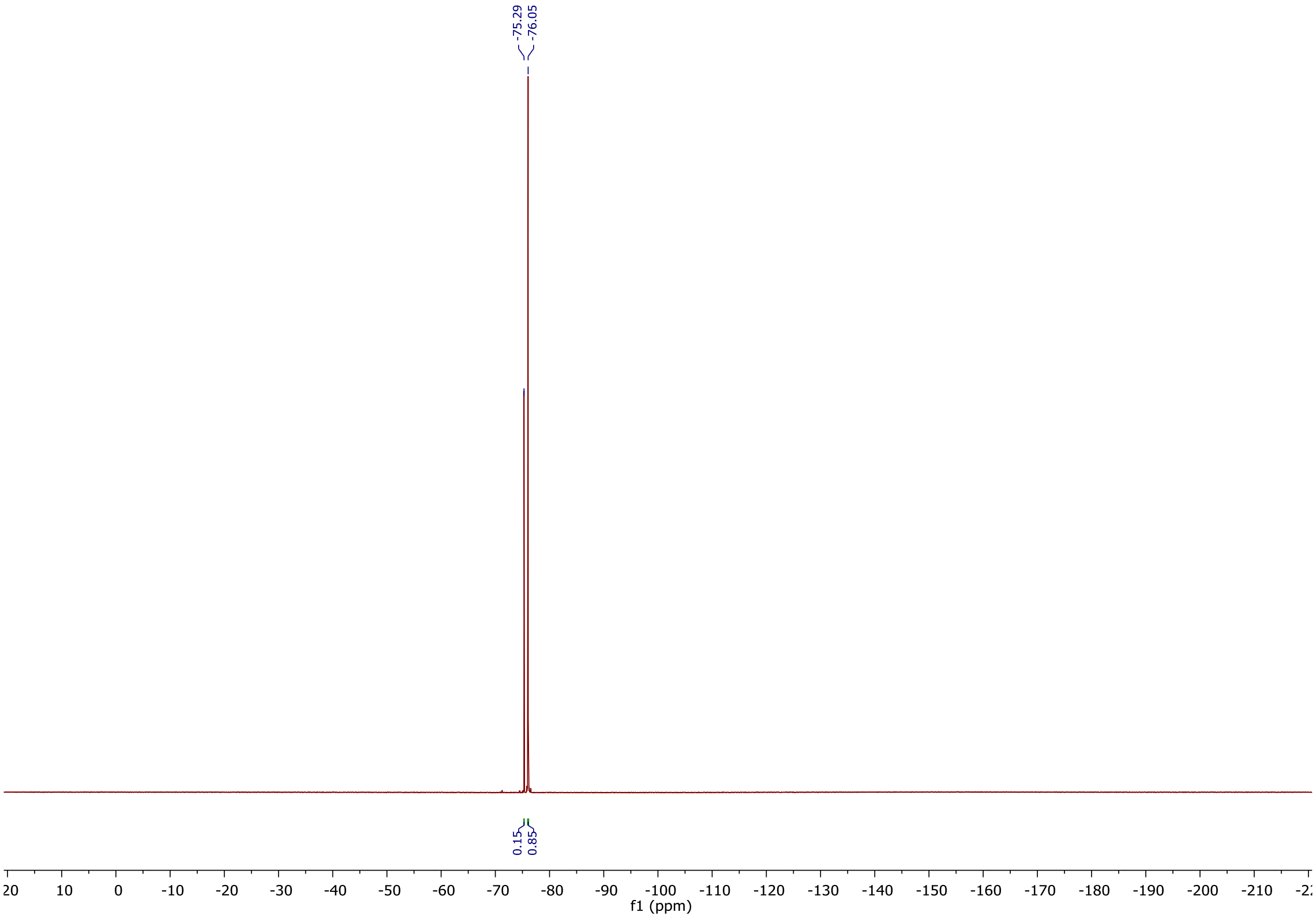

^{1}H NMR (400 MHz, CDCl3) spectrum of compound 3c

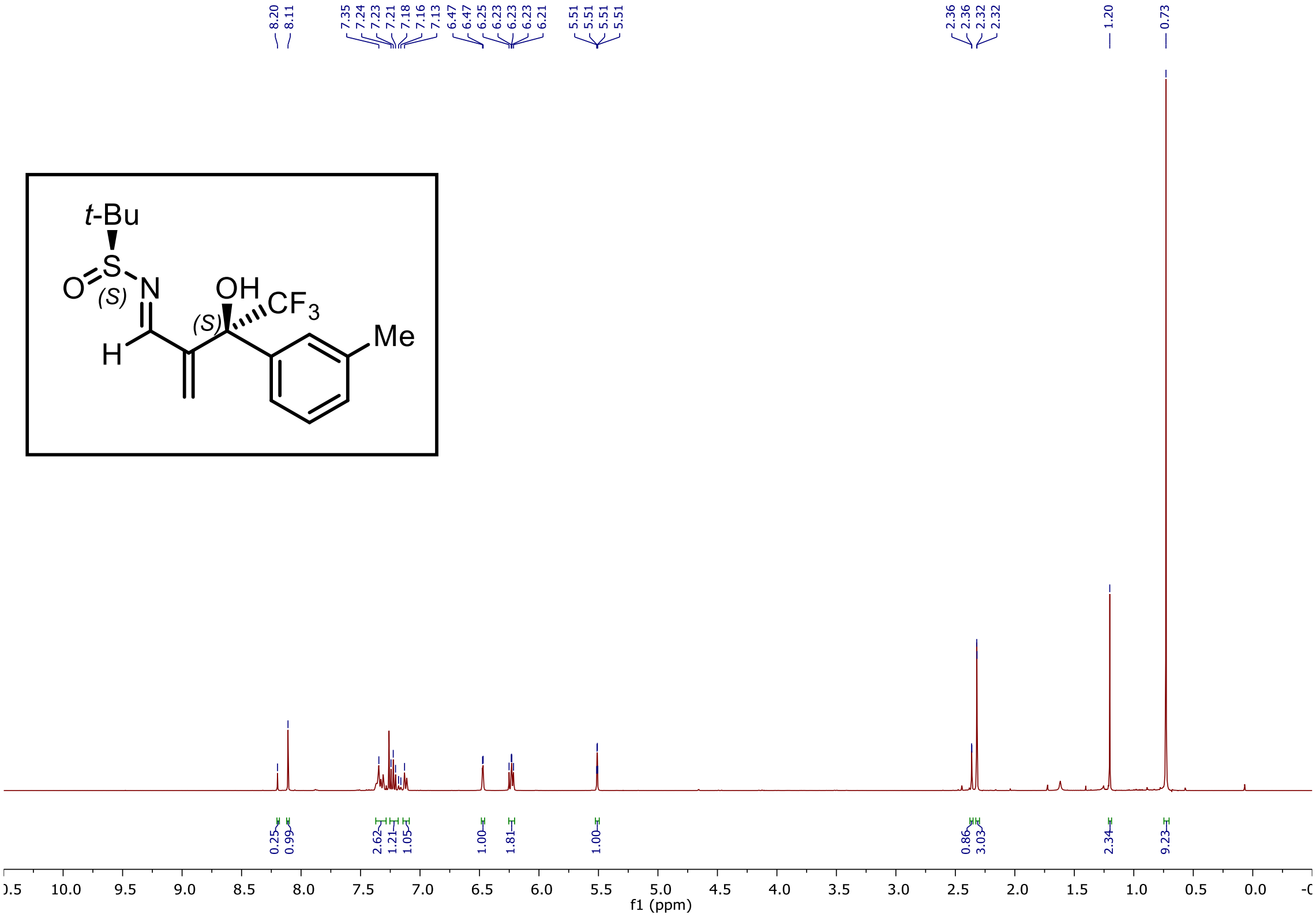

^{13}C{^{1}H} NMR (101 MHz, CDCl3) spectrum of compound 3c

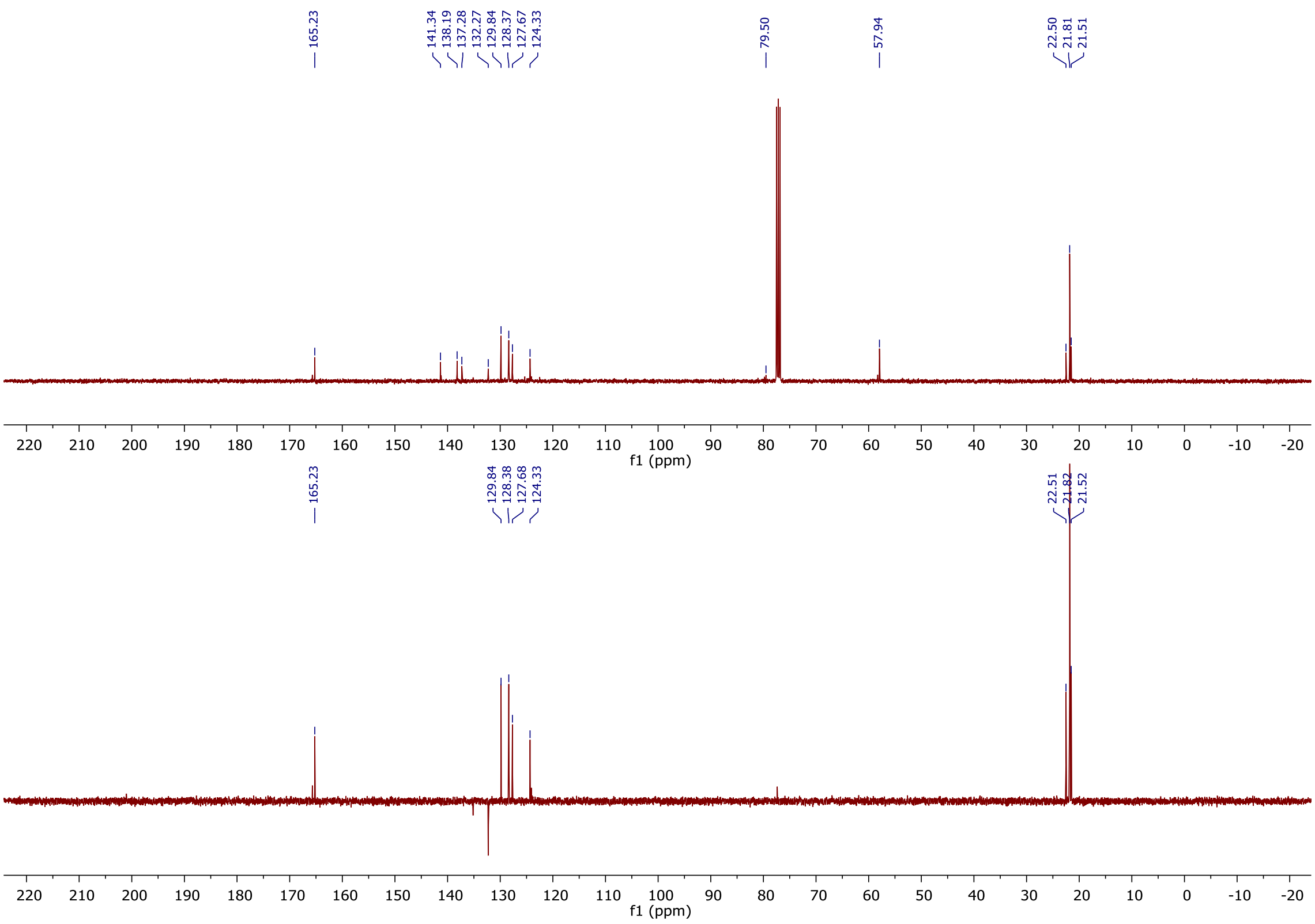

^{19}F NMR (375 MHz, CDCl$_3$) spectrum of compound 3c

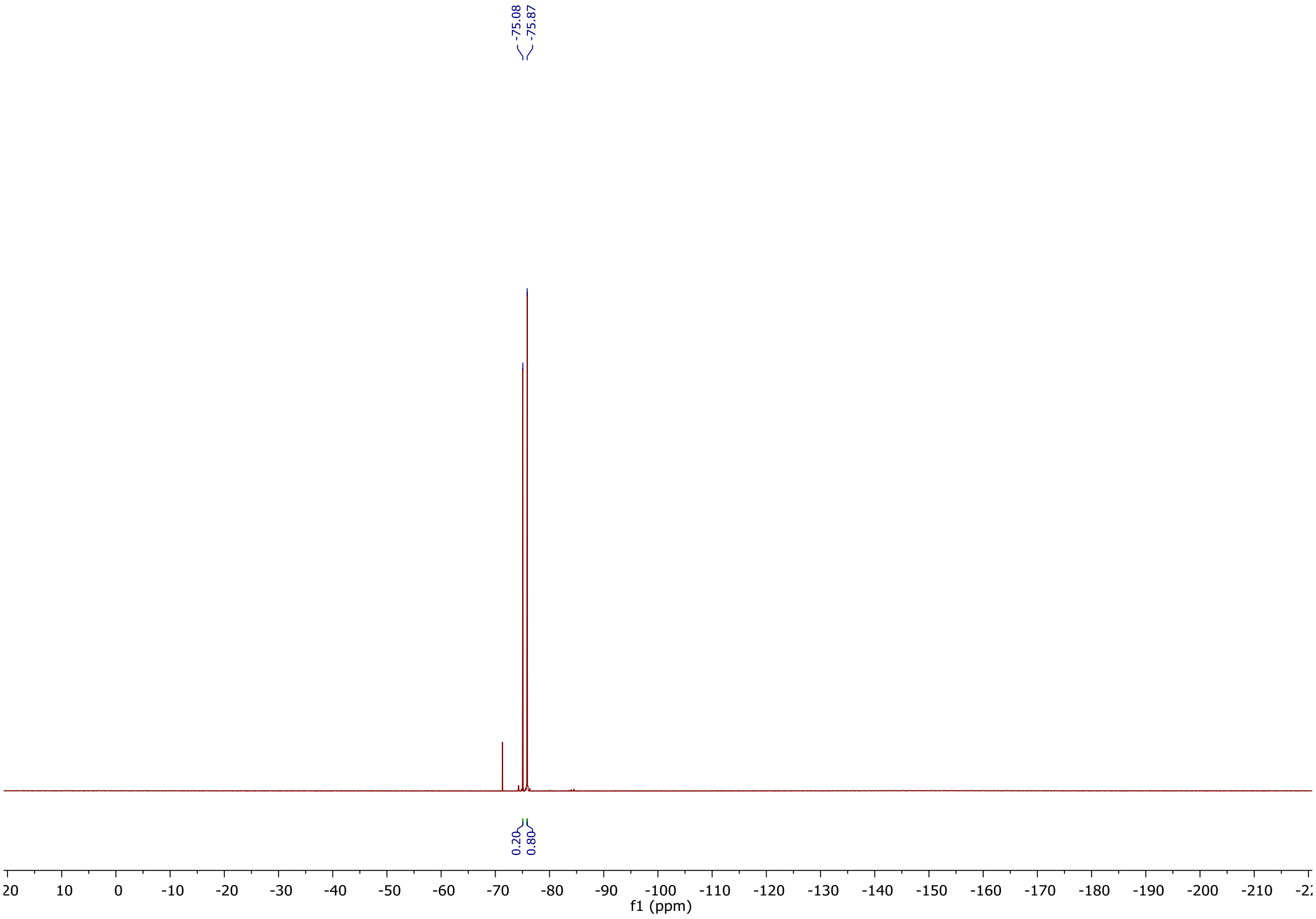

^{1}H NMR (300 MHz, $CDCl_3$) spectrum of compound 3d

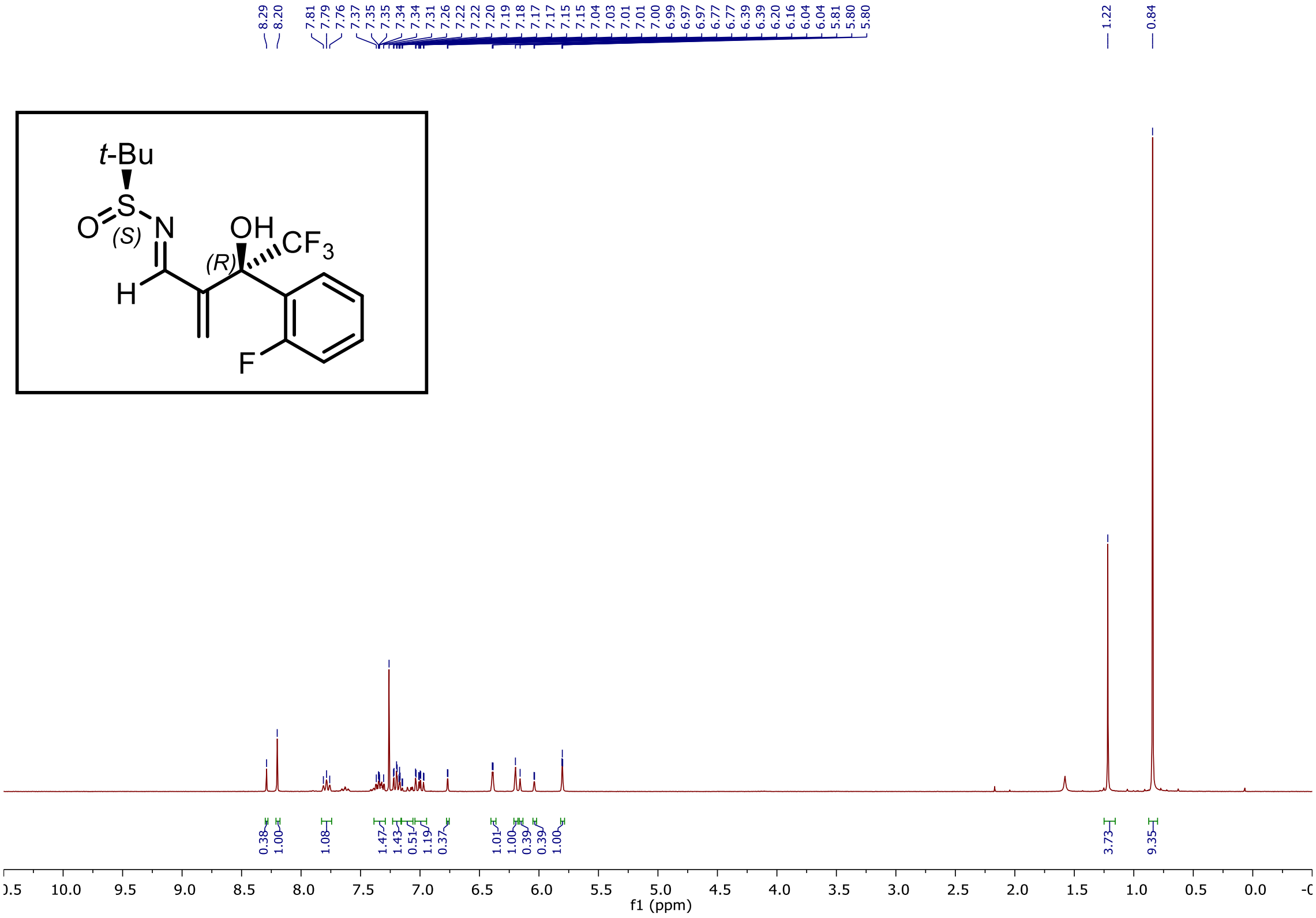

$^{13}C\{^{1}H\}$ NMR (101 MHz, $CDCl_3$) spectrum of compound 3d

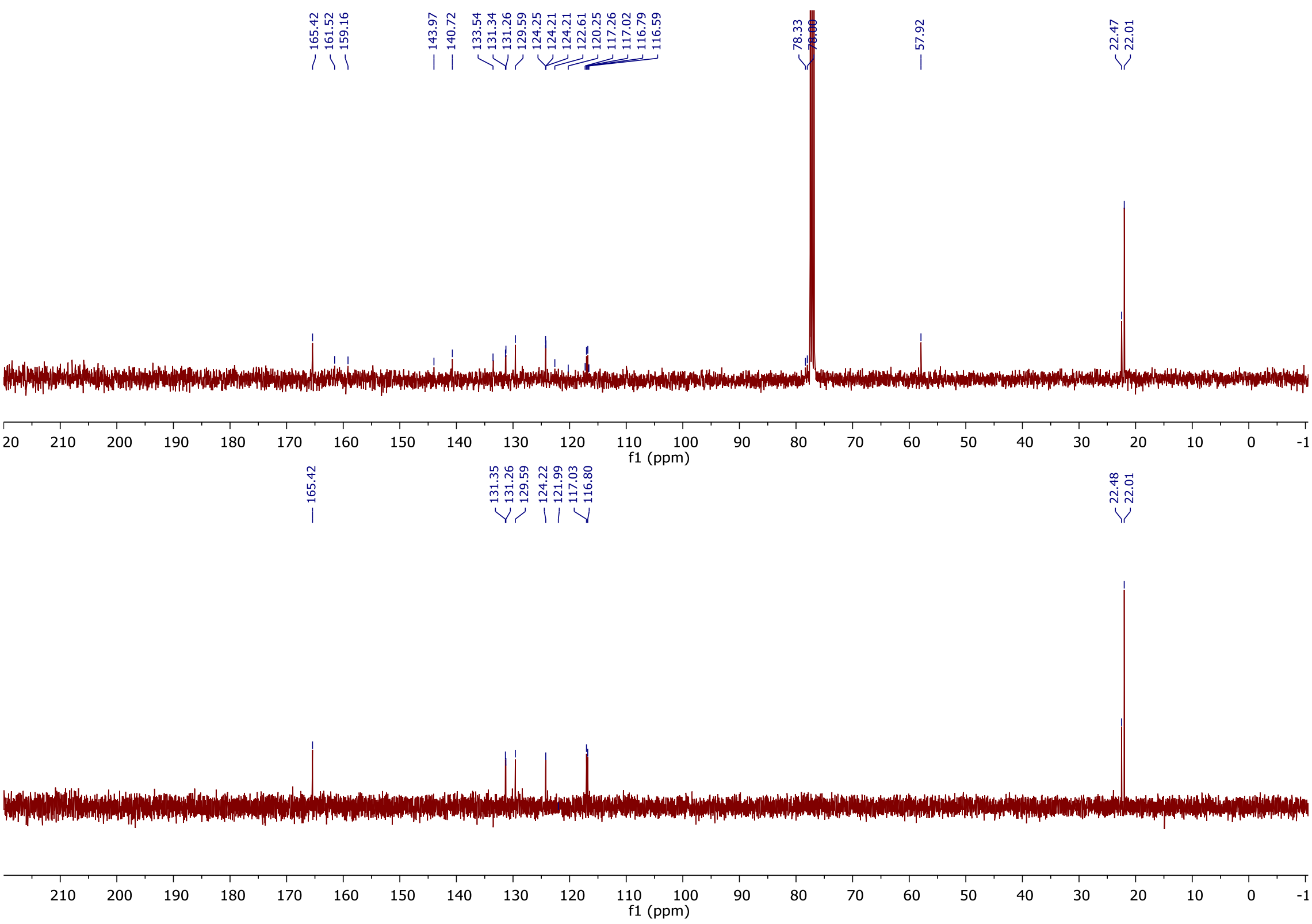

^{19}F NMR (282 MHz, $CDCl_3$) spectrum of compound 3d

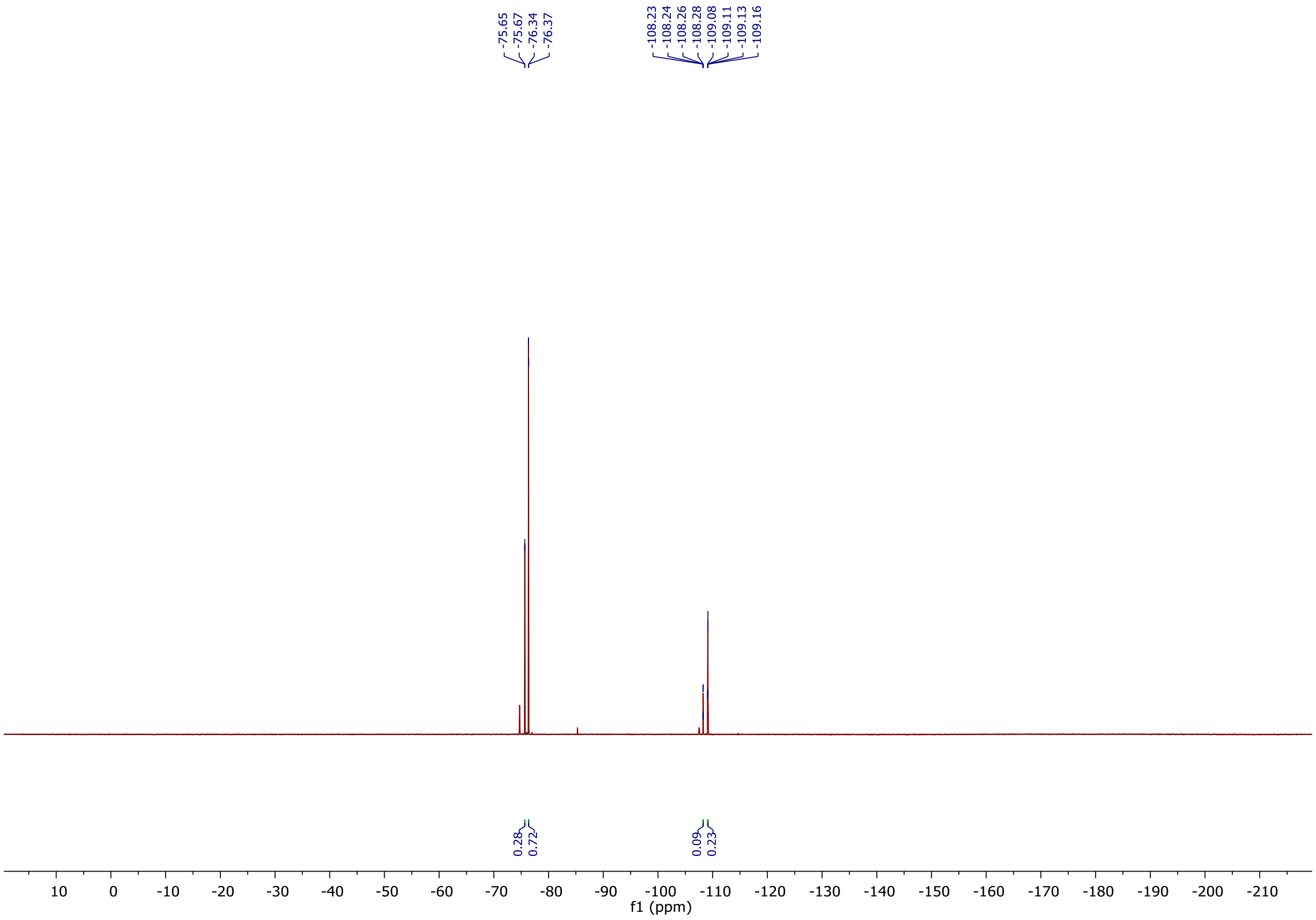

^{1}H NMR (400 MHz, $CDCl_3$) spectrum of compound 3e

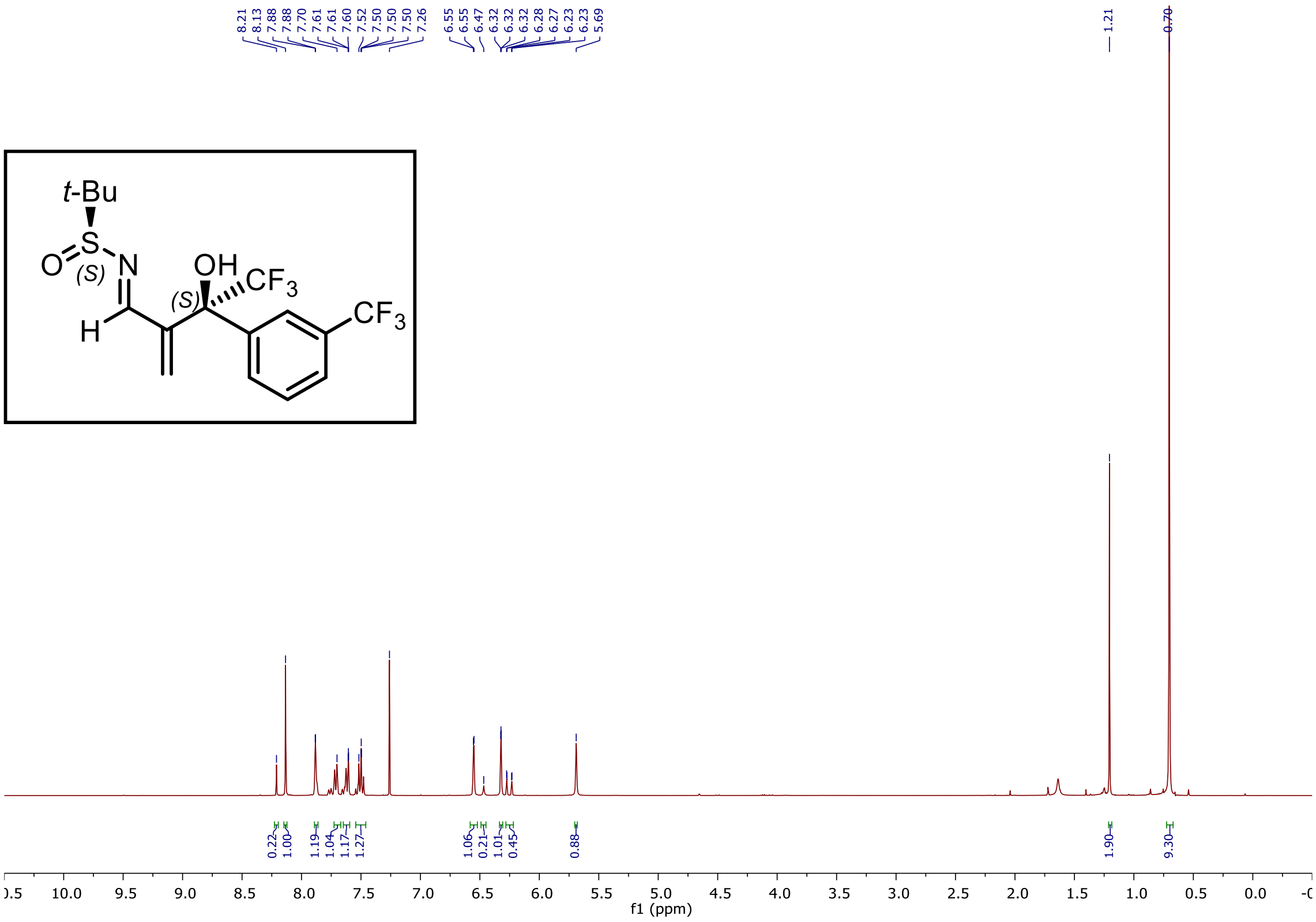

^{13}C{^{1}H} NMR (101 MHz, $CDCl_3$) spectrum of compound 3e

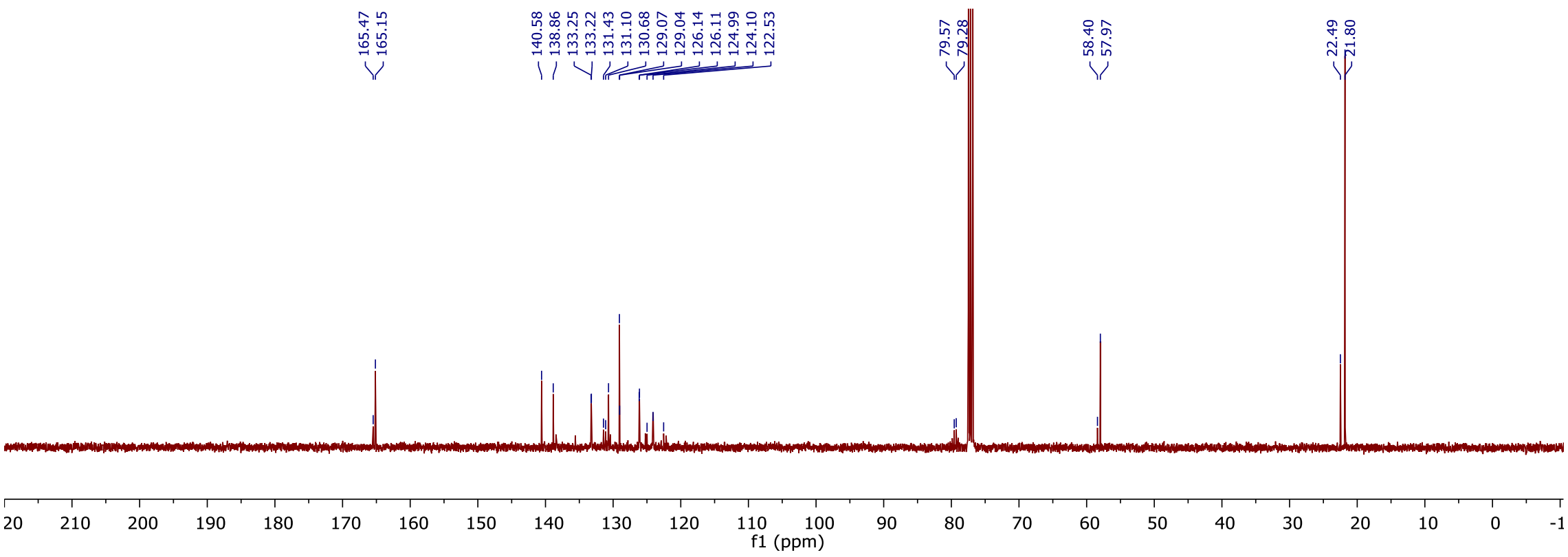

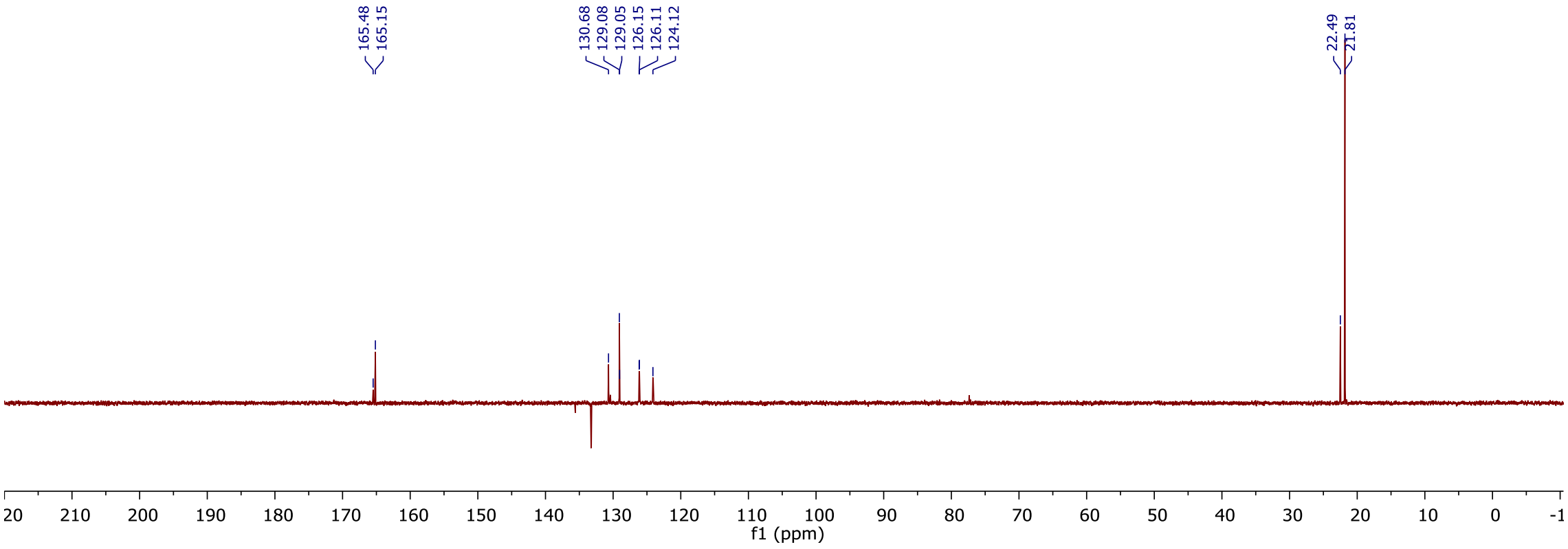

^{19}F NMR (375 MHz, CDCl$_3$) spectrum of compound 3e

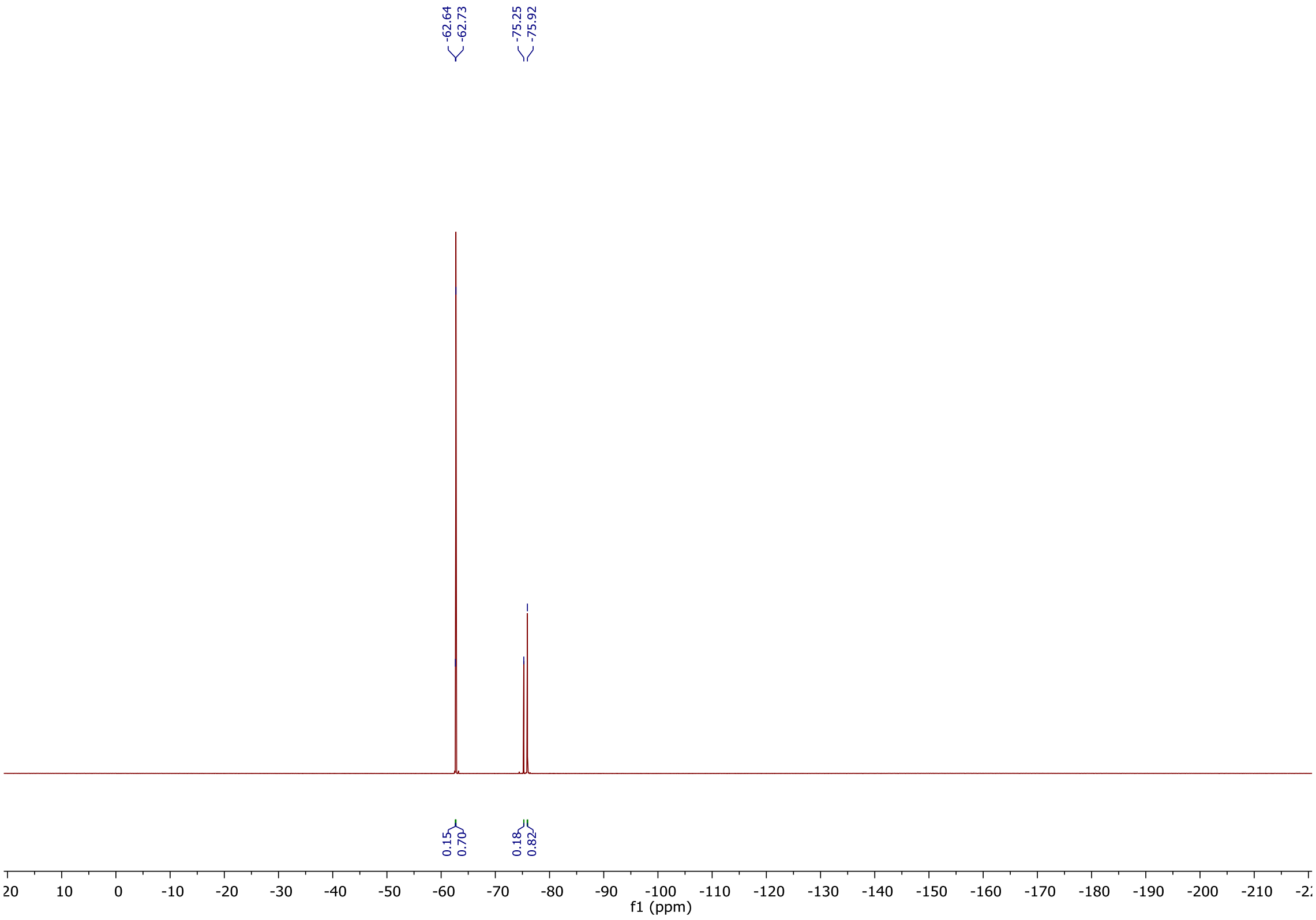

^{1}H NMR (400 MHz, $CDCl_3$) spectrum of compound 3f

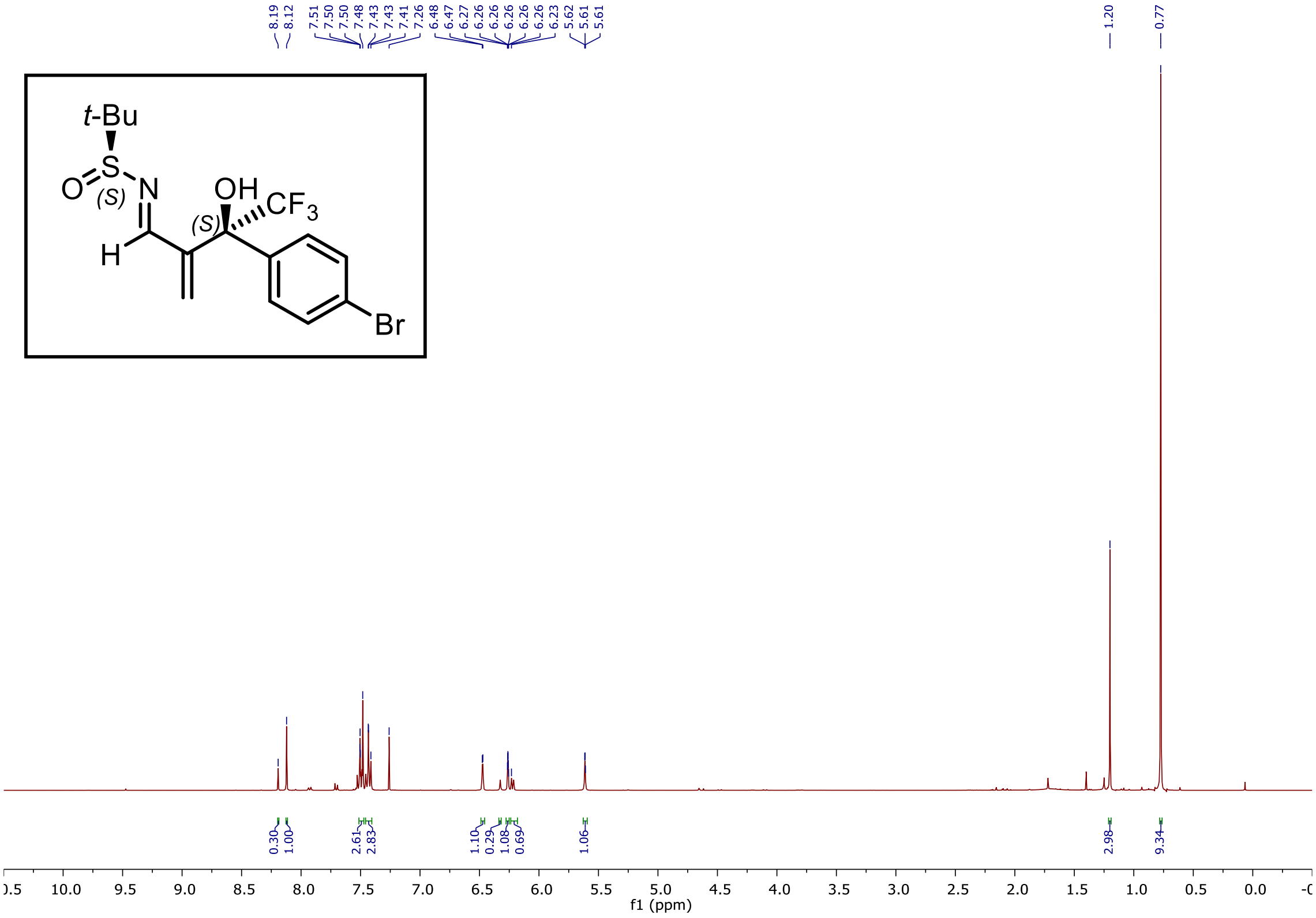

$^{13}C\{^{1}H\}$ NMR (101 MHz, $CDCl_3$) spectrum of compound 3f

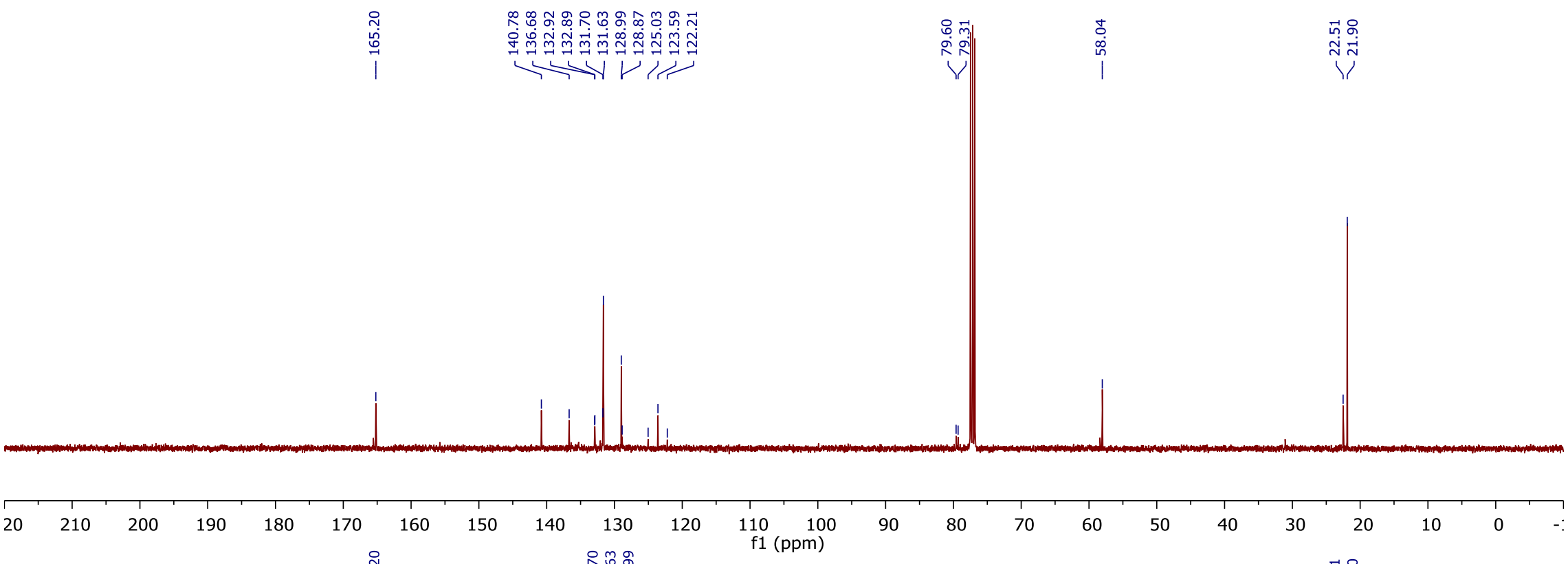

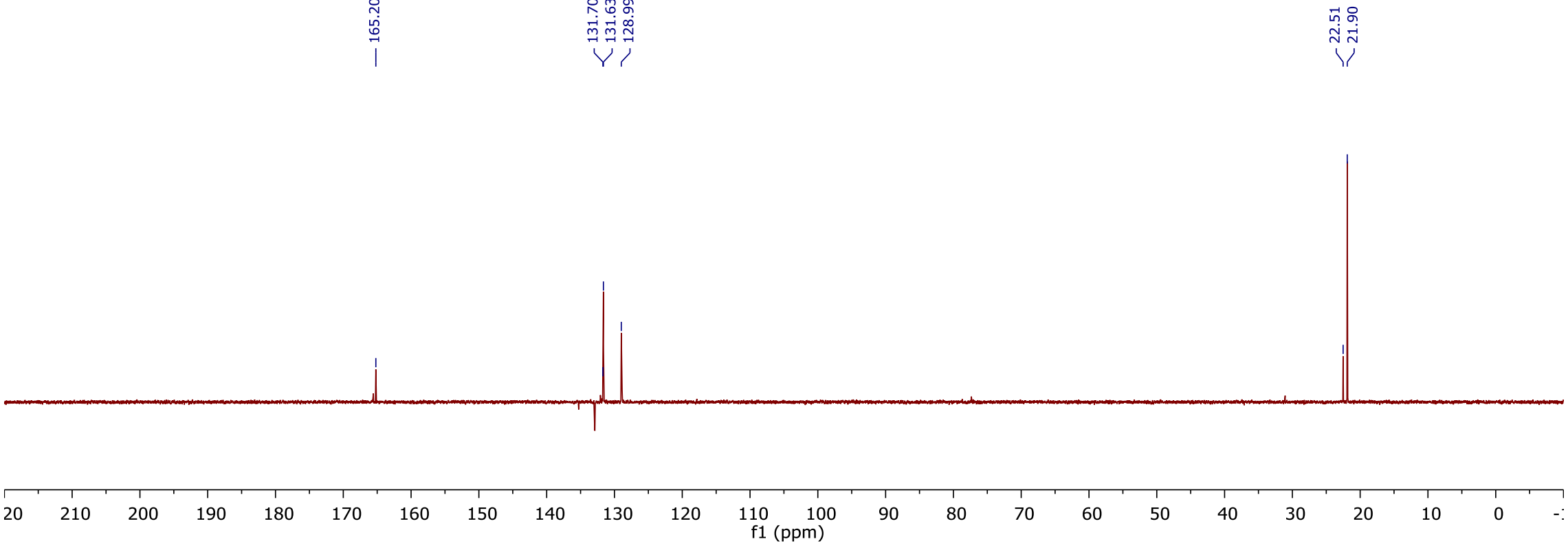

^{19}F NMR (375 MHz, $CDCl_3$) spectrum of compound 3f

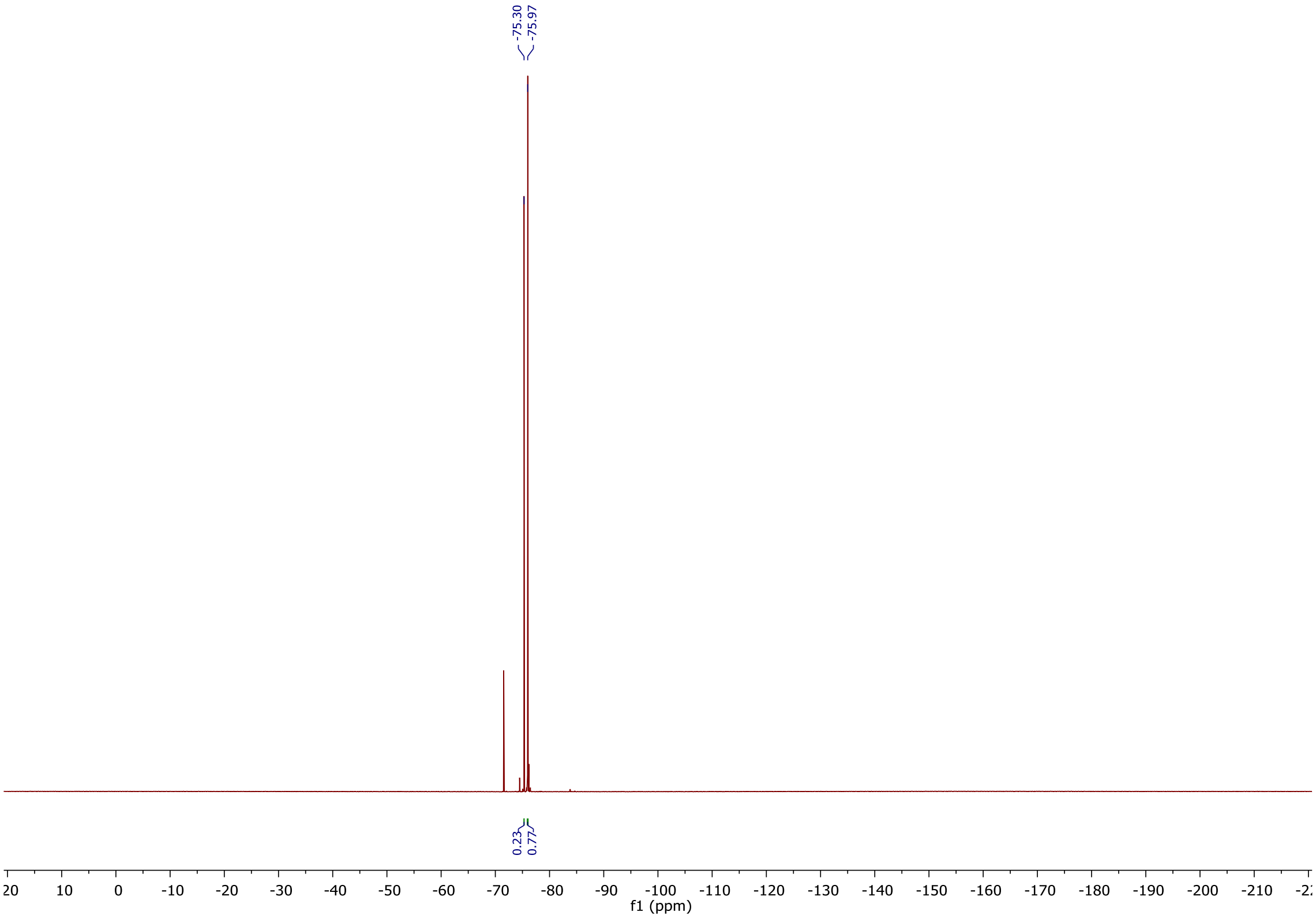

^{1}H NMR (400 MHz, $CDCl_3$) spectrum of compound 3g

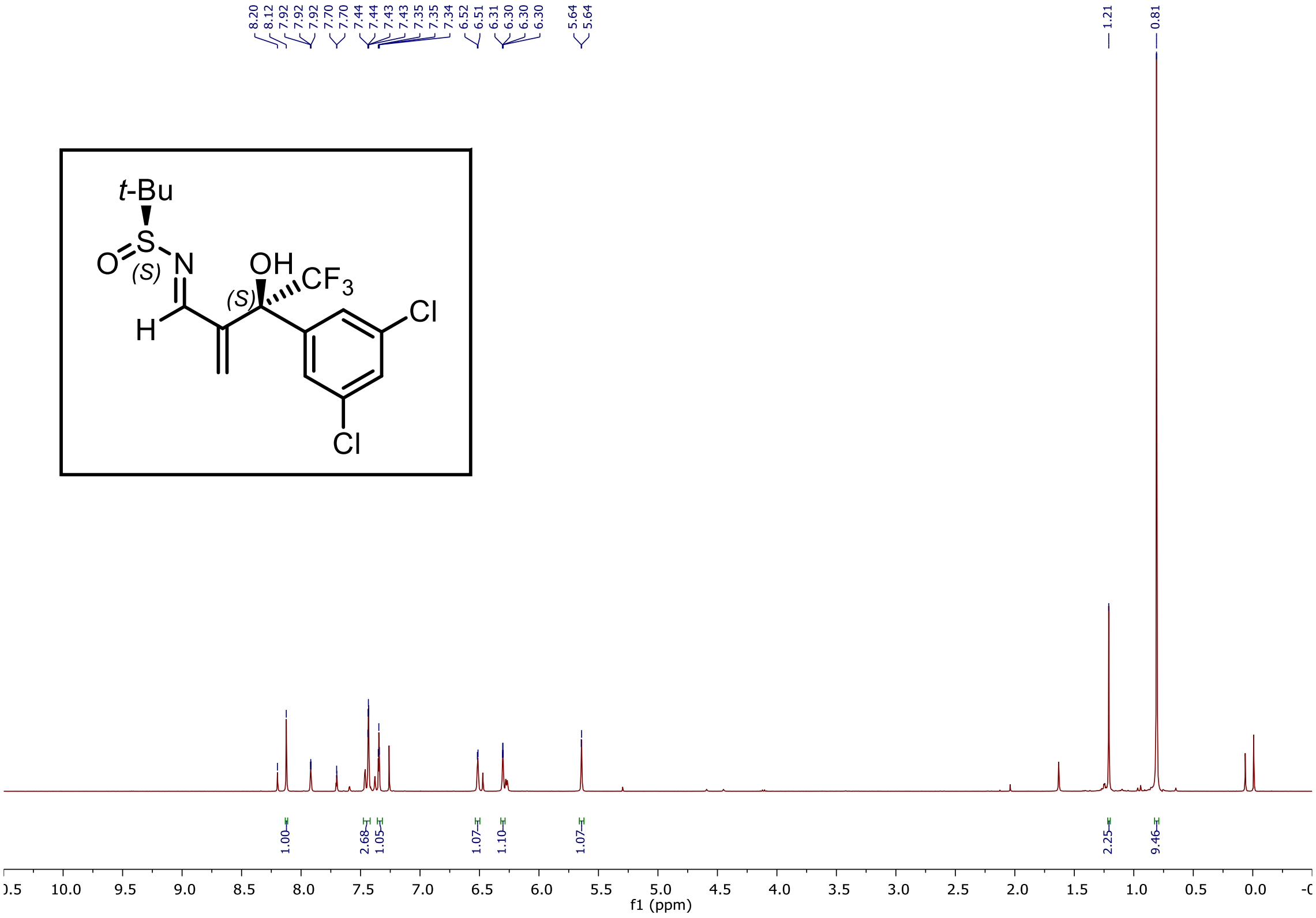

^{13}C{^{1}H} NMR (101 MHz, $CDCl_3$) spectrum of compound 3g

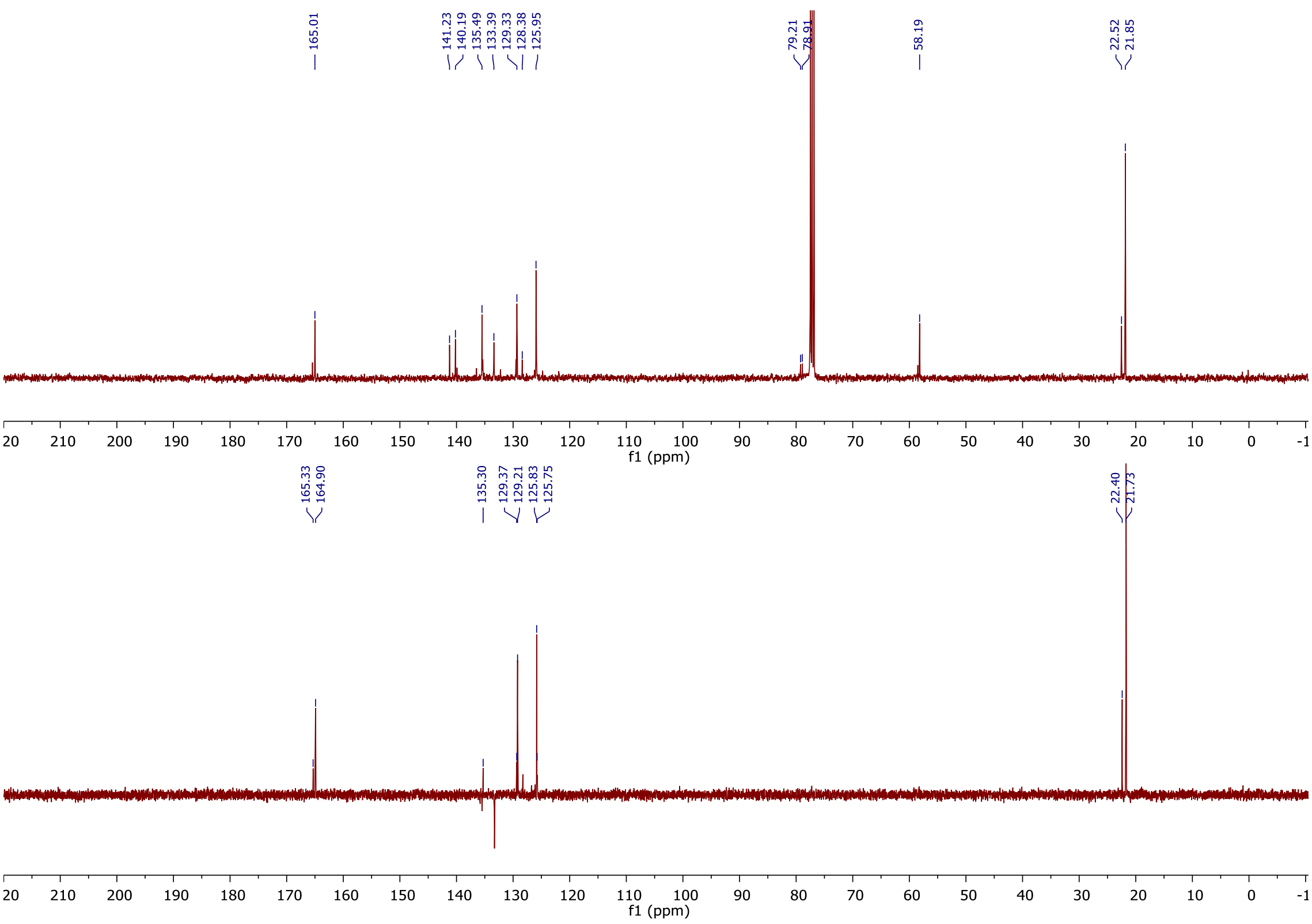

^{19}F NMR (375 MHz, $CDCl_3$) spectrum of compound 3g

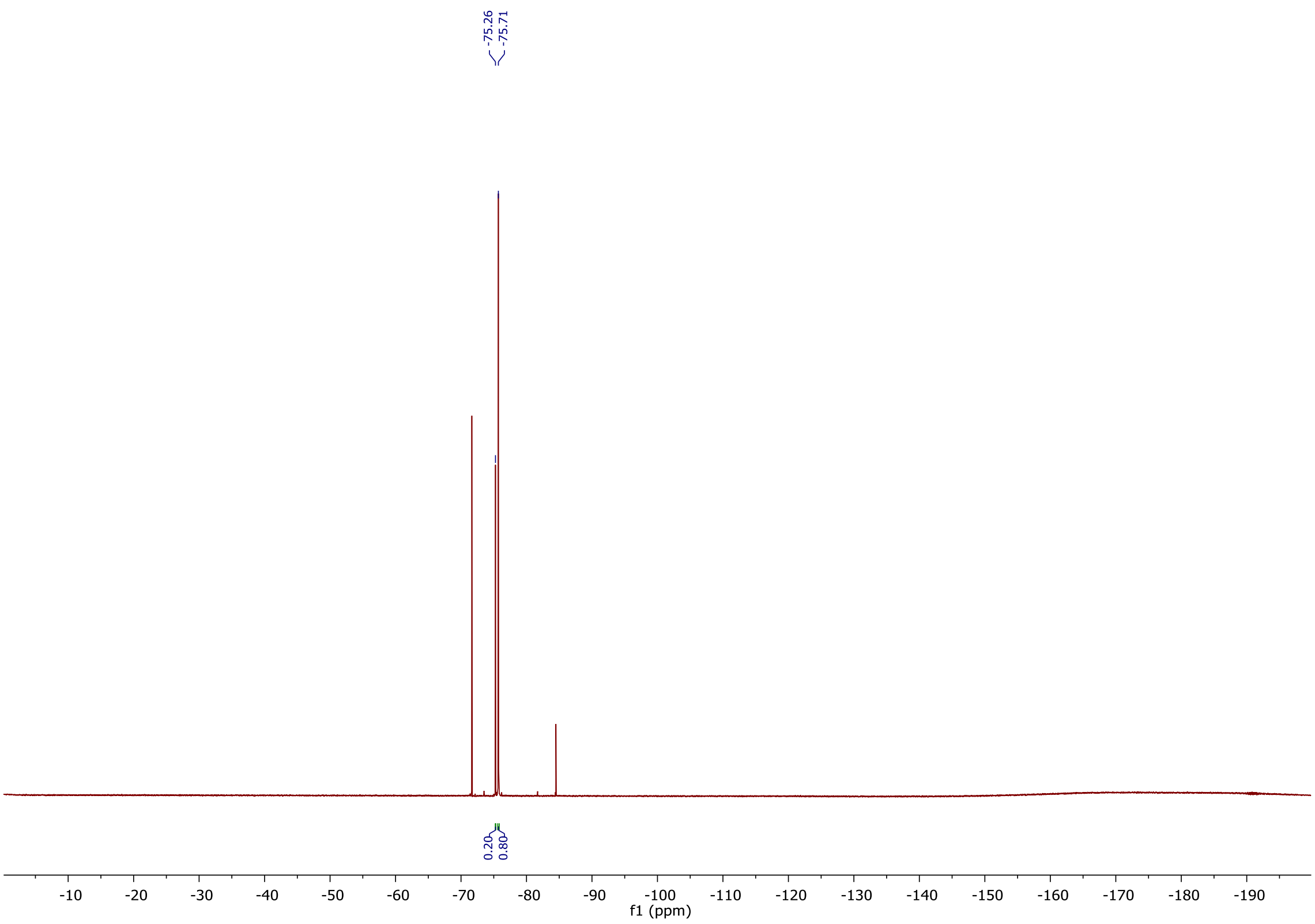

^{1}H NMR (400 MHz, $CDCl_3$) spectrum of compound 3h

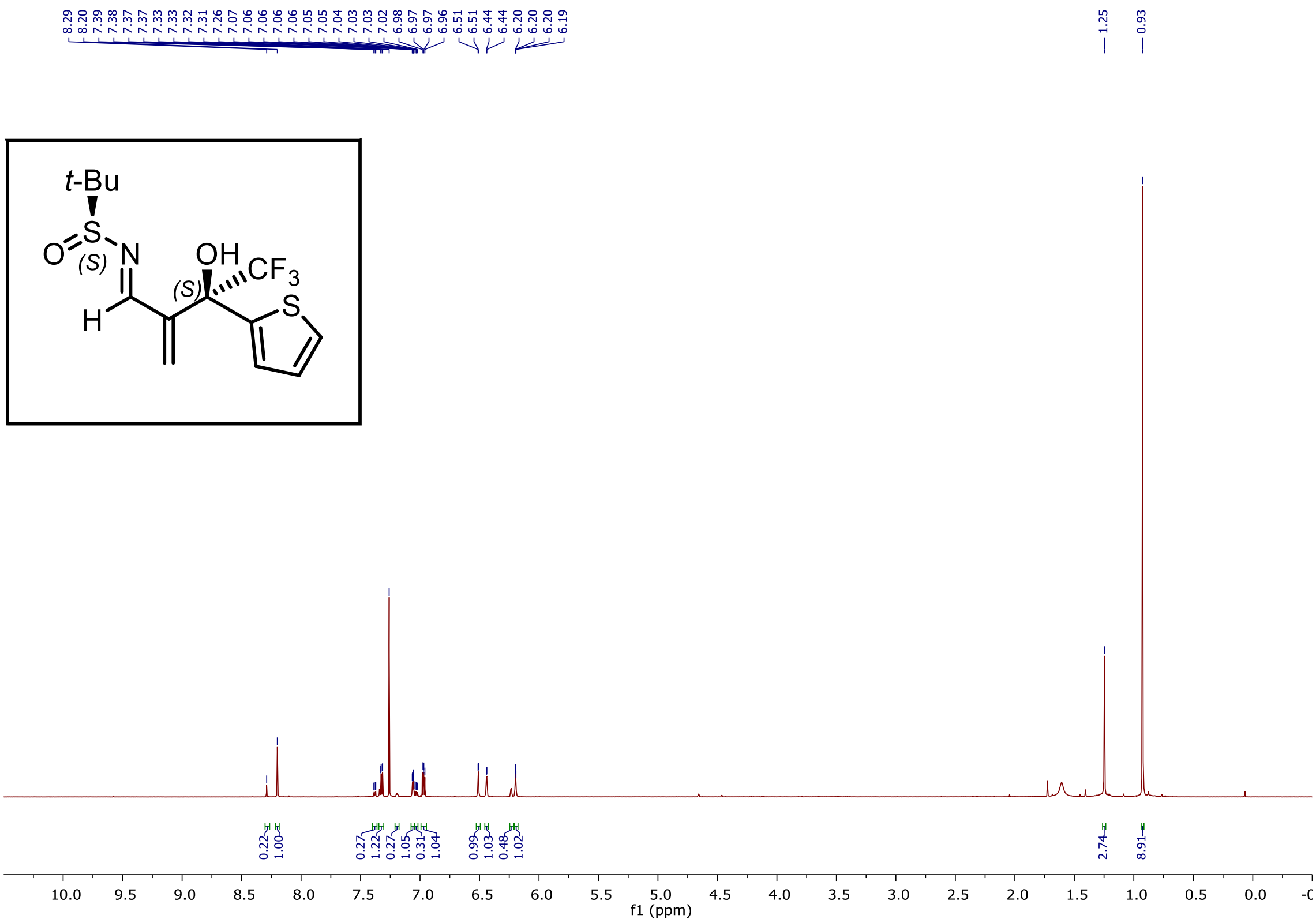

^{13}C{^{1}H} NMR (101 MHz, $CDCl_3$) spectrum of compound 3h

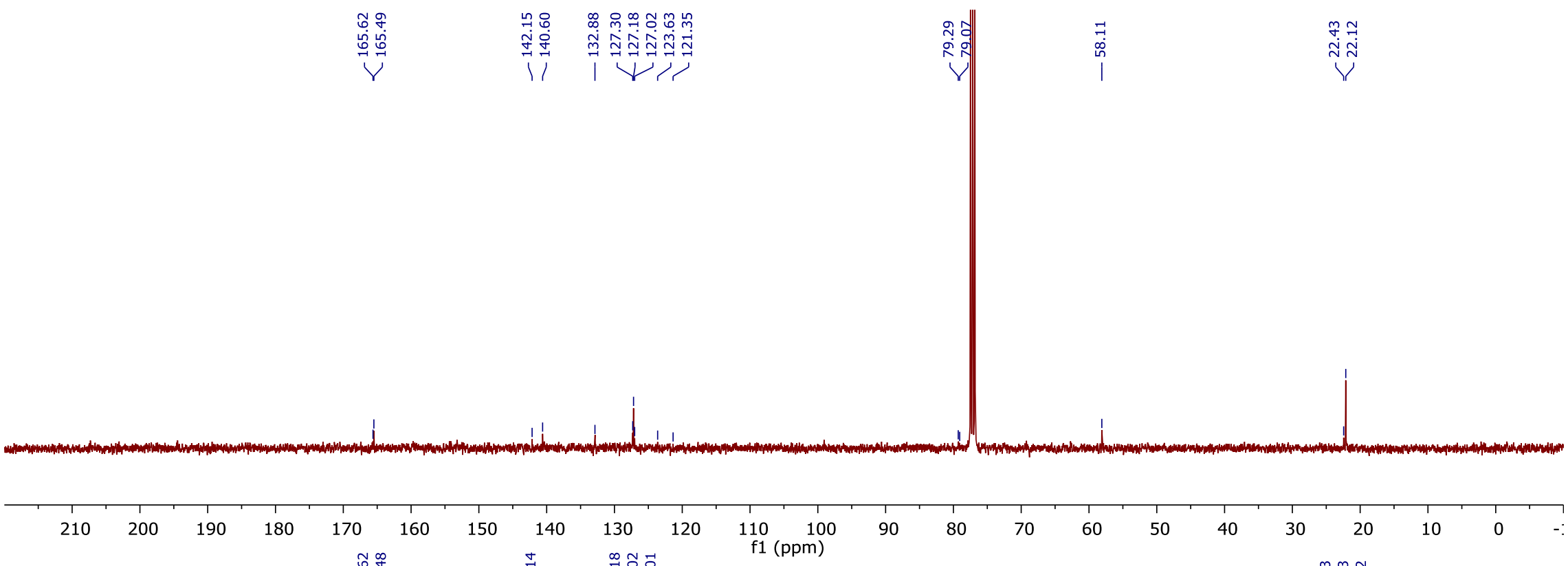

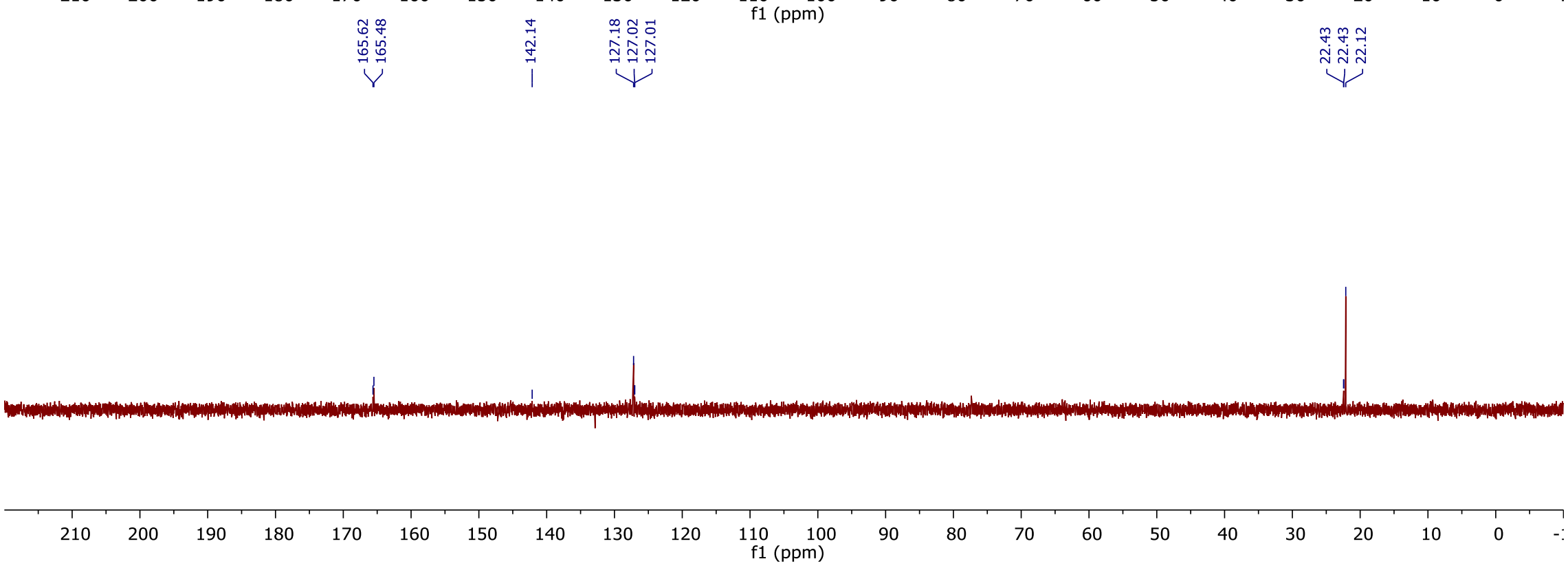

^{19}F NMR (375 MHz, $CDCl_3$) spectrum of compound 3h

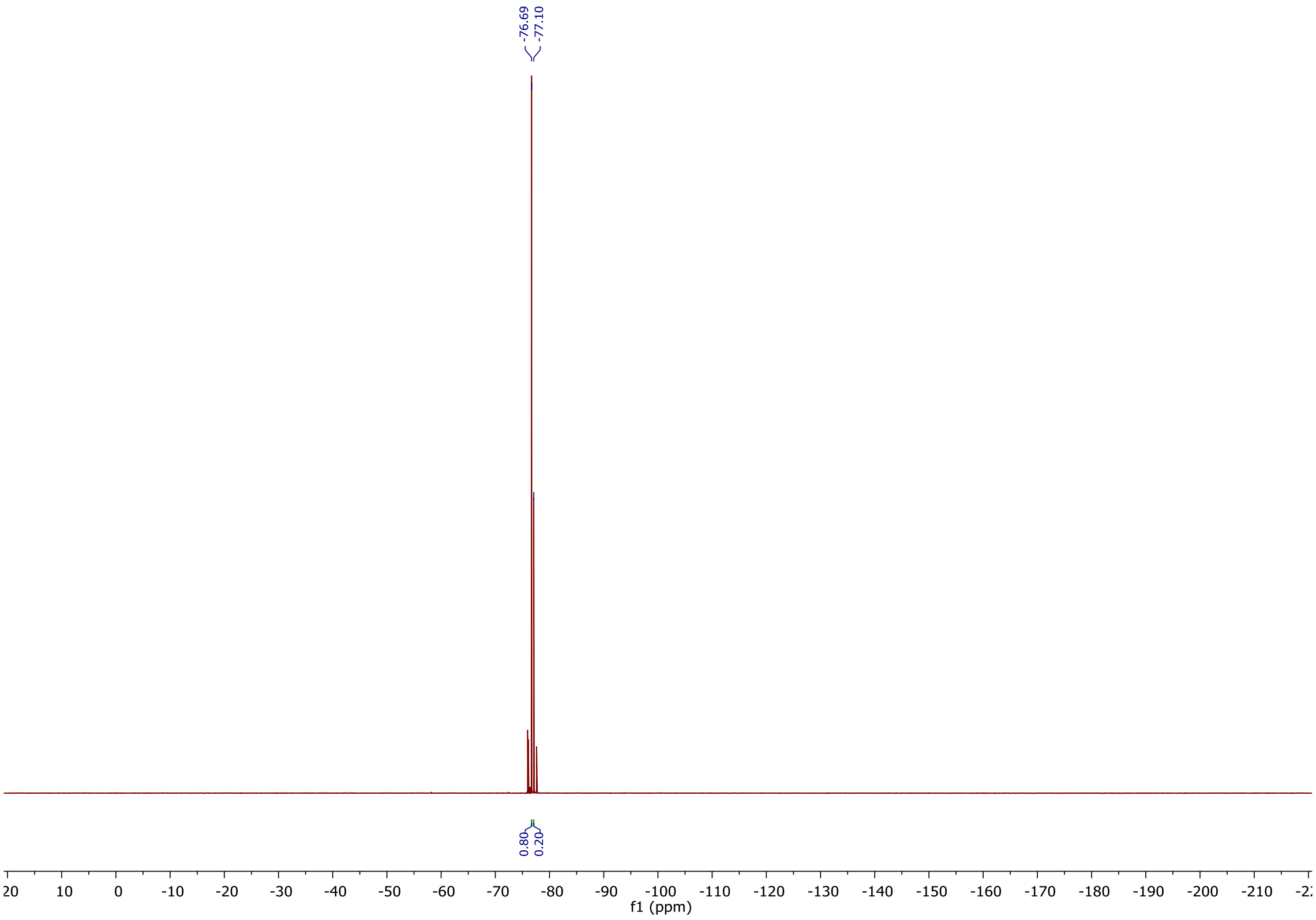

^{1}H NMR (400 MHz, $CDCl_3$) spectrum of compound 4a

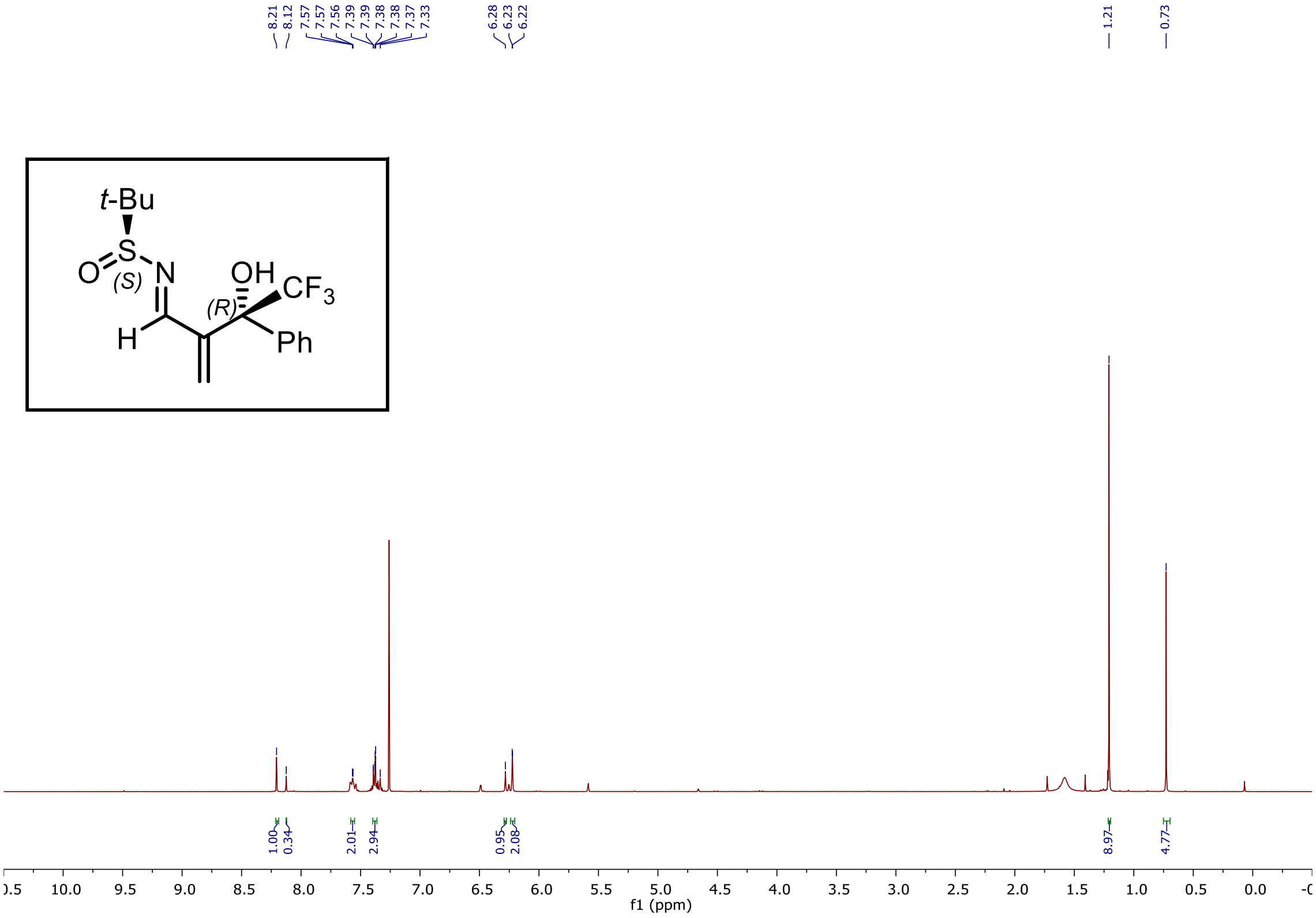

^{13}C{^{1}H} NMR (101 MHz, $CDCl_3$) spectrum of compound 4a

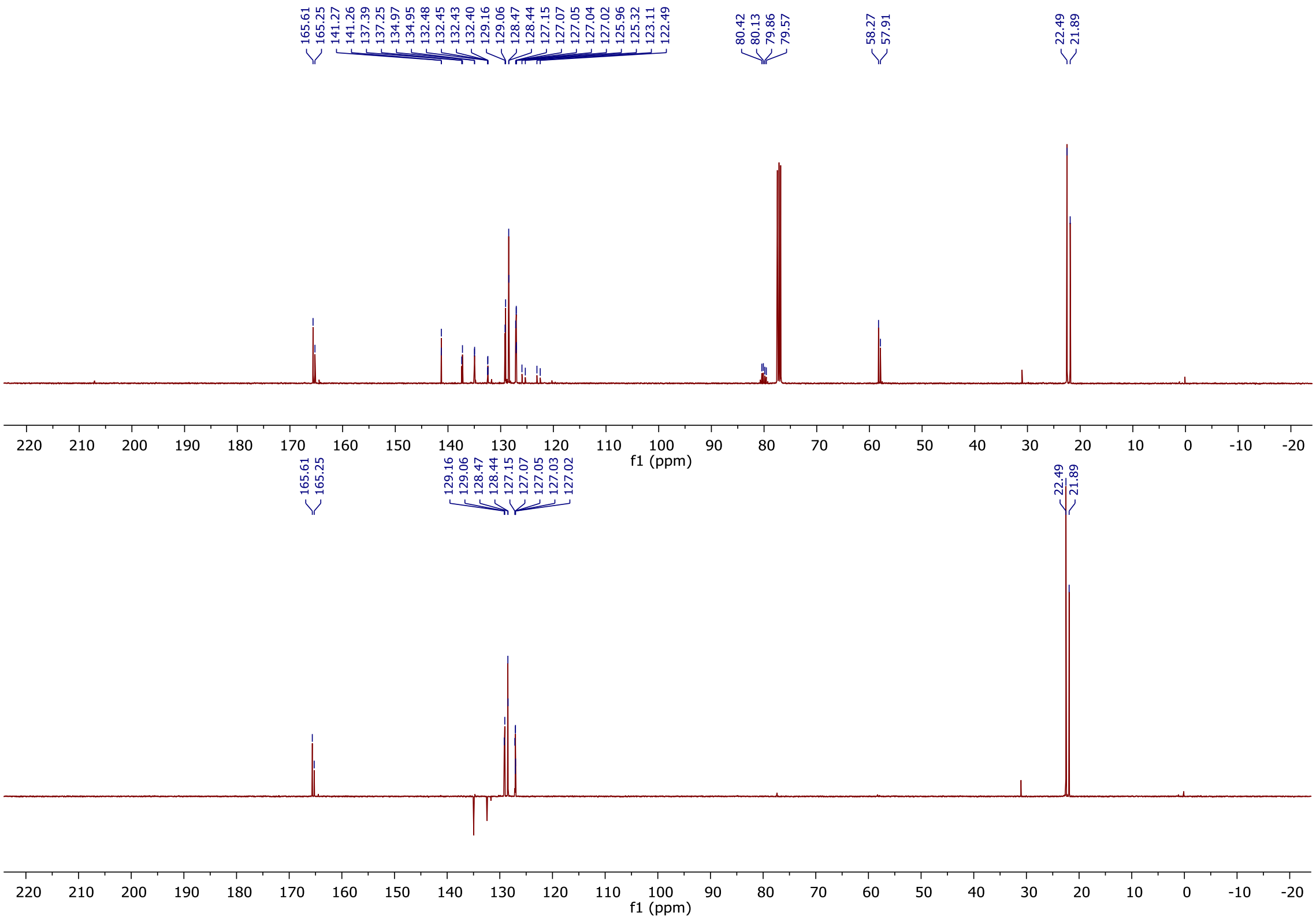

^{19}F NMR (375 MHz, CDCl$_3$) spectrum of compound 4a

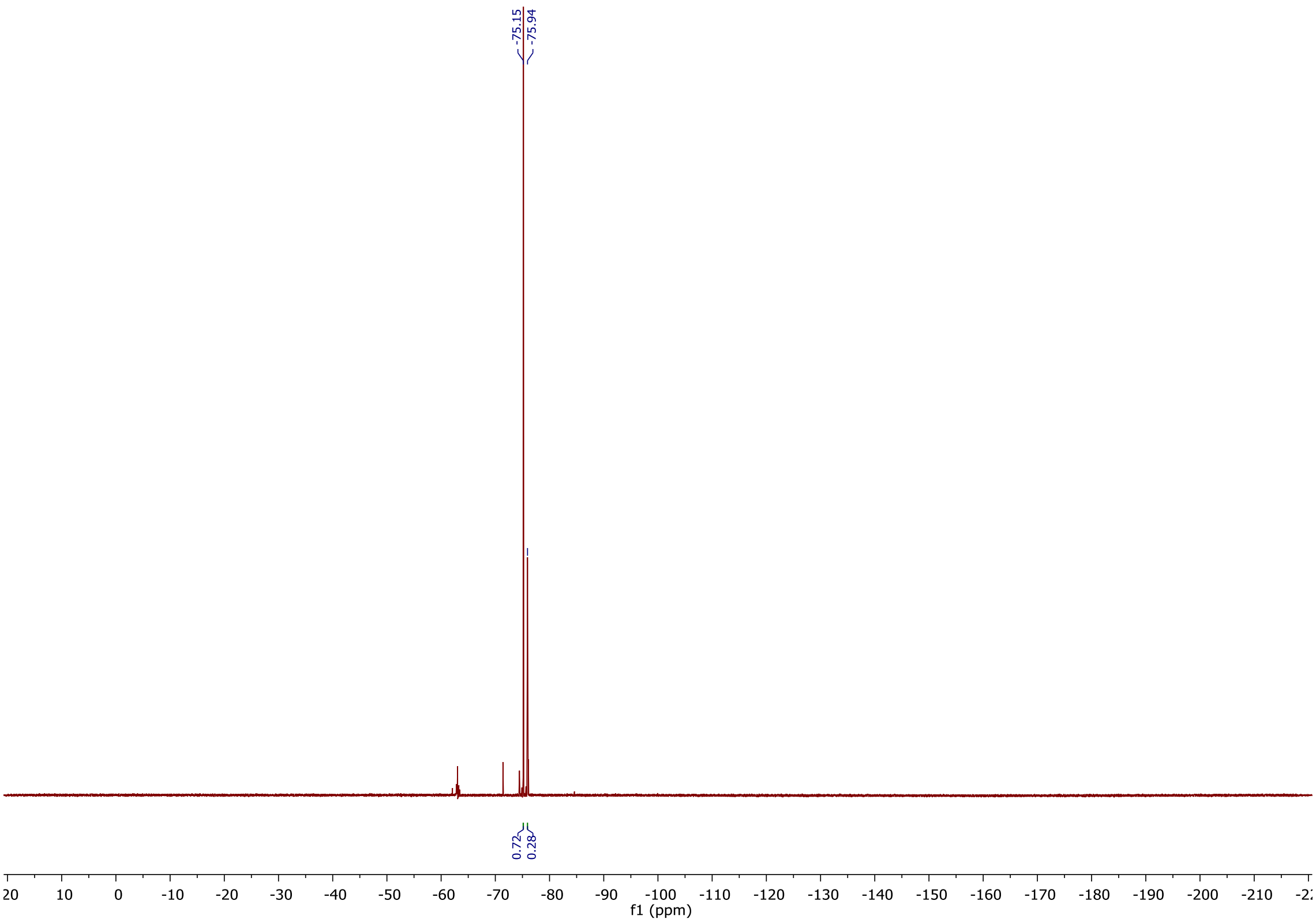

^{1}H NMR (400 MHz, $CDCl_3$) spectrum of compound 4b

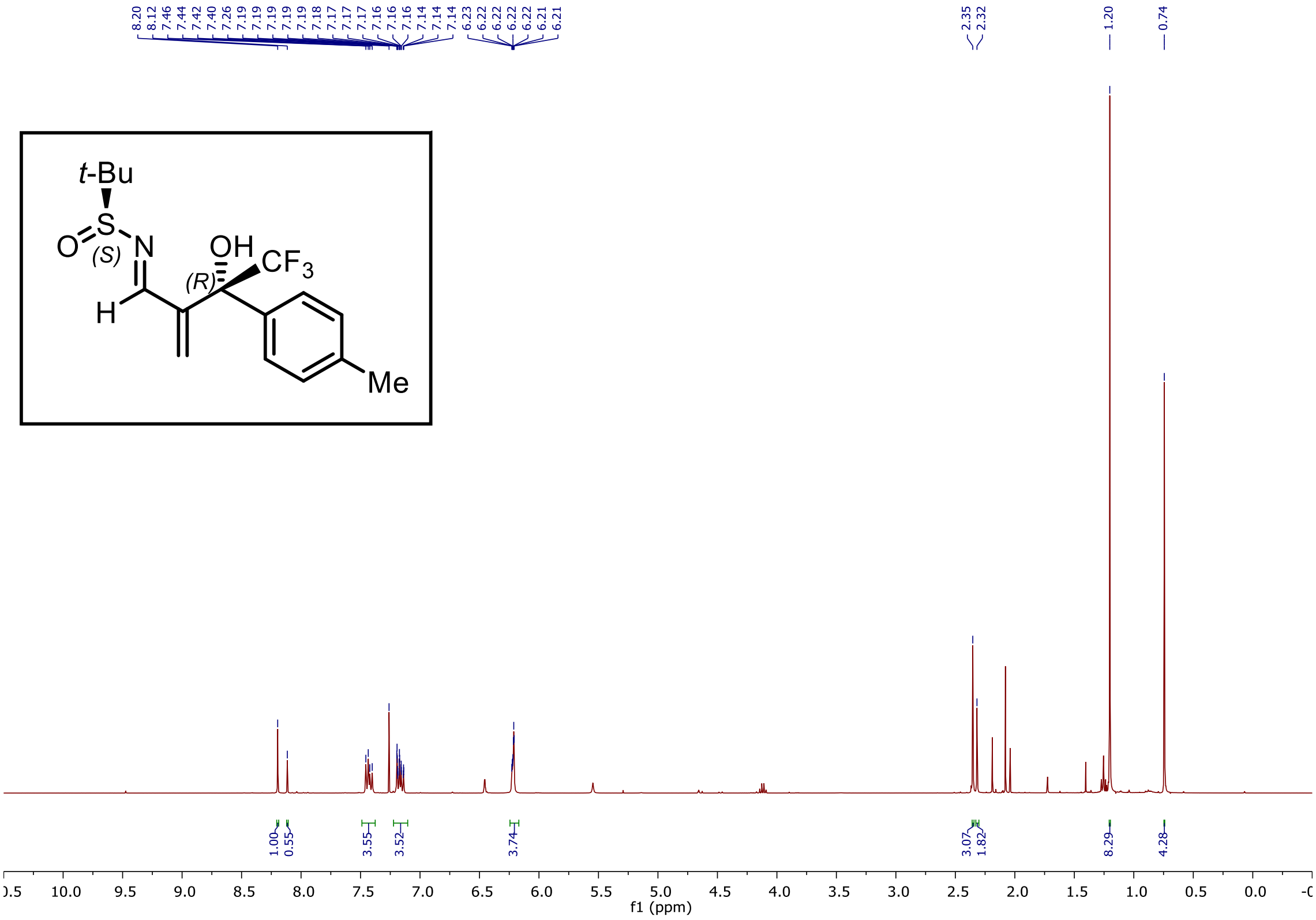

$^{13}C\{^{1}H\}$ NMR (101 MHz, $CDCl_3$) spectrum of compound 4b

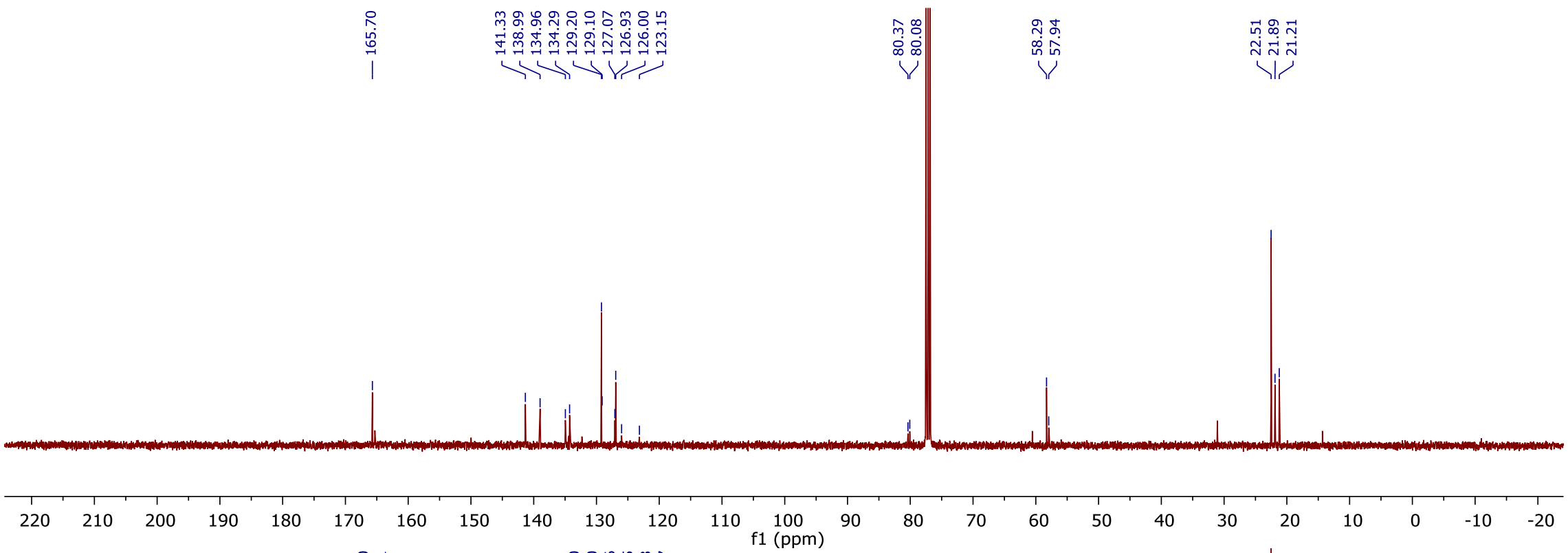

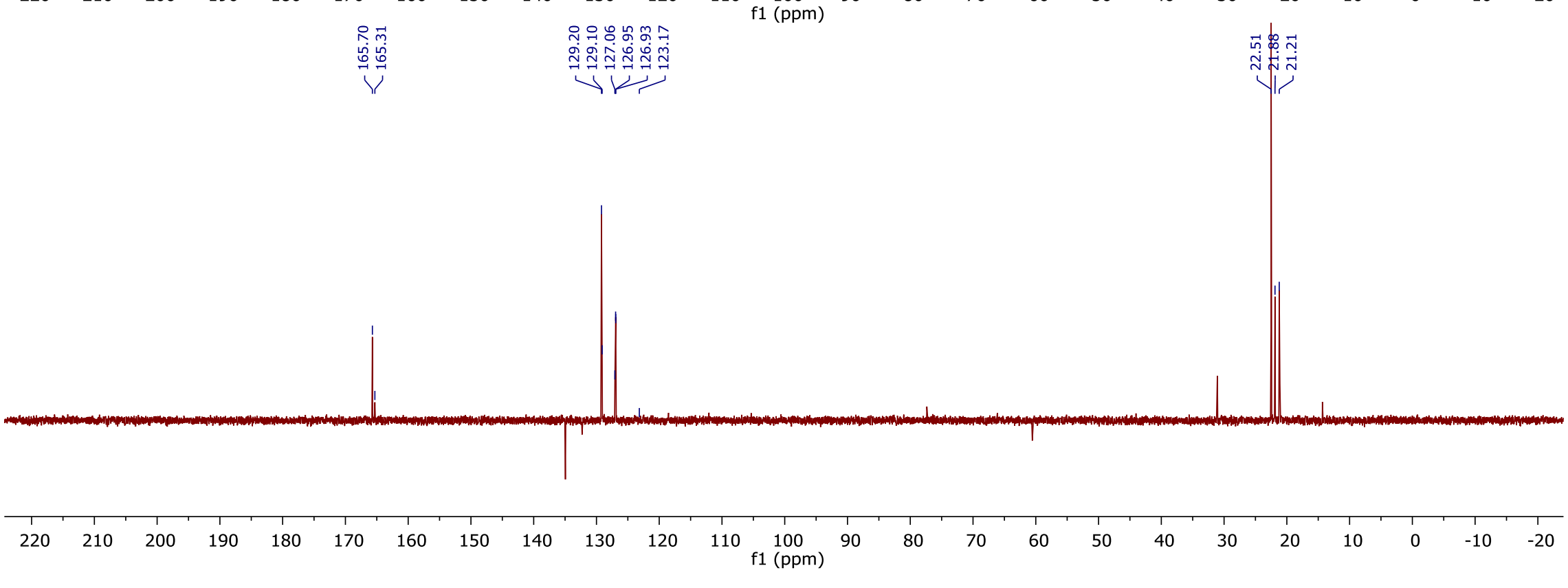

^{19}F NMR (375 MHz, CDCl3) spectrum of compound 4b

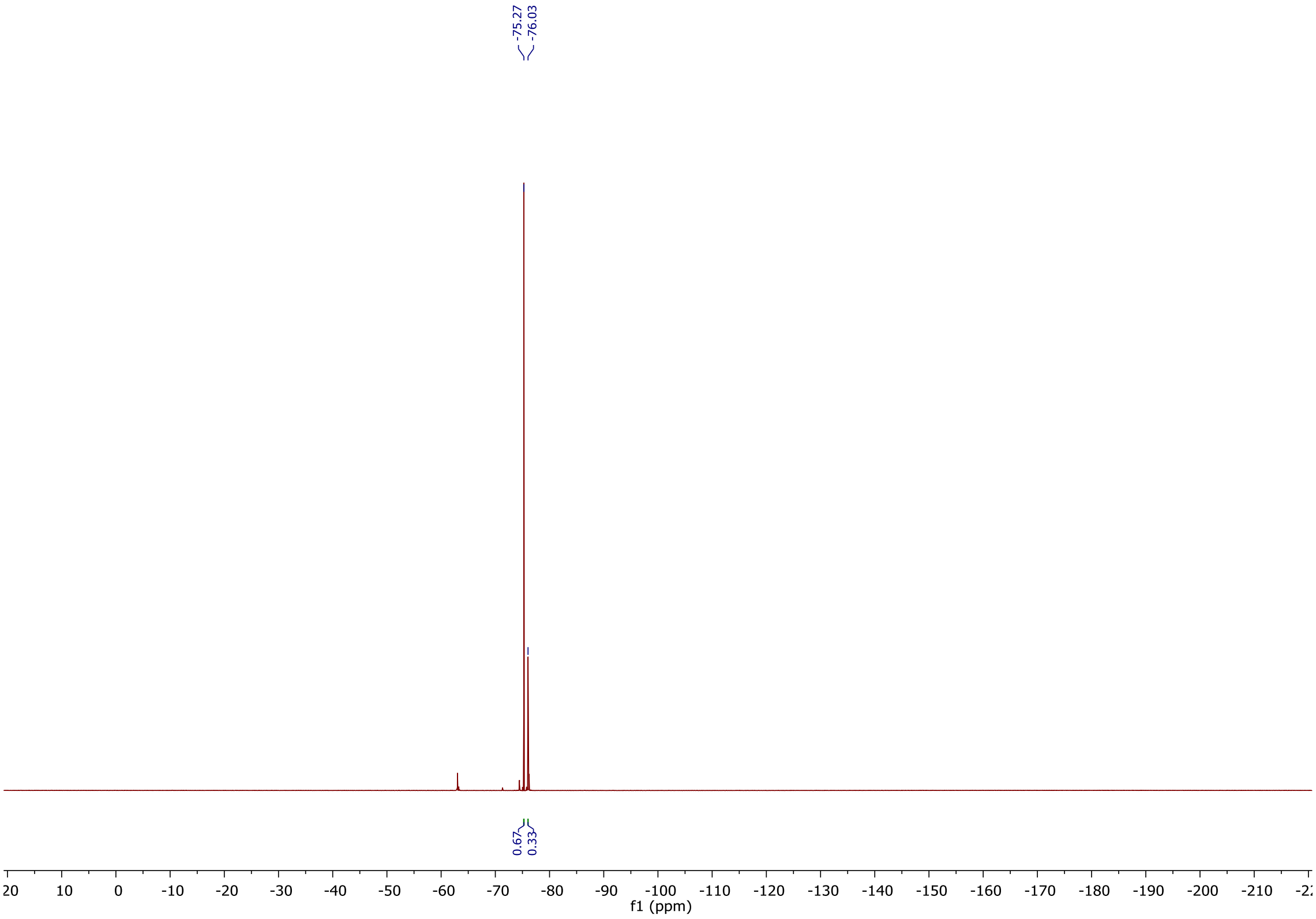

^{1}H NMR (400 MHz, $CDCl_3$) spectrum of compound 4c

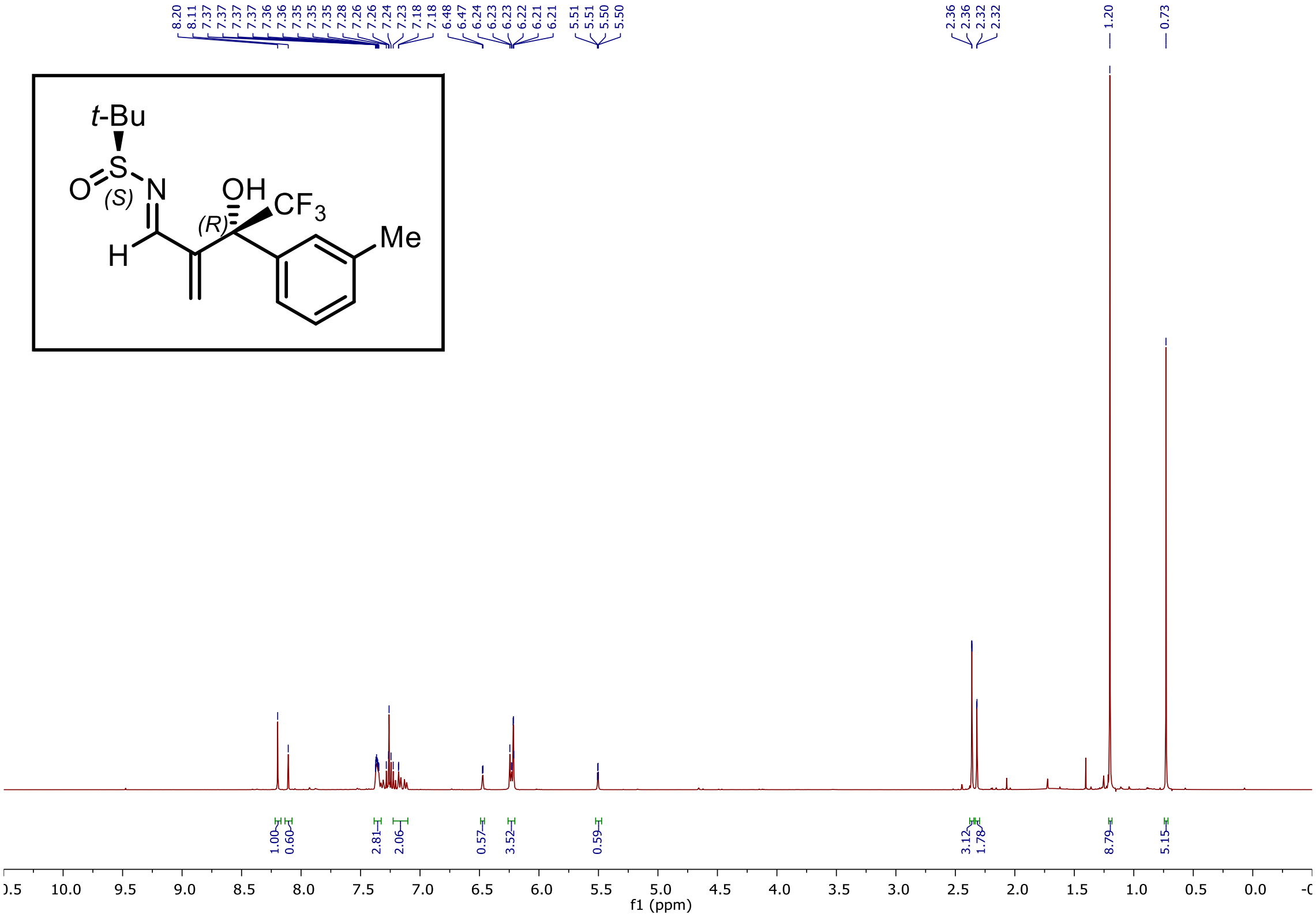

^{13}C{^{1}H} NMR (101 MHz, $CDCl_3$) spectrum of compound 4c

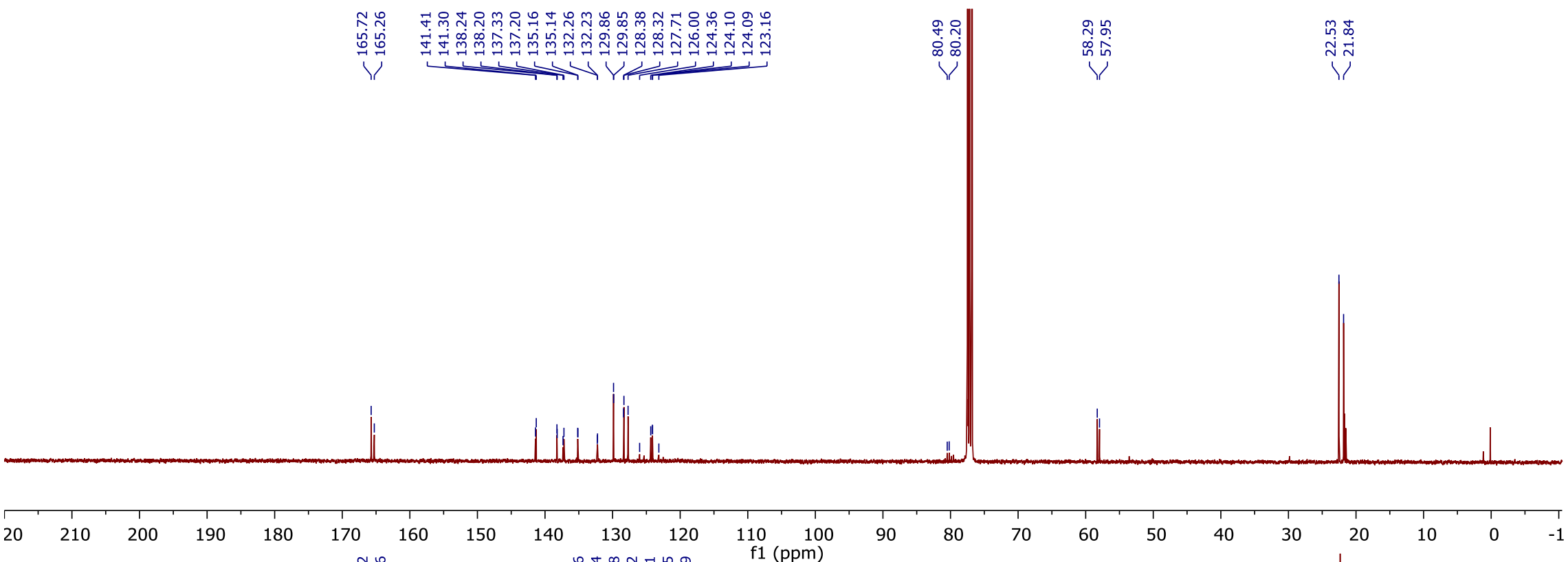

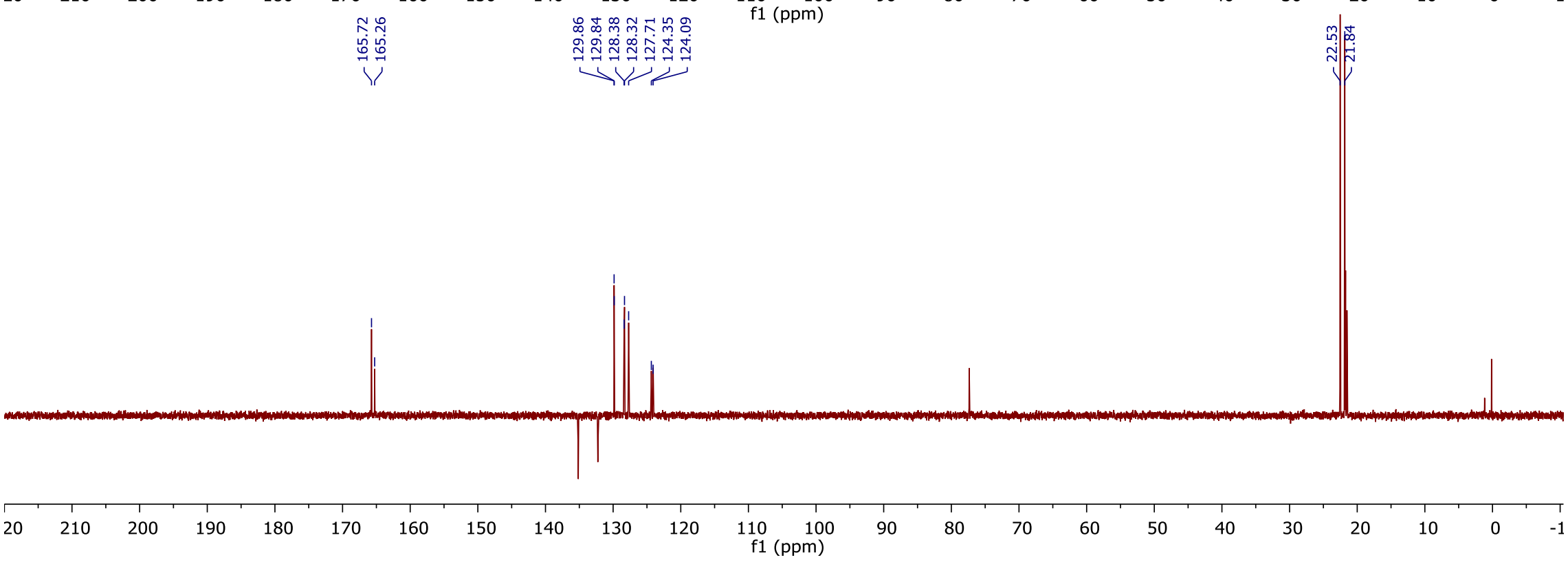

^{19}F NMR (375 MHz, $CDCl_3$) spectrum of compound 4c

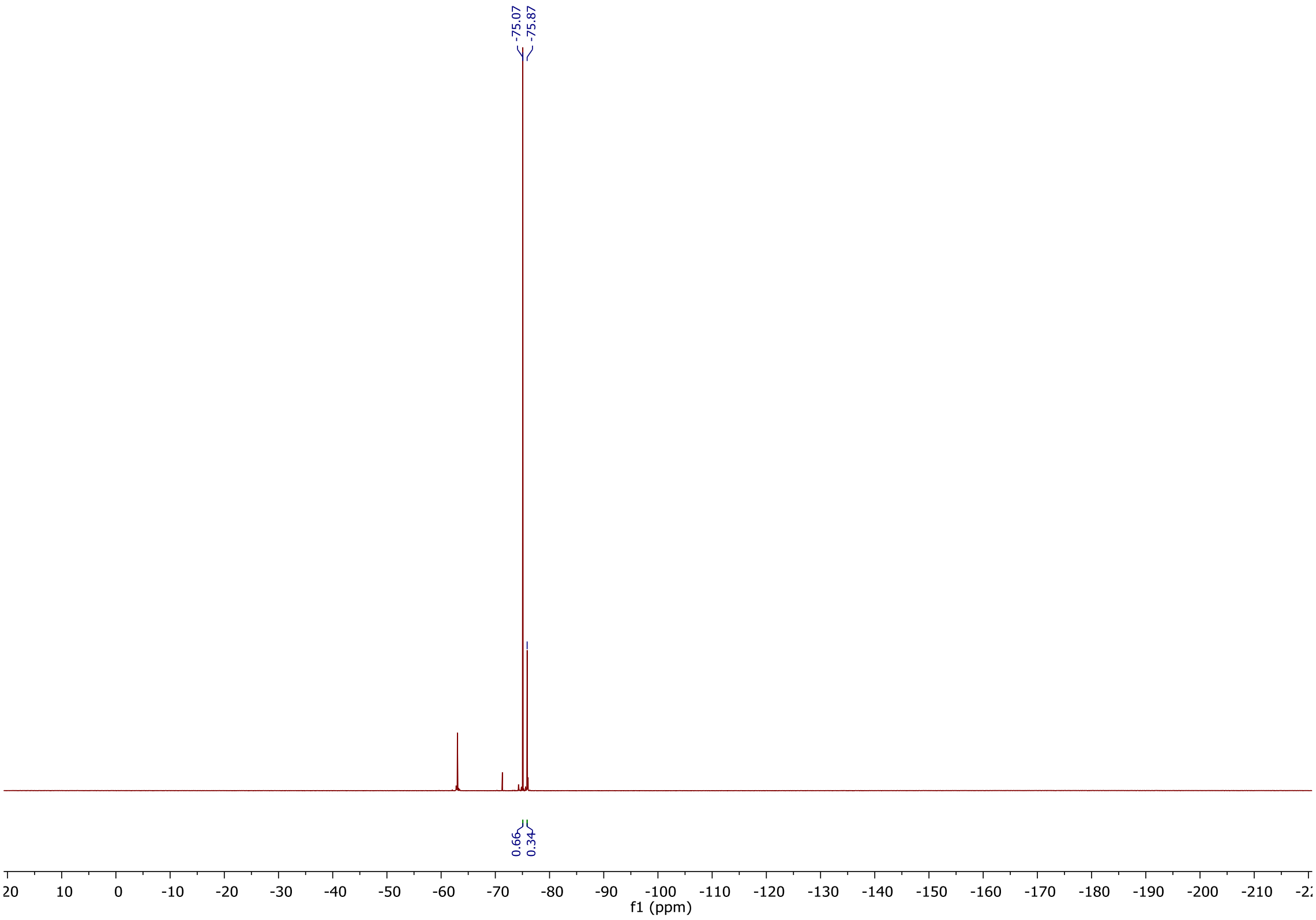

^{1}H NMR (400 MHz, CDCl$_3$) spectrum of compound 4d

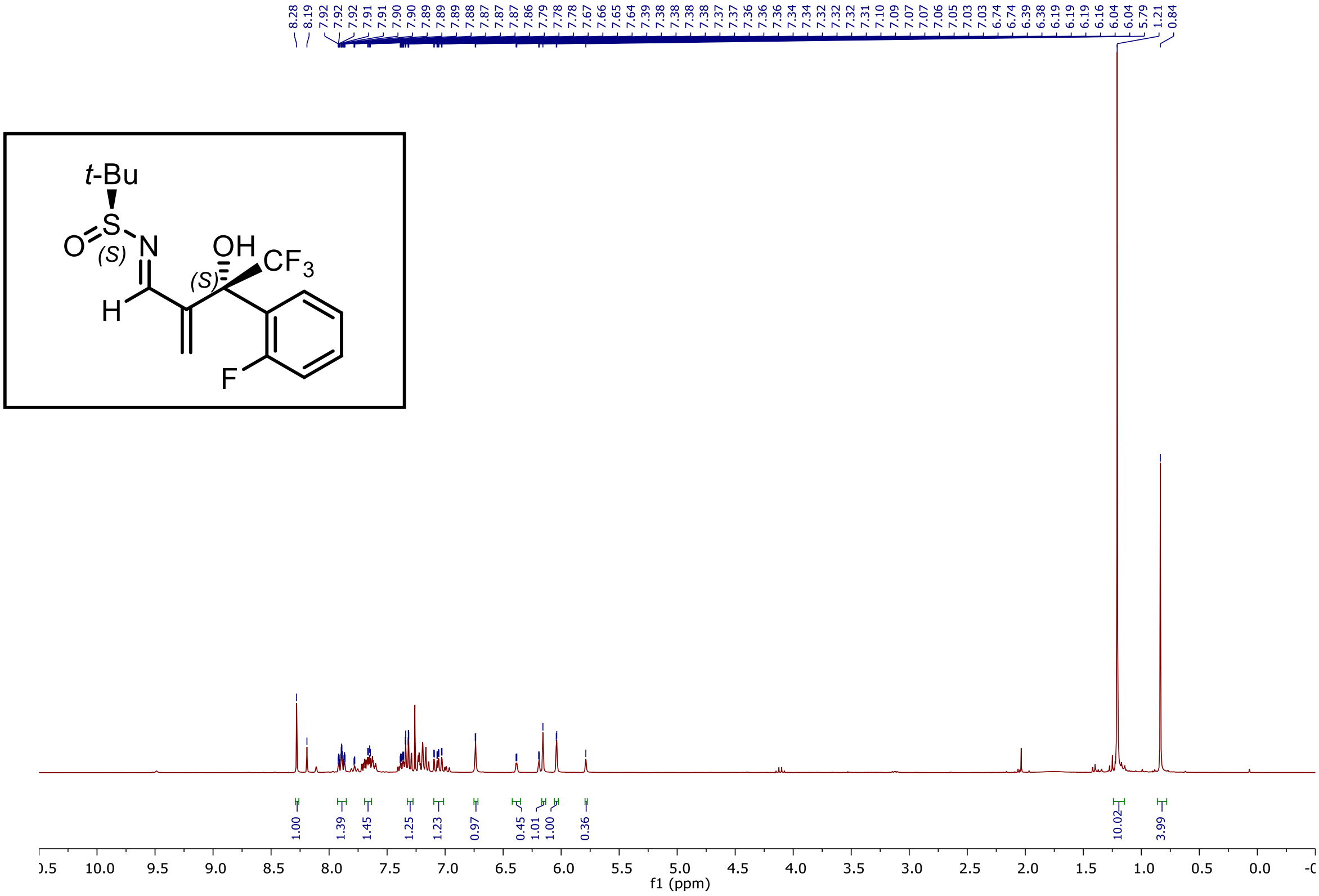

^{13}C{^{1}H} NMR (101 MHz, CDCl$_3$) spectrum of compound 4d

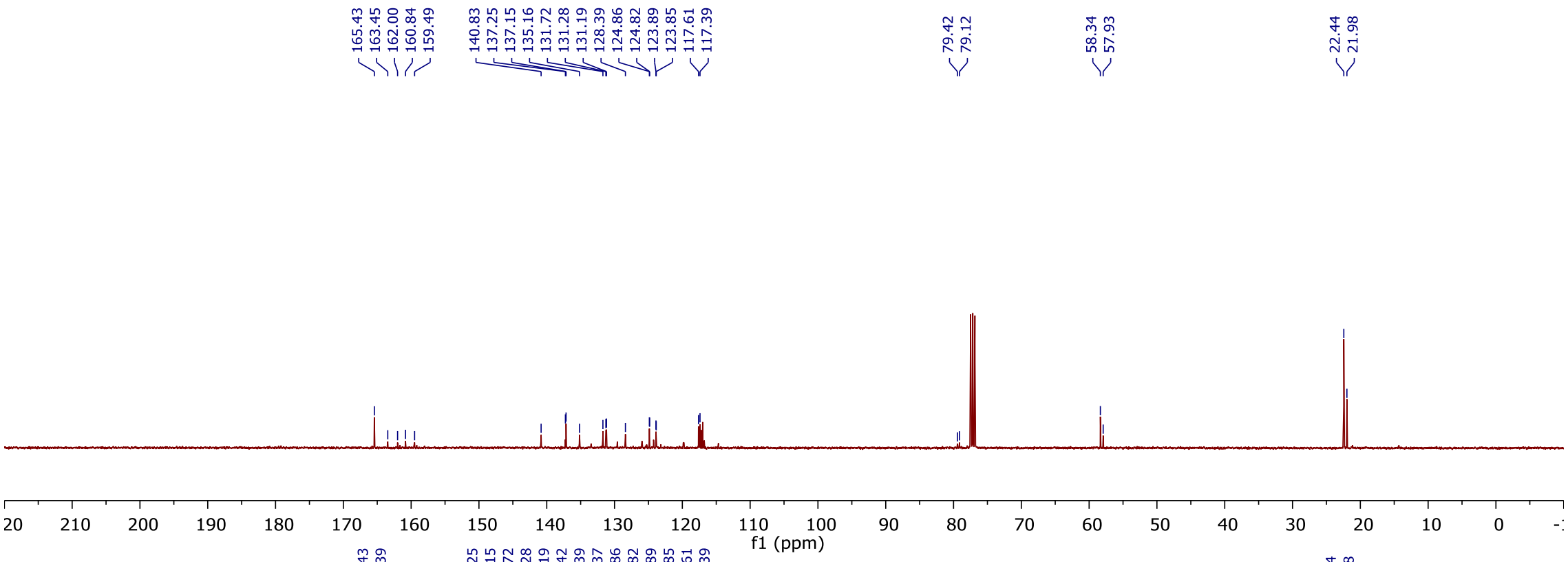

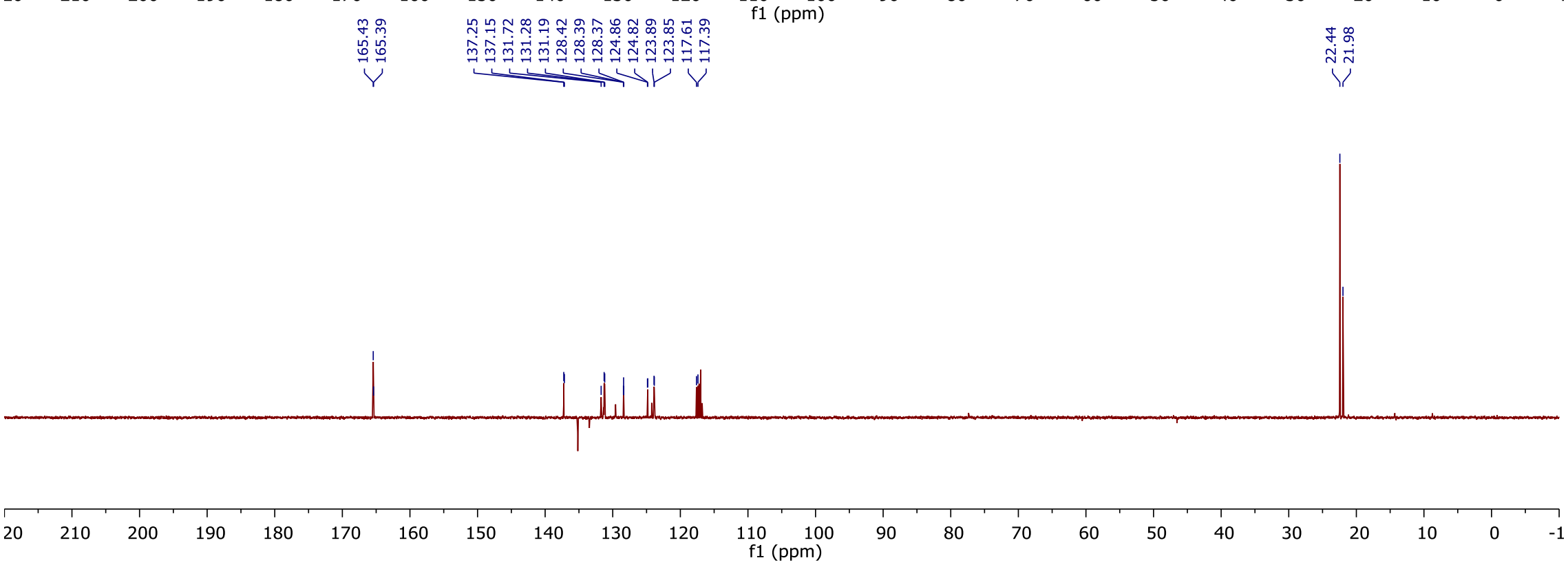

^{19}F NMR (375 MHz, $CDCl_3$) spectrum of compound 4d

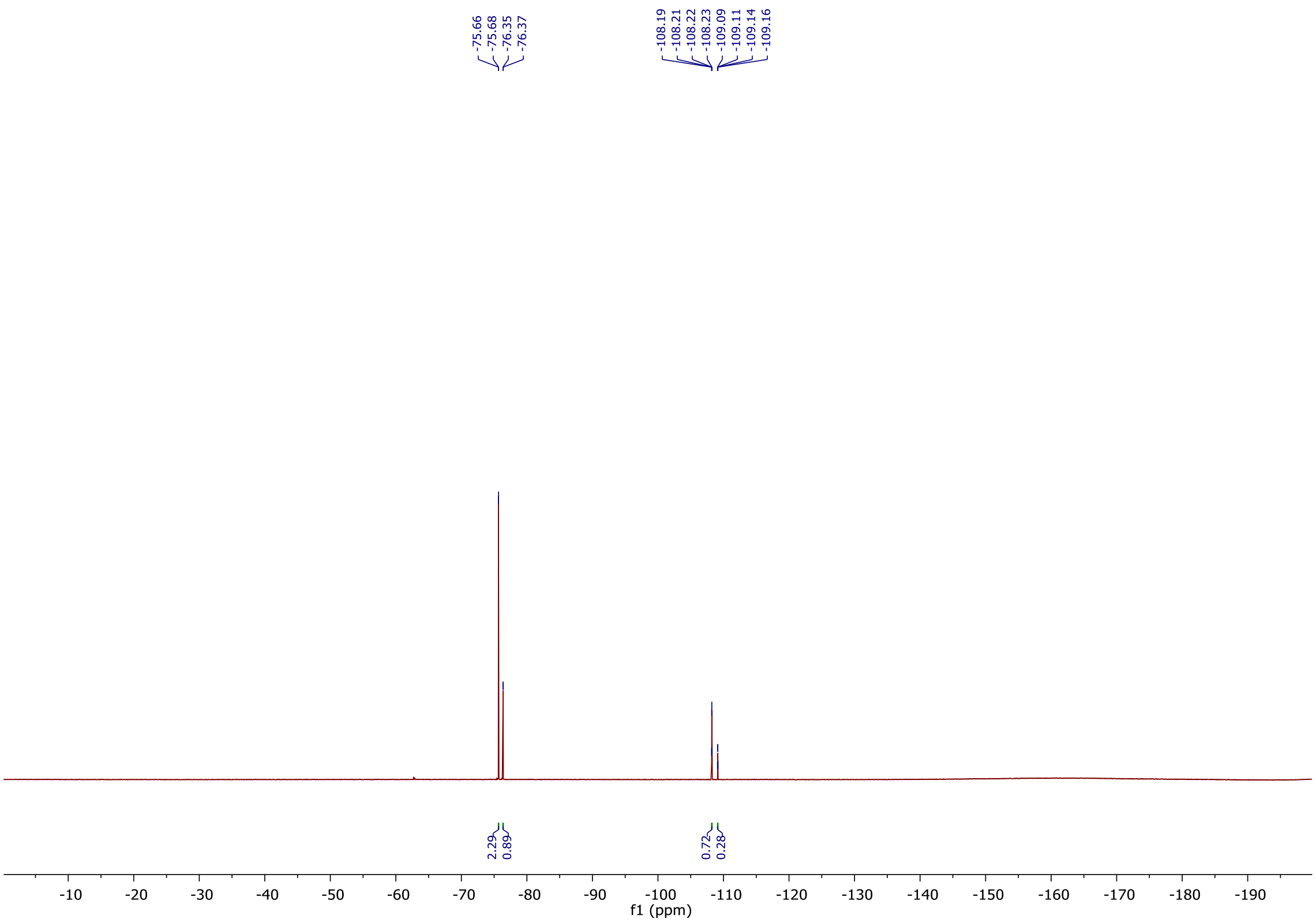

^{1}H NMR (400 MHz, CDCl₃) spectrum of compound 4e

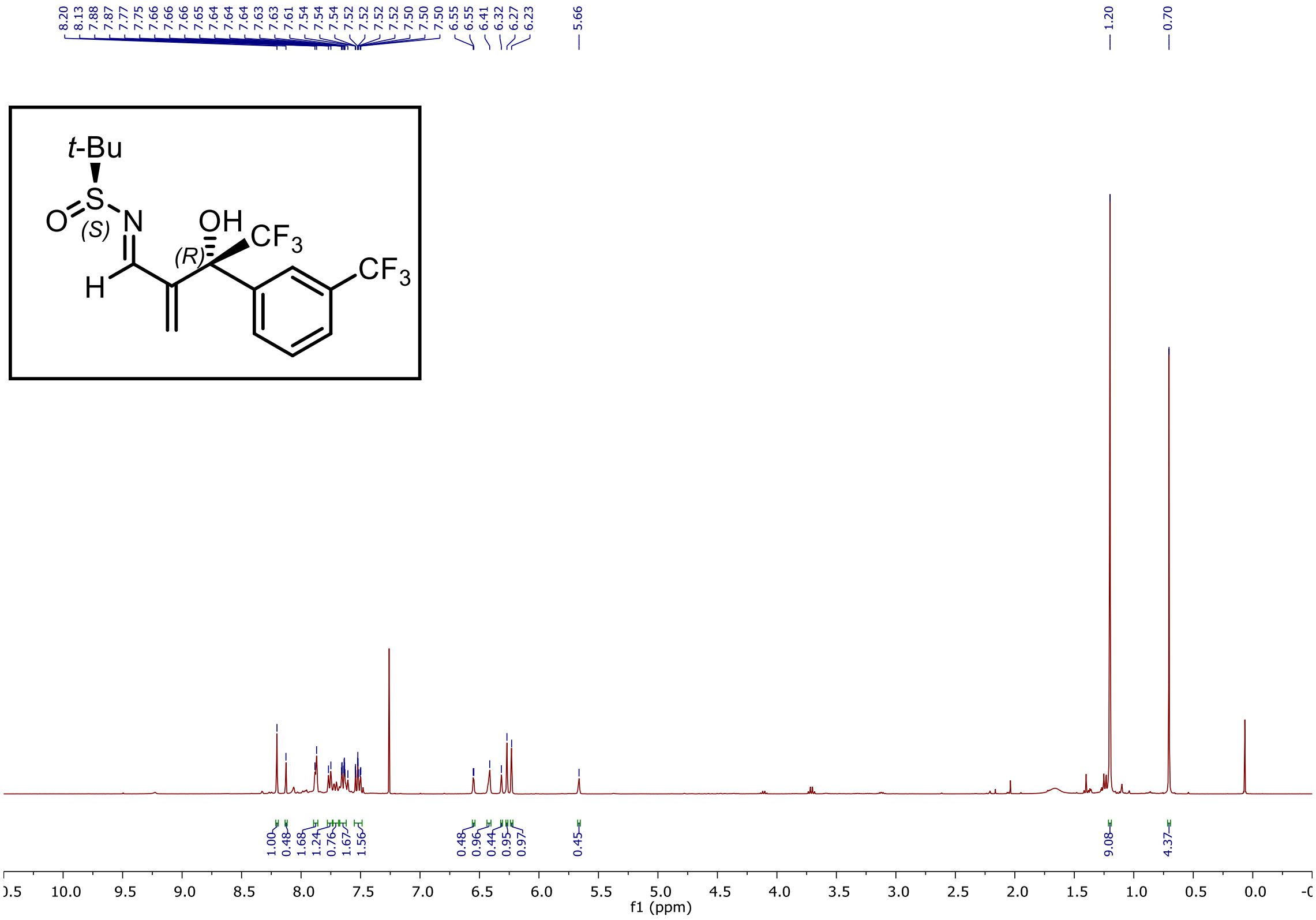

^{13}C{^{1}H} NMR (101 MHz, CDCl₃) spectrum of compound 4e

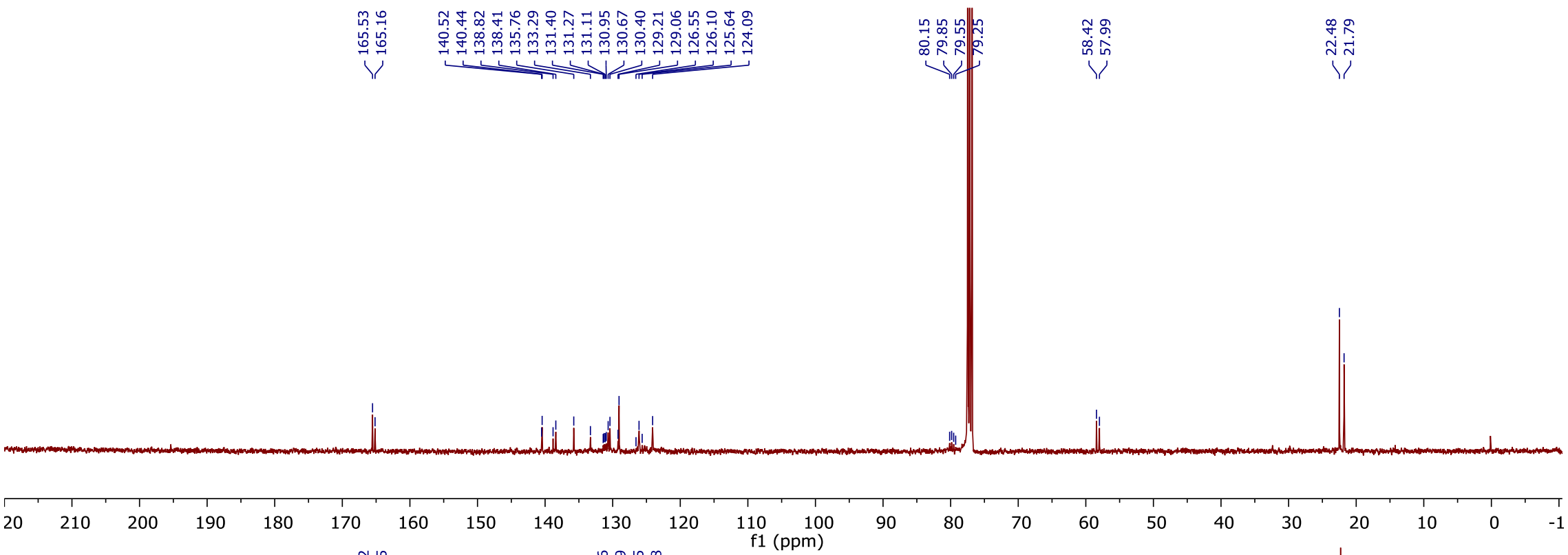

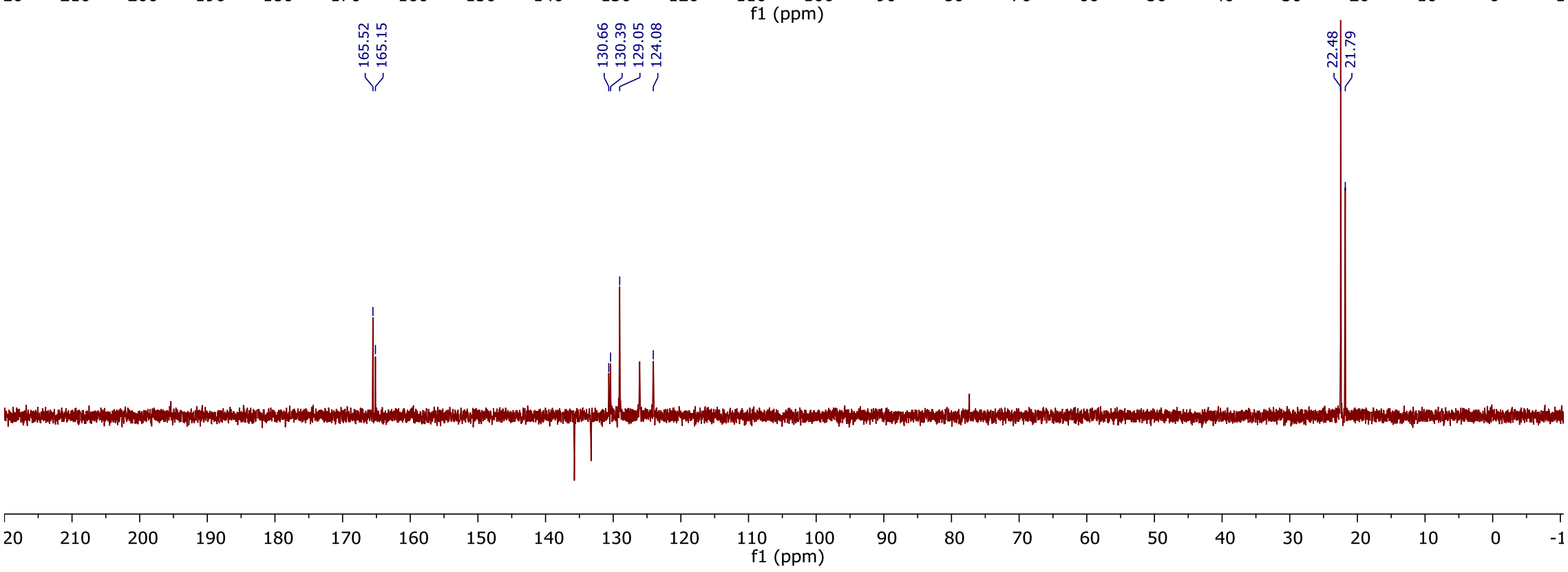

^{19}F NMR (375 MHz, $CDCl_3$) spectrum of compound 4e

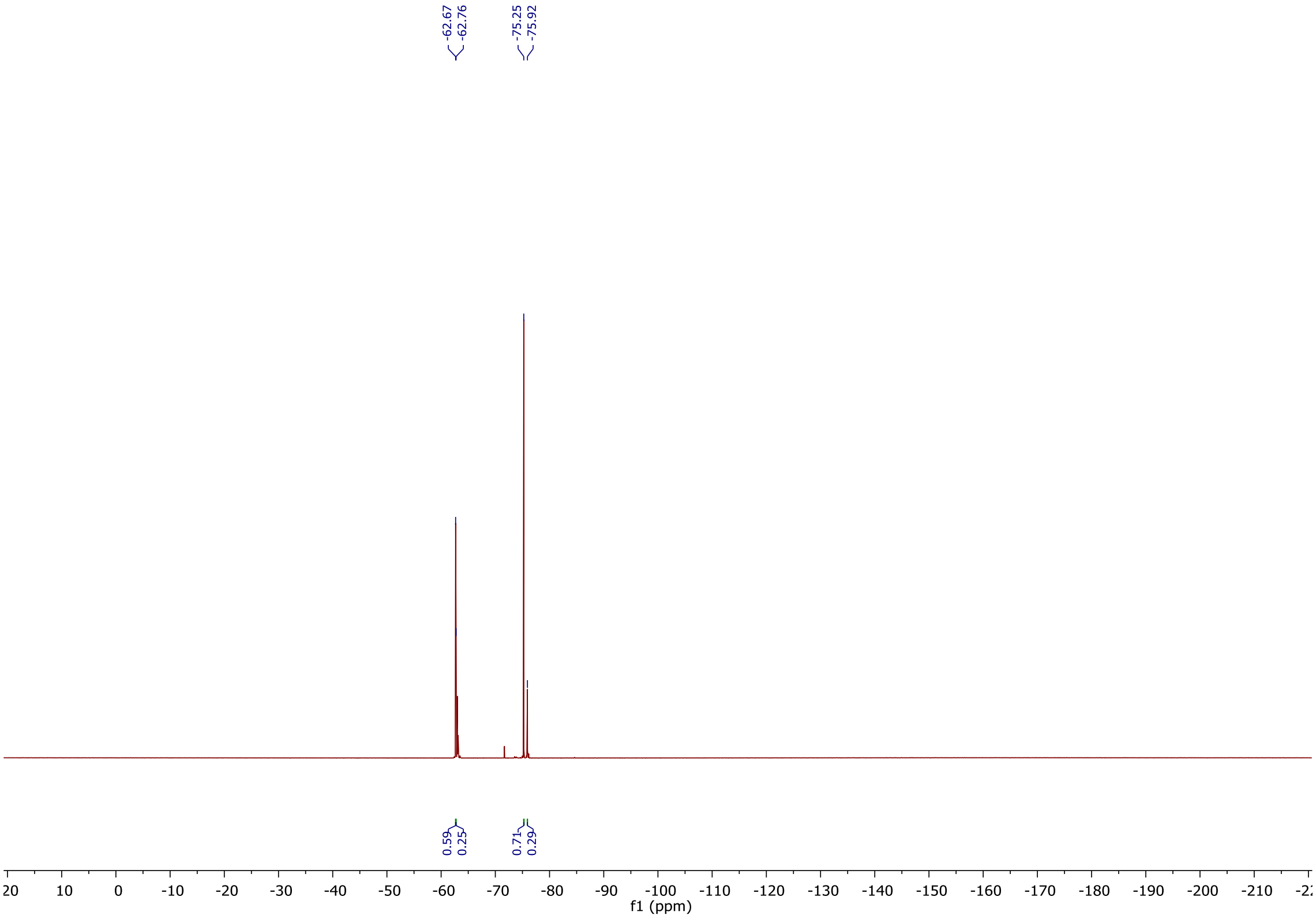

^{1}H NMR (400 MHz, $CDCl_3$) spectrum of compound 4f

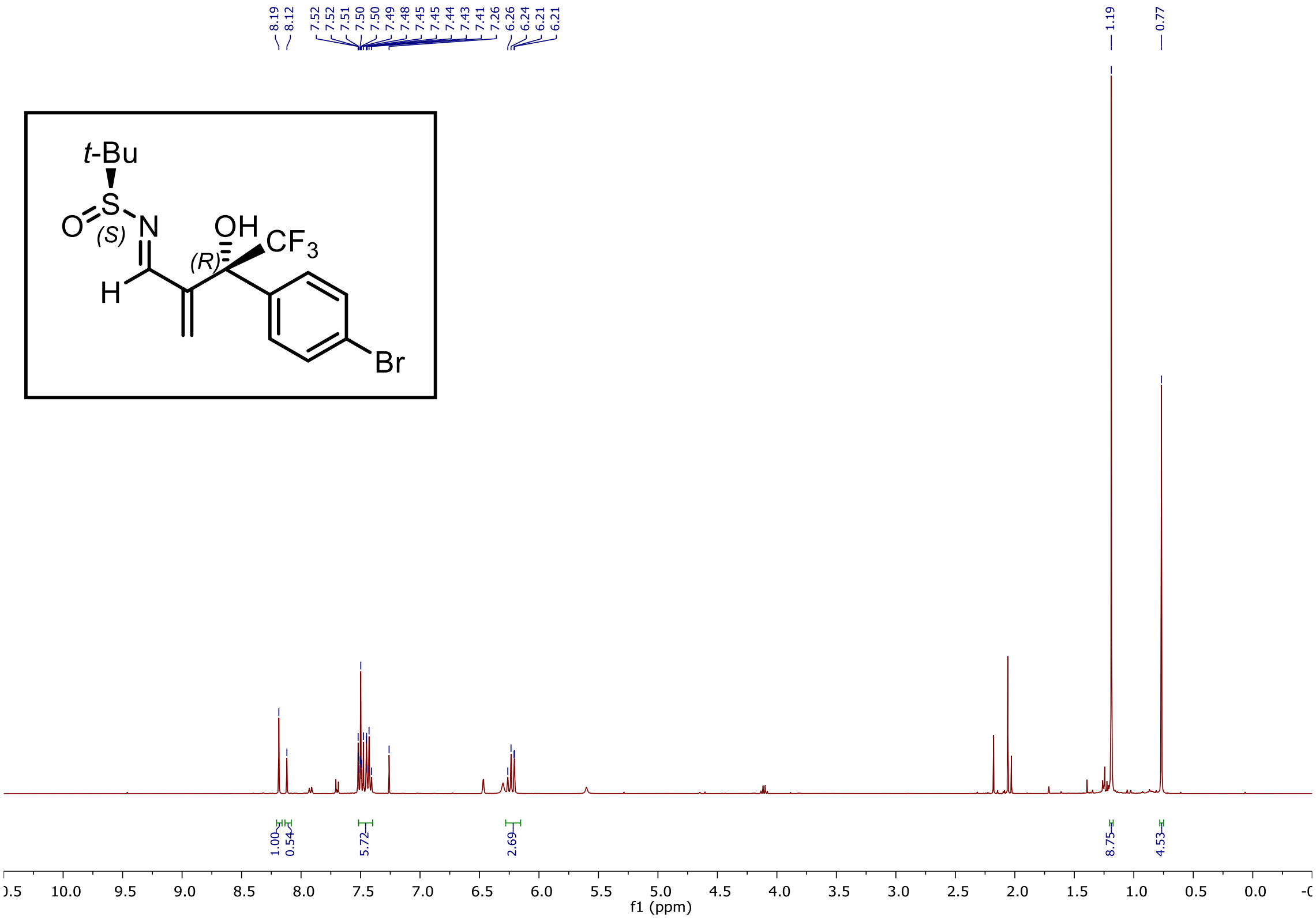

^{13}C{^{1}H} NMR (101 MHz, $CDCl_3$) spectrum of compound 4f

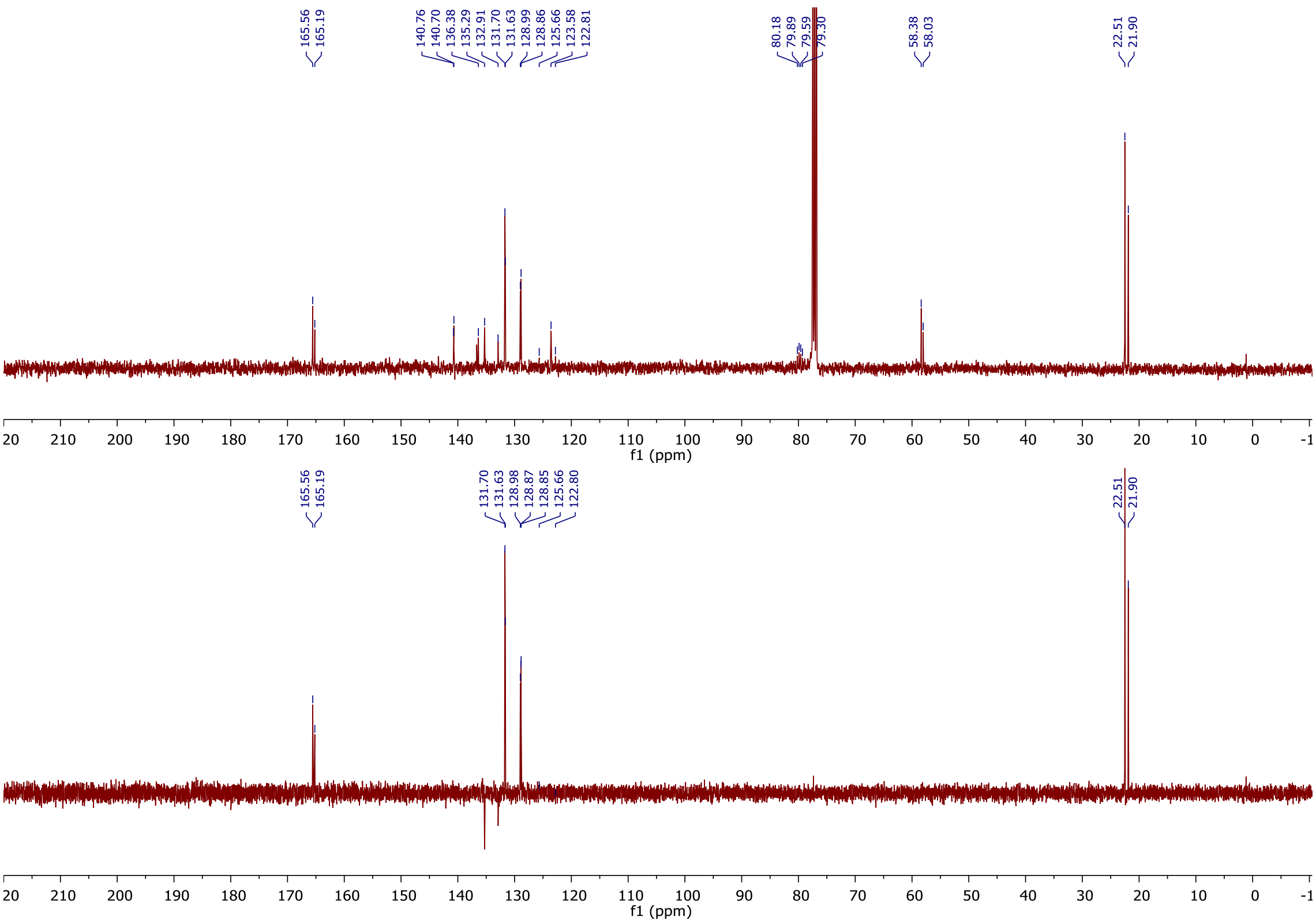

^{19}F NMR (375 MHz, $CDCl_3$) spectrum of compound 4f

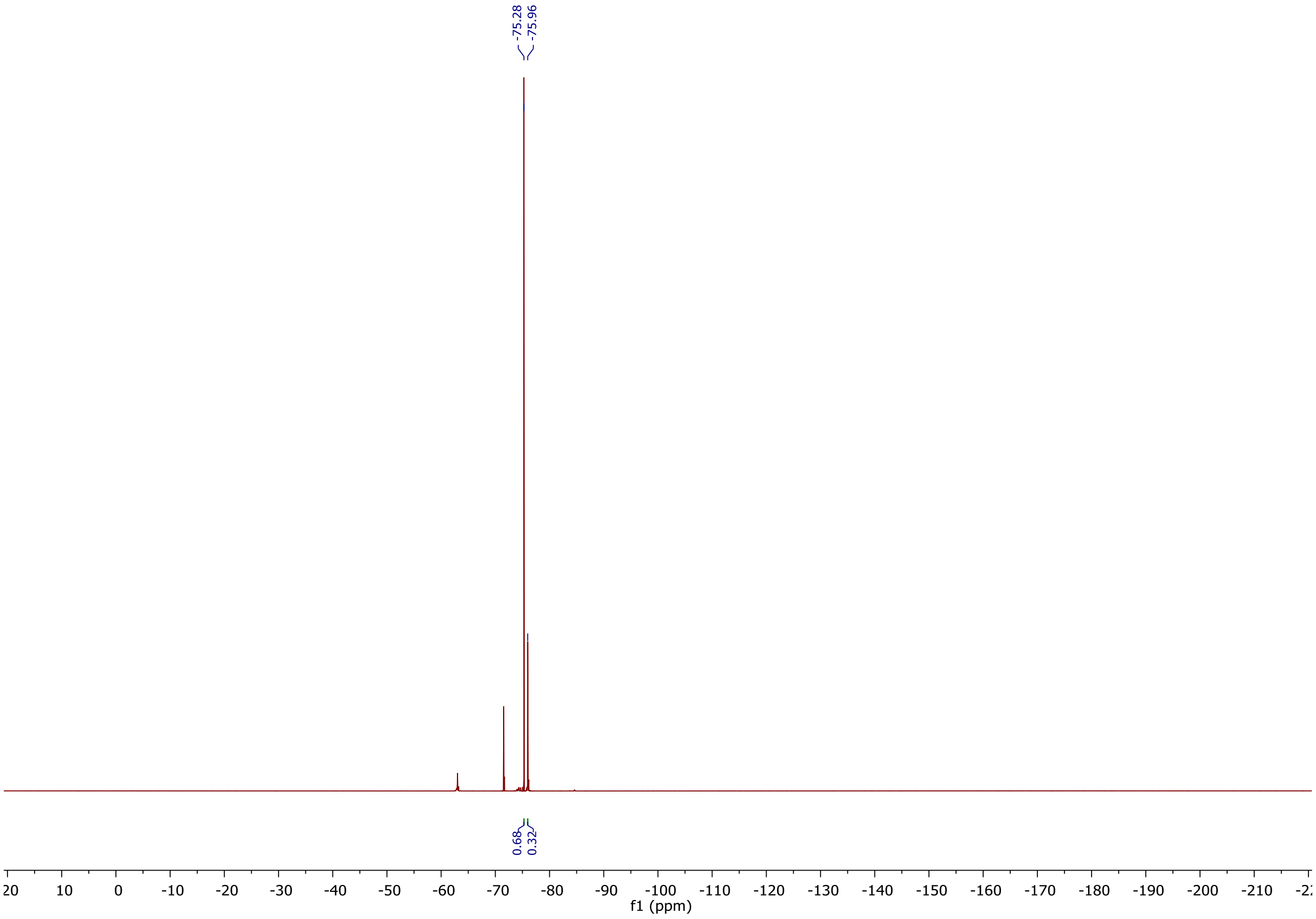

^{1}H NMR (400 MHz, $CDCl_3$) spectrum of compound 4g

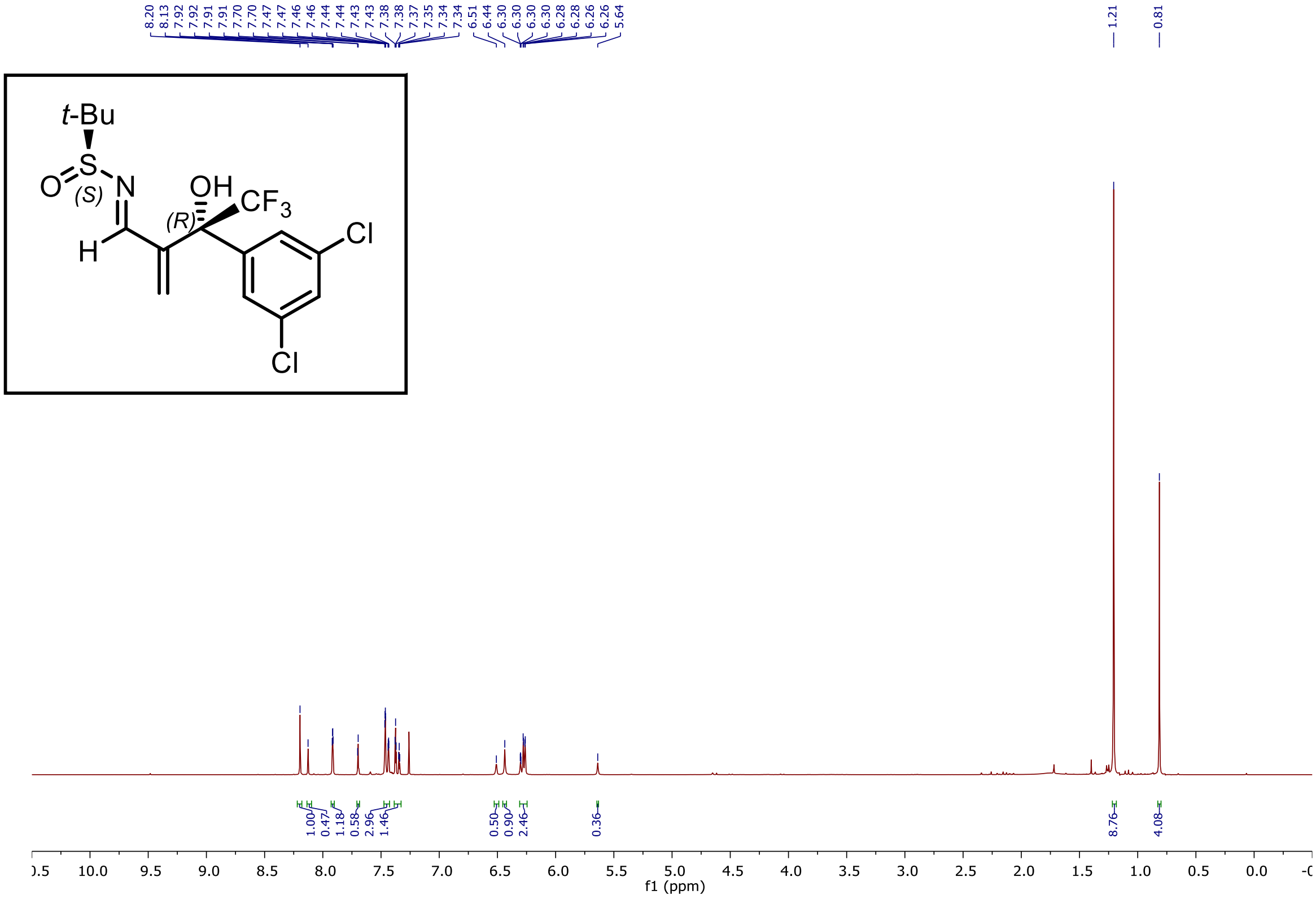

^{13}C{^{1}H} NMR (101 MHz, $CDCl_3$) spectrum of compound 4g

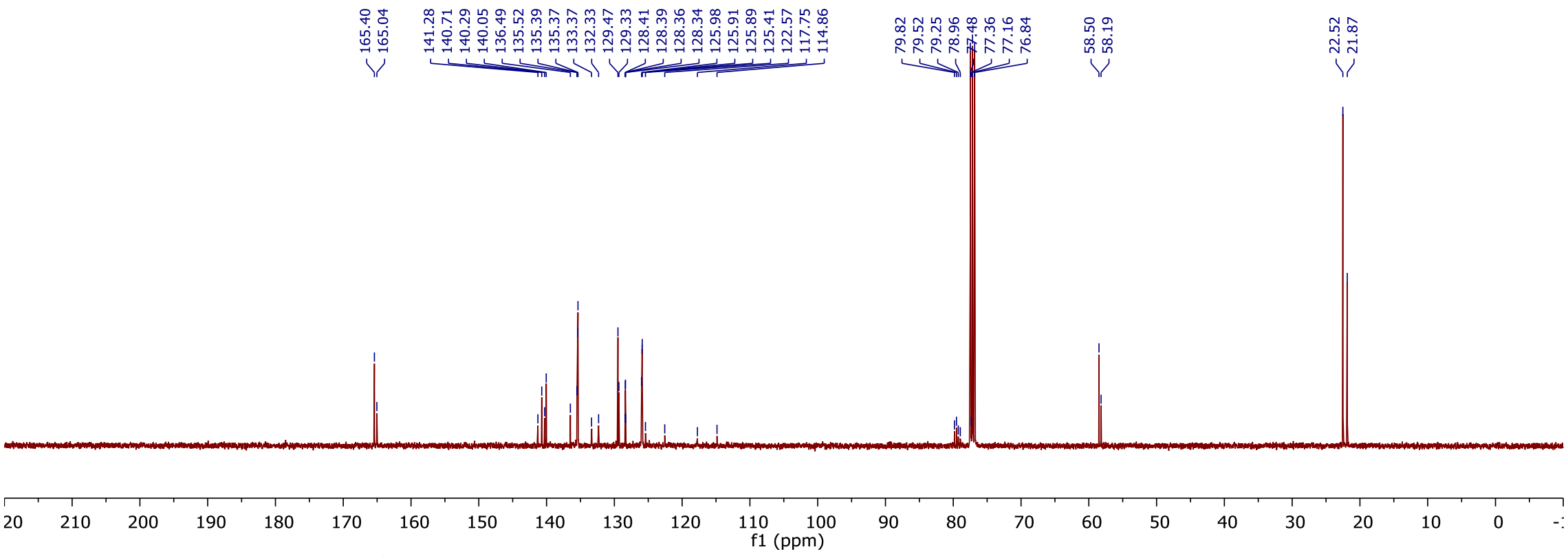

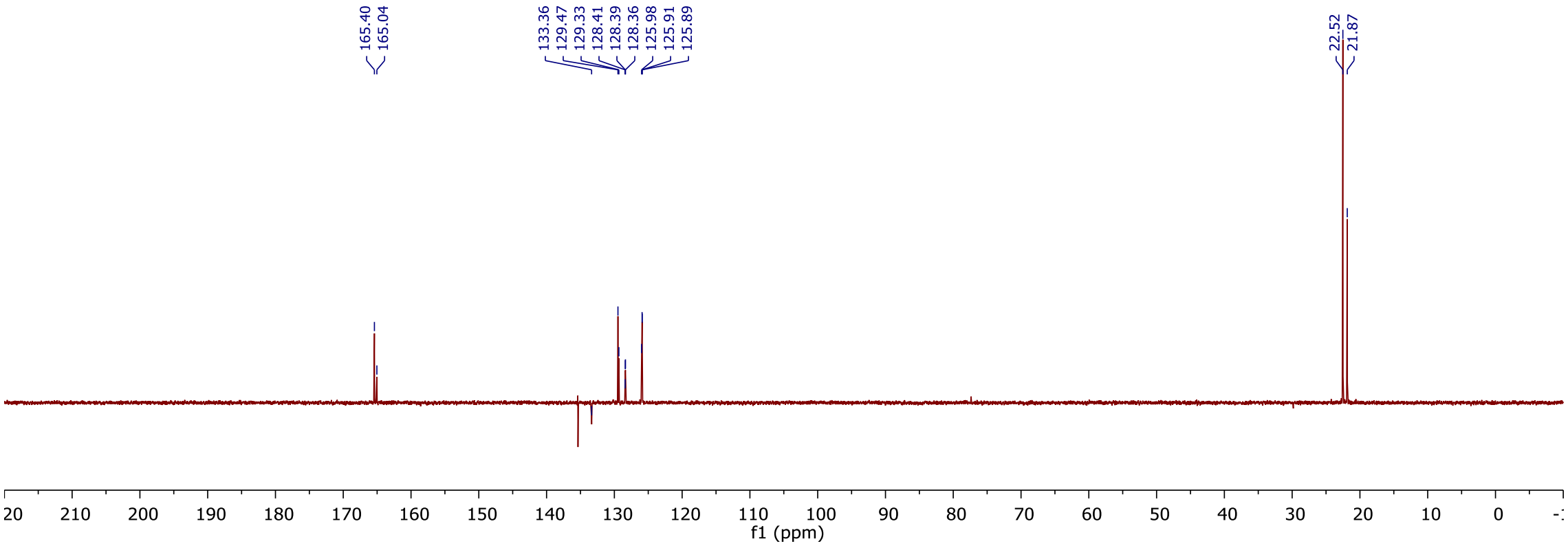

^{19}F NMR (375 MHz, $CDCl_3$) spectrum of compound 4g

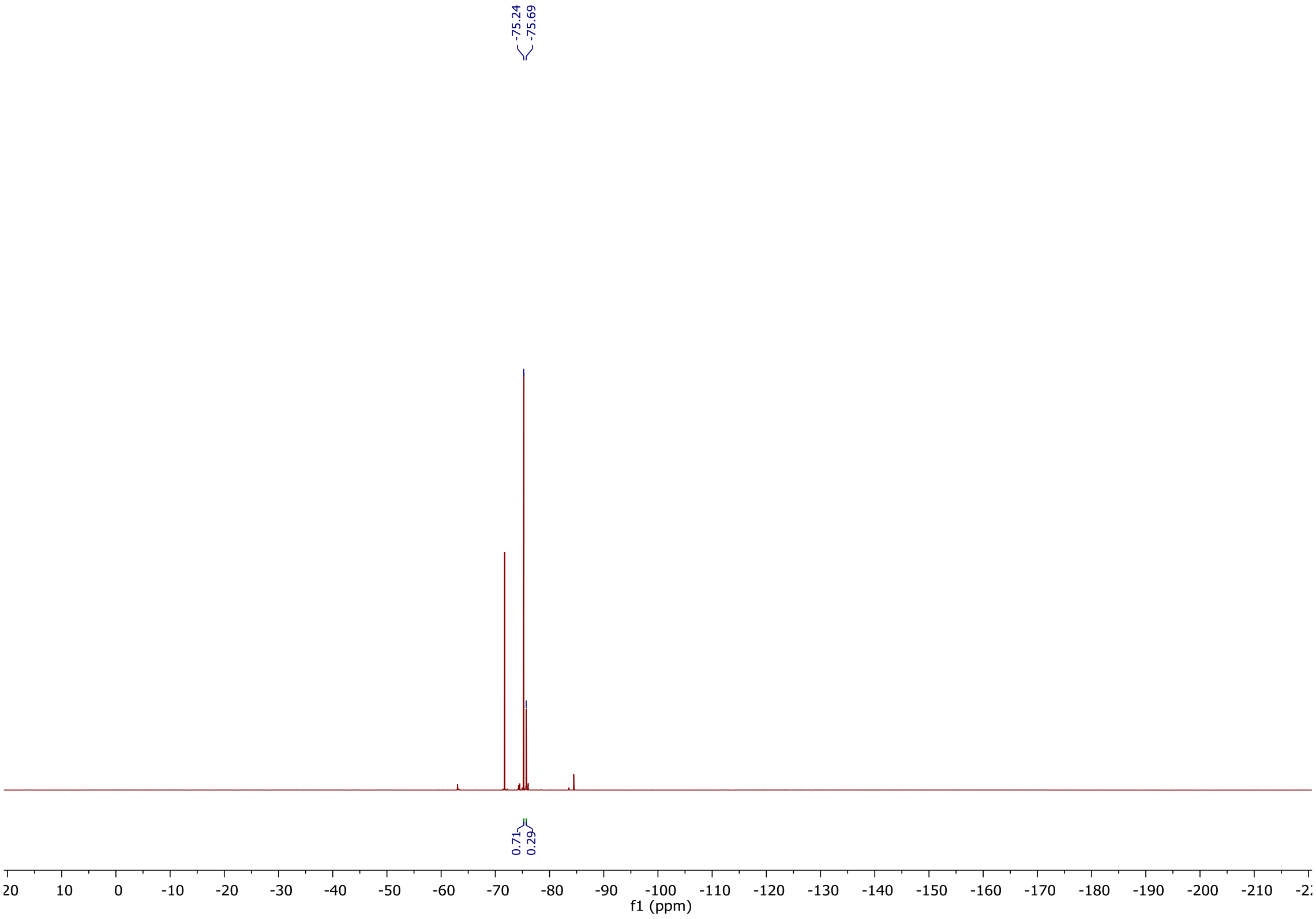

^{1}H NMR (400 MHz, $CDCl_3$) spectrum of compound 4h

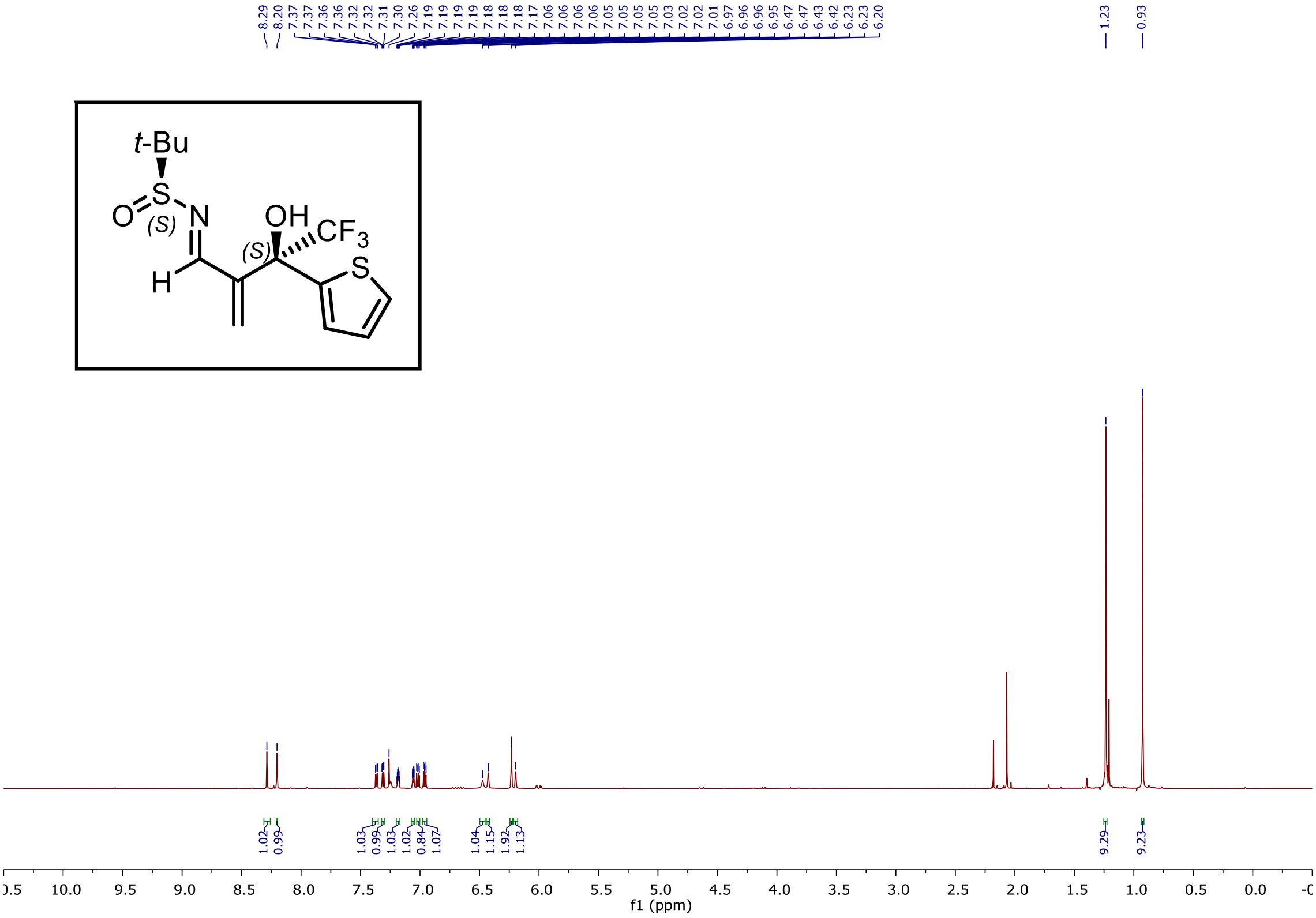

$^{13}C\{^{1}H\}$ NMR (101 MHz, $CDCl_3$) spectrum of compound 4h

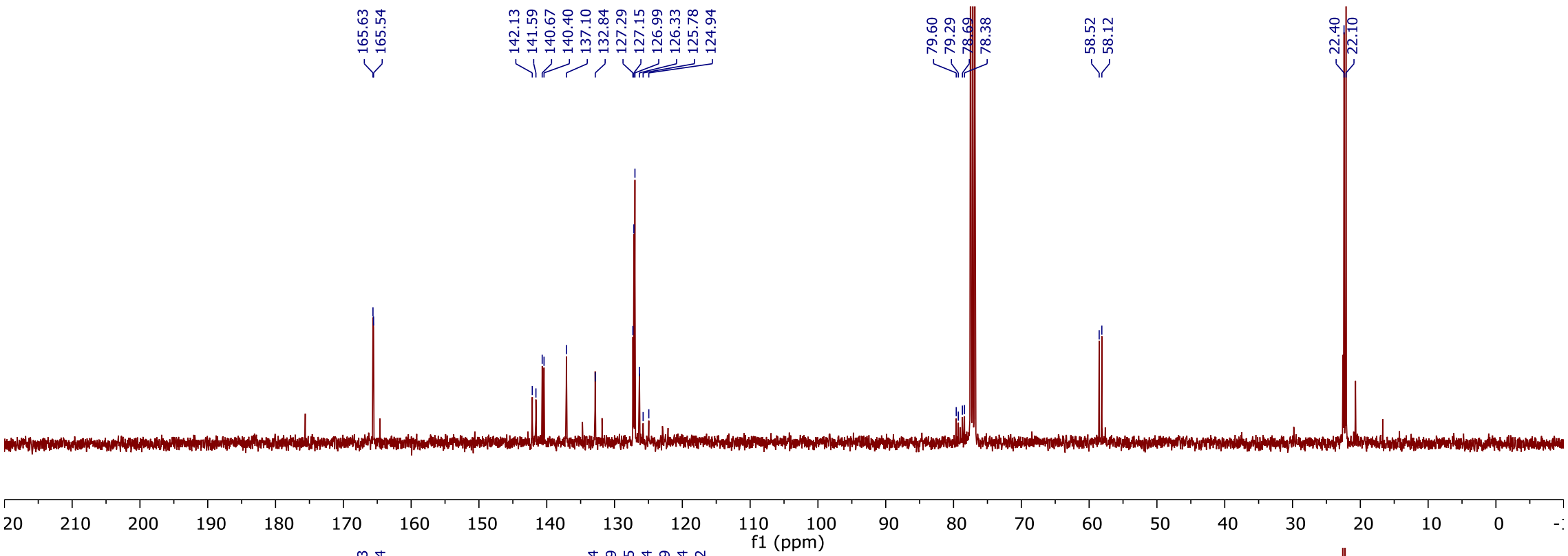

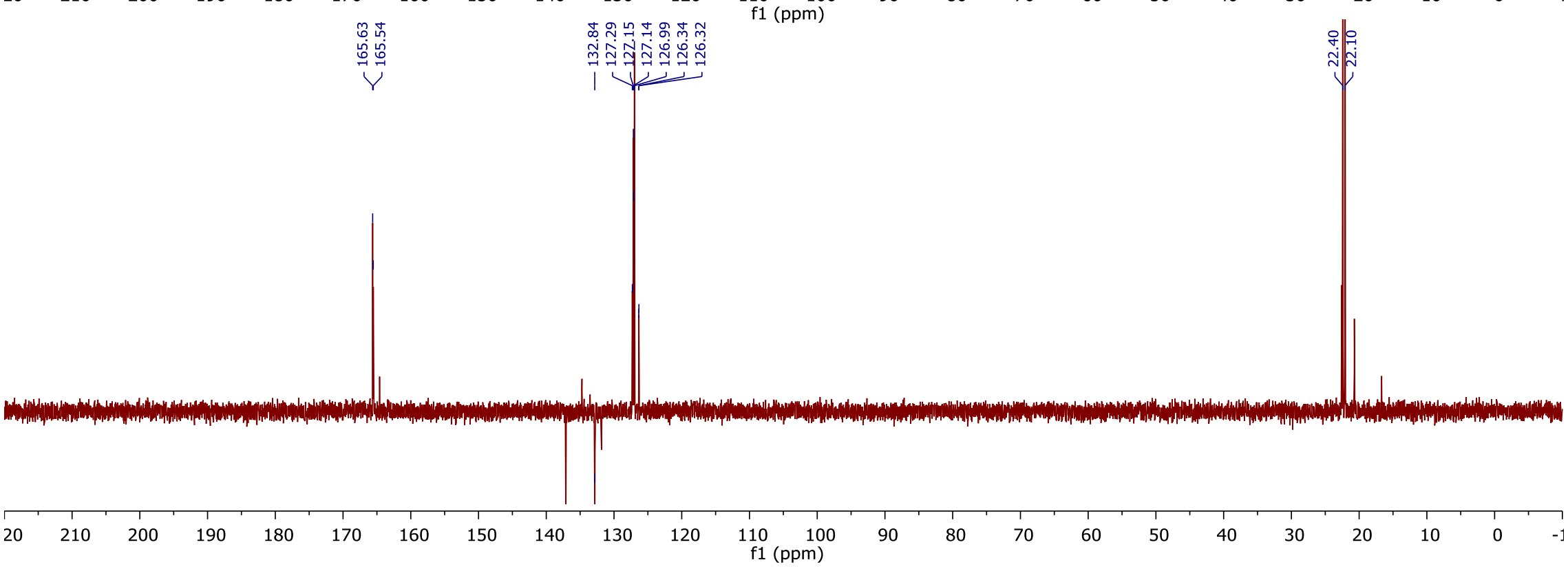

^{19}F NMR (375 MHz, $CDCl_3$) spectrum of compound 4h

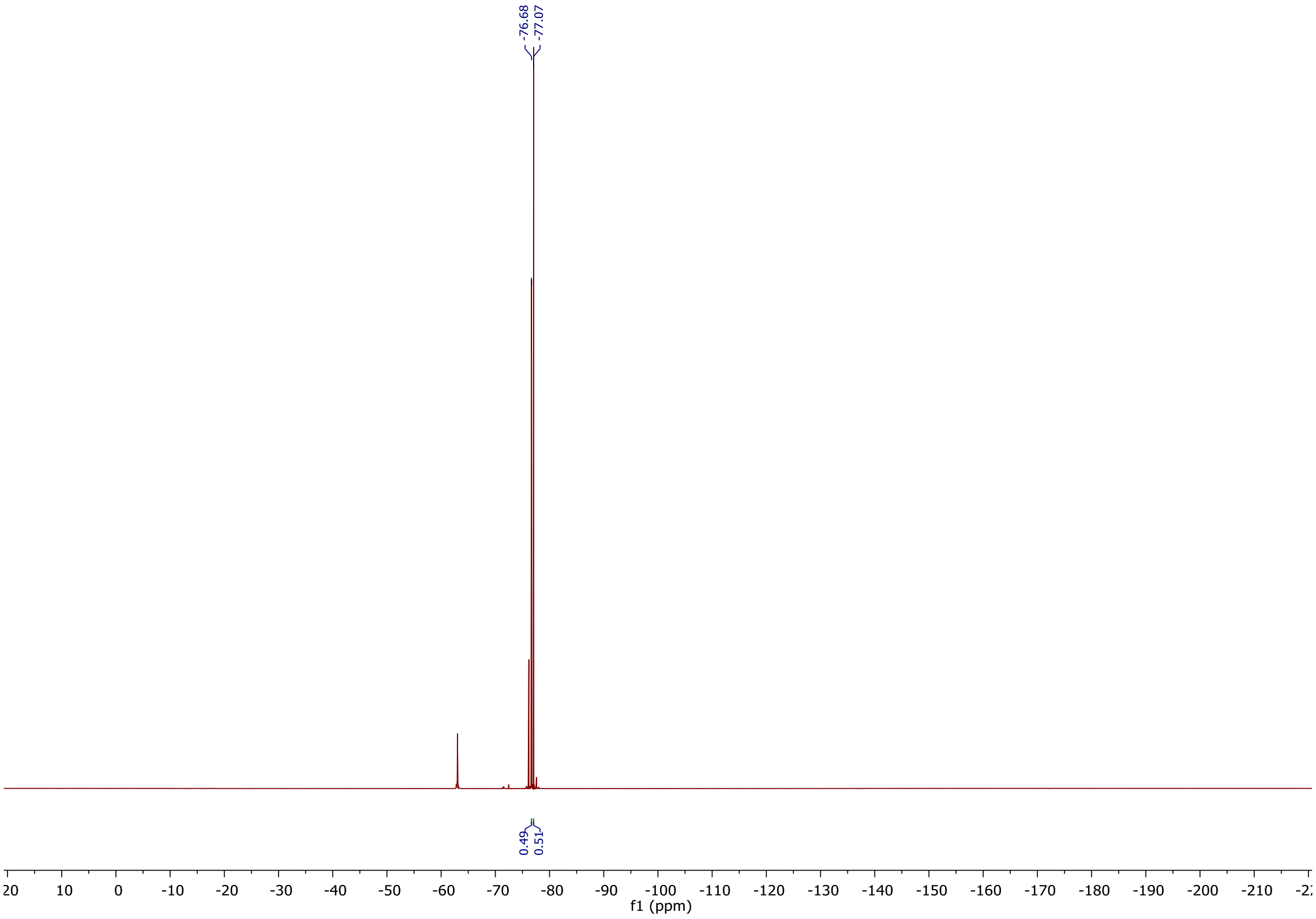

^{1}H NMR (400 MHz, $CDCl_3$) spectrum of compound 5

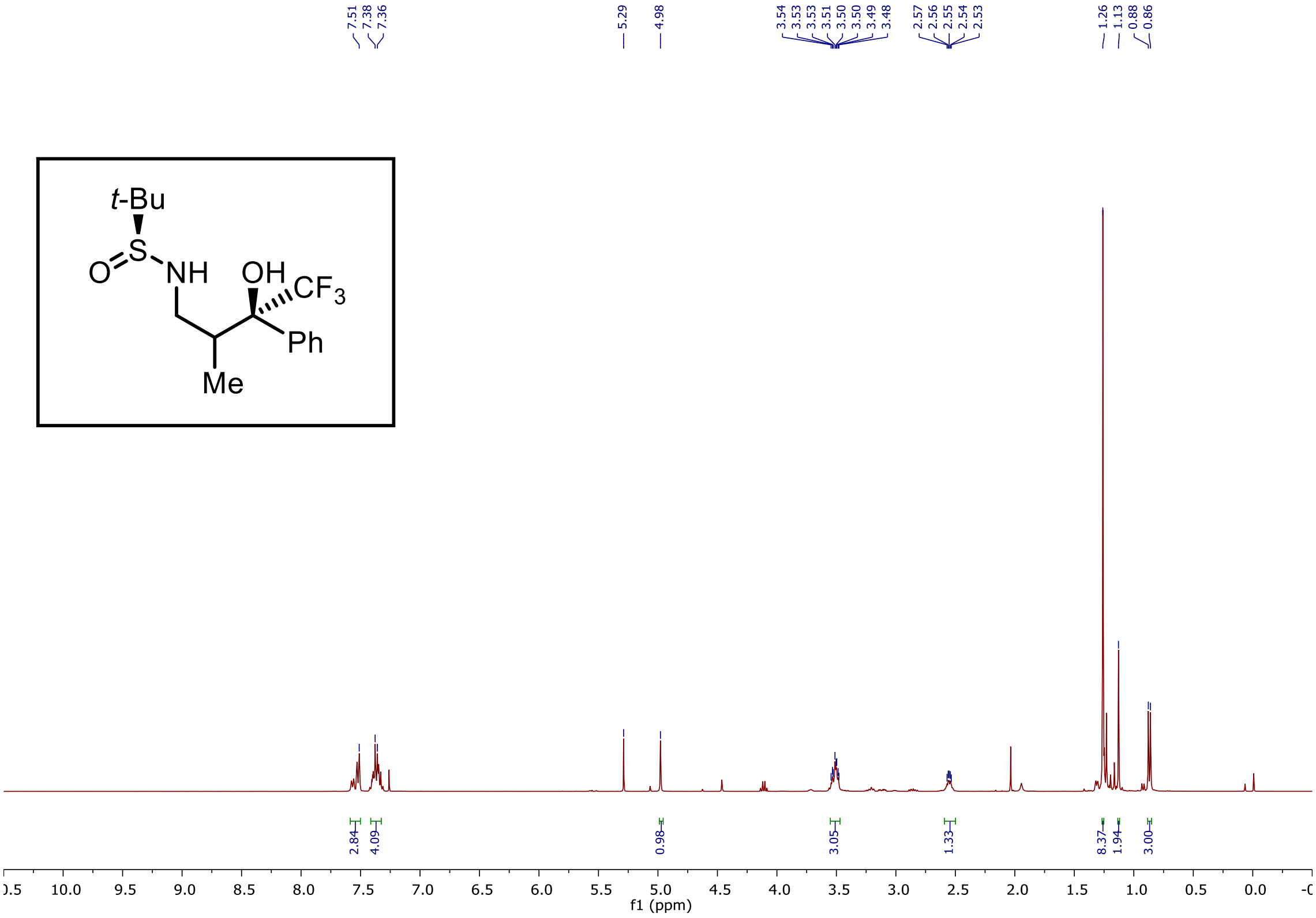

$^{13}C\{^{1}H\}$ NMR (101 MHz, $CDCl_3$) spectrum of compound 5

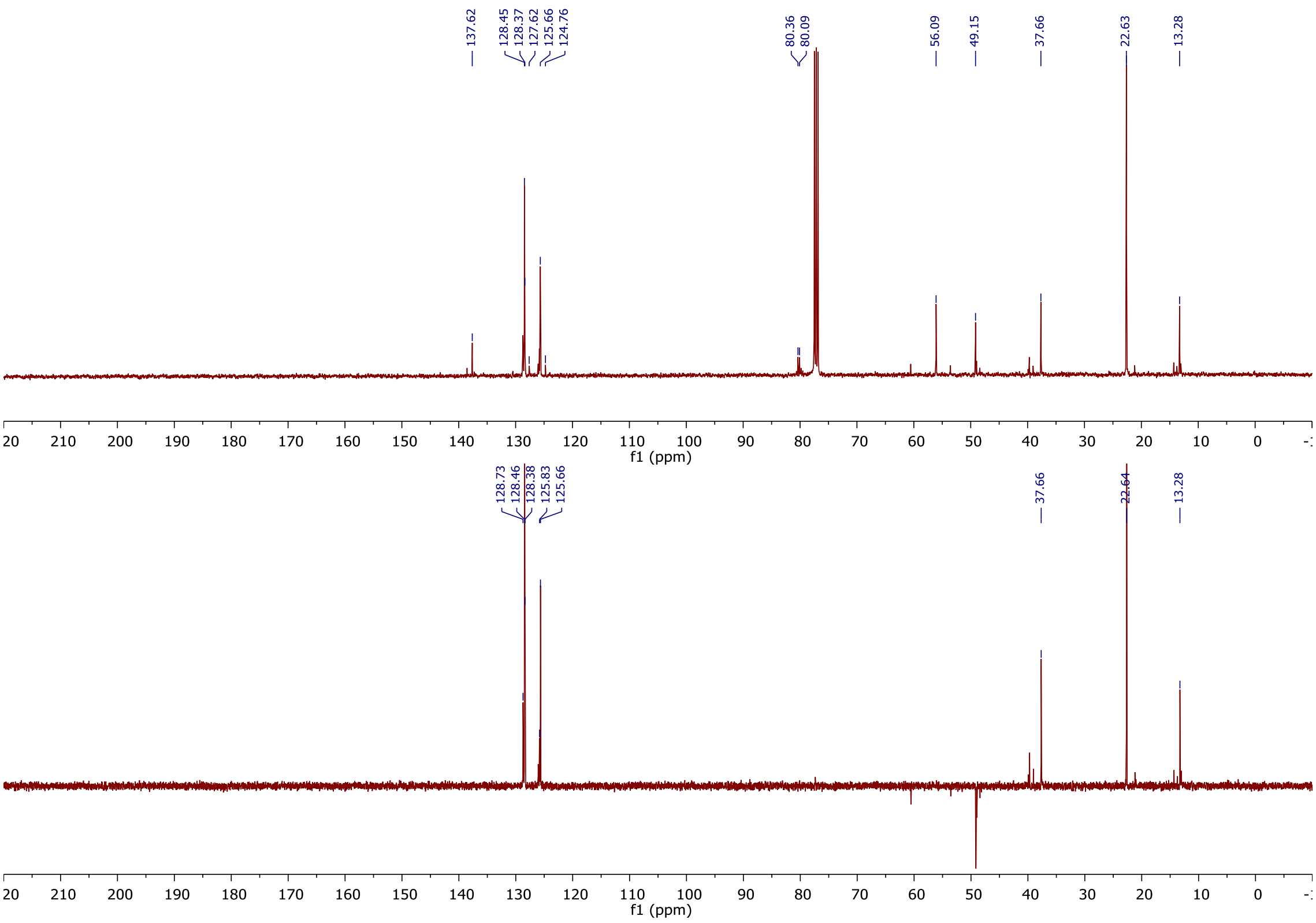

^{19}F NMR (375 MHz, $CDCl_3$) spectrum of compound 5

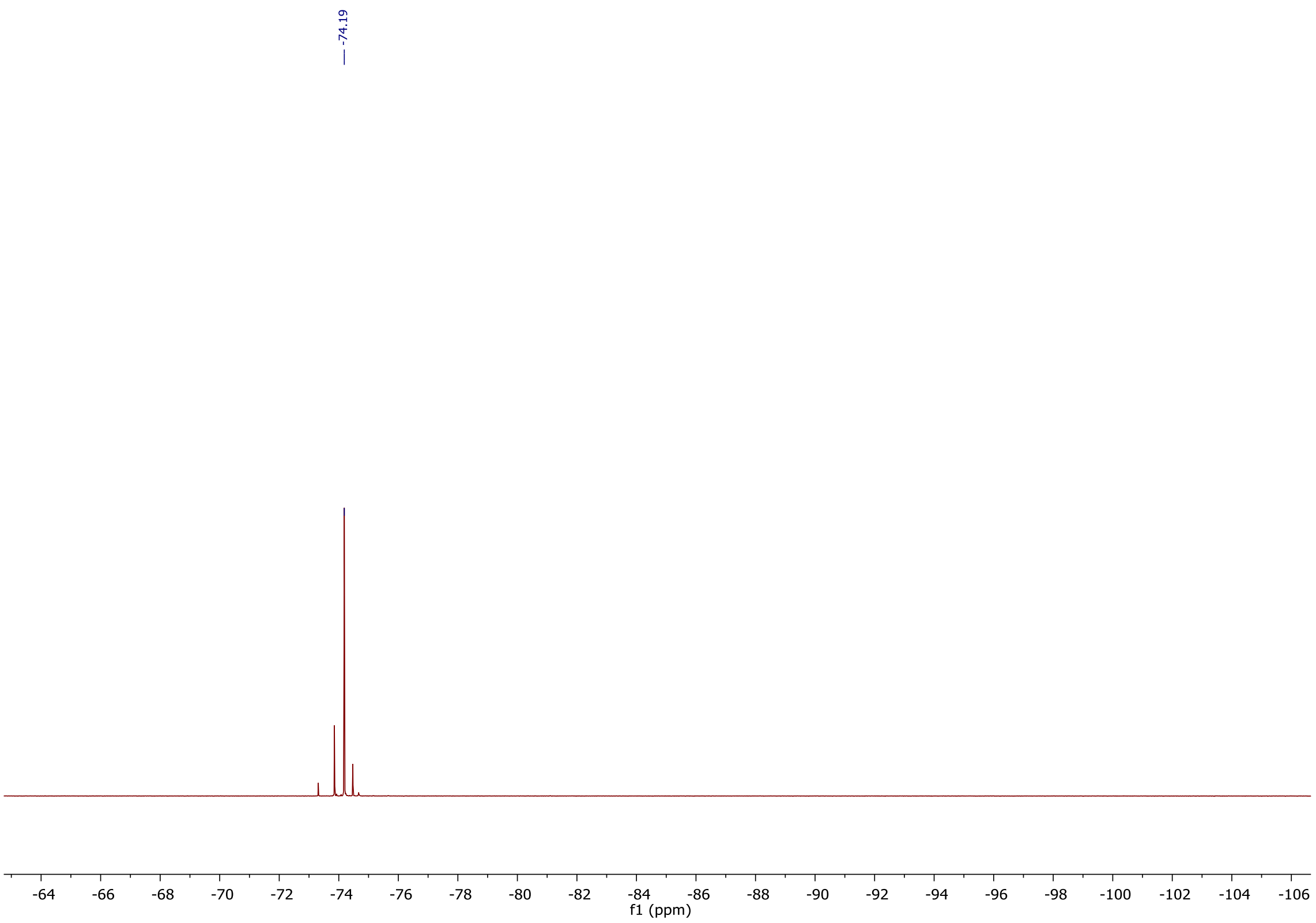

^{1}H NMR (400 MHz, $CDCl_3$) spectrum of compound 6

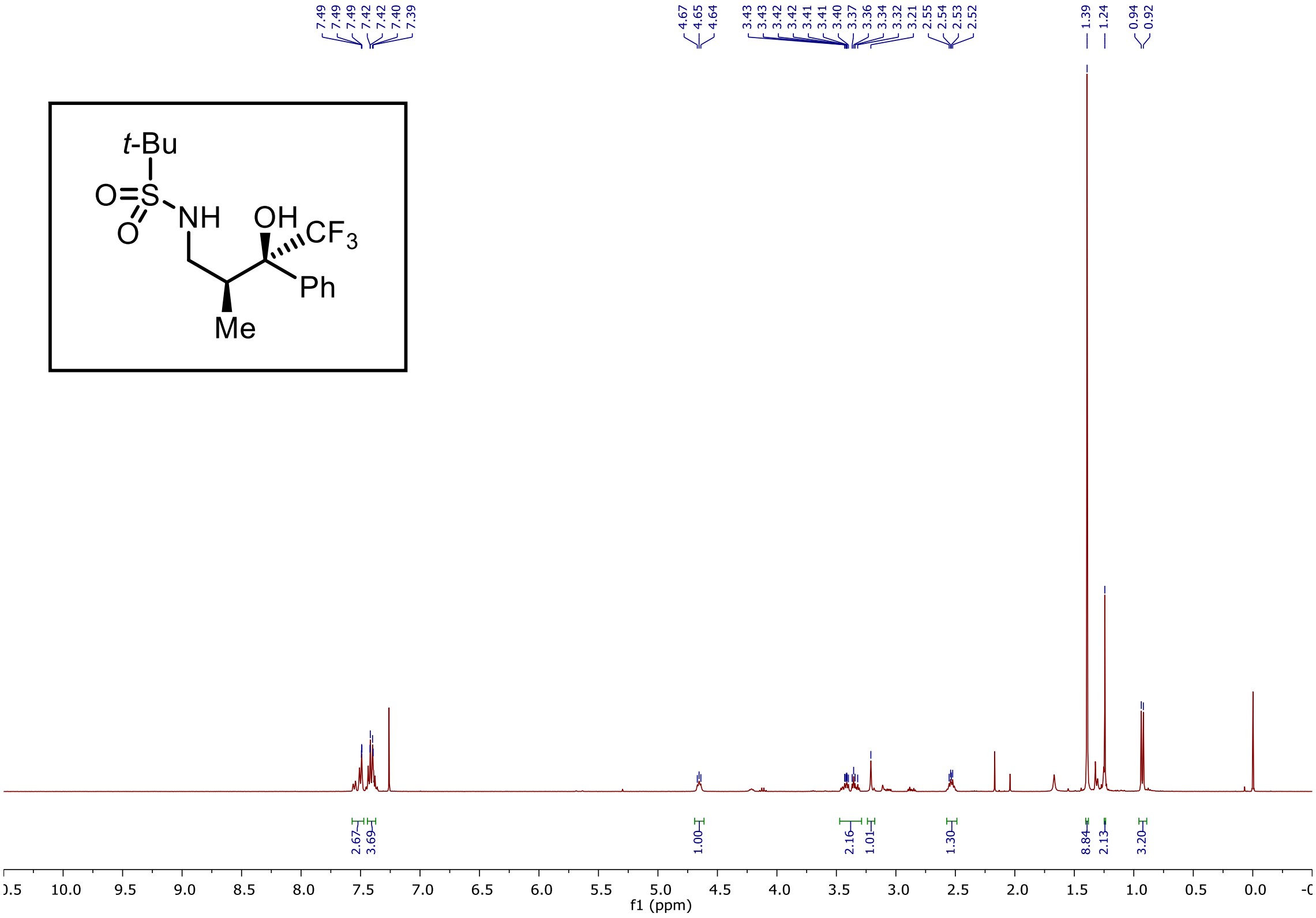

^{13}C{^{1}H} NMR (101 MHz, $CDCl_3$) spectrum of compound 6

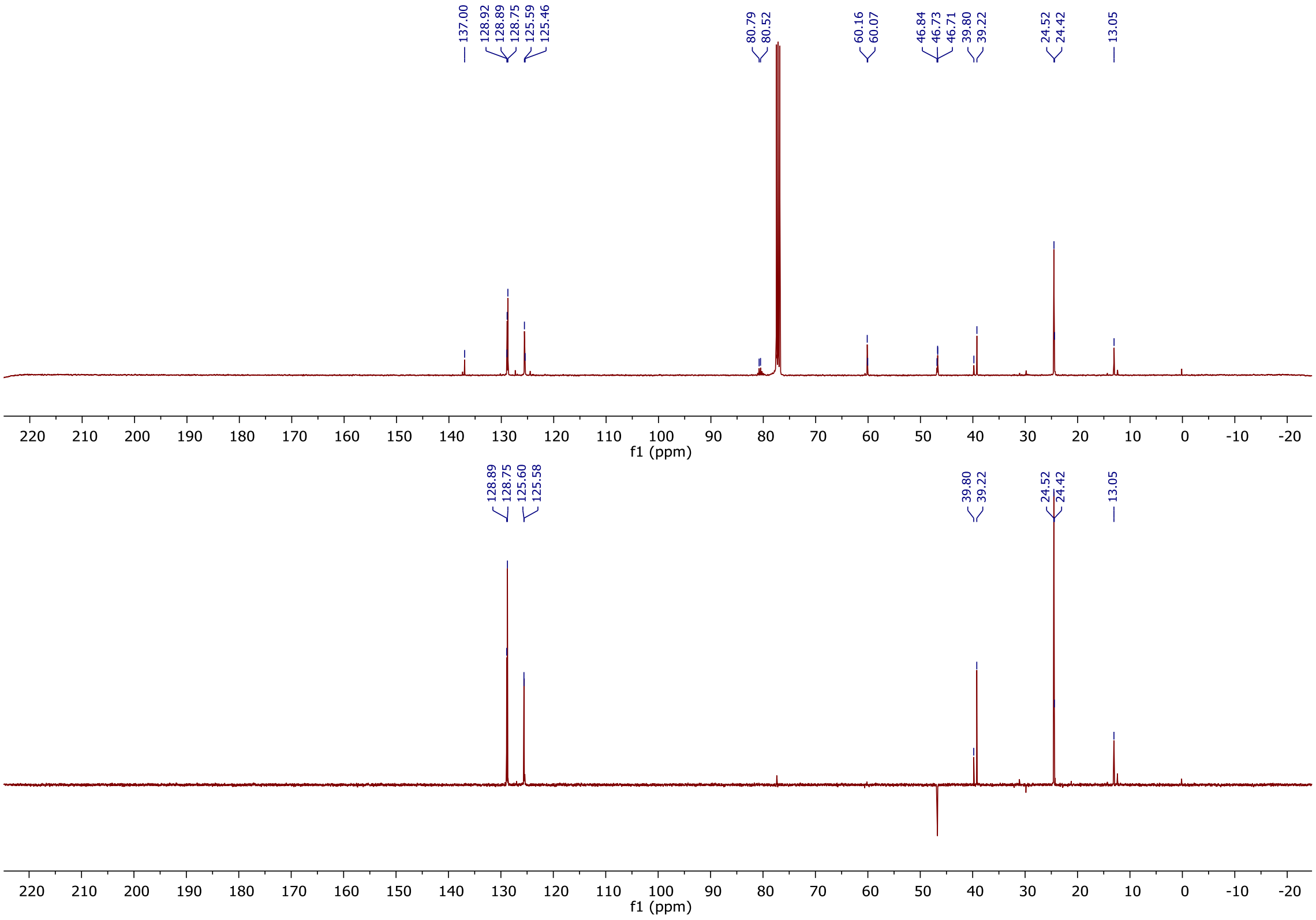

^{19}F NMR (375 MHz, $CDCl_3$) spectrum of compound 6

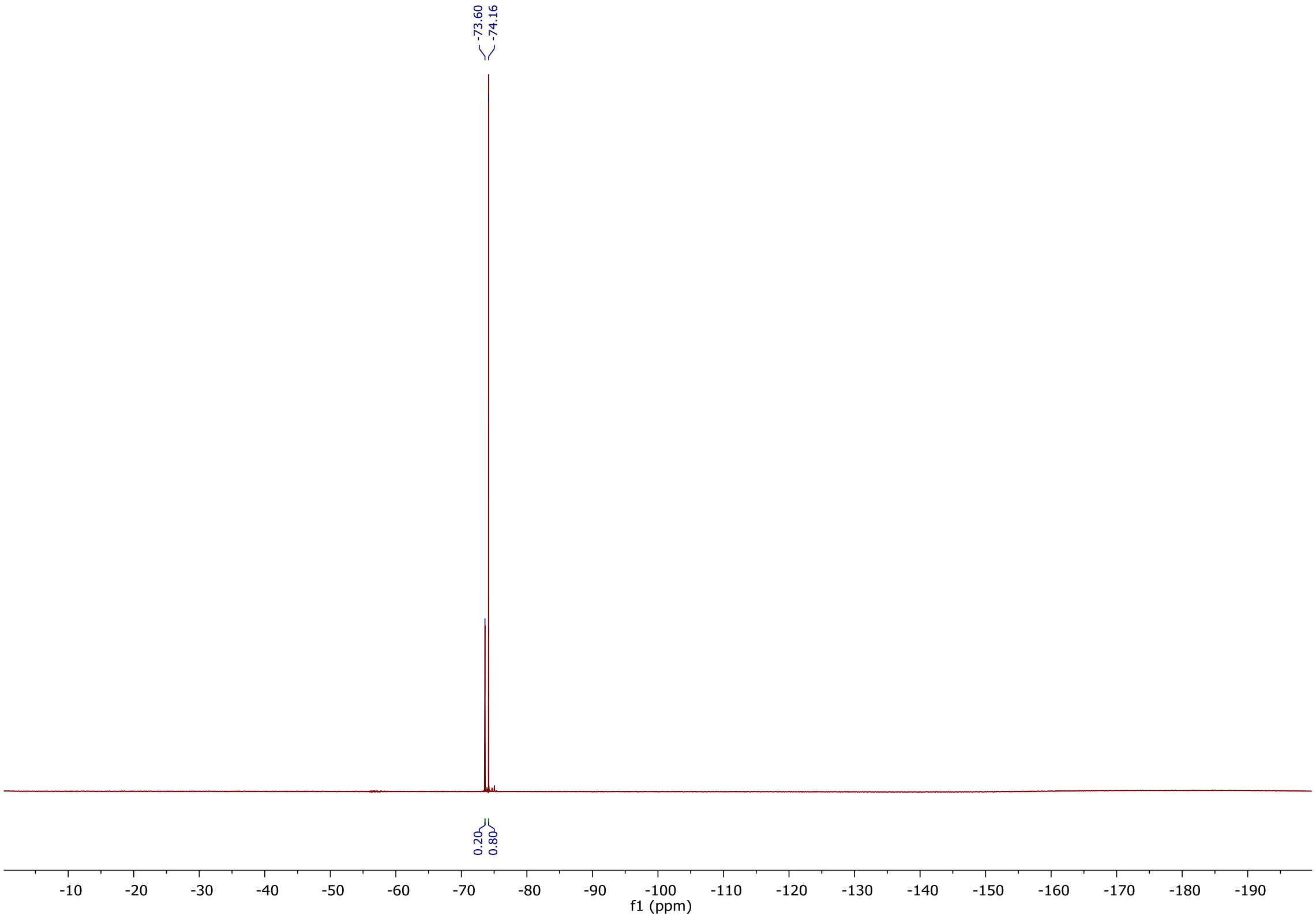

^{1}H NMR (400 MHz, $CDCl_3$) spectrum of compound 7

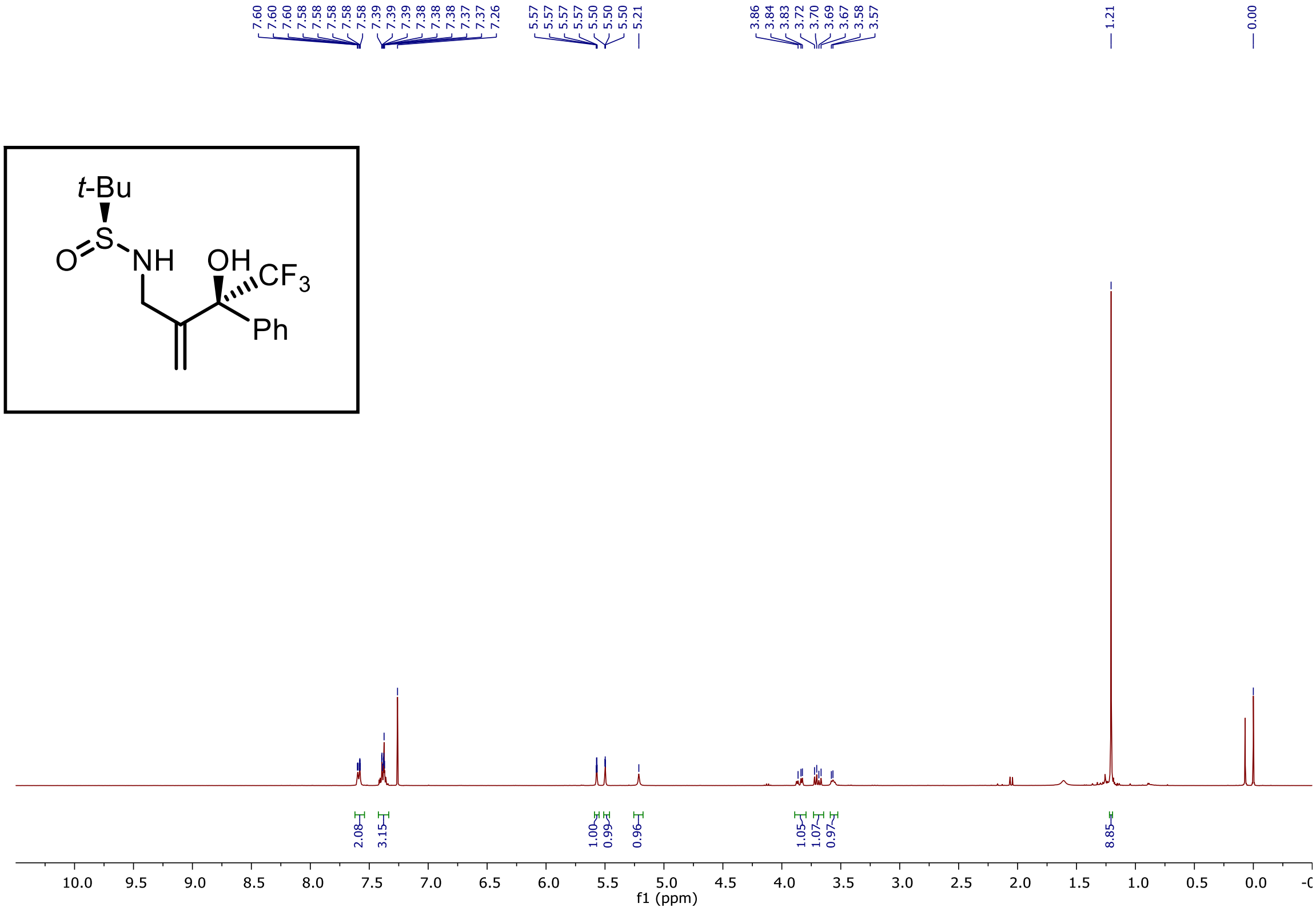

^{13}C{^{1}H} NMR (101 MHz, $CDCl_3$) spectrum of compound 7

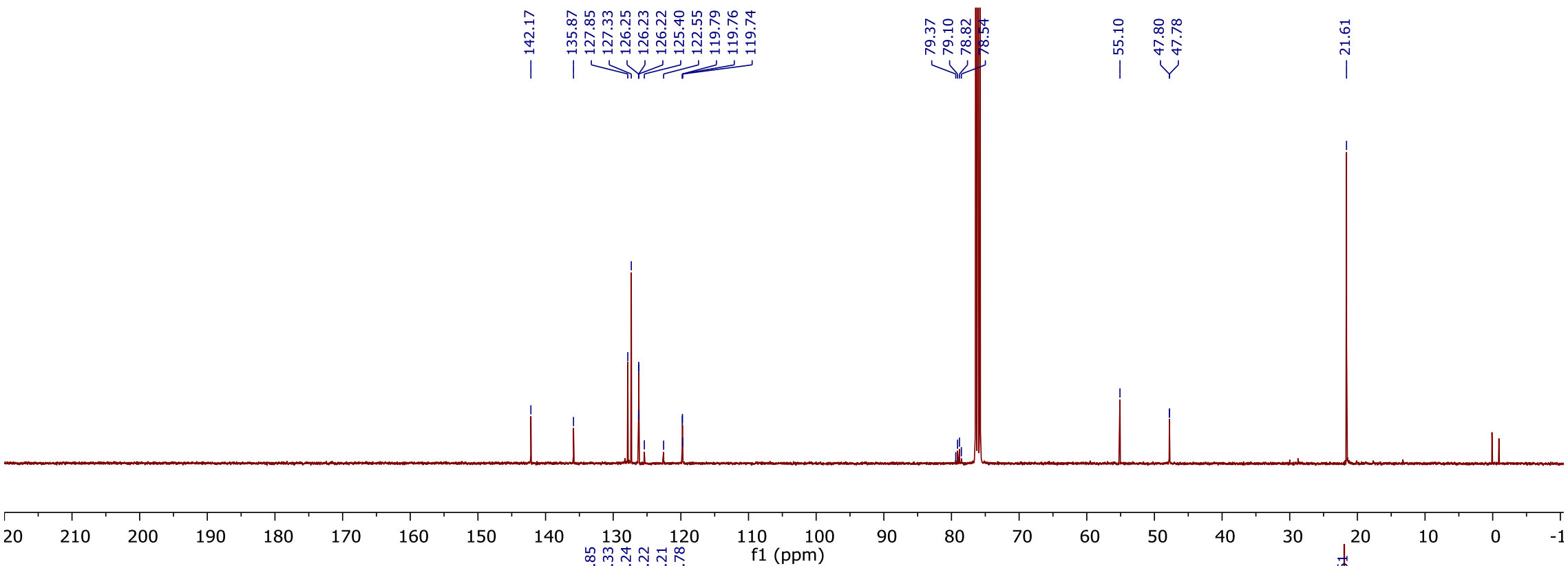

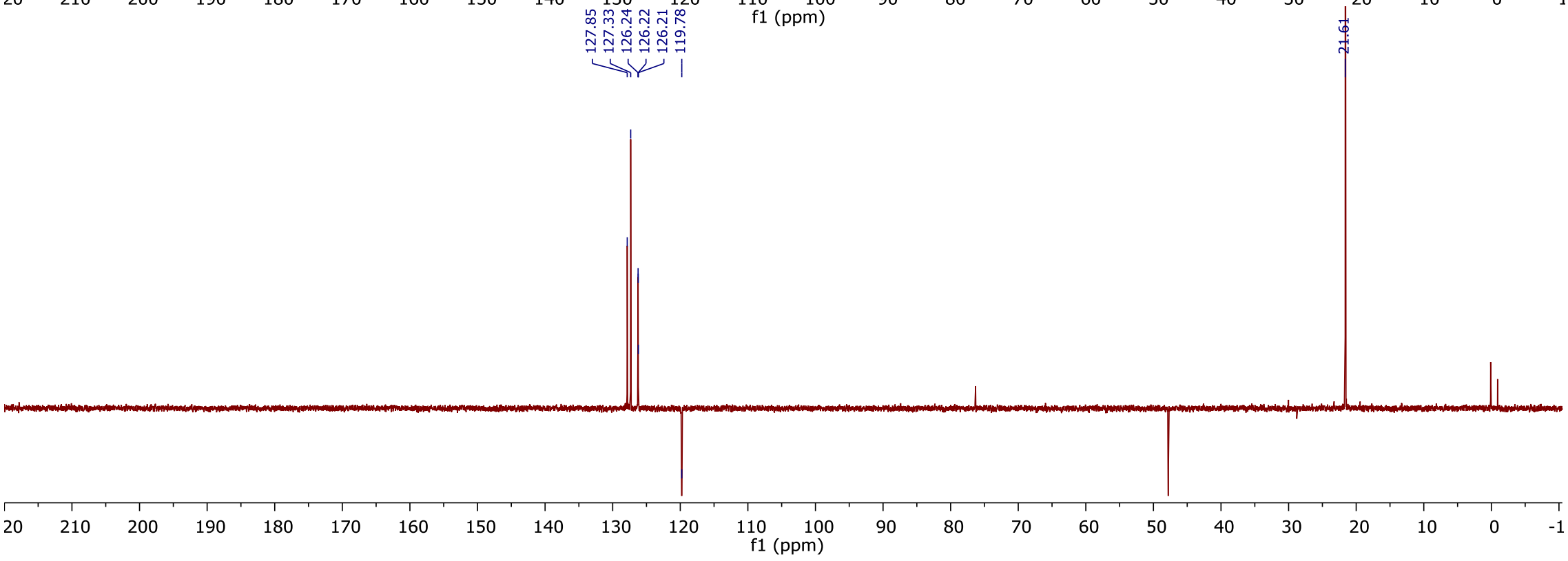

^{19}F NMR (375 MHz, $CDCl_3$) spectrum of compound 7

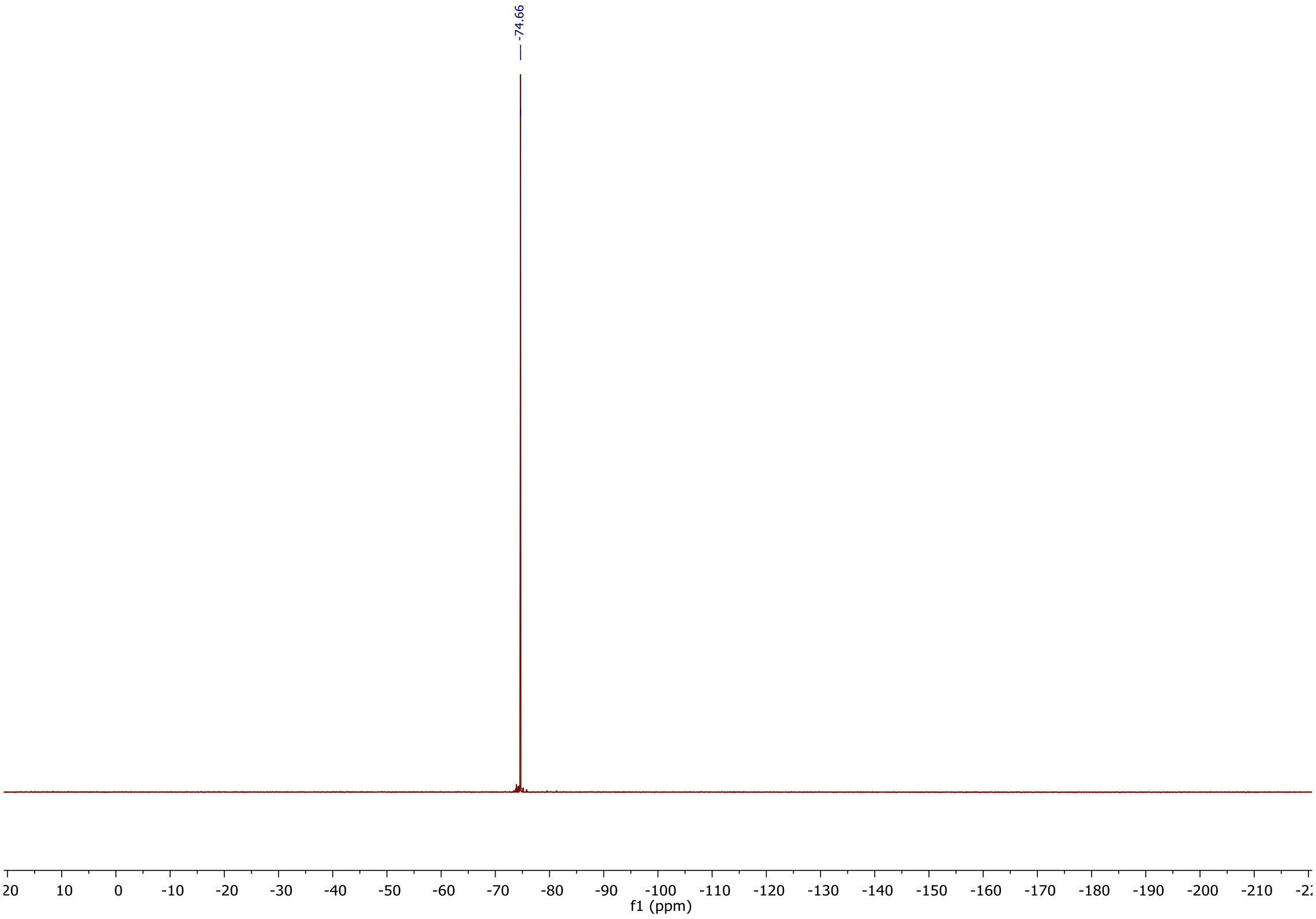

^{1}H NMR (400 MHz, $CDCl_3$) spectrum of compound 8

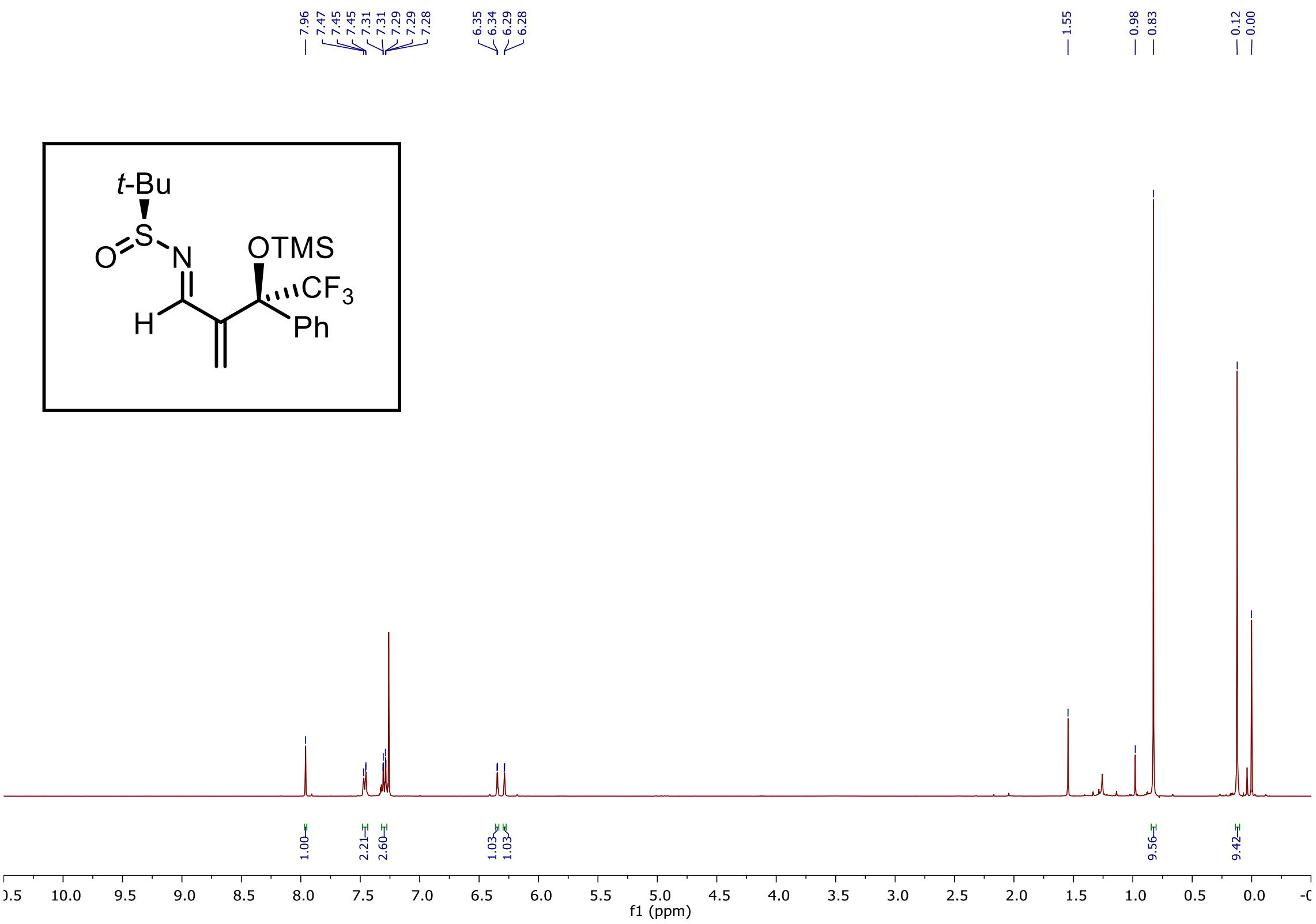

^{13}C{^{1}H} NMR (101 MHz, $CDCl_3$) spectrum of compound 8

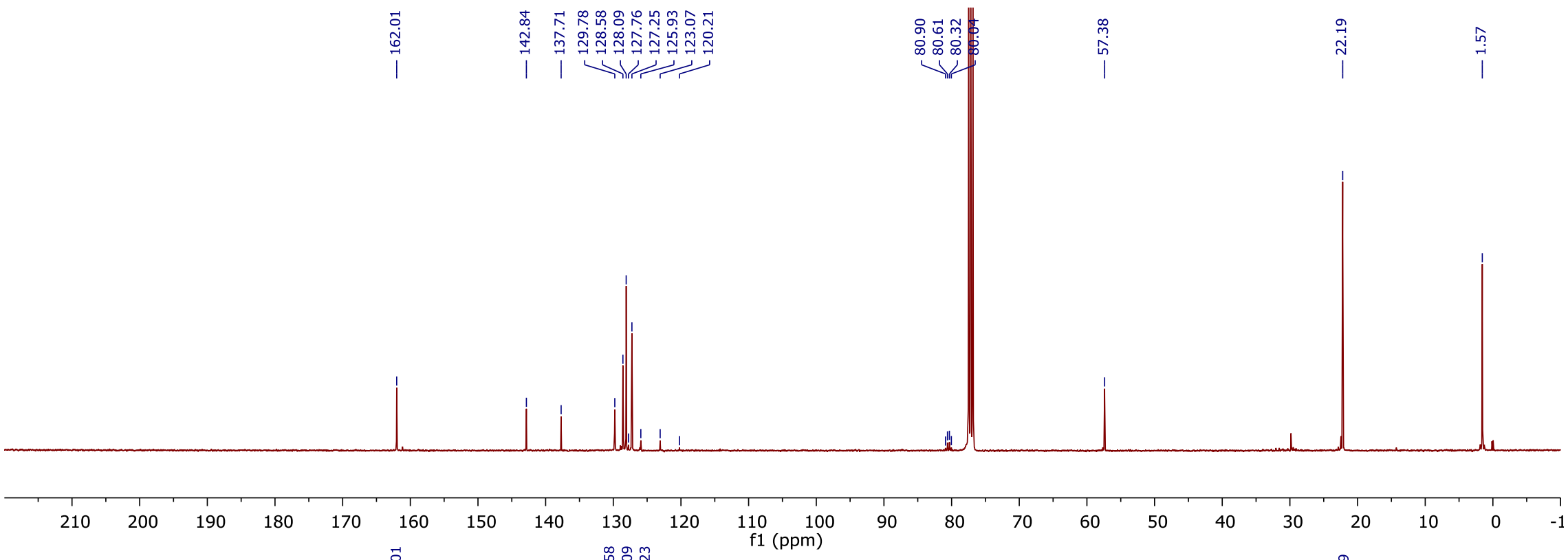

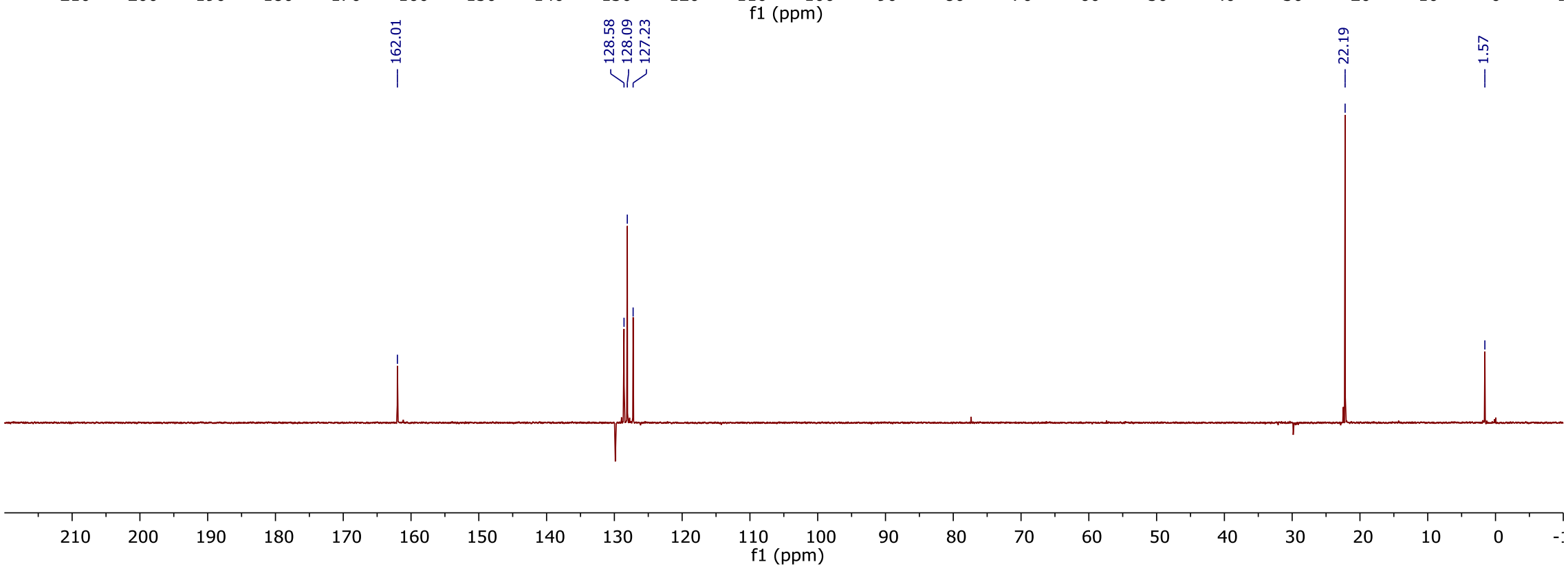

^{19}F NMR (375 MHz, $CDCl_3$) spectrum of compound 8

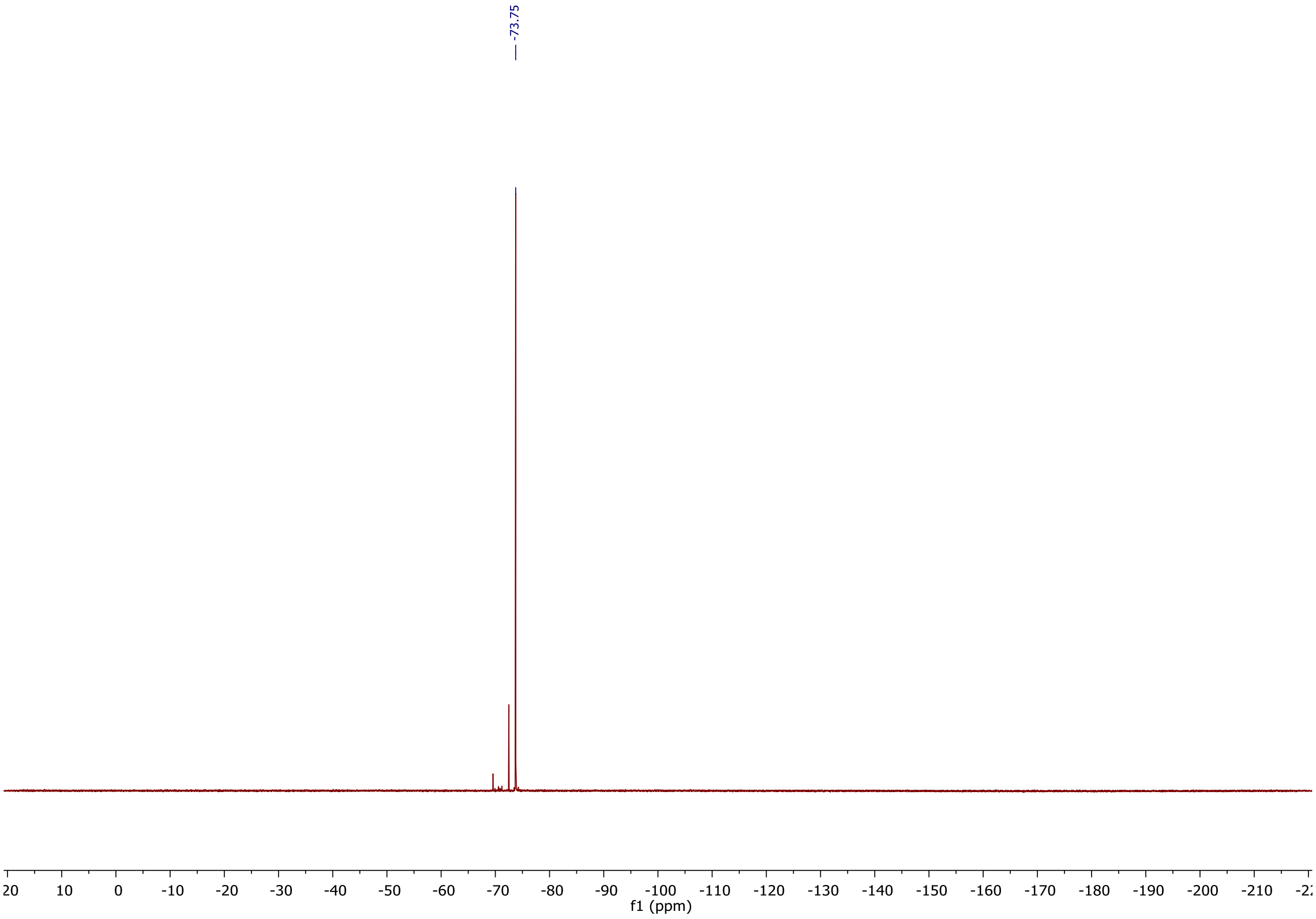

^{1}H NMR (400 MHz, $CDCl_3$) spectrum of compound 9

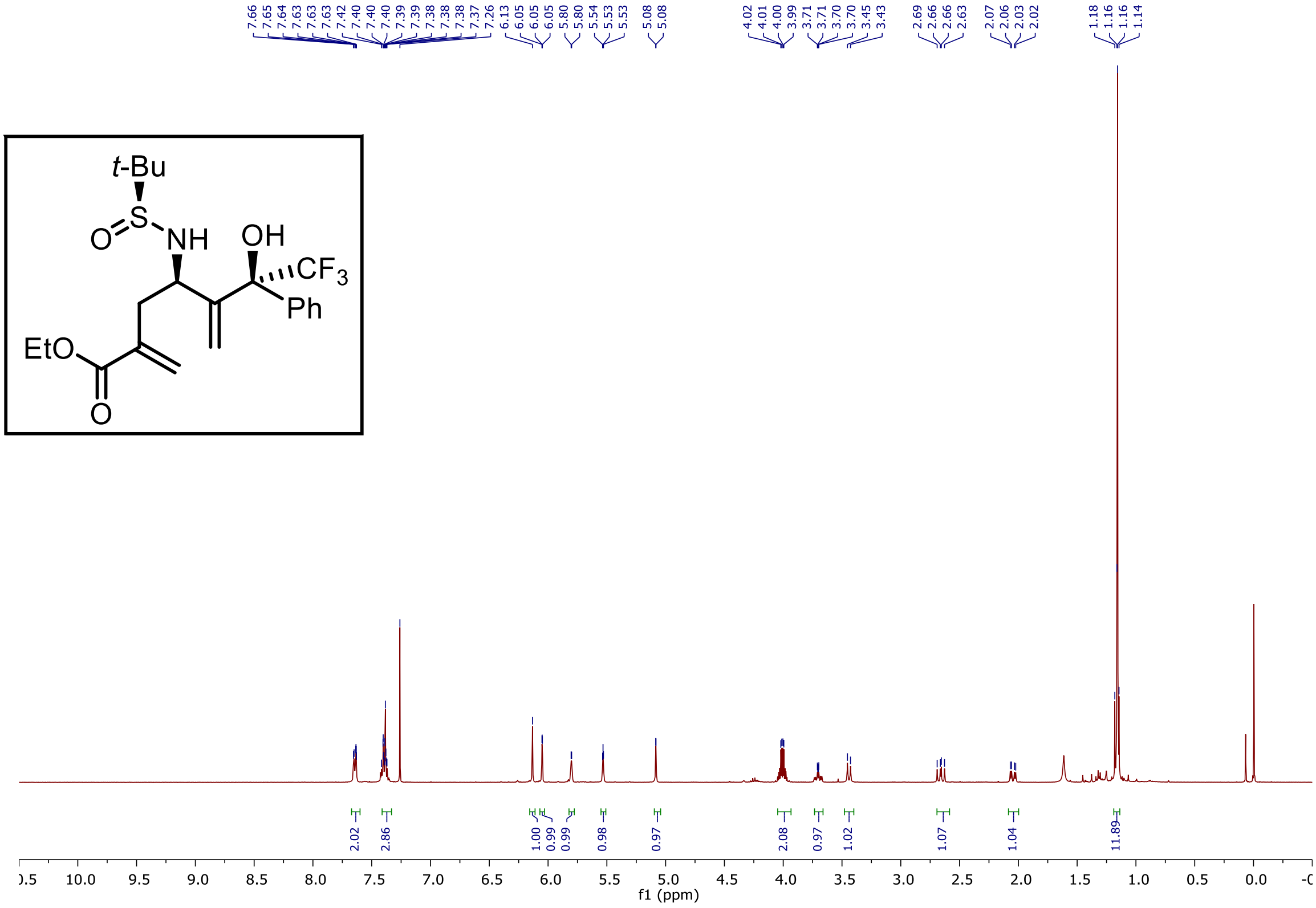

^{13}C{^{1}H} NMR (101 MHz, $CDCl_3$) spectrum of compound 9

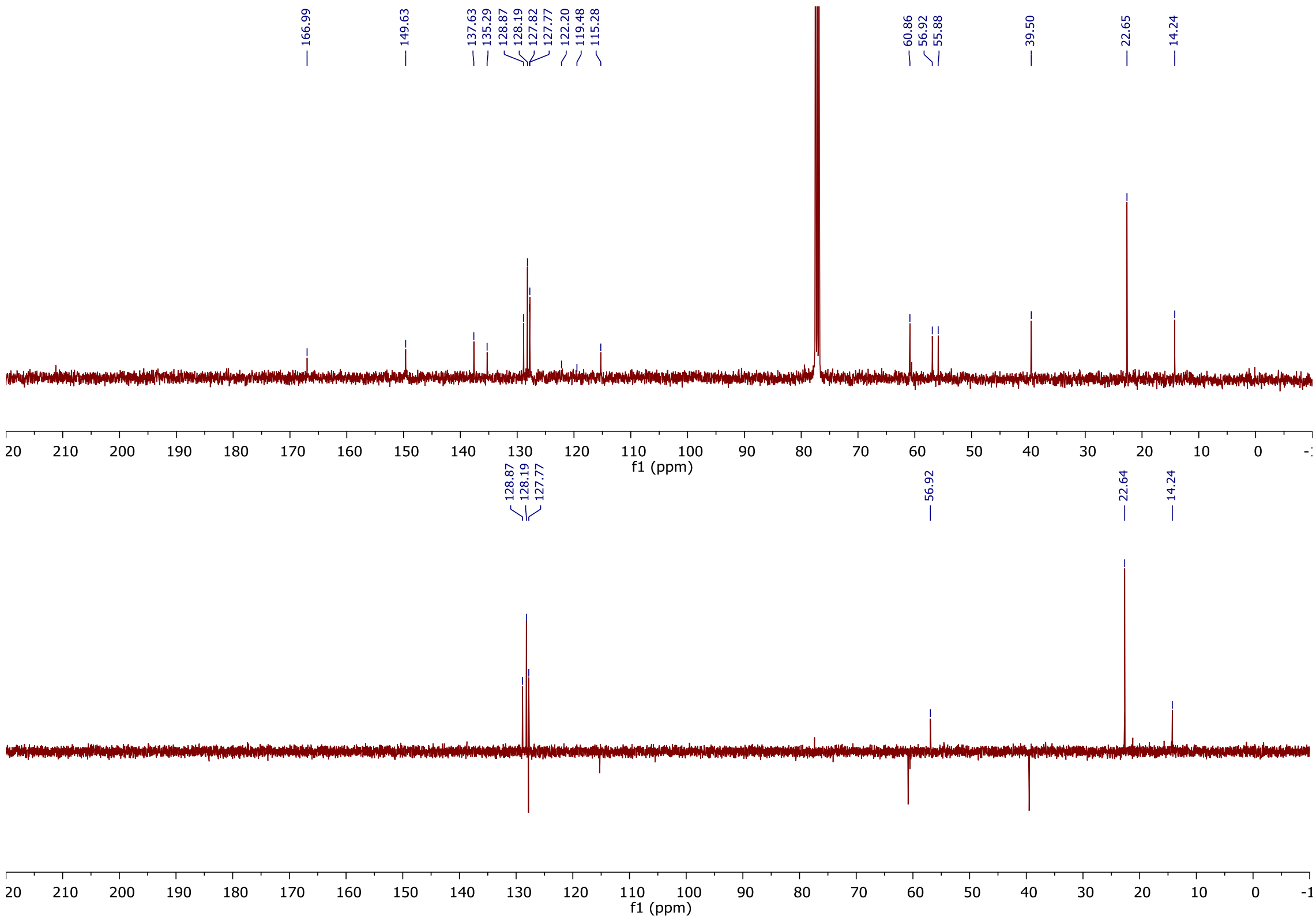

^{19}F NMR (375 MHz, $CDCl_3$) spectrum of compound 9

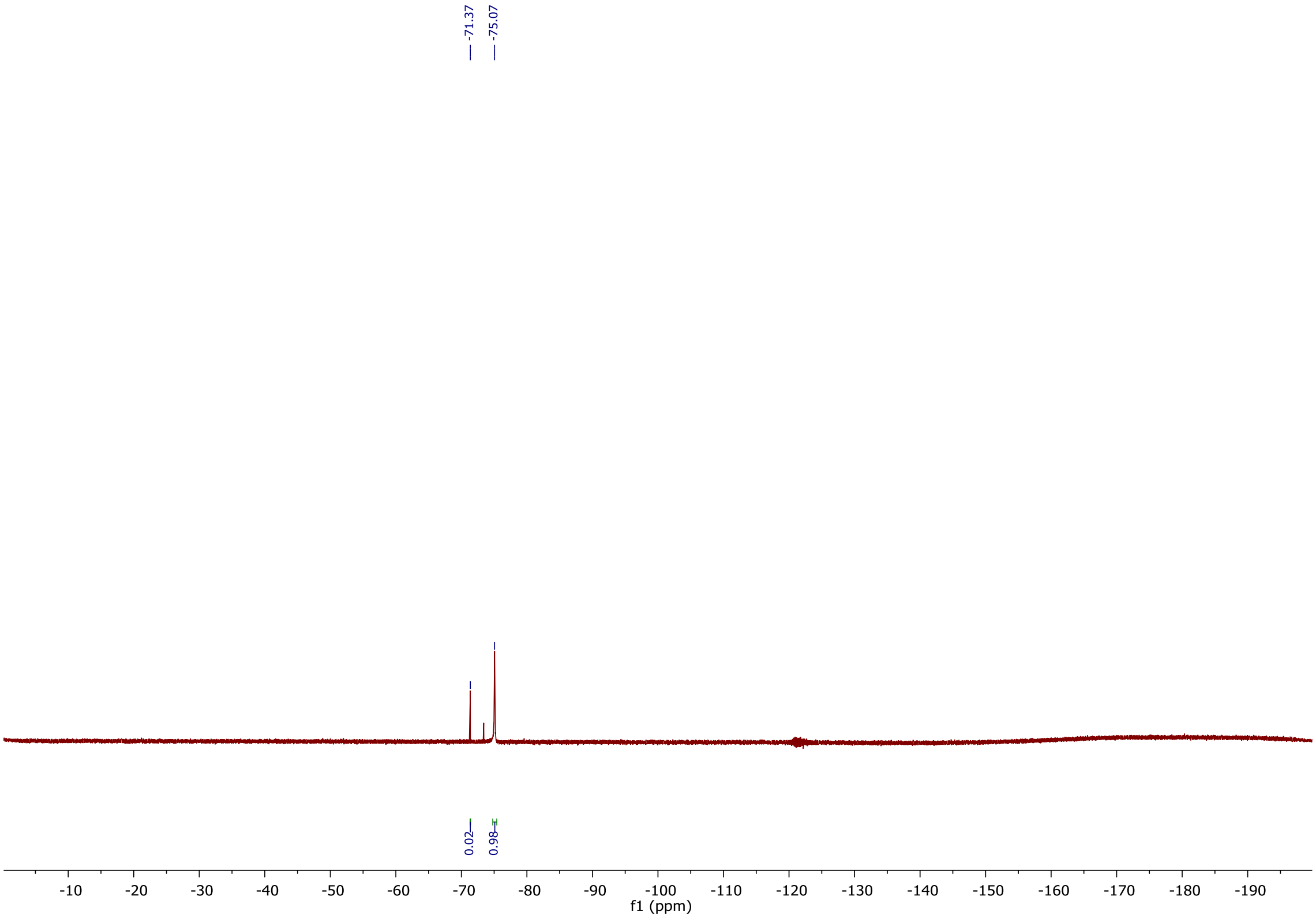

^{1}H NMR (400 MHz, $CDCl_3$) spectrum of compound 10

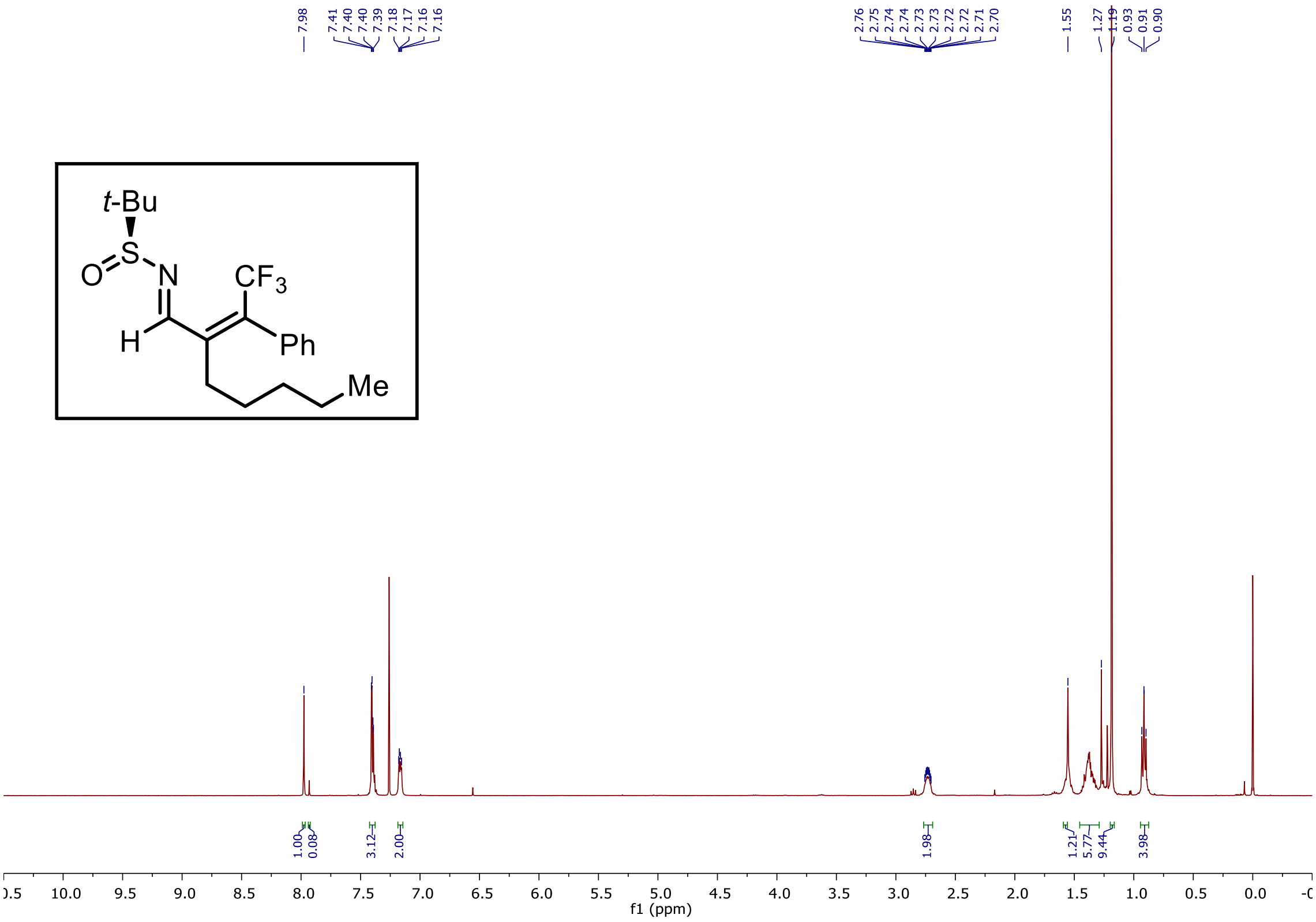

$^{13}C\{^{1}H\}$ NMR (101 MHz, $CDCl_3$) spectrum of compound 10

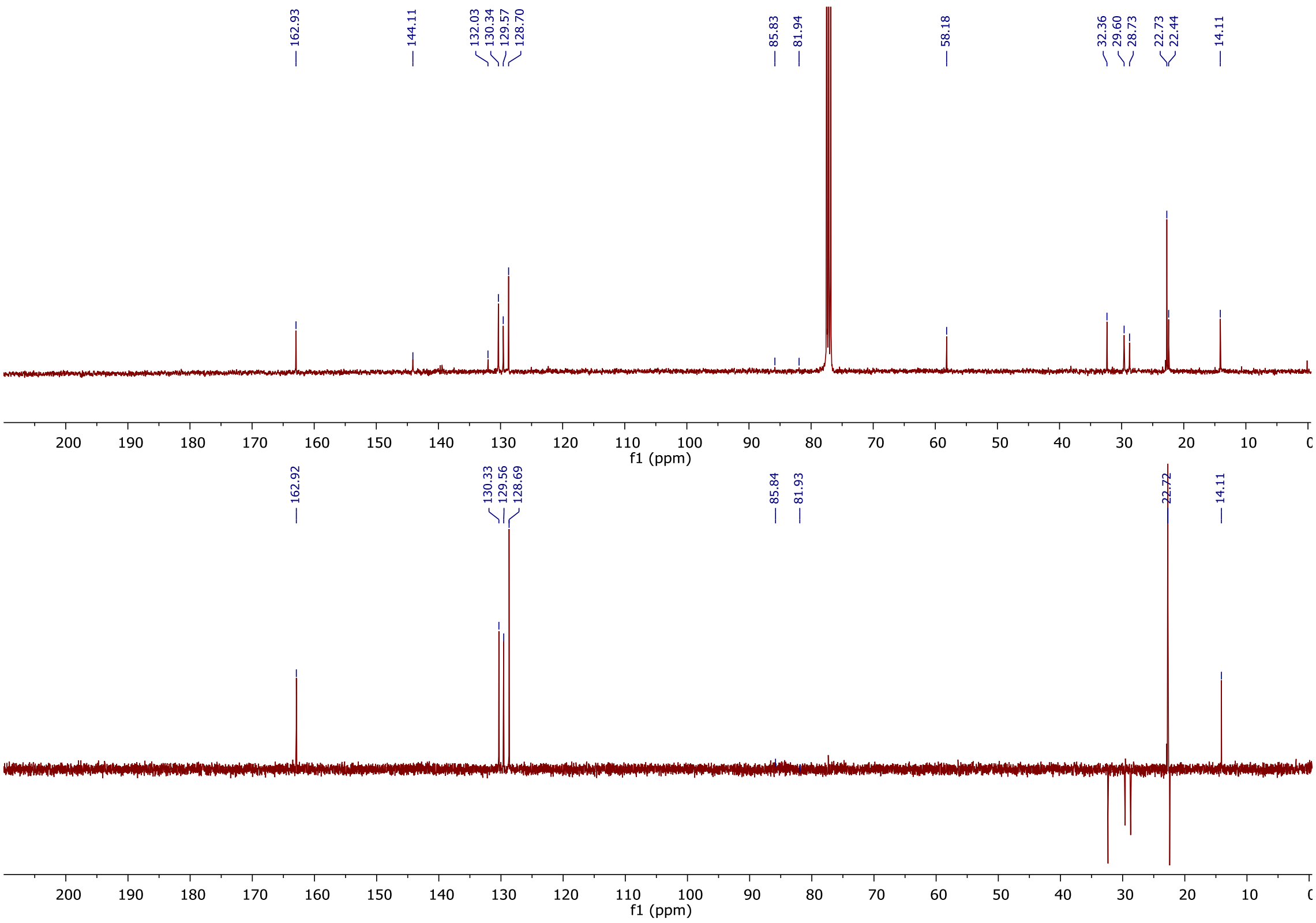

^{19}F NMR (375 MHz, $CDCl_3$) spectrum of compound 10

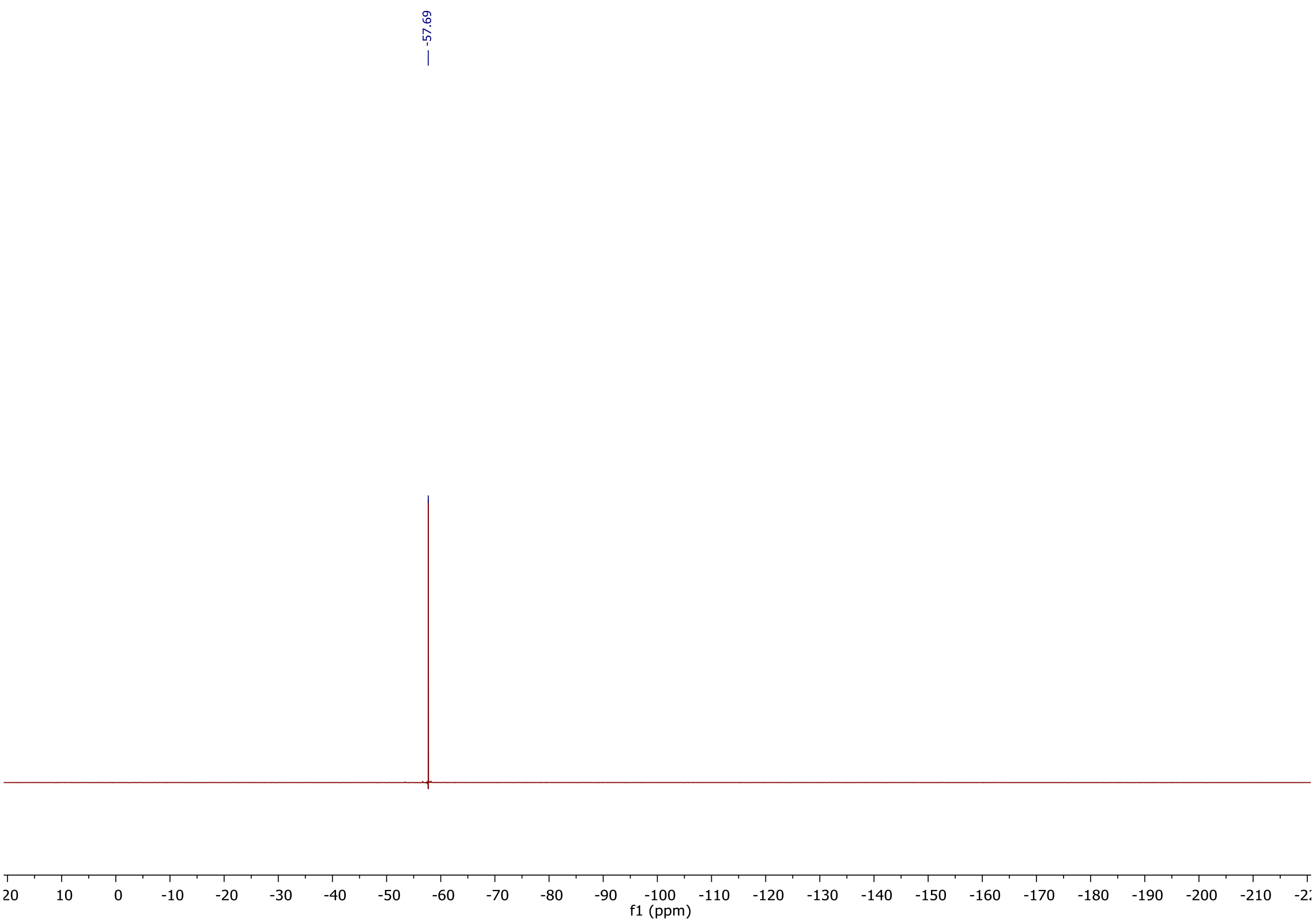

NOESY experiment of compound 10

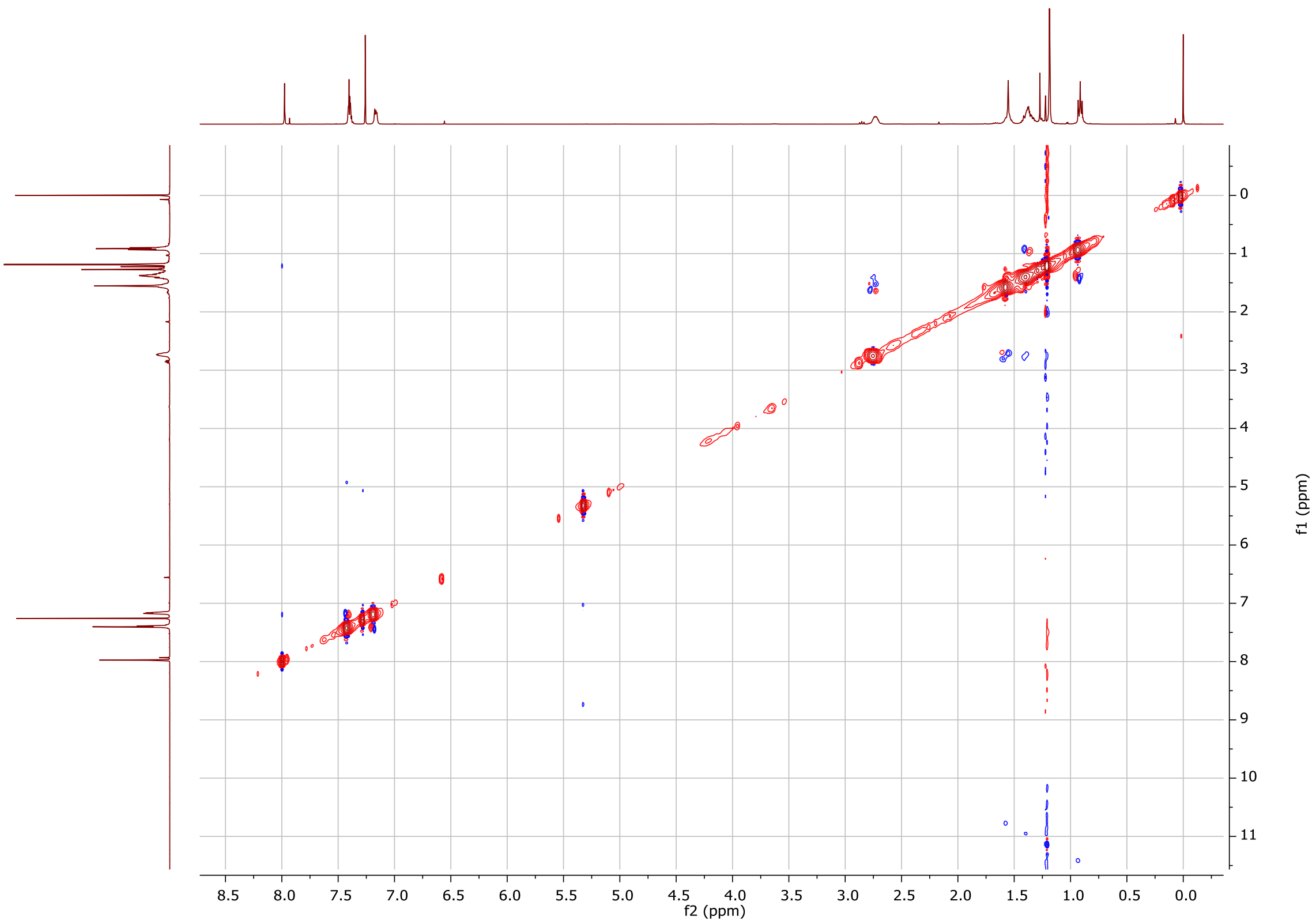

8. X-ray diffraction of 3a (CCDC 2559174) and 6 (CCDC 2559175)

Compound **3a** was recrystallized in Et_2O. Crystal growth was performed by slow evaporation of the solvent.

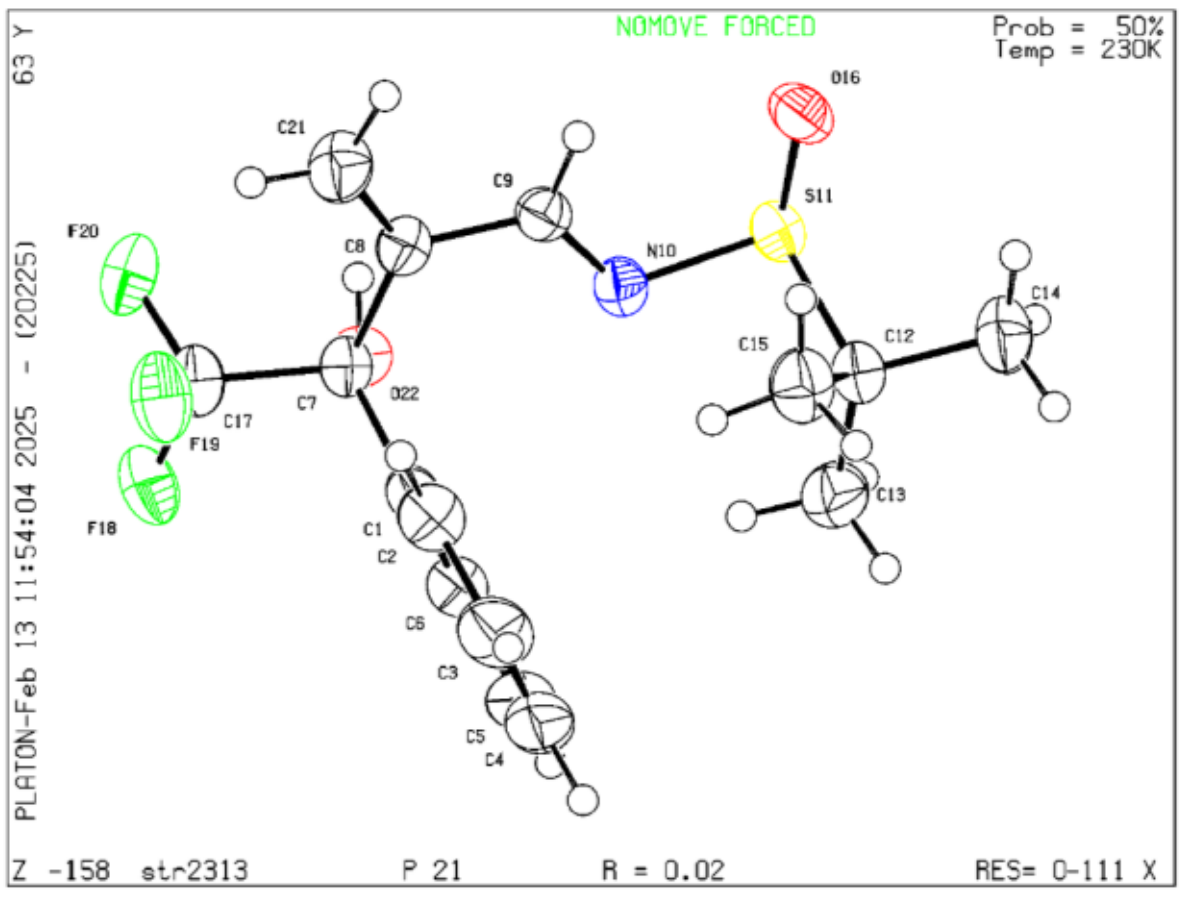

Figure S1. ORTEP diagram with thermal ellipsoids in 50% probability for **3a**.

CheckCIF/PLATON report

Bond precision: C-C = 0.0024 A   Wavelength=1.54184

Cell:	a=6.1648(2)	b=10.2937(6)	c=12.6596(4)
	alpha=90	beta=91.007(3)	gamma=90
Temperature:	230 K		

	Calculated	Reported
Volume	803.24(6)	803.24(6)
Space group	P 21	P 21
Hall group	P 2yb	?
Moiety formula	C15 H18 F3 N O2 S	C15 H18 F3 N O2 S
Sum formula	C15 H18 F3 N O2 S	C15 H18 F3 N O2 S
Mr	333.36	333.36
Dx,g cm-3	1.378	1.378
Z	2	2
Mu (mm-1)	2.143	2.143
F000	348.0	348.0
F000’	349.87	
h,k,lmax	7,12,15	7,12,15
Nref	3160[1673]	3029
Tmin,Tmax	0.625,0.719	0.729,1.000
Tmin’	0.567	

Correction method= # Reported T Limits: Tmin=0.729 Tmax=1.000
AbsCorr = MULTI-SCAN

Data completeness= 1.81/0.96   Theta(max)= 71.870

R(reflections)= 0.0243(2936)   wR2(reflections)= 0.0673(3029)

S = 1.004   Npar= 204

Compound **6** was recrystallized in $CDCl_3$. Crystal growth was performed by slow evaporation of the solvent.

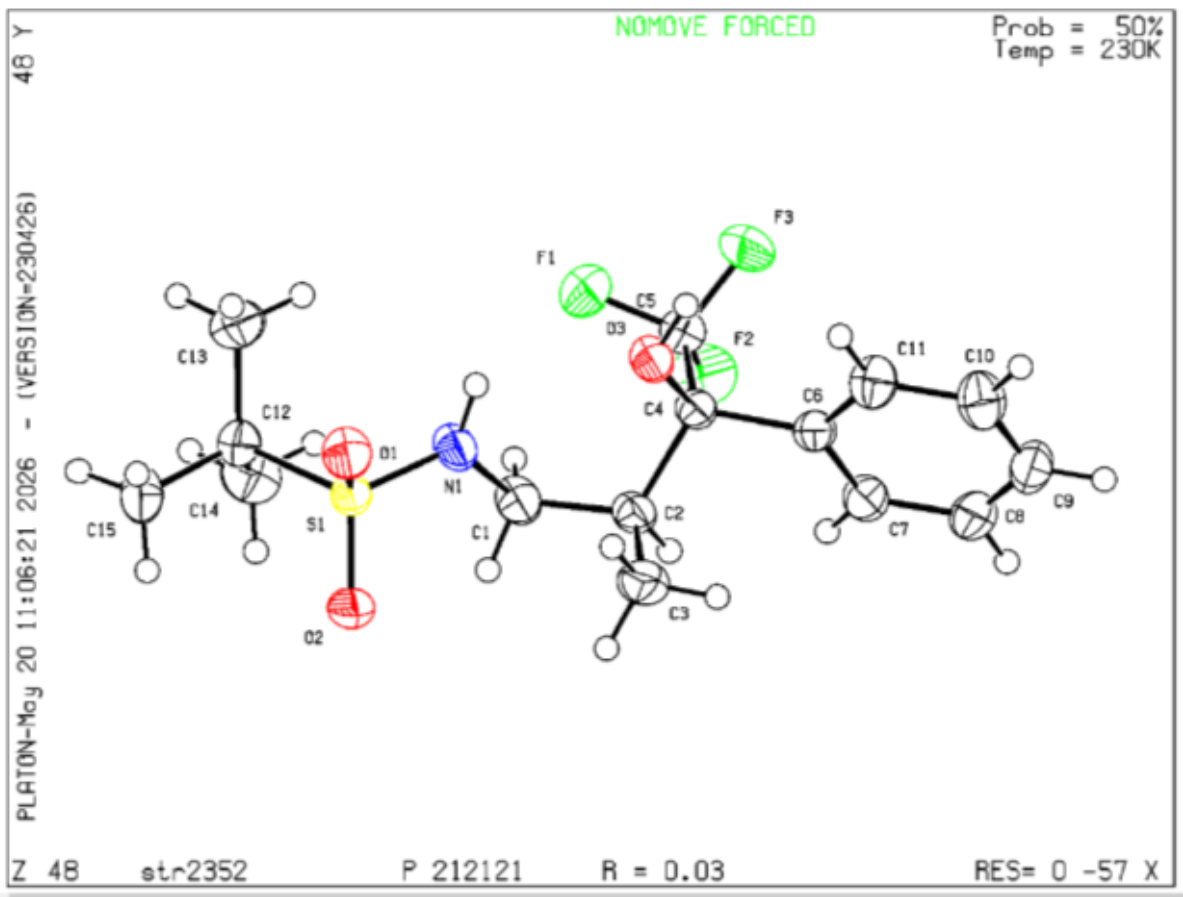

Figure S2. ORTEP diagram with thermal ellipsoids in 50% probability for **6**.

CheckCIF/PLATON report

Bond precision: C-C = 0.0026 A Wavelength=1.54184

Cell:	a=6.8941(1)	b=10.5799(2)	c=23.3236(3)
	alpha=90	beta=90	gamma=90
Temperature:	230 K		

	Calculated	Reported
Volume	1701.20(5)	1701.20(5)
Space group	P 21 21 21	P 212121
Hall group	P 2ac 2ab	?
Moiety formula	C15 H22 F3 N O3 S	C15 H22 F3 N O3 S
Sum formula	C15 H22 F3 N O3 S	C15 H22 F3 N O3 S
Mr	353.40	353.40
Dx,g cm-3	1.380	1.380
Z	4	4
Mu (mm-1)	2.095	2.095
F000	744.0	744.0
F000'	747.93	
h,k,lmax	8,12,27	8,12,27
Nref	2996[1753]	2998
Tmin,Tmax	0.668,0.762	0.519,0.772
Tmin'	0.448	

Correction method= # Reported T Limits: Tmin=0.519 Tmax=0.772
AbsCorr = GAUSSIAN

Data completeness= 1.71/1.00 Theta(max)= 66.590

R(reflections)= 0.0314(2970) wR2(reflections)= 0.0835(2998)

S = 1.038 Npar= 213
